# Microbial interactions and metabolisms in response to bacterial wilt and black shank pathogens in the tobacco rhizosphere

**DOI:** 10.3389/fpls.2023.1200136

**Published:** 2023-06-20

**Authors:** Qianjun Tang, Tianbo Liu, Kai Teng, Zhipeng Xiao, Hailin Cai, Yunsheng Wang, Yunhua Xiao, Wu Chen

**Affiliations:** ^1^ College of Plant Protection, Hunan Agricultural University, Changsha, China; ^2^ Laboratory of Plant Protection, Hunan Tobacco Science Institute, Changsha, China; ^3^ College of Bioscience and Biotechnology, Hunan Agricultural University, Changsha, China

**Keywords:** bacterial wilt, black shank, actinobacteria, bacterial interaction, biosynthesis of antibiotics

## Abstract

**Background:**

Tobacco bacterial wilt (TBW) and black shank (TBS) are responsible for substantial economic losses worldwide; however, microbial interactions and metabolisms in response to TBW and TBS pathogens in the tobacco rhizosphere remain unclear.

**Methods:**

We explored and compared the response of rhizosphere microbial communities to these two plant diseases with the incidences in moderate and heavy degrees by sequencing of 16S rRNA gene amplicons and bioinformatics analysis.

**Results and discussions:**

We found that the structure of rhizosphere soil bacterial communities was significantly (*p* < 0.05) changed from the incidences of TBW and TBS, which also led to decreased Shannon diversity and Pielou evenness. Compared with the healthy group (CK), the OTUs with significantly (*p* < 0.05) decreased relative abundances were mostly affiliated with Actinobacteria (e.g., *Streptomyces* and *Arthrobacter*) in the diseased groups, and the OTUs with significantly (*p* < 0.05) increased relative abundances were mainly identified as Proteobacteria and Acidobacteria. Also, molecular ecological network analysis showed that the nodes (<467) and links (<641) were decreased in the diseased groups compared with the control group (572; 1056), suggesting that both TBW and TBS weakened bacterial interactions. In addition, the predictive functional analysis indicated that the relative abundance of genes related to the biosynthesis of antibiotics (e.g., ansamycins and streptomycin) was significantly (*p* < 0.05) decreased due to incidences of TBW and TBS, and antimicrobial tests showed that some Actinobacteria strains (e.g., *Streptomyces*) and their secreted antibiotics (e.g., streptomycin) could effectively inhibit the growth of these two pathogens.

## Introduction

Soil-borne diseases caused by bacteria or fungi often occur in agriculture and bring huge economic losses, and plant resistance mechanisms to plant diseases are complex (Deverall, 1987). Many researchers showed that the incidence of plant soil-borne diseases was closely related to plant species and soil environments (Kyselková et al., 2009; Schreiter et al., 2018). For plant autoimmune mechanisms, plants can recognize pathogen-associated molecular patterns and pathogen-delivered effectors to stimulate corresponding responses. Plants can strengthen existing defenses, elicit other defense mechanisms (Gómez-Gómez and Boller, 2002) mediated by secondary signal molecules such as salicylic acid, ethylene, and jasmonic acid, and fine-tune their defense responses (Wang et al., 2002).

The soil environment, including biotic (e.g., populations of bacteria and fungi) and abiotic (e.g., soil type and nutrients) factors, is critical to impacting disease incidence (Choi et al., 2020; Gu et al., 2022; Kim et al., 2022; Ren et al., 2022; Zhang et al., 2022). Microbes in the rhizosphere soil are the first protective screen against plant soil-borne pathogens (Kwak et al., 2018), and they can hinder pathogen growth through competent (e.g., nutrient and space) and antagonistic (e.g., antimicrobial peptide and phenolic acid antibacterial agent) actions. Many microbial ecological agents have recently been isolated from rhizosphere soil samples to control plant diseases (Tan et al., 2021; Wang et al., 2021; Kim et al., 2022). For example, *Bacillus* spp., isolated from wheat soil samples, could effectively reduce the growth of *Fusarium graminearum* strains causing fusarium head blight (Stumbriene et al., 2018), and *Flavobacterium* sp. TRM1, isolated from rhizosphere soil, could suppress *Ralstonia solanacearum* disease development (Kwak et al., 2018). Therefore, it is necessary to understand rhizosphere soil microbial communities and their associations with plant disease incidence.

Some soil-borne diseases often co-occur, making disease management more difficult. Many methods, e.g., promoting plant immune reaction, optimizing agronomic management, and developing biocontrol agents, were studied to prevent disease incidence. The plant immune system is determined by plant genes (Deverall, 1987), which need direct and hard genetic manipulation. The disease inhibiting efficiency of optimizing agronomic management is limited. Therefore, more and more researchers have focused on the beneficial rhizosphere soil or endophytic microbes. The anti-pathogen mechanism of microbes is expected to be complex, especially for different pathogens (Bacon and White, 2016). For example, some antibiotics are broad-spectrum antimicrobials, and some are limited (Yao et al., 2016; Cao et al., 2022). Therefore, high abundances and diverse species of probiotics in the rhizosphere soil may effectively prevent and control multiple diseases. Besides, disease incidence or infection was largely related to indigenous ecological interactions and stability disrupted by pathogens, and in return, close interactions and strong stability of soil microbial communities may be necessary to suppress disease incidences or infections for plants (Miliute et al., 2015; Xiao et al., 2018).

Tobacco (*Nicotiana tobacum*) is an economically important crop worldwide. Tobacco bacterial wilt (TBW) and tobacco black shank (TBS), respectively caused by the bacterial pathogen *Ralstonia solanacearum* (Yin et al., 2022) and fungal pathogen *Phytophthora nicotianae* (Jin and Shew, 2021), are two accompanying of the most devastating soil-borne diseases resulting in severe losses in yield and quality of tobacco. Although many previous studies found a difference in rhizosphere microbial diversity after the infection of pathogenic bacteria and pathogenic fungi, few studies were conducted in the same field, crop, and period. In this study, we explored the composition and interactions of rhizosphere soil microbial communities in response to incidences of TBW and TBS in the same field, crop, and period by sequencing of 16S rRNA gene amplicons. Our results indicated that the diseased rhizosphere microbial communities decreased diversity and interactions as well as decreased functional species for the biosynthesis of antibiotics, leading to incidences of both bacterial and fungal diseases, and these plant pathogens could further weaken community functions. This study provides new insights into our understanding of broad-spectrum Actinobacteria and has important implications for controlling TBW and TBS with microbes.

## Materials and methods

### Rhizosphere soil sampling

Rhizosphere soil samples were collected from a long-term ecological research site for a tobacco (*N. tabacum* cultivar K326) consecutive monoculture experiment located in Xiangxi, Hunan, China. Soil type of this research site was clayey and climate is humid subtropical monsoon. Fertilizer consisted of 50 kg/mu special basal fertilizer, 20 kg/mu special top dressing, 15 kg/mu bioorganic fertilizer, and 5 kg/mu hole-applied fertilizer. Its N:P:K (N, P_2_O_5_, and K_2_O) was 1:1.2:2.43. Transplanted tobacco was irrigated with 300 kg/mu water and 5 kg/mu hole-applied fertilizer. No pest or disease controls was applied during the whole experiment. *N. tabacum* cultivar K326 was introduced by Northup King Seed Company (America) to Yunnan province (China) in 1985. It was now preserved in the Tobacco Research Institute, Chinese Academy of Agricultural Sciences, where we obtained and used seeds with permission.

At the *N. tabacum* harvesting stage in July 2017, we investigated the disease index of each tobacco plant in an area planted with 100 tobacco plants. The bacterial wilt and black shank disease index of each plant were determined based on the Chinese national standard GB/T 23222-2008, as described by Gao et al. (2015), ranging from 0 (lowest) to 9 (highest). In this study, moderate infection with a disease index of 1, 3 and 5, and heavy infection with a disease index of 7 and 9.

We designed five groups: the group with no disease (CK), the group moderately infected by TBW (BWM), the group heavily infected by TBW (BWH), the group moderately infected by TBS (BSM), and the group of heavily infected by TBS (BSH). Eight rhizosphere soil samples were collected using a clean spade for each group following Kwak et al. (2018). The whole roots of each tobacco plant were collected at a soil depth of 0–20 cm. The loose soil particles adhering to the roots were shaken off. Afterward, the roots were placed in a 200-mL tube containing 100 mL of phosphate buffered saline buffer, and washed 3 times with shaking at 180 r/min. Finally, the rhizosphere soils were obtained after centrifuged at 10 000 r/min for 10 min and removing the supernatant. The rhizosphere soil samples were stored at -80°C before DNA extraction.

### DNA extraction, amplification, 16S rRNA gene sequencing and data processing

DNA extraction, amplification, 16S rRNA gene amplicon sequencing, and data processing followed by Xiao et al. (2018). The rhizosphere soil from each root collection was homogenized, and 1.0 g from each sample was used for DNA extraction using a PowerSoil DNA Isolation Kit (MO BIO, San Diego, USA). DNA extracts were purified by electrophoresis on a 0.7% agarose gel using a DNA gel extraction kit (OMEGA, USA). The 16S rRNA gene was amplified with primer pair 515F (5’-GTGCCAGCMGCCGCGGTAA-3’) and 806R (5’-GGACTACHVGGGTWTCTAAT-3’), combined with Illumina adapter sequences, a pad and a linker of two bases, and barcodes on the reverse primer. PCR products were purified, and the concentration was quantified with a NanoDrop ND-1000 spectrophotometer (NanoDrop Technologies, Wilmington, USA). The products were sequenced on a MiSeq machine (Illumina, San Diego, CA) using a 500-cycle kit. All 16S rRNA gene sequences were submitted to the Sequence Read Archive (SRA) of NCBI, and the accession number is PRJNA899683.

### Molecular ecological network construction and characterization

Random matrix theory (RMT)-based approaches were used for network construction (Yin et al., 2015), hub and connector OTU identification and the topological property were determined with a similar threshold (0.95). OTUs, presented in 8 out of 8 replicates, were used for network analysis to ensure data reliability. Various network properties were characterized, such as average degree, average path distance, average clustering coefficient, and modularity index. The network modules were generated using rapid greedy modularity optimization. The experimental data used for constructing phylogenetic molecular ecological networks (pMENs) were based on 16S rRNA gene sequences, and Gephi 0.9.2 software was used to visualize network graphs. The pMENs were constructed separately based on sequencing data of five treatments to reveal the effects of different disease incidences on the microbial network interactions.

### Functional profiling

Prior to functional gene prediction using PICRUSt (phylogenetic investigation of communities by reconstruction of unobserved states) described by Langille et al. (2013), the detected OTUs were reclassified using the GREENGENES reference database. Subsequently, PICRUSt uses 16S rRNA genes to infer metagenome gene functional content from phylogenetic information. The predictions are precalculated for genes in databases, including the Kyoto Encyclopaedia of Genes and Genomes (KEGG). The input data were first normalized by copy number by dividing each OTU by the known 16S rRNA copy number abundance before metagenome predictions and subsequent collapse into functional pathways. The output of PICRUSt consists of a table of functional gene counts as KEGG orthologs (KOs). The Nearest Sequenced Taxon Index (NSTI) value was used to validate the reliability of predicted metagenomes and functional pathways.

### Species isolation and identification

According to data analysis, Actinobacteria could play a key role in inhibiting *P. nicotianae* and *R. solanacearum* growth. We isolated some Actinobacteria strains from the rhizosphere soil of healthy tobacco plants in the above sampling site using GAUZE’s Medium NO.1. *P. nicotianae* HD1 and *R. solanacearum* GMI1000 were obtained originally from diseased *N. tabacum* roots. The single-species antagonism experiments were conducted as the description in our previous study (Liu et al., 2020). And the antagonistic isolates were identified following Ghodhbane-Gtari et al. (2010) based on 16S rRNA gene sequences against the NCBI database. The 16S rDNA sequence was amplified by PCR using primers 27F (5-AGAGTTTGATCACTGGCTCAG-3) and 1492R (5-CGGCTTACCTTGTTACGACTT-3).

### Antimicrobial tests of antibiotics

The antimicrobial effect of several antibiotics was tested by the filtering paper method. Antibiotics included streptomycin, tetracycline and novobiocin, which are biosynthesized by Actinobacteria. The pathogens were *P. nicotianae* (incubated in the PDA medium) and bacterial pathogen *R. solanacearum* (incubated in the LB Agar).

### Pot experiments

The effect of antagonistic strain on TBW and TBS was investigated by a pot experiment in the greenhouse. The cultivated tobacco seedlings (5 to 6 true leaves) were transplanted into pots with sterilized soil. Fifteen days after transplantation, the root was rinsed with the bacterial solution of strain 4-9 (1×10^8^ CFU/mL). The treatment with sterile distilled water replaced bacterial solution was a negative control (CK). Eighteen seedlings of each treatment were repeated 3 times, and 50 mL bacterial solution or sterile distilled water was applied to each plant. 24 h after root irrigation, zoospore/bacterial suspensions were inoculated with a concentration of 1×10^4^/mL (20 mL per plant). The incidence of TBS was observed 30 days after inoculation with *P. nicotiana*, and the incidence of TBW was observed 15 days after inoculation with *R. solanacearum*. Disease classification was according to the Chinese national standard GB/T 23222-2008, further calculating the incidence, disease index and prevention effect.

### Statistical analysis

The community diversity was assessed using the Shannon diversity index (*H*′) and species diversity index. Differences in diversity and relative abundances of bacterial composition based on Tukey’s test were conducted by a one-way analysis of variance (ANOVA) and response ratio analysis (Deng et al., 2012). Detrended correspondence analysis (DCA) and dissimilarity tests were conducted to analyze the bacterial community structure. Linear regression analysis was carried out to explore relationships between disease suppression and microbial diversity (Barberán et al., 2014; Wagg et al., 2014; Cui et al., 2016). All analyses were performed using R v.3.6.3 and STAMP v 2.1.3. The functional/key OTUs were identified following Sheng et al. (2008) by comparing their 16S rRNA gene sequences to the NCBI database, and the phylogenetic tree was constructed using MEGA 5.2 (Kumar et al., 2018).

## Results

### Overview of rhizosphere soil bacterial communities

**Figure 1:**
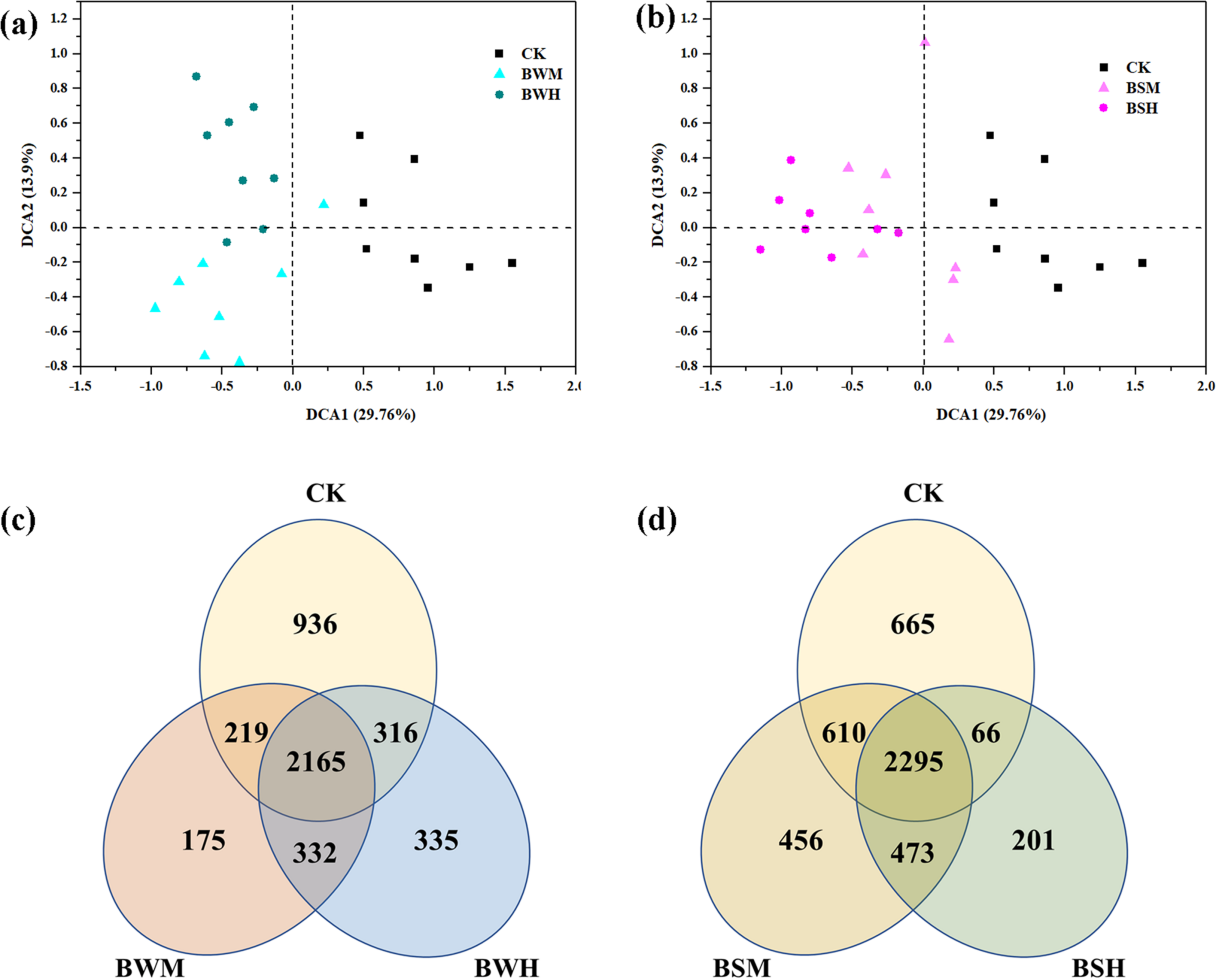
The ordination plots of all samples for the community structure analyzed by detrended correspondence analysis **(A, B)** and Venn diagram **(C, D)**. CK: control group; BWM: the group of moderately infected by TBW; BWH: the group of heavily infected by TBW; BSM: the group of moderately infected by TBS; BSH: the group of heavily infected by TBS.

After clustering at the 97% sequence identity, 4955 OTUs were identified to comprise the soil bacterial community. The α-diversity index, including Shannon diversity and Pielou evenness, were shown in **Table 1**. Compared with the CK group (*H*’: 6.387; *J*’: 0.824), the Shannon diversity index was significantly (*p* < 0.05) decreased in the BWH (5.830), BSM (6.054) and BSH (5.679) groups, and Pielou evenness index was significantly (*p* < 0.05) reduced in the BSH group (0.758). DCA showed that the CK samples were segregated from the BWM and BWH samples (**Figure 1A**), and the BWM and BWH samples were also segregated from each other. Similarly, the CK samples were separated from the BSM and BSH samples (**Figure 1B**). Further dissimilarity tests showed that although the structure of BWM or BSM microbial communities was not significantly (*p* < 0.05) different, the CK group was significantly (*p* < 0.05) different from the other four pathogenic groups, and the heavily infected groups (BWH and BSH) were significantly (*p* < 0.05) different (**Additional file 1**), indicating the structure of rhizosphere soil bacterial communities was influenced by plant diseases at different degrees.

**Table 1:** Shannon diversity and Pielou evenness of microbial communities in the five groups.

Sample	Shannon diversity (H’)	Pielou evenness (J’)
CK	6.387 ± 0.341a	0.824 ± 0.034a
BWM	5.867 ± 0.253ab	0.784 ± 0.031ab
BWH	5.83 ± 0.196b	0.776 ± 0.03ab
BSM	6.054 ± 0.501b	0.783 ± 0.05ab
BSH	5.679 ± 0.311b	0.758 ± 0.036b

“a” and “b” indicated the significant (p < 0.05) difference.

The Venn diagram showed that there were 936, 175 and 335 unique OTUs in the CK, BWM and BWH groups, respectively, and the number of core OTUs was 2165 (**Figure 1C**), occupying the relative abundance up to 99%. Similarly, there were 665, 456 and 201 unique OTUs in the CK, BSM and BSH groups, respectively (**Figure 1D**), and 2295 core OTUs, occupying up to 99% relative abundance.

### The compositions of rhizosphere soil bacterial communities shifted under the incidence of TBW and TBS

**Figure 2:**
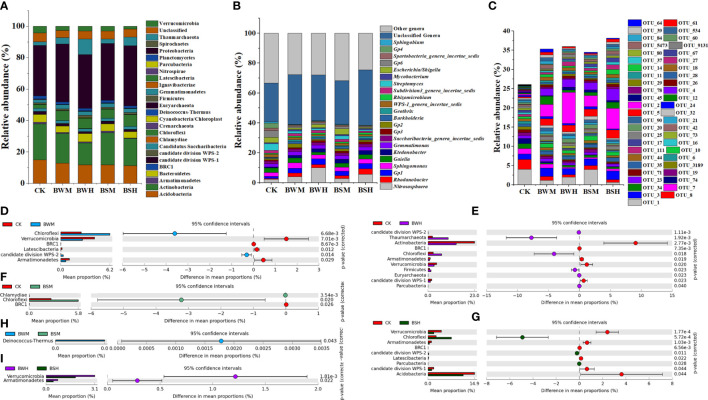
The compositions of microbial communities in phylum **(A)**, genus **(B)**, OTU **(C)** levels, and the significant phyla between each two groups **(D–I)**.

At the phylum level, the bacterial composition of five groups consisted of 26 phyla, which were mostly dominated by Proteobacteria (26.0~53.8%), Actinobacteria (8.0~33.6%), Acidobacteria (5.1~19.8%), Chloroflexi (1.2~15.1%), Bacteroidetes (1.4~9.3%), Thaumarchaeota (0.1~18.6%), and Verrucomicrobia (0.7~7.5%) (**Figure 2A**). However, compared with the CK group, the relative abundances of some phyla were changed under the incidence of plant diseases. ANOVA results showed that the average relative abundances of four phyla (Verrucomicrobia, BRC1, Latescibacteria, and Armatimonadetes) were significantly (*p* < 0.05) lower, and two (Chloroflexi and WPS-2) were significantly (*p* < 0.05) higher in the BWM group (**Figure 2D**); the average relative abundances of five phyla (Actinobacteria, Verrucomicrobia, BRC1, Latescibacteria, and Armatimonadetes) were significantly (*p* < 0.05) lower, and six (Chloroflexi, Thaumarchaeota, Firmicutes, Euryarchaeota, Parcubacteria and WPS-2) were significantly (*p* < 0.05) higher (**Figure 2E**) in the BWH group; the average relative abundance of one phylum (BRC1) were significantly (*p* < 0.05) lower, and two (Chloroflexi and Chlamydiae) were significantly (*p* < 0.05) higher in the BSM group (**Figure 2F**); the average relative abundances of six phyla (Acidobacteria, Verrucomicrobia, BRC1, Latescibacteria, Armatimonadetes and WPS-1) were significantly (*p* < 0.05) lower, and three (Chloroflexi, Parcubacteria and WPS-2) were significantly (*p* < 0.05) higher in the BSH group (**Figure 2G**). Besides, only one phylum showed a significant (*p* < 0.05) difference between BWM and BSM groups and two phyla between BWH and BSH groups (**Figures 2H, I**).

At the genus level (**Figure 2B**), compared with the CK group, the relative abundances of some genera were also changed by plant diseases. ANOVA results showed that: the average relative abundances of 84 genera (e.g., *Gp6*, *Streptomyces* and *Gp4*) were significantly (*p* < 0.05) lower and 43 (e.g., *Rhodanobacter*, *Gp1*, and *Gp2*) were significantly (*p* < 0.05) higher in the BWM group (**Additional file 2**); the average relative abundances of 91 genera (e.g., *Streptomyces*, *Gp6*, and *Gp4*) were significantly (*p* < 0.05) lower and 26 (e.g., *Nitrososphaera*, *Gp1* and *Rhodanobacter*) were significantly (*p* < 0.05) higher in the BWH group (**Additional file 3**); the average relative abundance of 60 genera (e.g., Gp6, Gp4 and *Spartobacteria*_*genera*_*incertae*_*sedis*) were significantly (*p* < 0.05) lower, and 27 (e.g., *Ktedonobacter*, *Geothrix* and *Terriglobus*) were significantly (*p* < 0.05) higher in the BSM group (**Additional file 4**); the average relative abundances of 113 genera (e.g., *Gp6*, *Streptomyces* and *Gp4*) were significantly (*p* < 0.05) lower, and 37 (e.g., *Rhodanobacter*, *Gp1* and *Ktedonobacter*) were significantly (*p* < 0.05) higher in the BSH group (**Additional file 5**).

At the OTU level (**Figure 2C**), compared with the CK group, the composition of the other four groups was significantly (*p* < 0.05) different. ANOVA results showed that: the average relative abundances of 691 OTUs were significantly lower, and 239 were significantly (*p* < 0.05) higher in the BWM group (**Additional file 6**); the average relative abundances of 654 OTUs were significantly (*p* < 0.05) lower, and 197 were significantly (*p* < 0.05) higher in the BWH group (**Additional file 7**); the average relative abundance of 456 OTUs were significantly (*p* < 0.05) lower, and 198 were significantly higher in the BSM group (**Additional file 8**); the average relative abundances of 821 OTUs were significantly (*p* < 0.05) lower, and 128 were significantly (*p* < 0.05) higher in the BSH group (**Additional file 9**).

**Figure 3:**
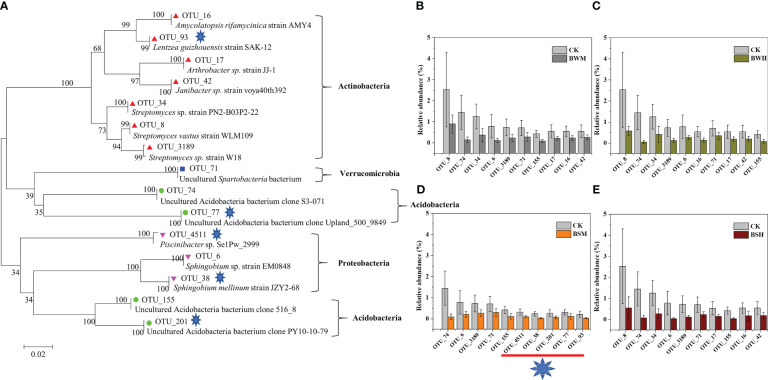
Phylogenetic tree **(A)** of the top 10 decreased OTUs in BWM **(B)**, BWH **(C)**, BSM **(D)**, and BSH **(E)** groups, compared with the CK group.

**Figure 4:**
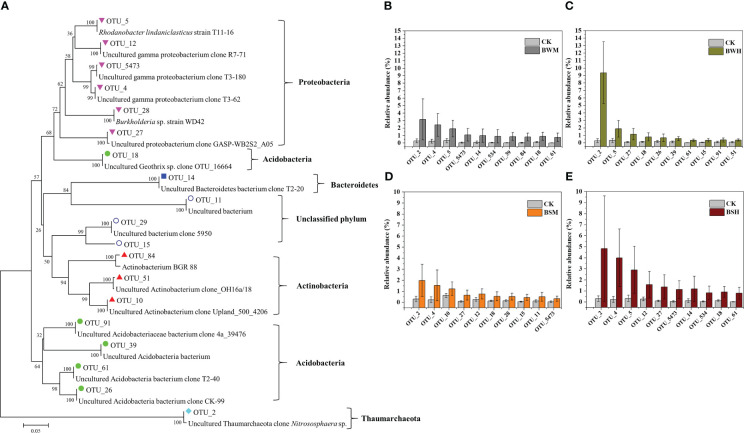
Phylogenetic tree **(A)** of the top 10 increased OTUs in BWM **(B)**, BWH **(C)**, BSM **(D)**, and BSH **(E)** groups, compared with the CK group.

We further aligned the 16S rRNA sequences of top 10 OTUs with the largest decrease (15 OTUs, **Figure 3**) or increase (19 OTUs, **Figure 4**) of relative abundances compared with the CK group to the NCBI database. The top 10 decreased OTUs were identical in the BWM, BWH, and BSH groups (**Figures 3B, C, E**), suggesting their importance in controlling the incidence of TBW and TBS. The phylogenetic tree (**Figure 3A**) indicated that six OTUs (OTU_8, OTU_16, OTU_17, OTU_34, OTU_42, and OTU_3189) were identified as Actinobacteria (*Amycolatopsis*, *Lentzea*, *Arthrobacter*, *Janibacter*, *Streptomyces*), two OTUs (OTU_74 and OTU_155) were identified as Acidobacteria (*Gp4* and *Gp6*), and one OTU (OTU_6) was identified as proteobacteria (*Sphingobium*). In the BSM group, five OTUs were the same as the other three groups, and five were different (**Figure 3D**). The top 10 increased OTUs (19 OTUs in total) were different in the different groups (**Figures 4B–E**), but most of them were identified as Proteobacteria (6 OTUs) and Acidobacteria (5 OTUs) (**Figure 4A**). The relative abundances of OTU_2 (Thaumarchaeota) and OTU_18 (Acidobacteria_*Geothrix*) were increased in the four diseased groups.

### Network interactions of bacterial communities shifted under the incidence of TBW and TBS

**Figure 5:**
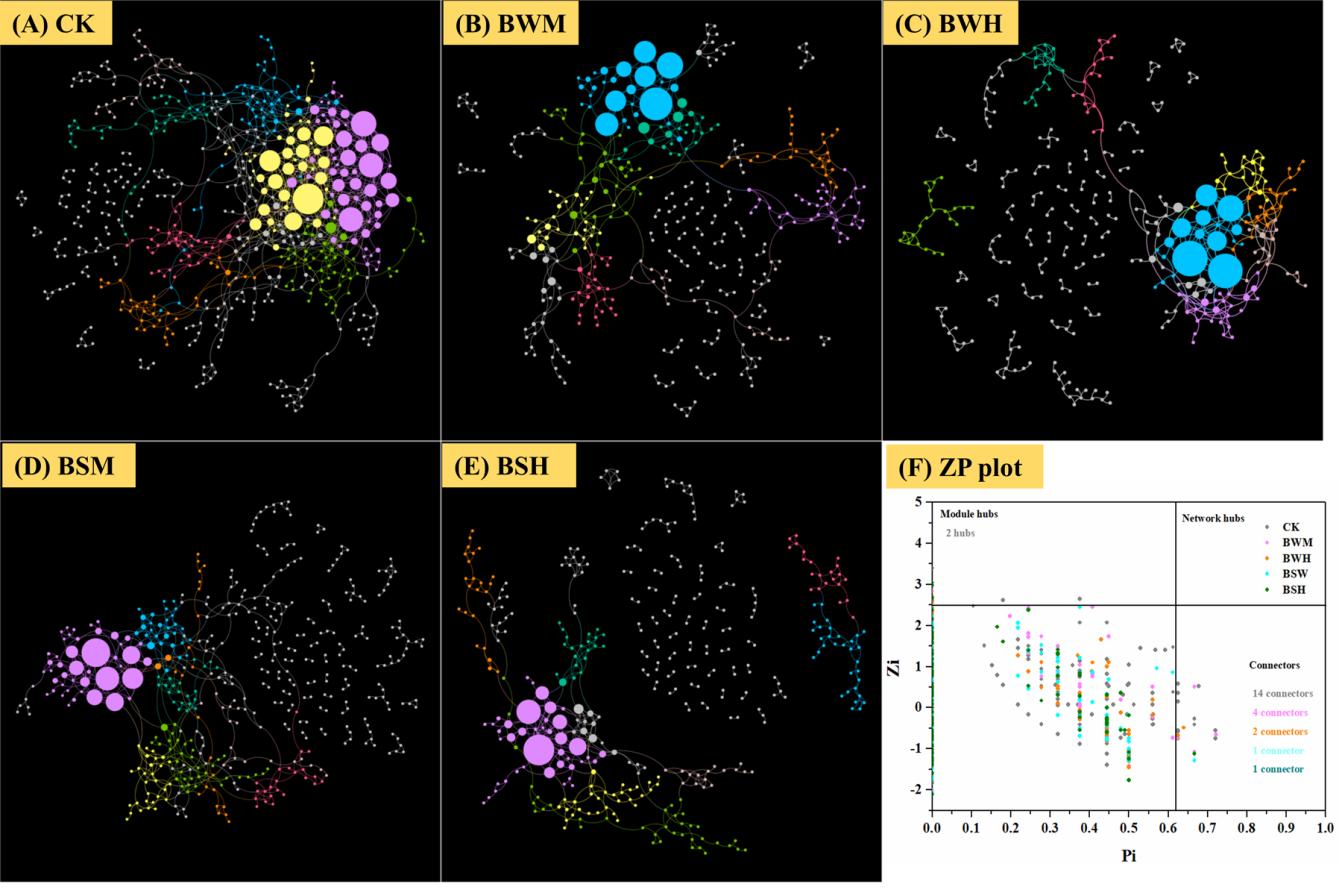
Molecular ecological networks in CK **(A)**, BWM **(B)**, BWH **(C)**, BSM **(D)**, and BSH **(E)** groups, and the *Z*-*P* plot showing the distribution of OTUs based on their topological roles **(F)**. The color of the circle represented the module in the network interaction. The topological role of each OTU was determined according to the scatter plot of within-module connectivity (Zi) and among-module connectivity (Pi).

To discern possible microbial interactions in response to TBW and TBS, molecular ecological networks (MENs) were constructed with 16S rRNA sequencing data using the RMT-based network approaches (**Figures 5A–E**). Major topological properties of empirical MENs of microbial communities in the nine groups were shown in **Table 2**. With the same threshold (0.900), their correlations were more than 0.750, indicating that the degree distributions in both constructed molecular ecological networks fitted the power-law model well. There were more nodes and links in the CK group (572 nodes and 1056 links) than those in the BWM (396 and 521), BWH (346 and 435), BSM (466 and 640), and BSH (392 and 512) groups. It showed interactions of rhizosphere soil microbial communities could be disrupted by pathogens of plant diseases, and the higher degree of plant diseases could lead to more network destruction compared to the CK group.

**Table 2:** Topological properties of the empirical pMENs of soil bacterial communities in five groups.

Network Indexes	CK	BWM	BWH	BSM	BSH
Total nodes	572	396	346	466	392
Total links	1056	521	435	640	512
Threshold	0.900	0.900	0.900	0.900	0.900
R square of power-law	0.84	0.755	0.869	0.82	0.859
moduel	46	49	56	63	61
modurity	0.742	0.846	0.848	0.829	0.859
Average degree (avgK)	3.692	2.631	2.514	2.747	2.612
Average clustering coefficient (avgCC)	0.237	0.201	0.223	0.182	0.229
Average path distance (GD)	7.692	8.381	7.557	7.357	7.609
Connectedness (Con)	0.731	0.527	0.242	0.415	0.31
Efficiency	0.993	0.992	0.98	0.99	0.986

There were also more module hubs (2 hubs) and connectors (14 connectors) in the CK group (**Figure 5F**). We further aligned the 16S rRNA sequences of all hubs and connectors (23 OTUs; 16 in CK; 4 in BWM; 2 in BWH; 1 in BSM; 1 in BSH) to the NCBI database (**Additional file 10**), and found that 11 OTUs were associated with Actinobacteria, and the other 12 OTUs with Proteobacteria (3 OTUs), Acidobacteria (3 OTUs), Verrucomicrobia (2 OTUs), Firmicutes (1 OTU), Gematimonadetes(1 OTU), Armatimonadetes(1 OTU), and unclassified phylum (1 OTU). OTU_48 (Proteobacteria_*Ramlibacter*) was a connector in both CK and BSM groups. Among the 23 OTUs, 14 OTUs (OTU_624, OTU_42, OTU_7584, OTU_2102, OTU_48, OTU_7351, OTU_9474, OTU_631, OTU_483, OTU_38, OTU_96, OTU_48, OTU_279, and OTU_671) were significantly (*p* < 0.05) different between the CK group and the diseased groups, and interestingly, the other 13 OTUs were higher in the CK group except OTU_671 (**Additional file 10**).

### Metabolic diversity

**Figure 6:**
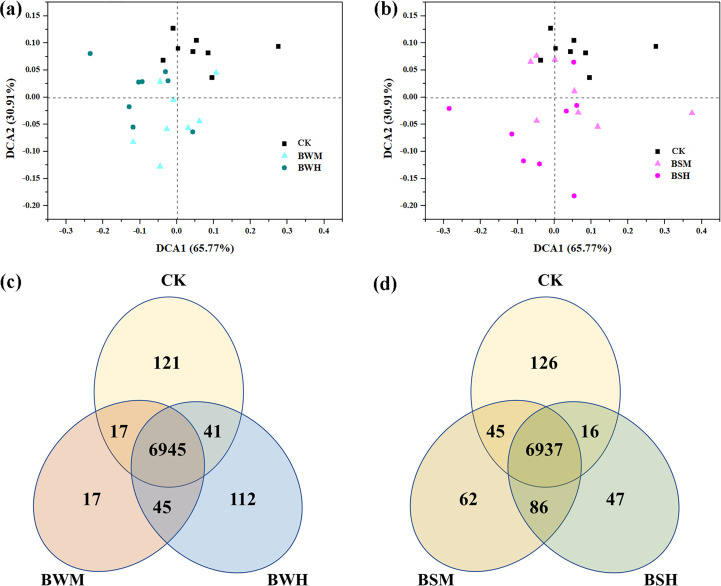
The ordination plots of all samples for the predicted genes analyzed by detrended correspondence analysis **(A, B)** and Venn diagram **(C, D)**.

Based on the 16S rRNA sequences, potential functions were predicted to explore changes of functional diversity and composition by plant pathogens. Although the functional diversity index showed no significant difference among these five groups (**Additional file 11**), DCA results showed that the CK samples were segregated from the diseased samples (**Figures 6A, B**), which was further verified using the dissimilarity tests (**Additional file 12**). There were about 6900 core genes in these groups, and more unique genes (121/126) were in the CK group compared with the other diseased groups (**Figures 6C, D**). Compared with the CK group, the relative abundance of genes related to metabolisms of carbohydrate, terpenoids and polyketides was significantly (*p* < 0.05) decreased in the diseased groups, especially in the BWH group. In contrast, the relative abundance of genes related to cell process (e.g., cell motility) was significantly (*p* < 0.05) increased (**Additional file 13**) in the diseased groups.

We further explored the change in gene abundances related to antibiotics biosynthesis pathways (metabolism of terpenoids and polyketides and biosynthesis of other secondary metabolites). ANOVA results showed that the relative abundances of 6 pathways, including biosynthesis of 12-, 14- and 16-membered macrolides, biosynthesis of ansamycins, polyketide sugar unit biosynthesis, biosynthesis of enediyne antibiotics, streptomycin biosynthesis, and penicillin and cephalosporin biosynthesis, were significantly decreased in all diseased groups (**Table 3**). Besides, 13 pathways (e.g., tetracycline biosynthesis and carbapenem biosynthesis) were also weakened, and three pathways (caffeine metabolism, tropane, piperidine and pyridine alkaloid biosynthesis, and isoflavonoid biosynthesis) were enhanced in individual diseased groups (**Table 3**).

**Table 3:** The key pathways with significant (*p* < 0.05) differences between the CK group and each diseased group.

Metabolism	Pathway	CK vs. BWM (%)	CK vs. BWH (%)	CK vs. BSM (%)	CK vs. BSH (%)
Metabolism of terpenoids and polyketides	Biosynthesis of 12-, 14- and 16-membered macrolides	9.07E-05	1.16E-04	7.45E-05	1.11E-04
	Biosynthesis of type II polyketide backbone	6.10E-03	7.15E-03	–	7.84E-03
	Biosynthesis of ansamycins	7.62E-04	1.35E-03	8.25E-04	1.24E-03
	Tetracycline biosynthesis	1.21E-04	–	–	9.48E-05
	Polyketide sugar unit biosynthesis	2.71E-02	3.09E-02	1.71E-02	3.19E-02
	Biosynthesis of vancomycin group antibiotics	3.00E-03	2.89E-03	–	3.26E-03
	Biosynthesis of enediyne antibiotics	8.15E-04	1.00E-03	7.15E-04	9.47E-04
	Type I polyketide structures	–	1.62E-03	–	1.76E-03
	Insect hormone biosynthesis	5.82E-06	–	5.82E-06	–
	Biosynthesis of type II polyketide products	–	3.00E-03	–	–
	Limonene and pinene degradation	–	1.31E-03	–	–
	Geraniol degradation	–	3.68E-03	–	–
Biosynthesis of other secondary metabolites	Carbapenem biosynthesis	1.07E-03	1.08E-03	–	1.22E-03
	Biosynthesis of secondary metabolites-other antibiotics	1.33E-03	1.57E-03	–	1.67E-03
	Caffeine metabolism	-9.96E-05	–	–	-1.61E-04
	Streptomycin biosynthesis	3.30E-04	3.91E-04	3.09E-04	3.61E-04
	Tropane, piperidine and pyridine alkaloid biosynthesis	-3.83E-03	–	–	-4.86E-03
	Penicillin and cephalosporin biosynthesis	5.90E-03	1.12E-02	7.00E-03	8.02E-03
	Novobiocin biosynthesis	–	5.91E-05	5.34E-05	6.16E-05
	Isoflavonoid biosynthesis	–	–	–	-4.06E-03
	Indole alkaloid biosynthesis	4.91E-06	–	–	5.88E-06
	Phenylpropanoid biosynthesis	1.33E-03	–	–	–
	Betalain biosynthesis	-2.69E-03	–	–	–

CK vs. BWM, BWH, BSM, or BSH indicated the relative abundance of genes in the CK group minus that in the BWM, BWH, BSM, or BSH group, respectively.

### Antagonistic effects of Actinobacteria and inhibition effects of antibiotics

**Figure 7:**
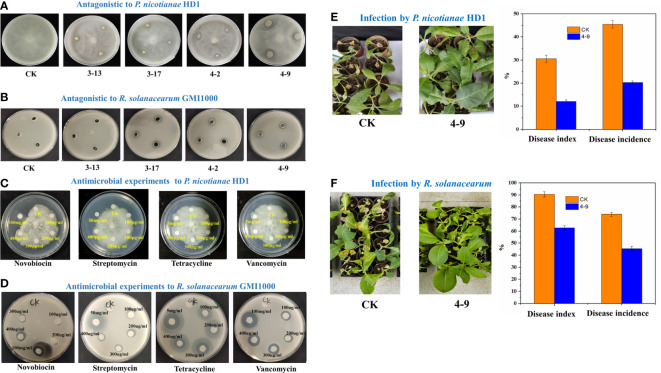
Antagonistic effects. The plate-antagonistic effects of isolated Actinobacteria strains on *P. nicotianae* HD1 **(A)** and *R. solanacearum* GMI1000 **(B)**; The plate-inhibition experiments of some antibiotics on *P. nicotianae* HD1 **(C)** and *R. solanacearum* GMI1000 **(D)**; The pot-antagonistic effects of strain 4-9 on *P. nicotianae* HD1 **(E)** and *R. solanacearum* GMI1000 **(F)**.

Six strains were obtained after isolation on GAUZE’s medium NO. 1 of the rhizosphere soils in healthy tobacco plants. Through antagonism experiments, four strains could inhibit the growth of *P. nicotianae* HD1 (**Figure 7A**). After identification, two strains (3-13 and 3-17) had the highest similarity to *Streptomyces sakaiensis* (99%), one (4-9) similarity to *Streptomyces acidiscabies* (99%), and one (4-2) similarity to *Stenotrophomonas maltophilia* (99%). A further experiment found that three strains could inhibit the growth of *R. solanacearum* GMI1000 (**Figure 7B**).

Filtering paper methods (**Figures 7C, D**) showed that streptomycin (100 μg/ml), tetracycline (100 μg/ml), novobiocin (300 μg/ml) and vancomycin (100 μg/ml) could inhibit the growth of *R. solanacearum* GMI1000, and streptomycin (50 mg/ml) and novobiocin (100 mg/ml) could inhibit the growth of *P. nicotianae* HD1.

Results of the pot experiment indicated that compared with the CK group, the index and incidence of TBW (62.76%; 45.53%) and TBS (12.1%; 20.36%) in the group inoculated with strain 4-9 were significantly decreased (**Figures 7E, F**). The control efficiency of strain 4-9 was 60.40% on TBS and 30.67% on TBW.

## Discussion

Previous studies focused on using microbial antagonists for controlling soil-borne diseases, and many biocontrol agents were found virtually for plant disease biocontrol against a single pathogen (Dunne et al., 1997; Liu et al., 2007; Shanks et al., 2012; Han et al., 2016). However, managing just one of the pathogens may not fully solve the problem since different pathogens often co-occur on the same plant, resulting in a disease complex causing synergistic yield losses (Shen et al., 2022). An environmentally friendly alternative could be using multiple strains with a broad spectrum of antagonism against multiple pathogens, which may provide excellent and new options for soil-borne disease control. TBW is a bacterial disease, and TBS is a fungal disease, which usually results in severe losses of yield and quality of tobacco, so that TBW and TBS can be excellent model for biocontrol research. The first task is to find effectively broad-spectrum microorganisms (Zheng et al., 2021; Shi et al., 2022). In this study, we studied the response of rhizosphere soil microbial communities to these two plant diseases.

The relationship between rhizosphere soil bacterial community composition/structure and crop disease incidence was well studied (Mengesha et al., 2017; Ma et al., 2018; Xiao et al., 2018). It is clear that rhizosphere soil microbial communities generally differ between healthy and diseased plants (Niu et al., 2017; Yang et al., 2017). After plants are infected, the soil microbial diversity and the abundance of probiotics decrease, and microbial interactions could be destroyed (Niu et al., 2017), which is also found in this study. It was also indicated that the change in rhizosphere soil microbial communities was more evident with the increased disease index. Although rhizosphere soil microbial communities showed some differences between different types (caused by fungi or bacteria) of plant diseases, they offered much more similarities. It is hypothesized that bacterial and fungal diseases would have different effects on rhizosphere soil microecology. Indeed, the composition and structure of rhizosphere soil microbial communities were different between the BWH and BSH groups, and three possible mechanisms are: the invasive ability of various pathogens is different; the plant immunocompetence to various pathogens are different; the effective probiotics are different (Horst and Kenneth, 1990; Keen et al., 1993; Ojiambo and Scherm, 2006). However, in our study, many results suggest that after different diseases are outbursts, the rhizosphere soil microbial community alters in a similar direction. The diversity index did not show differences between the TBS and TBW groups (**Table 1**); microbial interactions appeared to be simple; most of the varied populations (e.g., Actinobacteria) were found to be similar; most varied pathways (e.g., biosynthesis of secondary metabolites, biosynthesis of ansamycins) were almost the same among diseased groups.

The resistance mechanisms to plant diseases are complex, mainly depending on the plant-soil-microbe interaction in the soil system (Rasmussen et al., 2019). After the plants were transplanted in the same area, some were healthy, some were lightly diseased, and some were heavily diseased, which seems stochastic. Once pathogens occur in plant tissues, root exudates will be influenced (Gulati et al., 2020). Root exudates have specialized roles in nutrient cycling and signal transduction between a root and its surrounding soil (Jonasson et al., 1999), as well as in plant responses to environmental stresses (Liu et al., 2020). Root exudates play essential roles in the root-root, root-microbe, and microbe-microbe interactions (Bais et al., 2004). Some secretions, e.g., cutin, are screened against plant pathogens (Maurel et al., 1994). The diversity and stability of normal plant flora are other factors to inhibit or kill pathogens (Tilman and Downing, 1994; Tilman et al., 1997; Fitter et al., 2005). Generally, the more closely and stable this system is, the stronger its protective ability is. Also, probiotics and their secondary metabolites play important roles in decreasing the infection of pathogens (Fravel, 1988; Howell, 2003). A well-conditioned cycle should be that normal root exudates improve soil nutrients and soil microecological environments and are beneficial for probiotics to grow, which protects the plant from pathogen infection and accelerates plant growth. Otherwise, a vicious cycle may be that the changed and bad root exudates, as toxic resources, decrease soil nutrients, promote pathogen growth, inhibit probiotics, destroy the stability of soil microecology, and further aggravate the plant disease. Therefore, although the appearance of pathogens or their types is random, the plant-soil-microbe interaction determines the colonization of pathogens and the occurrence of plant diseases.

Analyses of high-throughput sequencing data suggested that Actinobacteria (e.g., *Amycolatopsis*, *Lentzea*, *Arthrobacter Mycobacterium*, and *Streptomyces*) and their biosynthesis of antimicrobial peptides (e.g., ansamycins, novobiocin, streptomycin, tetracycline) played crucial roles in inhibiting both TBS and TBW. We further emphasized that some isolated Actinobacteria and some antimicrobial peptides (e.g., novobiocin, streptomycin) could inhibit both *P. nicotianae* HD1. and *R. solanacearum* GMI1000. The researches for Actinobacteria as biological control agents of plant disease are of interest, given their ability to colonize healthy plant tissues and produce antibiotics *in situ* (Trujillo et al., 2015; Jose et al., 2021; Yadav et al., 2021). For example, *Streptomyces* isolated from healthy banana plants could produce antifungal molecules that inhibited the growth of *Fusarium oxysporum* (Cao et al., 2005). Yacine et al. (2014) found that *Streptomyces* were isolated from tomatoes and native plants of the *Algerian Sahara* and screened for biocontrol activity against *Rhizotocnia solani*. Actinobacteria are the source of two-thirds of naturally derived antibiotics and a range of antihelminthic, antifungal, antibacterial, anticancer, and immunosuppressive drugs (Ventura et al., 2007). The impressive list of important anti-infective compounds synthesized by Actinobacteria includes streptomycin, tetracyclines, chloramphenicol, novobiocin, ansamycins, erythromycin, vancomycin, and kanamycin, and some of these (e.g., ansamycins) are broad-spectrum antibiotics (Mahajan and Balachandran, 2012). Our pot experiment also showed *Streptomyces acidiscabies* 4-9 could effectively control TBW and TBS. Therefore, some broad-spectrum Actinobacteria may be biocontrol agents for both tobacco bacterial and fungal diseases in the future. However, since in the final pot experiments sterilized soil was applied and heavy inoculation with selected Streptomyces strains was used, there is still a long way to a practical application of Streptomyces strains for biocontrol of wilt and black shark diseases of tobacco.

## Conclusions

Compared with healthy plants, the composition and structure of rhizosphere soil microbial communities showed some differences in diseased plants infected with TBW or TBSwith decreased species diversity, destructive networks, and decreased functionality involved in the biosynthesis of antibiotics, which might be the characteristics of the diseased soils. Actinobacteria (e.g., *Amycolatopsis*, *Arthrobacter Mycobacterium, Streptomyces*) were the effective probiotics, and their biosynthesis of antimicrobial peptides (e.g., novobiocin and streptomycin) played crucial roles in controlling the growth of bacterial and fungal pathogens.

## Data availability statement

The data presented in the study are deposited in the Sequence Read Archive (SRA) of NCBI repository, accession number PRJNA899683.

## Author contributions

WC and QT contributed to the study’s conception and design. Research, material preparation, data collection and analysis were performed by TL, KT, ZX, and HC. The first draft of the manuscript was written by YX. TL, QT, YW and WC commented on previous versions of the manuscript. All authors contributed to the article and approved the submitted version. 
