# Supplementary material for: Microbial interactions and metabolisms in response to bacterial wilt and black shank pathogens in the tobacco rhizosphere
Source: Front Plant Sci. 2023 Jun 20;14:1200136. doi: 10.3389/fpls.2023.1200136 (PMC10319149; doi:10.3389/fpls.2023.1200136)
Supplement: Supplementary file 1 [file DataSheet_1.docx]

Additional file 1

Table S1 Dissimilarity tests of bacterial community structure among five groups

| Sample | | BWM | BWH | BSM | BSH |
| --- | --- | --- | --- | --- | --- |
| CK | MRPP^a^ | 0.494*** | 0.49*** | 0.513*** | 0.507*** |
|  | ANONISM^b^ | 0.84*** | 0.855*** | 0523*** | 0.928*** |
|  | ADONIS^c^ | 0.31*** | 0.306*** | 0.2*** | 0.347*** |
| BWM | MRPP |  | 0.493*** | 0.516 | 0.51 |
|  | ANONISM |  | 0.455*** | 0.078 | 0.048 |
|  | ADONIS |  | 0.158*** | 0.088 | 0.064 |
| BWH | MRPP |  |  | 0.512* | 0.506** |
|  | ANONISM |  |  | 0.153* | 0.268** |
|  | ADONIS |  |  | 0.098* | 0.123** |
| BSM | MRPP |  |  |  | 0.529* |
|  | ANONISM |  |  |  | 0.204** |
|  | ADONIS |  |  |  | 0.108** |

^a^Multiple response permutation procedure, a nonparametric procedure that does not depend on assumptions such as normally distributed data or homogeneous variances, but rather depends on the internal variability of the data.

^b^Non-parametric multivariate analysis of variance (MANOVA) with the adonis function.

^c^Analysis of similarities.

All three tests are non-parametric multivariate analyses based on dissimilarities among samples.

*: *p* < 0.1; **: *p* < 0.05; ***: *p* < 0.01.

Additional file 2

Table S2 Genera with significant differences between CK and BWM groups (Unit: %)

| Genus | CK | BWM | Genus | CK | BWM | Genus | CK | BWM |
| --- | --- | --- | --- | --- | --- | --- | --- | --- |
| *Gp6* | 4.541 | 1.138 | *Blastocatella* | 0.045 | 0.006 | *Thalassobacillus* | 0.000 | 0.003 |
| *Streptomyces* | 4.521 | 1.486 | *Actinomadura* | 0.066 | 0.027 | *Gemmobacter* | 0.000 | 0.006 |
| *Gp4* | 3.299 | 0.378 | *Pedomicrobium* | 0.050 | 0.014 | *Asticcacaulis* | 0.003 | 0.009 |
| *Spartobacteria_genera_incertae_sedis* | 1.565 | 0.577 | *Gp22* | 0.038 | 0.002 | *Amnibacterium* | 0.001 | 0.008 |
| *Sphingobium* | 1.023 | 0.159 | *Tepidisphaera* | 0.039 | 0.003 | *Sporosarcina* | 0.002 | 0.010 |
| *Gp7* | 0.708 | 0.201 | *Bosea* | 0.043 | 0.013 | *Pullulanibacillus* | 0.000 | 0.009 |
| *Gp16* | 0.640 | 0.262 | *Caulobacter* | 0.057 | 0.029 | *Actinocatenispora* | 0.000 | 0.013 |
| *Nocardioides* | 0.848 | 0.475 | *Kofleria* | 0.041 | 0.017 | *Clostridium sensu stricto* | 0.003 | 0.017 |
| *Arthrobacter* | 0.660 | 0.331 | *Microvirga* | 0.032 | 0.010 | *Castellaniella* | 0.008 | 0.024 |
| *Janibacter* | 0.545 | 0.256 | *Litorilinea* | 0.024 | 0.003 | *Azospirillum* | 0.002 | 0.019 |
| *Conexibacter* | 0.740 | 0.469 | *Panacagrimonas* | 0.027 | 0.009 | *Ensifer* | 0.006 | 0.024 |
| *Terrimonas* | 0.357 | 0.094 | *Bauldia* | 0.025 | 0.007 | *Niabella* | 0.008 | 0.038 |
| *Unclassified_Planctomycetes* | 0.598 | 0.336 | *Duganella* | 0.020 | 0.004 | *Sphaerobacter* | 0.009 | 0.042 |
| *Aquihabitans* | 0.324 | 0.069 | *Inquilinus* | 0.019 | 0.002 | *Acidisoma* | 0.046 | 0.082 |
| *Rhizobium* | 0.547 | 0.297 | *Methylophilus* | 0.020 | 0.004 | *Candidimonas* | 0.008 | 0.047 |
| *Solirubrobacter* | 0.276 | 0.059 | *Bryobacter* | 0.019 | 0.005 | *Roseiarcus* | 0.011 | 0.051 |
| *Unclassified_Verrucomicrobia* | 0.249 | 0.037 | *Blastopirellula* | 0.014 | 0.000 | *Acidipila* | 0.010 | 0.050 |
| *Aridibacter* | 0.285 | 0.103 | *Saccharothrix* | 0.016 | 0.003 | *Aquisphaera* | 0.038 | 0.084 |
| *Lechevalieria* | 0.217 | 0.046 | *Acidiphilium* | 0.019 | 0.007 | *Acidimicrobium* | 0.005 | 0.056 |
| *Georgfuchsia* | 0.229 | 0.064 | *BRC1_genera_incertae_sedis* | 0.014 | 0.002 | *Gp14* | 0.000 | 0.056 |
| *Arenimonas* | 0.196 | 0.036 | *Actinoplanes* | 0.012 | 0.001 | *Nitrolancea* | 0.023 | 0.080 |
| *Latescibacteria_genera_incertae_sedis* | 0.187 | 0.028 | *Actinocorallia* | 0.009 | 0.000 | *Telmatobacter* | 0.003 | 0.072 |
| *Kribbella* | 0.214 | 0.056 | *Isosphaera* | 0.009 | 0.000 | *Tahibacter* | 0.032 | 0.102 |
| *Pirellula* | 0.210 | 0.052 | *Gp11* | 0.009 | 0.000 | *Acidobacterium* | 0.007 | 0.109 |
| *Armatimonadetes_gp4* | 0.254 | 0.107 | *Enhygromyxa* | 0.009 | 0.000 | *Kerstersia* | 0.002 | 0.132 |
| *Aciditerrimonas* | 0.362 | 0.228 | *Belnapia* | 0.009 | 0.000 | *Acidothermus* | 0.008 | 0.147 |
| *Gp17* | 0.147 | 0.017 | *Rubinisphaera* | 0.008 | 0.001 | *Geodermatophilus* | 0.024 | 0.174 |
| *Armatimonadetes_gp5* | 0.196 | 0.066 | *Chelatococcus* | 0.006 | 0.000 | *Actinospica* | 0.078 | 0.272 |
| *Povalibacter* | 0.151 | 0.035 | *Catelliglobosispora* | 0.009 | 0.002 | *Thermogutta* | 0.107 | 0.313 |
| *Gp10* | 0.136 | 0.035 | *Planctomicrobium* | 0.005 | 0.000 | *Terriglobus* | 0.053 | 0.282 |
| *Pelomonas* | 0.145 | 0.044 | *Peredibacter* | 0.006 | 0.001 | *Lacibacterium* | 0.019 | 0.296 |
| *Ilumatobacter* | 0.117 | 0.019 | *Paracoccus* | 0.005 | 0.000 | *WPS-2_genera_incertae_sedis* | 0.020 | 0.331 |
| *Minicystis* | 0.125 | 0.029 | *Ferrimicrobium* | 0.005 | 0.000 | *Granulicella* | 0.064 | 0.403 |
| *Pseudoduganella* | 0.124 | 0.034 | *Bellilinea* | 0.005 | 0.001 | *Frateuria* | 0.058 | 0.454 |
| *Cupriavidus* | 0.122 | 0.034 | *Actinomycetospora* | 0.006 | 0.002 | *Geothrix* | 0.269 | 1.081 |
| *Ferruginibacter* | 0.115 | 0.033 | *Azonexus* | 0.003 | 0.000 | *Burkholderia* | 0.653 | 1.660 |
| *Variovorax* | 0.151 | 0.076 | *Phycisphaera* | 0.003 | 0.000 | *Gp2* | 0.845 | 2.580 |
| *Parafilimonas* | 0.064 | 0.006 | *Tumebacillus* | 0.003 | 0.000 | *Gp1* | 1.378 | 3.187 |
| *Thermoleophilum* | 0.083 | 0.026 | *Fimbriimonas* | 0.002 | 0.000 | *Rhodanobacter* | 0.537 | 2.351 |
| *Terrabacter* | 0.090 | 0.040 | *Oscillochloris* | 0.002 | 0.000 | *Unclassified_Chloroflexi* | 1.685 | 4.277 |
| *Iamia* | 0.065 | 0.015 | *Hephaestia* | 0.002 | 0.000 | *Unclassified_Proteobacteria* | 11.124 | 15.638 |
| *Altererythrobacter* | 0.077 | 0.028 | *Clostridium XlVa* | 0.000 | 0.002 |  |  |  |
| *Actinophytocola* | 0.051 | 0.006 | *Staphylococcus* | 0.000 | 0.002 |  |  |  |

Additional file 3

Table S3 Genera with significant differences between CK and BWH groups (Unit: %)

| Genus | CK | BWH | Genus | CK | BWH | Genus | CK | BWH |
| --- | --- | --- | --- | --- | --- | --- | --- | --- |
| *Streptomyces* | 4.521 | 1.109 | *Thermoleophilum* | 0.083 | 0.014 | *Chelatococcus* | 0.006 | 0.000 |
| *Gp6* | 4.541 | 1.151 | *Dactylosporangium* | 0.112 | 0.052 | *Sphaerisporangium* | 0.006 | 0.000 |
| *Gp4* | 3.299 | 0.327 | *Iamia* | 0.065 | 0.006 | *Planctomicrobium* | 0.005 | 0.000 |
| *Gaiella* | 3.033 | 1.805 | *Altererythrobacter* | 0.077 | 0.022 | *Peredibacter* | 0.006 | 0.001 |
| *Sphingobium* | 1.023 | 0.287 | *Actinophytocola* | 0.051 | 0.000 | *Paracoccus* | 0.005 | 0.001 |
| *Spartobacteria_genera_incertae_sedis* | 1.565 | 0.837 | *Pedomicrobium* | 0.050 | 0.004 | *Solimonas* | 0.003 | 0.000 |
| *WPS-1_genera_incertae_sedis* | 1.841 | 1.150 | *Phaeodactylibacter* | 0.045 | 0.000 | *Candidatus Brocadia* | 0.003 | 0.000 |
| *Nocardioides* | 0.848 | 0.406 | *Armatimonas/Armatimonadetes_gp1* | 0.076 | 0.034 | *Azonexus* | 0.003 | 0.000 |
| *Conexibacter* | 0.740 | 0.307 | *Asanoa* | 0.042 | 0.002 | *Phycisphaera* | 0.003 | 0.000 |
| *Amycolatopsis* | 0.672 | 0.257 | *Pseudorhodoferax* | 0.082 | 0.041 | *Oscillochloris* | 0.002 | 0.000 |
| *Gp16* | 0.640 | 0.268 | *Blastocatella* | 0.045 | 0.004 | *Hephaestia* | 0.002 | 0.000 |
| *Janibacter* | 0.545 | 0.200 | *Tepidisphaera* | 0.039 | 0.002 | *Desulfosporosinus* | 0.000 | 0.002 |
| *Bradyrhizobium* | 0.784 | 0.507 | *Gp25* | 0.046 | 0.012 | *Brenneria* | 0.000 | 0.005 |
| *Gp7* | 0.708 | 0.452 | *Methylobacterium* | 0.045 | 0.014 | *Parcubacteria_genera_incertae_sedis* | 0.000 | 0.009 |
| *Terrimonas* | 0.357 | 0.102 | *Bacillariophyta* | 0.034 | 0.006 | *Magnetospirillum* | 0.006 | 0.022 |
| *Solirubrobacter* | 0.276 | 0.021 | *Anderseniella* | 0.030 | 0.002 | *Telmatobacter* | 0.003 | 0.019 |
| *Aquihabitans* | 0.324 | 0.082 | *GpXIII* | 0.024 | 0.000 | *Methanocella* | 0.000 | 0.018 |
| *Rhizobium* | 0.547 | 0.317 | *Microvirga* | 0.032 | 0.008 | *Acidipila* | 0.010 | 0.039 |
| *Aridibacter* | 0.285 | 0.062 | *Panacagrimonas* | 0.027 | 0.005 | *Geodermatophilus* | 0.024 | 0.054 |
| *Unclassified_Verrucomicrobia* | 0.249 | 0.048 | *Bosea* | 0.043 | 0.022 | *Acidimicrobium* | 0.005 | 0.037 |
| *Lechevalieria* | 0.217 | 0.017 | *Hamadaea* | 0.024 | 0.004 | *Acidobacterium* | 0.007 | 0.041 |
| *Aciditerrimonas* | 0.362 | 0.181 | *Microbacterium* | 0.030 | 0.012 | *Aquabacterium* | 0.010 | 0.046 |
| *Kribbella* | 0.214 | 0.041 | *Saccharopolyspora* | 0.020 | 0.002 | *Nevskia* | 0.025 | 0.067 |
| *Armatimonadetes_gp4* | 0.254 | 0.100 | *Bryobacter* | 0.019 | 0.002 | *Clostridium sensu stricto* | 0.003 | 0.070 |
| *Pirellula* | 0.210 | 0.058 | *Methylophilus* | 0.020 | 0.004 | *Lacibacterium* | 0.019 | 0.097 |
| *Ramlibacter* | 0.376 | 0.227 | *Inquilinus* | 0.019 | 0.003 | *Kerstersia* | 0.002 | 0.081 |
| *Phycicoccus* | 0.150 | 0.022 | *Bauldia* | 0.025 | 0.011 | *Granulicella* | 0.064 | 0.149 |
| *Arenimonas* | 0.196 | 0.071 | *Saccharothrix* | 0.016 | 0.003 | *WPS-2_genera_incertae_sedis* | 0.020 | 0.108 |
| *Gp17* | 0.147 | 0.023 | *Blastopirellula* | 0.014 | 0.002 | *Thermogutta* | 0.107 | 0.207 |
| *Flavisolibacter* | 0.302 | 0.191 | *BRC1_genera_incertae_sedis* | 0.014 | 0.002 | *Terriglobus* | 0.053 | 0.166 |
| *Rhodococcus* | 0.196 | 0.086 | *Crossiella* | 0.013 | 0.000 | *Actinospica* | 0.078 | 0.222 |
| *Gp10* | 0.136 | 0.028 | *Agromyces* | 0.011 | 0.000 | *Unclassified_Firmicutes* | 0.406 | 1.032 |
| *Ilumatobacter* | 0.117 | 0.012 | *Noviherbaspirillum* | 0.015 | 0.005 | *Geothrix* | 0.269 | 1.146 |
| *Blastococcus* | 0.137 | 0.037 | *Cellulomonas* | 0.012 | 0.002 | *Rhodanobacter* | 0.537 | 2.160 |
| *Minicystis* | 0.125 | 0.033 | *Geminicoccus* | 0.009 | 0.000 | *Unclassified_Chloroflexi* | 1.685 | 4.059 |
| *Armatimonadetes_gp5* | 0.196 | 0.106 | *Actinocorallia* | 0.009 | 0.000 | *Gp1* | 1.378 | 3.944 |
| *Pseudonocardia* | 0.131 | 0.049 | *Gp11* | 0.009 | 0.000 | *Nitrososphaera* | 2.208 | 9.960 |
| *Micromonospora* | 0.142 | 0.061 | *Catelliglobosispora* | 0.009 | 0.000 | *Isosphaera* | 0.009 | 0.002 |
| *Ferruginibacter* | 0.115 | 0.039 | *Enhygromyxa* | 0.009 | 0.001 | *Cupriavidus* | 0.122 | 0.047 |

Additional file 4

Table S4 Genera with significant differences between CK and BSM groups (Unit: %)

| Genus | CK | BSM | Genus | CK | BSM | Genus | CK | BSM |
| --- | --- | --- | --- | --- | --- | --- | --- | --- |
| *Gp6* | 4.541 | 1.429 | *Asanoa* | 0.042 | 0.000 | *Oscillochloris* | 0.002 | 0.000 |
| *Gp4* | 3.299 | 0.497 | *Blastocatella* | 0.045 | 0.006 | *Hephaestia* | 0.002 | 0.000 |
| *Spartobacteria_genera_incertae_sedis* | 1.565 | 0.767 | *Armatimonas/Armatimonadetes_gp1* | 0.076 | 0.045 | *Methanosarcina* | 0.000 | 0.001 |
| *Sphingobium* | 1.023 | 0.245 | *Pseudorhodoferax* | 0.082 | 0.051 | *Sporomusa* | 0.000 | 0.001 |
| *Gp16* | 0.640 | 0.319 | *Virgisporangium* | 0.038 | 0.008 | *Romboutsia* | 0.000 | 0.002 |
| *Solirubrobacter* | 0.276 | 0.032 | *Anderseniella* | 0.030 | 0.003 | *Unclassified_Chlamydiae* | 0.000 | 0.002 |
| *Terrimonas* | 0.357 | 0.118 | *Caulobacter* | 0.057 | 0.031 | *Methylococcus* | 0.000 | 0.002 |
| *Conexibacter* | 0.740 | 0.506 | *Microvirga* | 0.032 | 0.006 | *Gp15* | 0.000 | 0.003 |
| *Aquihabitans* | 0.324 | 0.125 | *Bryobacter* | 0.019 | 0.004 | *Parvibaculum* | 0.000 | 0.004 |
| *Lechevalieria* | 0.217 | 0.029 | *Panacagrimonas* | 0.027 | 0.012 | *Simkania* | 0.005 | 0.017 |
| *Aridibacter* | 0.285 | 0.117 | *Litorilinea* | 0.024 | 0.009 | *Azospirillum* | 0.002 | 0.018 |
| *Unclassified_Verrucomicrobia* | 0.249 | 0.083 | *Bauldia* | 0.025 | 0.012 | *Gp14* | 0.000 | 0.017 |
| *Ramlibacter* | 0.376 | 0.242 | *Noviherbaspirillum* | 0.015 | 0.002 | *Clostridium sensu stricto* | 0.003 | 0.036 |
| *Georgfuchsia* | 0.229 | 0.096 | *Saccharothrix* | 0.016 | 0.005 | *Acidipila* | 0.010 | 0.046 |
| *Armatimonadetes_gp4* | 0.254 | 0.126 | *Actinoplanes* | 0.012 | 0.001 | *Acidobacterium* | 0.007 | 0.057 |
| *Pirellula* | 0.210 | 0.094 | *BRC1_genera_incertae_sedis* | 0.014 | 0.004 | *Acidothermus* | 0.008 | 0.058 |
| *Arenimonas* | 0.196 | 0.085 | *Blastopirellula* | 0.014 | 0.004 | *Acidimicrobium* | 0.005 | 0.062 |
| *Gp17* | 0.147 | 0.046 | *Geminicoccus* | 0.009 | 0.000 | *Sphingobacterium* | 0.051 | 0.109 |
| *Gp10* | 0.136 | 0.043 | *Crossiella* | 0.013 | 0.004 | *Kerstersia* | 0.002 | 0.067 |
| *Minicystis* | 0.125 | 0.035 | *Catelliglobosispora* | 0.009 | 0.001 | *Lacibacterium* | 0.019 | 0.097 |
| *Blastococcus* | 0.137 | 0.057 | *Enhygromyxa* | 0.009 | 0.001 | *Rudaea* | 0.154 | 0.243 |
| *Pelomonas* | 0.145 | 0.079 | *Cytophaga* | 0.008 | 0.000 | *Tahibacter* | 0.032 | 0.131 |
| *Ferruginibacter* | 0.115 | 0.053 | *Actinocorallia* | 0.009 | 0.002 | *Thermogutta* | 0.107 | 0.218 |
| *Pseudoduganella* | 0.124 | 0.065 | *Cellulomonas* | 0.012 | 0.004 | *Granulicella* | 0.064 | 0.180 |
| *Terrimicrobium* | 0.068 | 0.016 | *Chelatococcus* | 0.006 | 0.000 | *Chryseobacterium* | 0.075 | 0.263 |
| *Thermoleophilum* | 0.083 | 0.031 | *Sphaerisporangium* | 0.006 | 0.001 | *Terriglobus* | 0.053 | 0.266 |
| *Altererythrobacter* | 0.077 | 0.026 | *Planctomicrobium* | 0.005 | 0.000 | *Geothrix* | 0.269 | 0.744 |
| *Iamia* | 0.065 | 0.017 | *Actinomycetospora* | 0.006 | 0.003 | *Unclassified_Chloroflexi* | 1.685 | 2.822 |
| *Pedomicrobium* | 0.050 | 0.007 | *Phycisphaera* | 0.003 | 0.000 | *Ktedonobacter* | 0.814 | 2.648 |

Additional file 5

Table S5 Genera with significant differences between CK and BSH groups (Unit: %)

| Genus | CK | BSH | Genus | CK | BSH | Genus | CK | BSH |
| --- | --- | --- | --- | --- | --- | --- | --- | --- |
| *Gp4* | 3.299 | 0.242 | *Bosea* | 0.043 | 0.014 | *Parcubacteria_genera_incertae_sedis* | 0.000 | 0.017 |
| *Kribbella* | 0.214 | 0.039 | *Minicystis* | 0.125 | 0.010 | *Humibacter* | 0.025 | 0.097 |
| *Pseudorhodoferax* | 0.082 | 0.024 | *Unclassified_Planctomycetes* | 0.598 | 0.246 | *Gp1* | 1.378 | 3.392 |
| *Aquihabitans* | 0.324 | 0.054 | *Lechevalieria* | 0.217 | 0.020 | *Byssovorax* | 0.008 | 0.000 |
| *Pelomonas* | 0.145 | 0.022 | *Virgisporangium* | 0.038 | 0.009 | *Chelatococcus* | 0.006 | 0.000 |
| *Mizugakiibacter* | 0.023 | 0.240 | *Rubinisphaera* | 0.008 | 0.000 | *Parvibaculum* | 0.000 | 0.003 |
| *Gp16* | 0.640 | 0.210 | *Ktedonobacter* | 0.814 | 2.621 | *Phycicoccus* | 0.150 | 0.028 |
| *Saccharothrix* | 0.016 | 0.000 | *Ferruginibacter* | 0.115 | 0.034 | *Lysobacter* | 0.132 | 0.030 |
| *Iamia* | 0.065 | 0.008 | *Acidobacterium* | 0.007 | 0.152 | *Parafilimonas* | 0.064 | 0.006 |
| *Armatimonadetes_gp4* | 0.254 | 0.057 | *Candidimonas* | 0.008 | 0.067 | *Skermanella* | 0.074 | 0.252 |
| *Armatimonadetes_gp5* | 0.196 | 0.051 | *Gp10* | 0.136 | 0.020 | *Pullulanibacillus* | 0.000 | 0.009 |
| *Pseudoduganella* | 0.124 | 0.037 | *Novosphingobium* | 0.114 | 0.017 | *Paracoccus* | 0.005 | 0.001 |
| *Clostridium sensu stricto* | 0.003 | 0.028 | *Unclassified_Chloroflexi* | 1.685 | 4.624 | *Rhodococcus* | 0.196 | 0.083 |
| *Gp6* | 4.541 | 0.597 | *Bauldia* | 0.025 | 0.009 | *Ilumatobacter* | 0.117 | 0.009 |
| *Frateuria* | 0.058 | 0.464 | *WPS-2_genera_incertae_sedis* | 0.020 | 0.226 | *Nitrospirillum* | 0.000 | 0.007 |
| *Kerstersia* | 0.002 | 0.124 | *Actinophytocola* | 0.051 | 0.002 | *Niastella* | 0.192 | 0.072 |
| *Blastopirellula* | 0.014 | 0.001 | *Aciditerrimonas* | 0.362 | 0.216 | *Enhygromyxa* | 0.009 | 0.001 |
| *Pirellula* | 0.210 | 0.029 | *Blastocatella* | 0.045 | 0.000 | *Tepidisphaera* | 0.039 | 0.002 |
| *Acidisoma* | 0.046 | 0.097 | *Gp17* | 0.147 | 0.024 | *Armatimonas/Armatimonadetes_gp1* | 0.076 | 0.042 |
| *Planctomicrobium* | 0.005 | 0.000 | *Lacibacterium* | 0.019 | 0.350 | *Actinoplanes* | 0.012 | 0.001 |
| *Ramlibacter* | 0.376 | 0.158 | *Chondromyces* | 0.063 | 0.024 | *Allokutzneria* | 0.020 | 0.001 |
| *Rhizobium* | 0.547 | 0.172 | *Altererythrobacter* | 0.077 | 0.015 | *Gp22* | 0.038 | 0.001 |
| *Bryobacter* | 0.019 | 0.000 | *Actinospica* | 0.078 | 0.385 | *Pseudonocardia* | 0.131 | 0.051 |
| *Opitutus* | 0.378 | 0.173 | *Ferrimicrobium* | 0.005 | 0.000 | *Fimbriimonas* | 0.002 | 0.000 |
| *Gp7* | 0.708 | 0.297 | *Acidothermus* | 0.008 | 0.140 | *Kofleria* | 0.041 | 0.019 |
| *Sediminibacterium* | 0.081 | 0.031 | *Dactylosporangium* | 0.112 | 0.049 | *Terrimicrobium* | 0.068 | 0.013 |
| *Variovorax* | 0.151 | 0.025 | *Isosphaera* | 0.009 | 0.001 | *GpXIII* | 0.024 | 0.000 |
| *Spartobacteria_genera_incertae_sedis* | 1.565 | 0.455 | *Rhodanobacter* | 0.537 | 3.785 | *Azonexus* | 0.003 | 0.000 |
| *Panacagrimonas* | 0.027 | 0.004 | *Janibacter* | 0.545 | 0.184 | *Terrabacter* | 0.090 | 0.044 |
| *Georgfuchsia* | 0.229 | 0.047 | *Actinocorallia* | 0.009 | 0.000 | *Blastochloris* | 0.062 | 0.021 |
| *Noviherbaspirillum* | 0.015 | 0.003 | *Arthrobacter* | 0.660 | 0.267 | *Phycisphaera* | 0.003 | 0.000 |
| *Bellilinea* | 0.005 | 0.000 | *Acidipila* | 0.010 | 0.064 | *Solimonas* | 0.003 | 0.000 |
| *Terriglobus* | 0.053 | 0.342 | *Jahnella* | 0.036 | 0.006 | *Cystobacter* | 0.004 | 0.002 |
| *Sphaerobacter* | 0.009 | 0.047 | *Roseiarcus* | 0.011 | 0.056 | *Geminicoccus* | 0.009 | 0.000 |
| *Arenimonas* | 0.196 | 0.045 | *Latescibacteria_genera_incertae_sedis* | 0.187 | 0.046 | *Massilia* | 0.239 | 0.111 |
| *Unclassified_Verrucomicrobia* | 0.249 | 0.021 | *Gp25* | 0.046 | 0.013 | *Povalibacter* | 0.151 | 0.057 |
| *Actinomycetospora* | 0.006 | 0.002 | *Granulicella* | 0.064 | 0.198 | *WPS-1_genera_incertae_sedis* | 1.841 | 1.189 |
| *Pedomicrobium* | 0.050 | 0.008 | *Conexibacter* | 0.740 | 0.400 | *Sandaracinus* | 0.027 | 0.001 |
| *Geothrix* | 0.269 | 1.193 | *Unclassified_Bacteroidetes* | 0.972 | 2.449 | *Phaeodactylibacter* | 0.045 | 0.001 |
| *Gp5* | 0.194 | 0.043 | *Gp14* | 0.000 | 0.111 | *Acidimicrobium* | 0.005 | 0.041 |
| *Unclassified_Proteobacteria* | 11.124 | 17.881 | *Saccharopolyspora* | 0.020 | 0.006 | *Blastococcus* | 0.137 | 0.064 |
| *Sphingobium* | 1.023 | 0.046 | *Hamadaea* | 0.024 | 0.005 | *Luteolibacter* | 0.038 | 0.004 |
| *Terrimonas* | 0.357 | 0.045 | *Anderseniella* | 0.030 | 0.002 | *Candidatus Brocadia* | 0.003 | 0.000 |
| *Aridibacter* | 0.285 | 0.042 | *Nevskia* | 0.025 | 0.098 | *Catelliglobosispora* | 0.009 | 0.002 |
| *BRC1_genera_incertae_sedis* | 0.014 | 0.002 | *Inquilinus* | 0.019 | 0.000 | *Sinosporangium* | 0.065 | 0.136 |
| *Streptomyces* | 4.521 | 0.938 | *Methylophilus* | 0.020 | 0.004 | *Roseomonas* | 0.007 | 0.000 |
| *Methylobacterium* | 0.045 | 0.006 | *Thermogutta* | 0.107 | 0.339 | *Oscillochloris* | 0.002 | 0.000 |
| *Subdivision3_genera_incertae_sedis* | 1.921 | 1.073 | *Thalassobacillus* | 0.000 | 0.003 | *Hephaestia* | 0.002 | 0.000 |
| *Telmatobacter* | 0.003 | 0.044 | *Luteimonas* | 0.194 | 0.054 | *Azospirillum* | 0.002 | 0.019 |
| *Litorilinea* | 0.024 | 0.006 | *Solirubrobacter* | 0.276 | 0.048 | *Flavisolibacter* | 0.302 | 0.187 |

Additional file 10


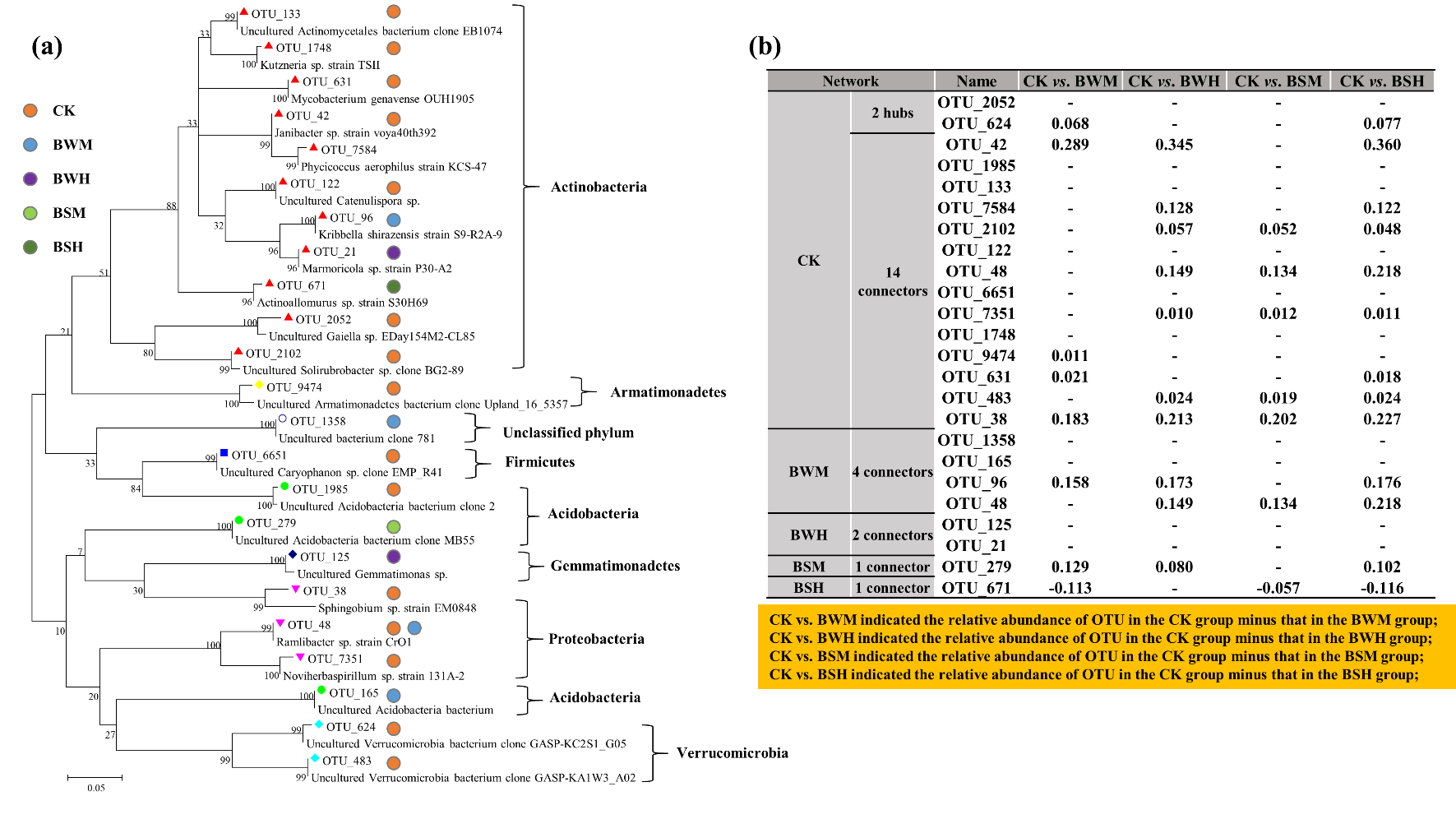


Fig. S1 Key OTUs in ecological network

Additional file 11

Table S10 Gene diversity of microbial community in the five groups.

| Sample | Shannon diversity (H') | Pielou evenness (J') |
| --- | --- | --- |
| CK | 7.356±0.139a | 0.832±0.015a |
| BWM | 7.377±0.100a | 0.837±0.012a |
| BWH | 7.354±0.073a | 0.832±0.008a |
| BSM | 7.340±0.122a | 0.831±0.014a |
| BSH | 7.264±0.206a | 0.824±0.024a |

“a” indicated no significant (p < 0.05) difference between different groups.

Additional file 12

Table S11 Dissimilarity tests of bacterial community structure among five treatments.

| Sample | | BWM | BWH | BSM | BSH |
| --- | --- | --- | --- | --- | --- |
| CK | MRPP^a^ | 0.064*** | 0.066*** | 0.074 | 0.075*** |
|  | ANONISM^b^ | 0.345*** | 0.536*** | 0.049 | 0.357*** |
|  | ADONIS^c^ | 0.226*** | 0.274*** | 0.088 | 0.227*** |
| BWM | MRPP |  | 0.065** | 0.073 | 0.074 |
|  | ANONISM |  | 0.261** | 0.008 | 0.031 |
|  | ADONIS |  | 0.167** | 0.051 | 0.035 |
| BWH | MRPP |  |  | 0.065** | 0.073** |
|  | ANONISM |  |  | 0.197** | 0.192** |
|  | ADONIS |  |  | 0.166* | 0.142* |
| BSM | MRPP |  |  |  | 0.075 |
|  | ANONISM |  |  |  | 0.03 |
|  | ADONIS |  |  |  | 0.094 |

^a^Mutiple response permutation procedure, a nonparametric procedure that does not depend on assumptions such as normally distributed data or homogeneous variances, but rather depends on the internal variability of the data.

^b^Non-parametric multivariate analysis of variance (MANOVA) with the adonis function.

^c^Analysis of similarities.

All three tests are non-parametric multivariate analyses based on dissimilarities among samples.

*: *p* < 0.1; **: *p* < 0.05; ***: *p* < 0.01.

Additional file 13


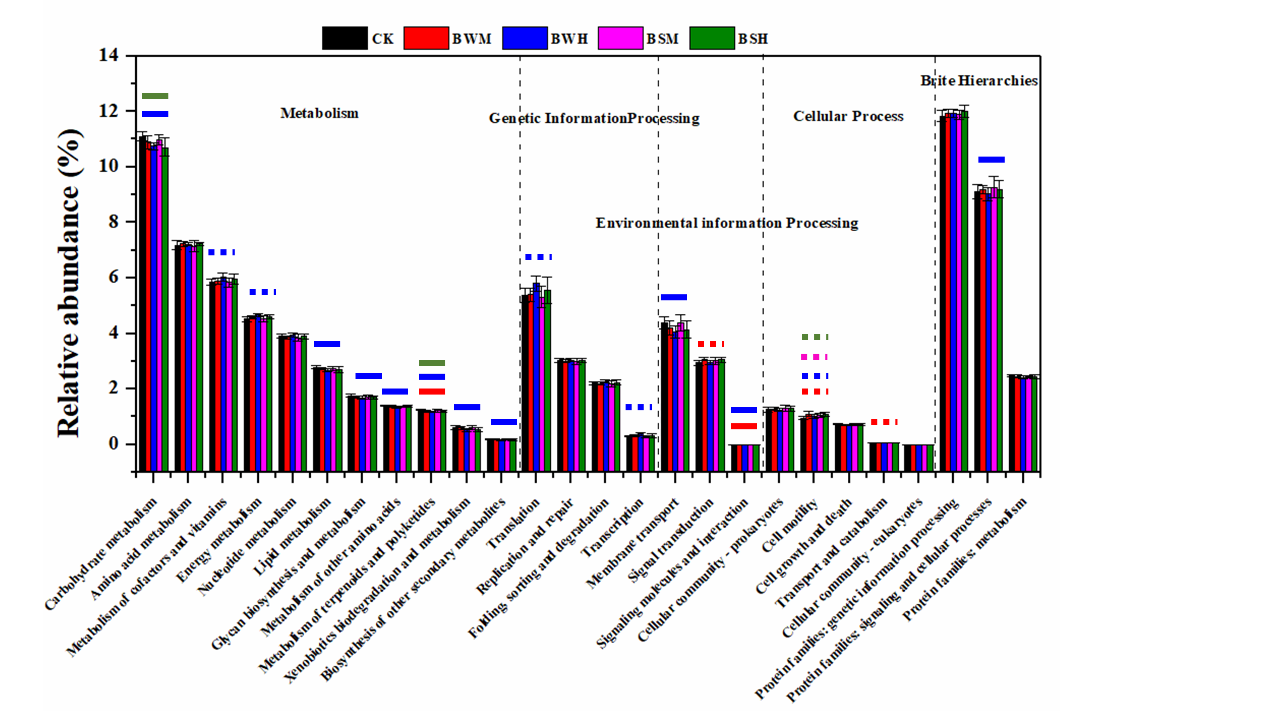


Fig. S2 The relative abundances of predicting-function genes in the main metabolisms
