# Supplementary material for: Microbial interactions and metabolisms in response to bacterial wilt and black shank pathogens in the tobacco rhizosphere
Source: Front Plant Sci. 2023 Jun 20;14:1200136. doi: 10.3389/fpls.2023.1200136 (PMC10319149; doi:10.3389/fpls.2023.1200136)
Supplement: Supplementary file 2 [file DataSheet_2.pdf]

Additional file 6

Table S6 Genera with significant differences between CK and BWM groups (Unit: %)

Domain	Phylum	Genus	OTU	CK: mean ı	CK: std. de	BWM: mea	BWM: std.	p-values
Bacteria	Actinobact	Streptomyc	OTU_8	2.532	1.773	0.892	0.433	0.045
Bacteria	Acidobacte	Gp4	OTU_74	1.449	0.811	0.136	0.136	0.003
Bacteria	Actinobact	Streptomyc	OTU_34	1.255	0.588	0.365	0.324	0.005
Bacteria	Proteobact	Sphingobiu	OTU_6	0.782	0.557	0.105	0.094	0.015
Bacteria	Actinobact	Streptomyc	OTU_3189	0.730	0.399	0.224	0.179	0.013
Bacteria	Verrucomi	Spartobact	OTU_71	0.705	0.366	0.276	0.216	0.021
Bacteria	Acidobacte	Gp6	OTU_155	0.422	0.177	0.076	0.069	0.001
Bacteria	Actinobact	Arthrobact	OTU_17	0.540	0.313	0.208	0.095	0.027
Bacteria	Actinobact	Amycolato	OTU_16	0.545	0.247	0.222	0.128	0.011
Bacteria	Actinobact	Janibacter	OTU_42	0.545	0.303	0.256	0.119	0.043
Bacteria	Unclassifie	Unclassifie	OTU_30	0.394	0.268	0.123	0.098	0.034
Bacteria	Proteobact	Unclassifie	OTU_4511	0.319	0.146	0.053	0.029	0.002
Bacteria	Actinobact	Marmorico	OTU_2959	0.404	0.151	0.148	0.068	0.002
Bacteria	Acidobacte	Gp6	OTU_70	0.285	0.142	0.074	0.058	0.005
Bacteria	Acidobacte	Gp16	OTU_77	0.306	0.124	0.098	0.071	0.003
Bacteria	Acidobacte	Gp6	OTU_201	0.261	0.167	0.062	0.085	0.018
Bacteria	Proteobact	Unclassifie	OTU_134	0.216	0.136	0.021	0.016	0.007
Bacteria	Acidobacte	Gp6	OTU_332	0.216	0.175	0.032	0.029	0.027
Bacteria	Proteobact	Sphingobiu	OTU_38	0.235	0.100	0.052	0.058	0.001
Bacteria	Acidobacte	Gp4	OTU_285	0.222	0.103	0.043	0.041	0.002
Bacteria	Acidobacte	Gp6	OTU_8332	0.215	0.065	0.037	0.026	0.000
Bacteria	Actinobact	Lechevalie	OTU_93	0.217	0.145	0.046	0.027	0.016
Bacteria	Proteobact	Georgfuchs	OTU_277	0.229	0.113	0.064	0.055	0.006
Bacteria	Acidobacte	Gp6	OTU_118	0.235	0.090	0.072	0.063	0.002
Bacteria	Actinobact	Kribbella	OTU_96	0.214	0.071	0.056	0.043	0.000
Bacteria	Candidatus	Saccharibа	OTU_76	0.174	0.145	0.017	0.039	0.024
Bacteria	Acidobacte	Gp7	OTU_2456	0.186	0.089	0.030	0.033	0.002
Bacteria	Proteobact	Unclassifie	OTU_202	0.174	0.106	0.018	0.032	0.006
Bacteria	Acidobacte	Gp4	OTU_1411	0.170	0.051	0.014	0.014	0.000
Bacteria	Unclassifie	Unclassifie	OTU_132	0.224	0.144	0.073	0.086	0.035
Bacteria	Actinobact	Unclassifie	OTU_249	0.199	0.060	0.050	0.043	0.000
Bacteria	Verrucomi	Subdivisio	OTU_207	0.167	0.147	0.020	0.039	0.033
Bacteria	Acidobacte	Gp4	OTU_295	0.168	0.128	0.022	0.032	0.020
Bacteria	Acidobacte	Gp4	OTU_3764	0.146	0.099	0.007	0.011	0.007
Bacteria	Proteobact	Arenimona	OTU_130	0.170	0.067	0.033	0.022	0.001
Bacteria	Acidobacte	Gp6	OTU_508	0.157	0.123	0.021	0.015	0.022
Bacteria	Proteobact	Unclassifie	OTU_106	0.232	0.120	0.098	0.072	0.027
Bacteria	Gemmatim	Gemmatim	OTU_46	0.180	0.137	0.046	0.059	0.040
Bacteria	Acidobacte	Gp7	OTU_279	0.183	0.074	0.055	0.029	0.002
Bacteria	Actinobact	Unclassifie	OTU_8280	0.175	0.133	0.050	0.025	0.043
Bacteria	Proteobact	Unclassifie	OTU_162	0.306	0.095	0.187	0.104	0.043
Bacteria	Proteobact	Unclassifie	OTU_341	0.155	0.081	0.041	0.037	0.007
Bacteria	Acidobacte	Gp4	OTU_2467	0.123	0.061	0.012	0.014	0.002
Bacteria	Verrucomi	Spartobact	OTU_432	0.154	0.107	0.045	0.039	0.033
Bacteria	Proteobact	Unclassifie	OTU_443	0.248	0.054	0.141	0.081	0.012
Bacteria	Acidobacte	Candidatus	OTU_1864	0.162	0.096	0.055	0.031	0.022
Bacteria	Actinobact	Conexibact	OTU_291	0.164	0.085	0.057	0.039	0.013
Bacteria	Unclassifie	Unclassifie	OTU_127	0.129	0.096	0.023	0.024	0.023
Bacteria	Proteobact	Pelomonas	OTU_239	0.145	0.051	0.044	0.039	0.001
Bacteria	Acidobacte	Gp6	OTU_110	0.135	0.075	0.035	0.043	0.010
Bacteria	Actinobact	Aquihabita	OTU_299	0.133	0.078	0.036	0.022	0.012
Bacteria	Verrucomi	Opitutus	OTU_208	0.160	0.072	0.063	0.044	0.011

Bacteria	Acidobacte	Gp4	OTU_7758	0.105	0.098	0.008	0.008	0.035
Bacteria	Actinobact	Unclassifie	OTU_147	0.207	0.080	0.111	0.059	0.025
Bacteria	Acidobacte	Gp6	OTU_2748	0.102	0.101	0.009	0.009	0.044
Bacteria	Gemmatim	Gemmatim	OTU_198	0.145	0.078	0.053	0.043	0.020
Bacteria	Actinobact	Nocardioid	OTU_69	0.175	0.087	0.083	0.069	0.047
Bacteria	candidate d	WPS-1_ge	OTU_464	0.097	0.085	0.007	0.009	0.026
Bacteria	Proteobact	Pseudodug	OTU_178	0.124	0.045	0.034	0.032	0.001
Bacteria	Proteobact	Unclassifie	OTU_618	0.107	0.048	0.017	0.016	0.001
Bacteria	Actinobact	Gaiella	OTU_896	0.096	0.091	0.009	0.010	0.039
Bacteria	Proteobact	Unclassifie	OTU_469	0.107	0.090	0.020	0.025	0.040
Bacteria	Bacteroidet	Terrimonas	OTU_544	0.110	0.052	0.024	0.020	0.003
Bacteria	Unclassifie	Unclassifie	OTU_150	0.138	0.080	0.053	0.037	0.029
Bacteria	Acidobacte	Gp4	OTU_750	0.093	0.060	0.008	0.010	0.007
Bacteria	Verrucomi	Spartobact	OTU_522	0.131	0.050	0.051	0.036	0.005
Bacteria	Actinobact	Solirubrob	OTU_497	0.098	0.082	0.018	0.020	0.037
Bacteria	Actinobact	Aciditerrim	OTU_638	0.087	0.073	0.007	0.006	0.023
Bacteria	Actinobact	Conexibact	OTU_211	0.123	0.041	0.044	0.038	0.002
Bacteria	Acidobacte	Gp6	OTU_6281	0.106	0.061	0.029	0.035	0.014
Bacteria	Actinobact	Solirubrob	OTU_657	0.077	0.077	0.000	0.000	0.033
Bacteria	Acidobacte	Gp4	OTU_8714	0.081	0.060	0.005	0.005	0.012
Bacteria	Proteobact	Unclassifie	OTU_589	0.097	0.054	0.022	0.014	0.007
Bacteria	Proteobact	Variovorax	OTU_2056	0.151	0.074	0.076	0.044	0.041
Bacteria	Verrucomi	Unclassifie	OTU_304	0.080	0.052	0.006	0.005	0.007
Bacteria	Acidobacte	Gp6	OTU_3585	0.091	0.051	0.018	0.017	0.006
Bacteria	Actinobact	Blastococc	OTU_115	0.133	0.078	0.060	0.033	0.047
Bacteria	Proteobact	Unclassifie	OTU_1479	0.177	0.048	0.104	0.046	0.012
Bacteria	Unclassifie	Unclassifie	OTU_1942	0.075	0.064	0.003	0.004	0.021
Bacteria	Proteobact	Unclassifie	OTU_513	0.072	0.048	0.003	0.003	0.006
Bacteria	Unclassifie	Unclassifie	OTU_506	0.076	0.056	0.007	0.008	0.014
Bacteria	Actinobact	Unclassifie	OTU_7451	0.092	0.056	0.023	0.013	0.014
Bacteria	Verrucomi	Subdivisio	OTU_624	0.117	0.046	0.049	0.048	0.017
Bacteria	Bacteroidet	Flavisoliba	OTU_120	0.145	0.058	0.078	0.033	0.022
Bacteria	Acidobacte	Gp6	OTU_6956	0.079	0.047	0.012	0.010	0.007
Bacteria	Proteobact	Unclassifie	OTU_334	0.076	0.071	0.009	0.009	0.043
Bacteria	Proteobact	Unclassifie	OTU_242	0.126	0.033	0.059	0.028	0.001
Bacteria	Verrucomi	Opitutus	OTU_645	0.087	0.054	0.020	0.019	0.014
Bacteria	Acidobacte	Aridibacter	OTU_407	0.107	0.065	0.042	0.033	0.039
Bacteria	Acidobacte	Unclassifie	OTU_2086	0.076	0.054	0.011	0.013	0.015
Bacteria	Actinobact	Gaiella	OTU_1345	0.073	0.048	0.008	0.007	0.009
Bacteria	Acidobacte	Gp6	OTU_1906	0.077	0.022	0.014	0.010	0.000
Bacteria	Acidobacte	Gp6	OTU_270	0.066	0.065	0.004	0.004	0.037
Bacteria	Proteobact	Massilia	OTU_216	0.125	0.065	0.063	0.033	0.047
Bacteria	Actinobact	Unclassifie	OTU_2033	0.122	0.055	0.063	0.031	0.031
Bacteria	Proteobact	Haliangium	OTU_3989	0.077	0.025	0.018	0.019	0.000
Bacteria	Bacteroidet	Parafilimor	OTU_889	0.064	0.058	0.006	0.010	0.033
Bacteria	Proteobact	Povalibact	OTU_697	0.072	0.064	0.014	0.012	0.049
Bacteria	Acidobacte	Gp6	OTU_4904	0.063	0.053	0.005	0.005	0.023
Bacteria	Verrucomi	Spartobact	OTU_1311	0.087	0.046	0.030	0.027	0.017
Bacteria	Actinobact	Gaiella	OTU_8708	0.062	0.044	0.005	0.007	0.011
Bacteria	Acidobacte	Gp6	OTU_6070	0.066	0.054	0.010	0.018	0.030
Bacteria	Gemmatim	Gemmatim	OTU_260	0.066	0.061	0.010	0.011	0.045
Bacteria	Acidobacte	Gp4	OTU_2388	0.066	0.055	0.010	0.008	0.031
Bacteria	Unclassifie	Unclassifie	OTU_658	0.058	0.037	0.003	0.003	0.006
Bacteria	Candidatus	Sacchariba	OTU_882	0.054	0.052	0.000	0.000	0.027
Bacteria	Actinobact	Unclassifie	OTU_408	0.085	0.051	0.031	0.028	0.032
Bacteria	Acidobacte	Gp16	OTU_496	0.099	0.032	0.046	0.037	0.012

Bacteria	Acidobacte	Gp6	OTU_159	0.087	0.041	0.033	0.028	0.014
Bacteria	Acidobacte	Gp4	OTU_8027	0.061	0.044	0.008	0.010	0.015
Bacteria	Proteobacte	Unclassifie	OTU_553	0.058	0.024	0.005	0.004	0.001
Bacteria	Actinobact	Gaiella	OTU_382	0.069	0.054	0.016	0.019	0.040
Bacteria	Gemmatim	Gemmatim	OTU_293	0.088	0.027	0.037	0.029	0.004
Bacteria	Acidobacte	Gp7	OTU_2331	0.056	0.039	0.006	0.008	0.011
Bacteria	Proteobacte	Unclassifie	OTU_880	0.054	0.052	0.004	0.005	0.040
Bacteria	Actinobact	Terrabacter	OTU_7197	0.090	0.047	0.040	0.020	0.028
Bacteria	Proteobacte	Reyranella	OTU_595	0.060	0.041	0.012	0.007	0.016
Bacteria	Proteobacte	Unclassifie	OTU_463	0.079	0.034	0.031	0.022	0.008
Bacteria	Actinobact	Gaiella	OTU_335	0.073	0.031	0.025	0.016	0.005
Bacteria	Actinobact	Gaiella	OTU_366	0.060	0.021	0.013	0.012	0.000
Bacteria	Acidobacte	Gp4	OTU_1101	0.057	0.029	0.010	0.010	0.003
Bacteria	Actinobact	Gaiella	OTU_2538	0.051	0.048	0.004	0.008	0.036
Bacteria	Actinobact	Ilumatobac	OTU_501	0.054	0.047	0.008	0.008	0.034
Bacteria	Actinobact	Nocardioid	OTU_2656	0.063	0.040	0.016	0.009	0.019
Bacteria	Actinobact	Conexibact	OTU_353	0.068	0.033	0.022	0.018	0.008
Bacteria	Actinobact	Gaiella	OTU_702	0.071	0.034	0.025	0.018	0.009
Bacteria	candidate d	WPS-1_ge	OTU_646	0.047	0.041	0.002	0.002	0.023
Bacteria	Unclassifie	Unclassifie	OTU_7265	0.052	0.047	0.008	0.009	0.043
Bacteria	Proteobacte	Unclassifie	OTU_576	0.062	0.032	0.018	0.013	0.007
Bacteria	Acidobacte	Gp6	OTU_7014	0.062	0.036	0.018	0.021	0.018
Bacteria	Proteobacte	Unclassifie	OTU_527	0.054	0.045	0.010	0.010	0.038
Bacteria	Proteobacte	Unclassifie	OTU_349	0.084	0.040	0.040	0.016	0.024
Bacteria	Unclassifie	Unclassifie	OTU_956	0.047	0.042	0.004	0.005	0.029
Bacteria	Verrucomi	Spartobacte	OTU_218	0.109	0.031	0.066	0.033	0.026
Bacteria	Proteobacte	Unclassifie	OTU_1616	0.049	0.036	0.006	0.006	0.016
Bacteria	Proteobacte	Unclassifie	OTU_193	0.077	0.040	0.034	0.028	0.039
Bacteria	candidate d	WPS-1_ge	OTU_816	0.061	0.035	0.019	0.013	0.014
Bacteria	Actinobact	Conexibact	OTU_500	0.047	0.033	0.004	0.006	0.011
Bacteria	Unclassifie	Unclassifie	OTU_590	0.046	0.044	0.003	0.005	0.039
Bacteria	Latescibact	Latescibact	OTU_5436	0.046	0.033	0.004	0.004	0.012
Bacteria	Acidobacte	Aridibacter	OTU_696	0.044	0.031	0.003	0.004	0.009
Bacteria	Verrucomi	Spartobacte	OTU_614	0.042	0.039	0.001	0.001	0.026
Bacteria	Unclassifie	Unclassifie	OTU_403	0.045	0.033	0.004	0.005	0.013
Bacteria	Acidobacte	Gp3	OTU_1183	0.051	0.026	0.010	0.011	0.004
Bacteria	candidate d	WPS-1_ge	OTU_479	0.059	0.034	0.018	0.015	0.015
Bacteria	Actinobact	Aquihabita	OTU_1418	0.057	0.031	0.017	0.022	0.014
Bacteria	Unclassifie	Unclassifie	OTU_4079	0.046	0.037	0.006	0.005	0.023
Bacteria	Unclassifie	Unclassifie	OTU_449	0.046	0.031	0.006	0.008	0.011
Bacteria	Chloroflexi	Unclassifie	OTU_1107	0.059	0.034	0.019	0.019	0.019
Bacteria	Armatimon	Armatimon	OTU_258	0.063	0.025	0.023	0.028	0.014
Bacteria	Proteobacte	Hyphomicr	OTU_276	0.085	0.029	0.045	0.037	0.041
Bacteria	Proteobacte	Unclassifie	OTU_1096	0.049	0.026	0.009	0.013	0.005
Bacteria	Actinobact	Unclassifie	OTU_1717	0.047	0.033	0.008	0.006	0.016
Bacteria	Acidobacte	Gp6	OTU_345	0.049	0.030	0.010	0.007	0.011
Bacteria	Proteobacte	Unclassifie	OTU_822	0.044	0.038	0.005	0.005	0.030
Bacteria	Proteobacte	Unclassifie	OTU_1235	0.046	0.035	0.007	0.010	0.022
Bacteria	Proteobacte	Unclassifie	OTU_1119	0.045	0.042	0.006	0.011	0.044
Bacteria	Proteobacte	Lysobacter	OTU_814	0.043	0.034	0.004	0.004	0.019
Bacteria	Actinobact	Actinophyt	OTU_4412	0.045	0.035	0.006	0.007	0.023
Bacteria	Actinobact	Unclassifie	OTU_441	0.089	0.022	0.051	0.021	0.005
Bacteria	Unclassifie	Unclassifie	OTU_1346	0.051	0.031	0.013	0.011	0.015
Bacteria	Proteobacte	Unclassifie	OTU_414	0.051	0.027	0.013	0.013	0.007
Bacteria	Proteobacte	Skermanell	OTU_352	0.058	0.033	0.021	0.015	0.024
Bacteria	Actinobact	Aquihabita	OTU_1180	0.040	0.022	0.004	0.006	0.003

Bacteria	Armatimon	Armatimon	OTU_582	0.041	0.022	0.004	0.009	0.003
Bacteria	candidate d	WPS-1_gei	OTU_716	0.042	0.028	0.006	0.012	0.012
Bacteria	candidate d	WPS-1_gei	OTU_591	0.049	0.023	0.013	0.008	0.004
Bacteria	Actinobact	Unclassifie	OTU_3055	0.048	0.025	0.012	0.011	0.007
Bacteria	Actinobact	Actinomad	OTU_712	0.062	0.028	0.027	0.023	0.023
Bacteria	Actinobact	Nocardioid	OTU_3526	0.041	0.035	0.006	0.008	0.031
Bacteria	Acidobacte	Gp6	OTU_6273	0.046	0.028	0.010	0.011	0.013
Bacteria	Actinobact	Unclassifie	OTU_391	0.043	0.015	0.007	0.006	0.000
Bacteria	Unclassifie	Unclassifie	OTU_1026	0.037	0.032	0.002	0.004	0.023
Bacteria	Verrucomi	Unclassifie	OTU_800	0.045	0.036	0.011	0.012	0.042
Bacteria	Proteobact	Unclassifie	OTU_921	0.034	0.021	0.000	0.000	0.004
Bacteria	Acidobacte	Gp6	OTU_3140	0.037	0.027	0.004	0.006	0.014
Bacteria	Unclassifie	Unclassifie	OTU_1623	0.034	0.021	0.001	0.002	0.004
Bacteria	Proteobact	Unclassifie	OTU_9399	0.048	0.031	0.015	0.011	0.028
Bacteria	Actinobact	Conexibact	OTU_358	0.054	0.018	0.021	0.006	0.001
Bacteria	Verrucomi	Subdivisio	OTU_493	0.050	0.025	0.018	0.025	0.028
Bacteria	Proteobact	Cupriavidu	OTU_320	0.040	0.024	0.008	0.011	0.008
Bacteria	Acidobacte	Gp3	OTU_7700	0.034	0.021	0.002	0.003	0.004
Bacteria	Acidobacte	Gp3	OTU_1020	0.035	0.030	0.003	0.005	0.027
Bacteria	Planctomyc	Zavarzinell	OTU_718	0.043	0.034	0.012	0.015	0.047
Bacteria	Proteobact	Unclassifie	OTU_280	0.036	0.020	0.004	0.004	0.004
Bacteria	Actinobact	Unclassifie	OTU_8659	0.037	0.030	0.005	0.007	0.027
Bacteria	Bacteroide	Unclassifie	OTU_1185	0.035	0.032	0.003	0.005	0.035
Bacteria	candidate d	WPS-1_gei	OTU_776	0.034	0.031	0.002	0.003	0.032
Bacteria	Gemmatim	Gemmatim	OTU_354	0.037	0.013	0.006	0.008	0.000
Bacteria	candidate d	WPS-1_gei	OTU_667	0.049	0.027	0.019	0.014	0.024
Bacteria	Proteobact	Bosea	OTU_383	0.043	0.021	0.013	0.011	0.006
Bacteria	Bacteroide	Unclassifie	OTU_647	0.032	0.032	0.002	0.002	0.044
Bacteria	Acidobacte	Gp4	OTU_2665	0.040	0.030	0.010	0.010	0.034
Bacteria	Proteobact	Unclassifie	OTU_878	0.038	0.017	0.008	0.006	0.002
Bacteria	Unclassifie	Unclassifie	OTU_8085	0.032	0.027	0.002	0.003	0.022
Bacteria	Acidobacte	Unclassifie	OTU_271	0.052	0.017	0.022	0.013	0.003
Bacteria	Unclassifie	Unclassifie	OTU_703	0.039	0.022	0.010	0.006	0.009
Bacteria	Acidobacte	Gp17	OTU_2055	0.032	0.024	0.002	0.004	0.014
Bacteria	Gemmatim	Gemmatim	OTU_1404	0.035	0.026	0.006	0.008	0.023
Bacteria	Proteobact	Pedomicrol	OTU_1126	0.042	0.025	0.014	0.017	0.028
Bacteria	Acidobacte	Gp6	OTU_674	0.032	0.027	0.003	0.004	0.026
Bacteria	Acidobacte	Gp6	OTU_7800	0.029	0.020	0.001	0.002	0.007
Bacteria	Armatimon	Armatimon	OTU_1589	0.030	0.017	0.002	0.003	0.003
Bacteria	Chloroflexi	Unclassifie	OTU_423	0.034	0.029	0.005	0.005	0.036
Bacteria	Actinobact	Aciditerrim	OTU_356	0.037	0.018	0.009	0.004	0.005
Bacteria	Actinobact	Gaiella	OTU_1998	0.038	0.025	0.010	0.005	0.021
Bacteria	Proteobact	Unclassifie	OTU_900	0.031	0.025	0.003	0.004	0.023
Bacteria	Proteobact	Unclassifie	OTU_5265	0.037	0.021	0.010	0.012	0.012
Bacteria	Bacteroide	Mucilaginil	OTU_1401	0.050	0.023	0.023	0.019	0.032
Bacteria	candidate d	WPS-1_gei	OTU_1919	0.029	0.025	0.003	0.003	0.025
Bacteria	Chloroflexi	Unclassifie	OTU_1171	0.029	0.022	0.002	0.003	0.014
Bacteria	Planctomyc	Pirellula	OTU_1057	0.041	0.018	0.015	0.017	0.014
Bacteria	Planctomyc	Pirellula	OTU_1711	0.036	0.012	0.010	0.012	0.001
Bacteria	Bacteroide	Unclassifie	OTU_1718	0.028	0.023	0.002	0.003	0.019
Bacteria	candidate d	WPS-1_gei	OTU_1535	0.028	0.018	0.002	0.004	0.007
Bacteria	Gemmatim	Gemmatim	OTU_1104	0.031	0.018	0.005	0.006	0.006
Bacteria	Proteobact	Sphingomo	OTU_1414	0.037	0.017	0.012	0.016	0.012
Bacteria	Actinobact	Conexibact	OTU_4156	0.034	0.021	0.008	0.009	0.014
Bacteria	Chloroflexi	Unclassifie	OTU_682	0.028	0.015	0.003	0.004	0.002
Bacteria	Acidobacte	Gp4	OTU_1424	0.026	0.019	0.001	0.001	0.011

Bacteria	Bacteroidet	Ferruginiba	OTU_945	0.025	0.026	0.000	0.000	0.036
Bacteria	Actinobact	Aciditerrim	OTU_739	0.039	0.015	0.014	0.005	0.002
Bacteria	Actinobact	Thermoleo	OTU_1112	0.031	0.014	0.007	0.007	0.002
Bacteria	Acidobacte	Gp6	OTU_4035	0.031	0.022	0.006	0.005	0.020
Bacteria	Actinobact	Gaiella	OTU_935	0.037	0.012	0.013	0.011	0.001
Bacteria	Chloroflexi	Unclassifie	OTU_568	0.032	0.021	0.008	0.010	0.021
Bacteria	Proteobacte	Nitrosospir	OTU_4299	0.027	0.016	0.003	0.005	0.004
Bacteria	Proteobacte	Unclassifie	OTU_1051	0.026	0.011	0.002	0.004	0.000
Bacteria	Unclassifie	Unclassifie	OTU_1391	0.028	0.015	0.004	0.007	0.004
Bacteria	Proteobacte	Unclassifie	OTU_642	0.027	0.018	0.003	0.005	0.008
Bacteria	Acidobacte	Candidatus	OTU_5632	0.028	0.024	0.004	0.005	0.031
Bacteria	Actinobact	Aquihabita	OTU_684	0.026	0.021	0.002	0.003	0.021
Bacteria	Proteobacte	Unclassifie	OTU_821	0.025	0.023	0.002	0.002	0.029
Bacteria	Proteobacte	Unclassifie	OTU_1304	0.028	0.020	0.004	0.006	0.015
Bacteria	Actinobact	Unclassifie	OTU_518	0.032	0.016	0.009	0.008	0.007
Bacteria	candidate d	WPS-1_ge	OTU_8125	0.026	0.020	0.003	0.006	0.018
Bacteria	Unclassifie	Unclassifie	OTU_1719	0.023	0.022	0.000	0.000	0.031
Bacteria	Unclassifie	Unclassifie	OTU_2969	0.024	0.019	0.002	0.003	0.017
Bacteria	Unclassifie	Unclassifie	OTU_751	0.029	0.020	0.007	0.009	0.020
Bacteria	Verrucomi	Spartobacte	OTU_976	0.026	0.022	0.004	0.006	0.032
Bacteria	Acidobacte	Gp6	OTU_2420	0.029	0.006	0.007	0.005	0.000
Bacteria	Verrucomi	Subdivisio	OTU_1194	0.022	0.017	0.000	0.000	0.010
Bacteria	Acidobacte	Gp3	OTU_1017	0.027	0.024	0.005	0.008	0.049
Bacteria	Actinobact	Gaiella	OTU_726	0.031	0.018	0.009	0.006	0.015
Bacteria	Bacteroidet	Unclassifie	OTU_957	0.029	0.012	0.007	0.009	0.002
Bacteria	Actinobact	Conexibact	OTU_560	0.030	0.016	0.008	0.008	0.009
Bacteria	Acidobacte	Gp4	OTU_842	0.030	0.014	0.008	0.008	0.004
Bacteria	Actinobact	Gaiella	OTU_1070	0.028	0.015	0.007	0.007	0.007
Bacteria	Acidobacte	Gp7	OTU_904	0.023	0.022	0.001	0.002	0.034
Bacteria	Proteobacte	Povalibacte	OTU_1074	0.023	0.017	0.002	0.002	0.011
Bacteria	Proteobacte	Unclassifie	OTU_580	0.031	0.020	0.010	0.014	0.040
Bacteria	Bacteroidet	Niastella	OTU_1837	0.023	0.015	0.002	0.003	0.007
Bacteria	Acidobacte	Gp4	OTU_770	0.022	0.020	0.001	0.003	0.028
Bacteria	Actinobact	Mycobacte	OTU_631	0.035	0.018	0.014	0.007	0.016
Bacteria	Bacteroidet	Unclassifie	OTU_8205	0.021	0.017	0.001	0.002	0.014
Bacteria	Actinobact	Nocardioid	OTU_2286	0.037	0.016	0.017	0.012	0.017
Bacteria	candidate d	WPS-1_ge	OTU_797	0.026	0.018	0.006	0.011	0.028
Bacteria	Latescibact	Latescibact	OTU_898	0.023	0.018	0.003	0.006	0.024
Bacteria	Armatimon	Armatimon	OTU_870	0.022	0.017	0.003	0.003	0.018
Bacteria	Proteobacte	Unclassifie	OTU_6685	0.024	0.017	0.005	0.008	0.024
Bacteria	Acidobacte	Gp6	OTU_466	0.027	0.018	0.008	0.007	0.031
Bacteria	Proteobacte	Unclassifie	OTU_1827	0.022	0.012	0.003	0.003	0.005
Bacteria	Unclassifie	Unclassifie	OTU_2649	0.022	0.021	0.003	0.004	0.048
Bacteria	Planctomyc	Gemmata	OTU_2315	0.022	0.015	0.003	0.003	0.012
Bacteria	Acidobacte	Gp7	OTU_1601	0.021	0.017	0.002	0.004	0.021
Bacteria	Unclassifie	Unclassifie	OTU_3201	0.020	0.017	0.001	0.003	0.022
Bacteria	candidate d	WPS-1_ge	OTU_825	0.029	0.018	0.010	0.007	0.031
Bacteria	Proteobacte	Unclassifie	OTU_1270	0.020	0.015	0.002	0.003	0.014
Bacteria	Actinobact	Thermoleo	OTU_5310	0.022	0.018	0.004	0.006	0.031
Bacteria	Proteobacte	Unclassifie	OTU_2100	0.022	0.015	0.004	0.005	0.014
Bacteria	Bacteroidet	Unclassifie	OTU_890	0.018	0.015	0.000	0.000	0.016
Bacteria	Actinobact	Unclassifie	OTU_845	0.023	0.014	0.004	0.005	0.011
Bacteria	Acidobacte	Gp3	OTU_3754	0.023	0.016	0.005	0.008	0.024
Bacteria	Proteobacte	Panacagrim	OTU_573	0.027	0.014	0.009	0.010	0.016
Bacteria	Gemmatim	Gemmatim	OTU_412	0.022	0.012	0.005	0.007	0.005
Bacteria	Acidobacte	Gp6	OTU_4250	0.020	0.016	0.002	0.002	0.025

Bacteria	Actinobact	Gaiella	OTU_7346	0.018	0.019	0.000	0.000	0.040
Bacteria	Unclassifie	Unclassifie	OTU_1369	0.023	0.019	0.006	0.007	0.050
Bacteria	Proteobact	Labilithrix	OTU_604	0.025	0.017	0.007	0.006	0.028
Bacteria	Unclassifie	Unclassifie	OTU_1603	0.017	0.018	0.000	0.000	0.036
Bacteria	Unclassifie	Unclassifie	OTU_7289	0.025	0.016	0.007	0.010	0.037
Bacteria	Proteobact	Labilithrix	OTU_1193	0.020	0.009	0.003	0.003	0.001
Bacteria	Actinobact	Conexibact	OTU_4812	0.025	0.015	0.008	0.009	0.026
Bacteria	candidate d	WPS-1_gen	OTU_920	0.018	0.009	0.002	0.002	0.001
Bacteria	Armatimon	Armatimon	OTU_1123	0.029	0.014	0.012	0.007	0.018
Bacteria	Acidobacte	Gp6	OTU_7880	0.019	0.014	0.002	0.003	0.017
Bacteria	Proteobact	Duganella	OTU_2112	0.020	0.016	0.004	0.004	0.028
Bacteria	Proteobact	Inquilinus	OTU_1480	0.019	0.017	0.002	0.003	0.041
Bacteria	Actinobact	Unclassifie	OTU_1599	0.018	0.013	0.001	0.002	0.015
Bacteria	Proteobact	Unclassifie	OTU_607	0.018	0.016	0.002	0.003	0.032
Bacteria	Proteobact	Unclassifie	OTU_9435	0.031	0.015	0.015	0.011	0.040
Bacteria	Armatimon	Armatimon	OTU_788	0.020	0.013	0.004	0.006	0.014
Bacteria	Actinobact	Unclassifie	OTU_1105	0.027	0.012	0.011	0.010	0.014
Bacteria	Proteobact	Unclassifie	OTU_2431	0.016	0.014	0.001	0.001	0.019
Bacteria	Proteobact	Methyloph	OTU_511	0.020	0.015	0.004	0.006	0.031
Bacteria	Proteobact	Reyranella	OTU_2459	0.020	0.015	0.005	0.002	0.028
Bacteria	Acidobacte	Gp25	OTU_1538	0.015	0.016	0.000	0.000	0.045
Bacteria	Proteobact	Bauldia	OTU_1063	0.020	0.012	0.005	0.006	0.012
Bacteria	Proteobact	Caulobacte	OTU_562	0.030	0.008	0.015	0.010	0.011
Bacteria	Planctomy	Pirellula	OTU_2971	0.015	0.011	0.000	0.000	0.010
Bacteria	Acidobacte	Gp6	OTU_528	0.021	0.013	0.007	0.006	0.022
Bacteria	Actinobact	Sporichthy	OTU_333	0.030	0.012	0.015	0.007	0.019
Bacteria	Proteobact	Unclassifie	OTU_2387	0.017	0.007	0.003	0.003	0.000
Bacteria	Unclassifie	Unclassifie	OTU_874	0.015	0.012	0.000	0.000	0.017
Bacteria	Planctomy	Pirellula	OTU_1492	0.017	0.010	0.003	0.004	0.007
Bacteria	candidate d	WPS-1_gen	OTU_1630	0.016	0.013	0.002	0.002	0.027
Bacteria	Unclassifie	Unclassifie	OTU_374	0.016	0.015	0.002	0.003	0.048
Bacteria	Actinobact	Unclassifie	OTU_4640	0.018	0.012	0.004	0.004	0.021
Bacteria	Verrucomi	Subdivision	OTU_2272	0.015	0.009	0.001	0.002	0.005
Bacteria	Unclassifie	Unclassifie	OTU_962	0.017	0.012	0.004	0.004	0.020
Bacteria	Actinobact	Saccharoth	OTU_2471	0.016	0.007	0.003	0.004	0.001
Bacteria	Unclassifie	Unclassifie	OTU_1230	0.015	0.008	0.002	0.003	0.003
Bacteria	Unclassifie	Unclassifie	OTU_813	0.018	0.014	0.005	0.003	0.038
Bacteria	Unclassifie	Unclassifie	OTU_1281	0.015	0.014	0.002	0.003	0.039
Bacteria	Unclassifie	Unclassifie	OTU_2794	0.014	0.010	0.001	0.002	0.009
Bacteria	Verrucomi	Subdivision	OTU_3356	0.013	0.011	0.000	0.000	0.017
Bacteria	Actinobact	Nocardioid	OTU_4115	0.017	0.009	0.004	0.005	0.006
Bacteria	Proteobact	Arenimona	OTU_8763	0.014	0.011	0.001	0.002	0.017
Bacteria	Unclassifie	Unclassifie	OTU_3622	0.013	0.013	0.000	0.000	0.029
Bacteria	Gemmatim	Gemmatim	OTU_486	0.014	0.011	0.001	0.002	0.014
Bacteria	Bacteroide	Unclassifie	OTU_1067	0.015	0.014	0.002	0.002	0.046
Bacteria	Verrucomi	Terrimicrol	OTU_2332	0.015	0.012	0.002	0.002	0.022
Bacteria	Proteobact	Unclassifie	OTU_1465	0.018	0.009	0.005	0.006	0.009
Bacteria	candidate d	WPS-1_gen	OTU_1377	0.015	0.012	0.002	0.003	0.025
Bacteria	Acidobacte	Gp6	OTU_6984	0.013	0.012	0.000	0.000	0.029
Bacteria	Proteobact	Unclassifie	OTU_8769	0.015	0.013	0.002	0.003	0.040
Bacteria	Verrucomi	Spartobact	OTU_6076	0.013	0.011	0.001	0.002	0.017
Bacteria	candidate d	WPS-1_gen	OTU_2155	0.017	0.009	0.004	0.004	0.006
Bacteria	Proteobact	Unclassifie	OTU_1357	0.019	0.010	0.006	0.005	0.016
Bacteria	Unclassifie	Unclassifie	OTU_1362	0.013	0.013	0.000	0.000	0.035
Bacteria	Planctomy	Unclassifie	OTU_1467	0.014	0.011	0.001	0.003	0.019
Bacteria	Acidobacte	Gp5	OTU_809	0.028	0.008	0.016	0.010	0.027

Bacteria	Proteobacte	Unclassifie	OTU_1326	0.014	0.013	0.002	0.002	0.041
Bacteria	Verrucomic	Subdivision	OTU_1517	0.012	0.013	0.000	0.000	0.038
Bacteria	Acidobacte	Gp3	OTU_1798	0.012	0.008	0.000	0.000	0.006
Bacteria	Chloroflexi	Unclassifie	OTU_2778	0.014	0.011	0.002	0.003	0.025
Bacteria	Unclassifie	Unclassifie	OTU_1863	0.012	0.013	0.000	0.000	0.046
Bacteria	Latescibact	Latescibact	OTU_3355	0.012	0.013	0.000	0.000	0.046
Bacteria	Actinobact	Aquihabita	OTU_1283	0.016	0.012	0.004	0.006	0.038
Bacteria	Actinobact	Thermoleo	OTU_2841	0.014	0.008	0.002	0.003	0.006
Bacteria	Planctomyc	Tepidispha	OTU_4314	0.013	0.012	0.001	0.002	0.039
Bacteria	Acidobacte	Unclassifie	OTU_1647	0.011	0.012	0.000	0.000	0.043
Bacteria	Planctomyc	Pirellula	OTU_4374	0.011	0.010	0.000	0.000	0.021
Bacteria	Planctomyc	Thermogut	OTU_2150	0.015	0.011	0.004	0.004	0.029
Bacteria	Armatimon	Armatimon	OTU_9474	0.015	0.012	0.003	0.003	0.045
Bacteria	Acidobacte	Bryobacter	OTU_1316	0.016	0.010	0.005	0.007	0.031
Bacteria	Proteobacte	Acidiphiliu	OTU_802	0.015	0.009	0.004	0.005	0.018
Bacteria	Chloroflexi	Unclassifie	OTU_698	0.018	0.010	0.006	0.009	0.048
Bacteria	Proteobacte	Unclassifie	OTU_1883	0.013	0.010	0.001	0.003	0.018
Bacteria	Proteobacte	Unclassifie	OTU_1721	0.013	0.008	0.002	0.002	0.007
Bacteria	Chloroflexi	Unclassifie	OTU_2769	0.011	0.009	0.000	0.000	0.011
Bacteria	Actinobact	Actinoplan	OTU_1936	0.012	0.012	0.001	0.002	0.039
Bacteria	Actinobact	Rhodococc	OTU_3768	0.014	0.010	0.003	0.005	0.029
Bacteria	Unclassifie	Unclassifie	OTU_1606	0.016	0.009	0.005	0.006	0.016
Bacteria	Bacteroidet	Unclassifie	OTU_810	0.011	0.012	0.000	0.000	0.039
Bacteria	Proteobacte	Unclassifie	OTU_3102	0.014	0.010	0.003	0.005	0.028
Bacteria	Proteobacte	Unclassifie	OTU_5005	0.013	0.012	0.002	0.003	0.043
Bacteria	Actinobact	Unclassifie	OTU_2744	0.014	0.007	0.003	0.005	0.006
Bacteria	Bacteroidet	Unclassifie	OTU_2603	0.011	0.010	0.000	0.000	0.030
Bacteria	Bacteroidet	Flavisoliba	OTU_6302	0.015	0.011	0.005	0.005	0.040
Bacteria	Planctomyc	Unclassifie	OTU_2541	0.012	0.010	0.001	0.003	0.022
Bacteria	Chloroflexi	Unclassifie	OTU_2208	0.011	0.009	0.000	0.000	0.021
Bacteria	Proteobacte	Unclassifie	OTU_830	0.014	0.006	0.003	0.004	0.003
Bacteria	Chloroflexi	Unclassifie	OTU_1560	0.011	0.009	0.000	0.000	0.014
Bacteria	Acidobacte	Gp16	OTU_2250	0.010	0.011	0.000	0.000	0.035
Bacteria	Chloroflexi	Unclassifie	OTU_1913	0.010	0.009	0.000	0.000	0.020
Bacteria	Latescibact	Latescibact	OTU_2755	0.010	0.010	0.000	0.000	0.029
Bacteria	Proteobacte	Bdellovibri	OTU_1115	0.013	0.011	0.002	0.003	0.042
Bacteria	Planctomyc	Singulispha	OTU_9305	0.013	0.010	0.003	0.003	0.033
Bacteria	Armatimon	Chthonomo	OTU_6933	0.016	0.004	0.006	0.009	0.022
Bacteria	Proteobacte	Unclassifie	OTU_3039	0.010	0.007	0.000	0.000	0.007
Bacteria	Acidobacte	Gp25	OTU_256	0.010	0.011	0.000	0.000	0.046
Bacteria	candidate d	WPS-1_ge	OTU_2446	0.010	0.008	0.000	0.000	0.012
Bacteria	Planctomyc	Zavarzinell	OTU_2294	0.013	0.008	0.003	0.003	0.015
Bacteria	Bacteroidet	Flavobacte	OTU_3711	0.010	0.010	0.000	0.000	0.040
Bacteria	Unclassifie	Unclassifie	OTU_1292	0.018	0.009	0.008	0.006	0.041
Bacteria	Actinobact	Nocardioid	OTU_2483	0.013	0.008	0.003	0.004	0.017
Bacteria	Gemmatim	Gemmatim	OTU_2325	0.010	0.010	0.000	0.000	0.042
Bacteria	Acidobacte	Gp6	OTU_355	0.010	0.008	0.000	0.000	0.017
Bacteria	Proteobacte	Unclassifie	OTU_7271	0.012	0.010	0.002	0.004	0.048
Bacteria	Unclassifie	Unclassifie	OTU_536	0.014	0.009	0.004	0.004	0.035
Bacteria	Verrucomic	Spartobact	OTU_4809	0.010	0.010	0.000	0.000	0.033
Bacteria	Unclassifie	Unclassifie	OTU_5626	0.011	0.010	0.002	0.003	0.037
Bacteria	Armatimon	Chthonomo	OTU_1917	0.010	0.011	0.000	0.000	0.048
Bacteria	Proteobacte	Unclassifie	OTU_3194	0.012	0.007	0.002	0.003	0.007
Bacteria	Planctomyc	Pirellula	OTU_6664	0.009	0.006	0.000	0.000	0.004
Bacteria	Acidobacte	Gp6	OTU_3297	0.009	0.007	0.000	0.000	0.008
Bacteria	Actinobact	Conexibact	OTU_612	0.009	0.008	0.000	0.000	0.021

Bacteria	Gemmatim	Gemmatim	OTU_1241	0.011	0.008	0.002	0.002	0.019
Bacteria	Firmicutes	Unclassifie	OTU_2845	0.012	0.007	0.003	0.003	0.009
Bacteria	Actinobact	Unclassifie	OTU_3931	0.012	0.010	0.003	0.004	0.047
Bacteria	Acidobacte	Gp6	OTU_5139	0.009	0.010	0.000	0.000	0.047
Bacteria	candidate d	WPS-1_ge	OTU_7250	0.011	0.010	0.002	0.003	0.042
Bacteria	Proteobacte	Unclassifie	OTU_2147	0.012	0.010	0.003	0.003	0.047
Bacteria	Planctomyc	Blastopirel	OTU_1534	0.009	0.007	0.000	0.000	0.011
Bacteria	Latescibact	Latescibact	OTU_2380	0.010	0.010	0.001	0.001	0.041
Bacteria	Planctomyc	Unclassifie	OTU_7512	0.010	0.009	0.001	0.003	0.031
Bacteria	Bacteroidet	Terrimonas	OTU_4309	0.009	0.006	0.000	0.000	0.004
Bacteria	Proteobacte	Unclassifie	OTU_2151	0.009	0.009	0.000	0.000	0.038
Bacteria	Bacteroidet	Ferruginiba	OTU_6886	0.010	0.007	0.001	0.001	0.013
Bacteria	Proteobacte	Caulobacte	OTU_2506	0.011	0.009	0.002	0.004	0.035
Bacteria	Acidobacte	Gp11	OTU_1537	0.009	0.008	0.000	0.000	0.027
Bacteria	Bacteroidet	Ferruginiba	OTU_1133	0.009	0.008	0.000	0.000	0.022
Bacteria	Acidobacte	Blastocatel	OTU_2445	0.011	0.008	0.002	0.004	0.026
Bacteria	Proteobacte	Unclassifie	OTU_1004	0.009	0.007	0.000	0.000	0.017
Bacteria	Bacteroidet	Chryseolin	OTU_2417	0.010	0.006	0.001	0.002	0.004
Bacteria	Actinobact	Conexibact	OTU_1829	0.009	0.009	0.000	0.000	0.032
Bacteria	Unclassifie	Unclassifie	OTU_4392	0.009	0.007	0.000	0.000	0.015
Bacteria	Actinobact	Conexibact	OTU_5450	0.009	0.007	0.000	0.000	0.015
Bacteria	Proteobacte	Enhygromy	OTU_1851	0.009	0.007	0.000	0.000	0.016
Bacteria	Bacteroidet	Unclassifie	OTU_1716	0.009	0.008	0.000	0.000	0.023
Bacteria	Proteobacte	Belnapia	OTU_2298	0.009	0.008	0.000	0.000	0.031
Bacteria	Candidatus	Saccharibac	OTU_5805	0.009	0.007	0.000	0.000	0.015
Bacteria	Actinobact	Aciditerrim	OTU_1500	0.009	0.007	0.000	0.000	0.011
Bacteria	Verrucomic	Spartobacte	OTU_7907	0.008	0.009	0.000	0.000	0.042
Bacteria	Acidobacte	Gp3	OTU_2047	0.008	0.009	0.000	0.000	0.050
Bacteria	Chloroflexi	Unclassifie	OTU_1621	0.011	0.007	0.002	0.004	0.017
Bacteria	Candidatus	Saccharibac	OTU_857	0.008	0.008	0.000	0.000	0.033
Bacteria	Actinobact	Unclassifie	OTU_7038	0.010	0.008	0.002	0.002	0.035
Bacteria	Actinobact	Gaiella	OTU_663	0.011	0.004	0.003	0.005	0.007
Bacteria	Unclassifie	Unclassifie	OTU_3064	0.008	0.005	0.000	0.000	0.003
Bacteria	Bacteroidet	Unclassifie	OTU_4163	0.009	0.008	0.001	0.002	0.026
Bacteria	Proteobacte	Unclassifie	OTU_1728	0.008	0.004	0.000	0.000	0.001
Bacteria	Bacteroidet	Chitinopha	OTU_2805	0.008	0.008	0.000	0.000	0.034
Bacteria	Unclassifie	Unclassifie	OTU_8947	0.008	0.008	0.000	0.000	0.036
Bacteria	Planctomyc	Pirellula	OTU_1713	0.008	0.008	0.000	0.000	0.042
Bacteria	Bacteroidet	Unclassifie	OTU_1498	0.008	0.005	0.000	0.000	0.006
Bacteria	Armatimon	Armatimon	OTU_2615	0.011	0.007	0.003	0.003	0.027
Bacteria	Acidobacte	Gp3	OTU_2792	0.008	0.006	0.000	0.000	0.013
Bacteria	Actinobact	Aciditerrim	OTU_2084	0.008	0.006	0.000	0.000	0.008
Bacteria	Proteobacte	Kofleria	OTU_2307	0.010	0.007	0.002	0.003	0.028
Bacteria	Proteobacte	Stella	OTU_9383	0.010	0.007	0.002	0.003	0.024
Bacteria	Unclassifie	Unclassifie	OTU_2003	0.008	0.006	0.000	0.000	0.012
Bacteria	Verrucomic	Subdivision	OTU_9375	0.008	0.008	0.001	0.001	0.034
Bacteria	Unclassifie	Unclassifie	OTU_4819	0.008	0.008	0.000	0.000	0.038
Bacteria	Acidobacte	Gp3	OTU_901	0.007	0.006	0.000	0.000	0.015
Bacteria	Proteobacte	Unclassifie	OTU_3392	0.007	0.005	0.000	0.000	0.007
Bacteria	Proteobacte	Rhodopseu	OTU_959	0.010	0.007	0.003	0.003	0.037
Bacteria	Proteobacte	Unclassifie	OTU_3786	0.007	0.006	0.000	0.000	0.018
Bacteria	Planctomyc	Singulispha	OTU_3706	0.007	0.006	0.000	0.000	0.014
Bacteria	Unclassifie	Unclassifie	OTU_3933	0.007	0.007	0.000	0.000	0.035
Bacteria	Acidobacte	Gp3	OTU_3338	0.007	0.005	0.000	0.000	0.009
Bacteria	candidate d	WPS-1_ge	OTU_9283	0.007	0.008	0.000	0.000	0.040
Bacteria	Unclassifie	Unclassifie	OTU_1615	0.007	0.004	0.000	0.000	0.003

Bacteria	Acidobacte	Blastocatel	OTU_1741	0.007	0.005	0.000	0.000	0.008
Bacteria	Bacteroidet	Terrimonas	OTU_4471	0.008	0.007	0.001	0.002	0.032
Bacteria	Proteobacte	Sphingomo	OTU_747	0.007	0.007	0.000	0.000	0.032
Bacteria	Planctomyc	Isosphaera	OTU_2246	0.007	0.005	0.000	0.000	0.009
Bacteria	candidate d	WPS-1_gen	OTU_7605	0.007	0.006	0.000	0.000	0.013
Bacteria	Acidobacte	Gp16	OTU_2911	0.007	0.006	0.000	0.000	0.013
Bacteria	Proteobacte	Unclassifie	OTU_7552	0.009	0.007	0.002	0.003	0.042
Bacteria	Unclassifie	Unclassifie	OTU_9118	0.007	0.007	0.000	0.000	0.037
Bacteria	Planctomyc	Unclassifie	OTU_2312	0.007	0.004	0.000	0.000	0.001
Bacteria	Planctomyc	Unclassifie	OTU_2244	0.009	0.006	0.003	0.003	0.018
Bacteria	Acidobacte	Gp6	OTU_2521	0.007	0.005	0.000	0.000	0.008
Bacteria	Proteobacte	Chondromy	OTU_3839	0.010	0.007	0.003	0.003	0.032
Bacteria	Proteobacte	Unclassifie	OTU_1201	0.007	0.005	0.000	0.000	0.009
Bacteria	Unclassifie	Unclassifie	OTU_2062	0.009	0.006	0.002	0.002	0.021
Bacteria	Acidobacte	Gp6	OTU_7338	0.008	0.006	0.001	0.003	0.022
Bacteria	candidate d	WPS-1_gen	OTU_1429	0.008	0.007	0.002	0.002	0.046
Bacteria	Acidobacte	Gp10	OTU_1677	0.009	0.006	0.003	0.004	0.030
Bacteria	Actinobacte	Iamia	OTU_4097	0.007	0.006	0.000	0.000	0.021
Bacteria	Proteobacte	Unclassifie	OTU_793	0.008	0.006	0.002	0.003	0.031
Bacteria	Verrucomic	Subdivision	OTU_4051	0.008	0.006	0.001	0.002	0.028
Bacteria	Verrucomic	Spartobacte	OTU_2401	0.007	0.007	0.000	0.000	0.042
Bacteria	Proteobacte	Unclassifie	OTU_2017	0.008	0.007	0.001	0.003	0.043
Bacteria	Proteobacte	Chelatococ	OTU_2012	0.006	0.006	0.000	0.000	0.029
Bacteria	Actinobacte	Iamia	OTU_2518	0.008	0.003	0.002	0.003	0.001
Bacteria	Acidobacte	Gp4	OTU_3425	0.007	0.007	0.001	0.002	0.050
Bacteria	Acidobacte	Gp3	OTU_4707	0.006	0.006	0.000	0.000	0.030
Bacteria	Planctomyc	Unclassifie	OTU_1478	0.006	0.005	0.000	0.000	0.014
Bacteria	Actinobacte	Gaiella	OTU_9030	0.006	0.005	0.000	0.000	0.011
Bacteria	Verrucomic	Subdivision	OTU_3939	0.006	0.005	0.000	0.000	0.014
Bacteria	Planctomyc	Gemmata	OTU_6905	0.006	0.006	0.000	0.000	0.035
Bacteria	candidate d	WPS-1_gen	OTU_2548	0.006	0.006	0.000	0.000	0.032
Bacteria	Armatimon	Armatimon	OTU_3113	0.006	0.006	0.000	0.000	0.025
Bacteria	Actinobacte	Catelliglob	OTU_3483	0.009	0.006	0.002	0.004	0.044
Bacteria	Latescibact	Latescibact	OTU_3746	0.006	0.005	0.000	0.000	0.016
Bacteria	candidate d	WPS-1_gen	OTU_9312	0.006	0.005	0.000	0.000	0.017
Bacteria	Unclassifie	Unclassifie	OTU_2080	0.006	0.006	0.000	0.000	0.037
Bacteria	Proteobacte	Unclassifie	OTU_2409	0.006	0.005	0.000	0.000	0.016
Bacteria	Actinobacte	Gaiella	OTU_3444	0.006	0.006	0.000	0.000	0.023
Bacteria	Armatimon	Armatimon	OTU_4866	0.006	0.005	0.000	0.000	0.011
Bacteria	Proteobacte	Unclassifie	OTU_3076	0.006	0.006	0.000	0.000	0.033
Bacteria	Armatimon	Armatimon	OTU_1951	0.007	0.004	0.001	0.002	0.007
Bacteria	Proteobacte	Unclassifie	OTU_2788	0.009	0.006	0.003	0.002	0.038
Bacteria	Bacteroidet	Lacibacter	OTU_1946	0.006	0.006	0.000	0.000	0.038
Bacteria	Proteobacte	Unclassifie	OTU_1165	0.006	0.006	0.001	0.001	0.035
Bacteria	Proteobacte	Unclassifie	OTU_3371	0.007	0.005	0.001	0.002	0.017
Bacteria	Actinobacte	Unclassifie	OTU_3396	0.006	0.005	0.000	0.000	0.019
Bacteria	Candidatus	Saccharibac	OTU_1958	0.006	0.005	0.000	0.001	0.013
Bacteria	Armatimon	Armatimon	OTU_1896	0.006	0.004	0.000	0.000	0.011
Bacteria	Acidobacte	Gp17	OTU_8421	0.006	0.003	0.000	0.000	0.002
Bacteria	Planctomyc	Unclassifie	OTU_2584	0.006	0.006	0.000	0.000	0.036
Bacteria	candidate d	WPS-1_gen	OTU_6717	0.006	0.006	0.000	0.000	0.045
Bacteria	Actinobacte	Ilumatobac	OTU_3819	0.007	0.006	0.001	0.002	0.048
Bacteria	Proteobacte	Unclassifie	OTU_1459	0.006	0.005	0.000	0.000	0.029
Bacteria	Planctomyc	Zavarzinell	OTU_5824	0.006	0.005	0.000	0.000	0.014
Bacteria	Actinobacte	Gaiella	OTU_5514	0.006	0.006	0.000	0.000	0.032
Bacteria	Planctomyc	Zavarzinell	OTU_2846	0.008	0.006	0.002	0.002	0.041

Bacteria	Planctomyc	Unclassifie	OTU_3806	0.006	0.004	0.000	0.000	0.012
Bacteria	Verrucomic	Spartobacte	OTU_2866	0.006	0.006	0.000	0.000	0.033
Bacteria	Latescibact	Latescibact	OTU_4546	0.005	0.005	0.000	0.000	0.019
Bacteria	Proteobacte	Unclassifie	OTU_1323	0.005	0.003	0.000	0.000	0.002
Bacteria	Unclassifie	Unclassifie	OTU_2504	0.005	0.004	0.000	0.000	0.008
Bacteria	Bacteroidet	Unclassifie	OTU_2087	0.005	0.006	0.000	0.000	0.044
Bacteria	Gemmatim	Gemmatim	OTU_6895	0.005	0.005	0.000	0.000	0.031
Bacteria	Planctomyc	Tepidispha	OTU_5781	0.005	0.006	0.000	0.000	0.042
Bacteria	Acidobacte	Gp6	OTU_3945	0.005	0.005	0.000	0.000	0.032
Bacteria	Verrucomic	Unclassifie	OTU_2451	0.007	0.005	0.001	0.002	0.031
Bacteria	Planctomyc	Rubinispha	OTU_3817	0.006	0.005	0.001	0.002	0.033
Bacteria	Acidobacte	Gp6	OTU_3325	0.005	0.005	0.000	0.000	0.035
Bacteria	Planctomyc	Planctomic	OTU_3460	0.005	0.003	0.000	0.000	0.002
Bacteria	Unclassifie	Unclassifie	OTU_3540	0.007	0.004	0.001	0.002	0.011
Bacteria	Proteobacte	Unclassifie	OTU_885	0.005	0.005	0.000	0.000	0.036
Bacteria	Unclassifie	Unclassifie	OTU_7035	0.005	0.004	0.000	0.000	0.013
Bacteria	Acidobacte	Gp6	OTU_8678	0.005	0.003	0.000	0.000	0.004
Bacteria	Armatimon	Armatimon	OTU_4155	0.005	0.004	0.000	0.000	0.017
Bacteria	Bacteroidet	Ferruginiba	OTU_4321	0.006	0.005	0.001	0.001	0.022
Bacteria	Acidobacte	Gp16	OTU_2624	0.005	0.005	0.000	0.000	0.029
Bacteria	Actinobact	Gaiella	OTU_7254	0.009	0.004	0.004	0.004	0.037
Bacteria	Chloroflexi	Unclassifie	OTU_6189	0.005	0.005	0.000	0.000	0.021
Bacteria	Unclassifie	Unclassifie	OTU_2567	0.005	0.004	0.000	0.000	0.013
Bacteria	Unclassifie	Unclassifie	OTU_5952	0.005	0.005	0.000	0.000	0.037
Bacteria	Actinobact	Aquihabita	OTU_7295	0.009	0.005	0.004	0.003	0.030
Bacteria	Proteobacte	Unclassifie	OTU_2909	0.005	0.004	0.000	0.000	0.008
Bacteria	Planctomyc	Blastopirel	OTU_6162	0.005	0.004	0.000	0.000	0.013
Bacteria	Verrucomic	Unclassifie	OTU_4063	0.005	0.006	0.000	0.000	0.050
Bacteria	Verrucomic	Spartobacte	OTU_3578	0.006	0.005	0.001	0.001	0.025
Bacteria	Unclassifie	Unclassifie	OTU_3601	0.005	0.005	0.000	0.000	0.031
Bacteria	Proteobacte	Paracoccus	OTU_1699	0.005	0.004	0.000	0.000	0.007
Bacteria	Proteobacte	Unclassifie	OTU_6521	0.005	0.005	0.000	0.000	0.032
Bacteria	Actinobact	Unclassifie	OTU_1598	0.008	0.004	0.003	0.003	0.019
Bacteria	Proteobacte	Haliangium	OTU_3125	0.005	0.005	0.000	0.000	0.040
Bacteria	Acidobacte	Gp6	OTU_5353	0.005	0.005	0.000	0.000	0.028
Bacteria	Actinobact	Conexibact	OTU_2276	0.006	0.005	0.001	0.002	0.050
Bacteria	Proteobacte	Unclassifie	OTU_1715	0.005	0.005	0.000	0.000	0.029
Bacteria	Proteobacte	Unclassifie	OTU_4636	0.005	0.005	0.000	0.000	0.028
Bacteria	Acidobacte	Gp6	OTU_2883	0.006	0.005	0.001	0.002	0.034
Bacteria	Proteobacte	Unclassifie	OTU_8293	0.006	0.004	0.002	0.003	0.033
Bacteria	Planctomyc	Planctopiru	OTU_2497	0.006	0.005	0.001	0.002	0.047
Bacteria	Actinobact	Unclassifie	OTU_3321	0.005	0.003	0.000	0.000	0.007
Bacteria	candidate d	WPS-1_gei	OTU_4134	0.005	0.005	0.000	0.000	0.031
Bacteria	Proteobacte	Unclassifie	OTU_6843	0.005	0.005	0.000	0.000	0.034
Bacteria	Actinobact	Unclassifie	OTU_2166	0.006	0.003	0.001	0.001	0.005
Bacteria	Chloroflexi	Ornatilinea	OTU_3850	0.005	0.004	0.000	0.000	0.029
Bacteria	Proteobacte	Unclassifie	OTU_9466	0.005	0.005	0.000	0.000	0.046
Bacteria	Actinobact	Solirubroba	OTU_7926	0.005	0.004	0.000	0.000	0.014
Bacteria	Actinobact	Ferrimicrol	OTU_4303	0.005	0.004	0.000	0.000	0.015
Bacteria	Acidobacte	Gp6	OTU_2450	0.005	0.005	0.000	0.000	0.032
Bacteria	Bacteroidet	Unclassifie	OTU_1821	0.006	0.003	0.001	0.002	0.012
Bacteria	Gemmatim	Gemmatim	OTU_5098	0.004	0.004	0.000	0.000	0.030
Bacteria	Gemmatim	Gemmatim	OTU_2111	0.004	0.004	0.000	0.000	0.016
Bacteria	Proteobacte	Labilithrix	OTU_5862	0.004	0.004	0.000	0.000	0.032
Bacteria	Planctomyc	Unclassifie	OTU_7472	0.004	0.005	0.000	0.000	0.043
Bacteria	Acidobacte	Gp10	OTU_4730	0.004	0.004	0.000	0.000	0.031

Bacteria	Bacteroidet	Terrimonas	OTU_6910	0.004	0.005	0.000	0.000	0.042
Bacteria	Acidobacte	Gp5	OTU_2586	0.004	0.004	0.000	0.000	0.021
Bacteria	Proteobacte	Unclassifie	OTU_3422	0.004	0.004	0.000	0.000	0.023
Bacteria	Proteobacte	Unclassifie	OTU_7894	0.006	0.004	0.001	0.002	0.030
Bacteria	Planctomyc	Zavarzinell	OTU_5643	0.004	0.005	0.000	0.000	0.049
Bacteria	Unclassifie	Unclassifie	OTU_4293	0.004	0.004	0.000	0.000	0.029
Bacteria	Proteobacte	Unclassifie	OTU_3993	0.004	0.004	0.000	0.000	0.026
Bacteria	Gemmatim	Gemmatim	OTU_2871	0.004	0.004	0.000	0.000	0.026
Bacteria	Proteobacte	Bdellovibri	OTU_3717	0.004	0.004	0.000	0.000	0.031
Bacteria	Actinobact	Unclassifie	OTU_5208	0.004	0.004	0.000	0.000	0.024
Bacteria	BRC1	BRC1_gen	OTU_4523	0.004	0.004	0.000	0.000	0.046
Bacteria	Armatimon	Armatimon	OTU_3382	0.005	0.004	0.001	0.001	0.027
Bacteria	candidate d	WPS-1_ge	OTU_3121	0.004	0.004	0.000	0.000	0.026
Bacteria	Unclassifie	Unclassifie	OTU_4642	0.004	0.004	0.000	0.000	0.048
Bacteria	Proteobacte	Unclassifie	OTU_4898	0.004	0.004	0.000	0.000	0.020
Bacteria	Chloroflexi	Bellilinea	OTU_1380	0.005	0.003	0.001	0.002	0.013
Bacteria	Candidatus	Saccharibac	OTU_1664	0.004	0.004	0.000	0.000	0.045
Bacteria	Proteobacte	Microvirga	OTU_3910	0.004	0.004	0.000	0.000	0.022
Bacteria	Actinobact	Unclassifie	OTU_2916	0.004	0.003	0.000	0.000	0.018
Archaea	Thaumarch	Nitrososph	OTU_54	0.004	0.004	0.000	0.000	0.046
Bacteria	Proteobacte	Unclassifie	OTU_2187	0.004	0.004	0.000	0.000	0.040
Bacteria	Proteobacte	Aureimona	OTU_2081	0.004	0.003	0.000	0.000	0.016
Bacteria	Bacteroidet	Unclassifie	OTU_7622	0.004	0.004	0.000	0.000	0.038
Bacteria	Bacteroidet	Terrimonas	OTU_3283	0.004	0.003	0.000	0.000	0.005
Bacteria	Actinobact	Actinomyc	OTU_2063	0.006	0.002	0.002	0.003	0.027
Bacteria	Chloroflexi	Unclassifie	OTU_4666	0.004	0.004	0.000	0.000	0.040
Bacteria	Unclassifie	Unclassifie	OTU_4753	0.004	0.004	0.000	0.000	0.036
Bacteria	Proteobacte	Unclassifie	OTU_2472	0.004	0.003	0.000	0.000	0.021
Bacteria	Armatimon	Chthonom	OTU_2108	0.004	0.004	0.000	0.000	0.035
Bacteria	Actinobact	Ilumatobac	OTU_7858	0.004	0.004	0.000	0.000	0.030
Bacteria	Actinobact	Unclassifie	OTU_2685	0.004	0.004	0.000	0.000	0.034
Bacteria	Proteobacte	Unclassifie	OTU_4647	0.004	0.002	0.000	0.000	0.005
Bacteria	Acidobacte	Gp6	OTU_7291	0.004	0.004	0.000	0.000	0.045
Bacteria	Unclassifie	Unclassifie	OTU_4196	0.004	0.004	0.000	0.000	0.030
Bacteria	Chloroflexi	Litorilinea	OTU_5391	0.004	0.003	0.000	0.000	0.021
Bacteria	Proteobacte	Rhizomicro	OTU_2172	0.004	0.004	0.000	0.001	0.035
Bacteria	Actinobact	Unclassifie	OTU_3650	0.004	0.003	0.000	0.000	0.024
Bacteria	Planctomyc	Thermogut	OTU_4149	0.004	0.004	0.000	0.000	0.038
Bacteria	Proteobacte	Unclassifie	OTU_2820	0.004	0.004	0.000	0.000	0.047
Bacteria	Acidobacte	Gp4	OTU_5736	0.004	0.004	0.000	0.000	0.050
Bacteria	Proteobacte	Unclassifie	OTU_6371	0.005	0.004	0.001	0.002	0.041
Bacteria	Bacteroidet	Ohtaekwan	OTU_1120	0.003	0.002	0.000	0.000	0.002
Bacteria	Actinobact	Nocardioid	OTU_4117	0.003	0.004	0.000	0.000	0.047
Bacteria	Acidobacte	Gp1	OTU_2070	0.003	0.004	0.000	0.000	0.043
Bacteria	Unclassifie	Unclassifie	OTU_7617	0.003	0.003	0.000	0.000	0.029
Bacteria	Unclassifie	Unclassifie	OTU_2284	0.003	0.003	0.000	0.000	0.029
Bacteria	Armatimon	Chthonom	OTU_2731	0.003	0.002	0.000	0.000	0.005
Bacteria	Proteobacte	Unclassifie	OTU_6078	0.003	0.004	0.000	0.000	0.047
Bacteria	Verrucomi	Luteolibact	OTU_3787	0.003	0.003	0.000	0.000	0.035
Bacteria	Proteobacte	Unclassifie	OTU_3281	0.003	0.003	0.000	0.000	0.012
Bacteria	Verrucomi	Unclassifie	OTU_2869	0.003	0.003	0.000	0.000	0.038
Bacteria	Armatimon	Chthonom	OTU_4176	0.003	0.003	0.000	0.000	0.038
Bacteria	Planctomyc	Unclassifie	OTU_3721	0.003	0.003	0.000	0.000	0.019
Bacteria	Acidobacte	Gp6	OTU_5299	0.003	0.003	0.000	0.000	0.012
Bacteria	Acidobacte	Bryobacter	OTU_1053	0.003	0.003	0.000	0.000	0.026
Bacteria	Planctomyc	Zavarzinell	OTU_6146	0.003	0.004	0.000	0.000	0.046

Bacteria	Unclassifie	Unclassifie	OTU_2980	0.003	0.003	0.000	0.000	0.043
Bacteria	Firmicutes	Clostridiun	OTU_4568	0.003	0.003	0.000	0.000	0.022
Bacteria	Unclassifie	Unclassifie	OTU_2422	0.003	0.003	0.000	0.000	0.043
Bacteria	Proteobacte	Sandaracin	OTU_6792	0.003	0.003	0.000	0.000	0.025
Bacteria	Acidobacte	Gp6	OTU_1157	0.003	0.003	0.000	0.000	0.017
Bacteria	Proteobacte	Azonexus	OTU_2893	0.003	0.003	0.000	0.000	0.041
Bacteria	Planctomyc	Unclassifie	OTU_3269	0.003	0.003	0.000	0.000	0.048
Bacteria	Bacteroidet	Unclassifie	OTU_4673	0.003	0.003	0.000	0.000	0.048
Bacteria	Unclassifie	Unclassifie	OTU_1547	0.003	0.003	0.000	0.000	0.015
Bacteria	Planctomyc	Unclassifie	OTU_2204	0.003	0.003	0.000	0.000	0.041
Bacteria	Bacteroidet	Unclassifie	OTU_1842	0.003	0.003	0.000	0.000	0.037
Bacteria	Proteobacte	Unclassifie	OTU_3921	0.003	0.003	0.000	0.000	0.035
Bacteria	Acidobacte	Gp3	OTU_2324	0.003	0.003	0.000	0.000	0.048
Bacteria	Chloroflexi	Unclassifie	OTU_5913	0.003	0.003	0.000	0.000	0.036
Bacteria	Proteobacte	Unclassifie	OTU_1182	0.003	0.003	0.000	0.000	0.038
Bacteria	Verrucomic	Subdivisioi	OTU_4741	0.003	0.003	0.000	0.000	0.042
Bacteria	Planctomyc	Singulispha	OTU_3833	0.003	0.003	0.000	0.000	0.036
Bacteria	Bacteroidet	Unclassifie	OTU_6943	0.003	0.003	0.000	0.000	0.036
Bacteria	Verrucomic	Unclassifie	OTU_9266	0.003	0.003	0.000	0.000	0.021
Bacteria	Actinobact	Aciditerrin	OTU_4042	0.003	0.003	0.000	0.000	0.037
Bacteria	Bacteroidet	Solitalea	OTU_6017	0.003	0.003	0.000	0.000	0.043
Bacteria	Acidobacte	Gp6	OTU_6021	0.003	0.003	0.000	0.000	0.045
Bacteria	Actinobact	Iamia	OTU_4186	0.003	0.003	0.000	0.000	0.048
Bacteria	Verrucomic	Unclassifie	OTU_6359	0.003	0.002	0.000	0.000	0.015
Bacteria	Proteobacte	Peredibacte	OTU_3130	0.003	0.003	0.000	0.000	0.035
Bacteria	Acidobacte	Gp4	OTU_2370	0.003	0.003	0.000	0.000	0.033
Bacteria	Unclassifie	Unclassifie	OTU_6450	0.003	0.003	0.000	0.000	0.042
Bacteria	Proteobacte	Unclassifie	OTU_5012	0.003	0.003	0.000	0.000	0.026
Bacteria	Chloroflexi	Unclassifie	OTU_2094	0.003	0.002	0.000	0.000	0.019
Bacteria	Proteobacte	Unclassifie	OTU_8374	0.003	0.003	0.000	0.000	0.041
Bacteria	Unclassifie	Unclassifie	OTU_3481	0.003	0.003	0.000	0.000	0.041
Bacteria	Bacteroidet	Unclassifie	OTU_2214	0.003	0.002	0.000	0.000	0.016
Bacteria	Planctomyc	Singulispha	OTU_7458	0.003	0.003	0.000	0.000	0.033
Bacteria	Firmicutes	Tumebacill	OTU_2780	0.003	0.003	0.000	0.000	0.042
Bacteria	Actinobact	Aciditerrin	OTU_3900	0.003	0.003	0.000	0.000	0.032
Bacteria	Proteobacte	Unclassifie	OTU_2340	0.003	0.002	0.001	0.001	0.032
Bacteria	Acidobacte	Gp6	OTU_3950	0.003	0.003	0.000	0.000	0.048
Bacteria	Proteobacte	Labilithrix	OTU_5073	0.003	0.003	0.000	0.000	0.043
Bacteria	Proteobacte	Unclassifie	OTU_3470	0.003	0.002	0.000	0.000	0.032
Bacteria	Actinobact	Unclassifie	OTU_6102	0.002	0.003	0.000	0.000	0.047
Bacteria	Armatimon	Fimbriimor	OTU_3750	0.002	0.003	0.000	0.000	0.039
Bacteria	Actinobact	Unclassifie	OTU_7326	0.003	0.002	0.001	0.002	0.035
Bacteria	Planctomyc	Unclassifie	OTU_5593	0.002	0.003	0.000	0.000	0.038
Bacteria	Proteobacte	Unclassifie	OTU_1489	0.002	0.002	0.000	0.000	0.025
Bacteria	Proteobacte	Unclassifie	OTU_4547	0.002	0.003	0.000	0.000	0.046
Bacteria	Proteobacte	Unclassifie	OTU_5971	0.002	0.002	0.000	0.000	0.036
Bacteria	Chloroflexi	Oscillochlc	OTU_2519	0.002	0.003	0.000	0.000	0.049
Bacteria	Acidobacte	Gp16	OTU_5152	0.002	0.002	0.000	0.000	0.024
Bacteria	Armatimon	Armatimon	OTU_6140	0.002	0.002	0.000	0.000	0.028
Bacteria	Bacteroidet	Unclassifie	OTU_2879	0.002	0.002	0.000	0.000	0.034
Bacteria	Chloroflexi	Unclassifie	OTU_2403	0.002	0.003	0.000	0.000	0.049
Bacteria	Unclassifie	Unclassifie	OTU_8341	0.002	0.003	0.000	0.000	0.044
Bacteria	Proteobacte	Unclassifie	OTU_8681	0.002	0.003	0.000	0.000	0.047
Bacteria	Unclassifie	Unclassifie	OTU_3300	0.002	0.002	0.000	0.000	0.036
Bacteria	Planctomyc	Zavarzinell	OTU_3782	0.002	0.002	0.000	0.000	0.043
Bacteria	Proteobacte	Hephaestia	OTU_6054	0.002	0.002	0.000	0.000	0.050

Bacteria	Unclassifie	Unclassifie	OTU_3979	0.002	0.002	0.000	0.000	0.050
Bacteria	Planctomyc	Zavarzinell	OTU_4633	0.002	0.002	0.000	0.000	0.043
Bacteria	Armatimon	Armatimon	OTU_4794	0.002	0.002	0.000	0.000	0.028
Bacteria	Latescibact	Latescibact	OTU_6469	0.002	0.002	0.000	0.000	0.029
Bacteria	Unclassifie	Unclassifie	OTU_3417	0.002	0.002	0.000	0.000	0.048
Bacteria	Verrucomic	Subdivision	OTU_5519	0.002	0.002	0.000	0.000	0.033
Bacteria	Proteobacte	Unclassifie	OTU_3581	0.002	0.002	0.000	0.000	0.037
Bacteria	Verrucomic	Spartobacte	OTU_6381	0.002	0.002	0.000	0.000	0.044
Bacteria	Chloroflexi	Litorilinea	OTU_4531	0.002	0.002	0.000	0.000	0.049
Bacteria	Planctomyc	Unclassifie	OTU_5241	0.002	0.002	0.000	0.000	0.045
Bacteria	Unclassifie	Unclassifie	OTU_3164	0.002	0.002	0.000	0.000	0.043
Bacteria	Verrucomic	Unclassifie	OTU_6230	0.002	0.002	0.000	0.000	0.039
Bacteria	Firmicutes	Unclassifie	OTU_4045	0.002	0.002	0.000	0.000	0.039
Bacteria	Gemmatim	Gemmatim	OTU_2314	0.002	0.002	0.000	0.000	0.039
Bacteria	Verrucomic	Subdivision	OTU_7595	0.002	0.002	0.000	0.000	0.039
Bacteria	Planctomyc	Unclassifie	OTU_7054	0.002	0.002	0.000	0.000	0.024
Bacteria	Verrucomic	Subdivision	OTU_9149	0.002	0.002	0.000	0.000	0.024
Bacteria	Proteobacte	Unclassifie	OTU_4290	0.002	0.002	0.000	0.000	0.024
Bacteria	Planctomyc	Unclassifie	OTU_8632	0.002	0.002	0.000	0.000	0.042
Bacteria	Unclassifie	Unclassifie	OTU_3239	0.002	0.002	0.000	0.000	0.045
Bacteria	Unclassifie	Unclassifie	OTU_3916	0.002	0.002	0.000	0.000	0.043
Bacteria	Proteobacte	Unclassifie	OTU_3450	0.002	0.002	0.000	0.000	0.036
Bacteria	Proteobacte	Unclassifie	OTU_2148	0.002	0.002	0.000	0.000	0.041
Bacteria	Actinobact	Unclassifie	OTU_1569	0.000	0.000	0.001	0.002	0.044
Bacteria	Proteobacte	Bdellovibri	OTU_4890	0.000	0.000	0.002	0.002	0.044
Bacteria	Planctomyc	Gemmata	OTU_4740	0.000	0.000	0.002	0.002	0.049
Bacteria	Verrucomic	Opitutus	OTU_1037	0.000	0.000	0.002	0.002	0.048
Bacteria	Planctomyc	Singulispha	OTU_3550	0.000	0.000	0.002	0.002	0.048
Bacteria	Proteobacte	Unclassifie	OTU_4765	0.000	0.000	0.002	0.002	0.050
Bacteria	Chloroflexi	Unclassifie	OTU_6948	0.000	0.000	0.002	0.002	0.045
Bacteria	Proteobacte	Unclassifie	OTU_7467	0.000	0.000	0.002	0.002	0.041
Bacteria	Proteobacte	Unclassifie	OTU_9226	0.000	0.000	0.002	0.002	0.046
Bacteria	Firmicutes	Staphyloco	OTU_3968	0.000	0.000	0.002	0.002	0.048
Bacteria	Firmicutes	Unclassifie	OTU_3482	0.000	0.000	0.002	0.002	0.019
Bacteria	Unclassifie	Unclassifie	OTU_7259	0.000	0.000	0.002	0.002	0.032
Bacteria	Candidatus	Saccharibac	OTU_1757	0.000	0.000	0.002	0.002	0.034
Bacteria	Gemmatim	Gemmatim	OTU_2310	0.000	0.000	0.002	0.003	0.046
Bacteria	Proteobacte	Aquicella	OTU_5144	0.000	0.000	0.002	0.002	0.015
Bacteria	Unclassifie	Unclassifie	OTU_2678	0.000	0.000	0.003	0.003	0.050
Bacteria	Proteobacte	Unclassifie	OTU_3684	0.000	0.000	0.003	0.003	0.044
Bacteria	Firmicutes	Clostridium	OTU_4539	0.000	0.000	0.003	0.003	0.047
Bacteria	Unclassifie	Unclassifie	OTU_4877	0.000	0.000	0.003	0.003	0.040
Bacteria	candidate d	WPS-1_gen	OTU_2059	0.000	0.000	0.003	0.003	0.045
Bacteria	Actinobact	Iamia	OTU_3256	0.000	0.000	0.003	0.003	0.026
Bacteria	Proteobacte	Unclassifie	OTU_3021	0.000	0.000	0.003	0.003	0.025
Bacteria	Proteobacte	Unclassifie	OTU_8033	0.000	0.000	0.003	0.003	0.022
Bacteria	candidate d	WPS-1_gen	OTU_2598	0.000	0.000	0.003	0.003	0.049
Bacteria	Firmicutes	Clostridium	OTU_2821	0.000	0.000	0.003	0.003	0.037
Bacteria	Verrucomic	Subdivision	OTU_5912	0.000	0.000	0.003	0.003	0.019
Bacteria	Actinobact	Unclassifie	OTU_9188	0.000	0.000	0.003	0.002	0.007
Bacteria	Bacteroidet	Unclassifie	OTU_3883	0.000	0.000	0.003	0.003	0.035
Bacteria	Firmicutes	Thalassoba	OTU_6739	0.000	0.000	0.003	0.003	0.023
Bacteria	Proteobacte	Unclassifie	OTU_4058	0.000	0.000	0.004	0.003	0.019
Bacteria	Candidatus	Saccharibac	OTU_6373	0.000	0.000	0.004	0.004	0.048
Bacteria	Acidobacte	Unclassifie	OTU_1393	0.000	0.000	0.004	0.004	0.031
Bacteria	Unclassifie	Unclassifie	OTU_5778	0.000	0.000	0.004	0.003	0.015

Bacteria	Firmicutes	Clostridiun	OTU_1915	0.000	0.000	0.004	0.003	0.006
Bacteria	Planctomyc	Unclassifie	OTU_7268	0.000	0.000	0.004	0.004	0.036
Bacteria	Bacteroidet	Unclassifie	OTU_1324	0.000	0.000	0.004	0.004	0.016
Bacteria	Unclassifie	Unclassifie	OTU_4012	0.000	0.000	0.004	0.004	0.015
Bacteria	Proteobacte	Unclassifie	OTU_8462	0.000	0.000	0.004	0.004	0.018
Bacteria	Bacteroidet	Sediminiba	OTU_3193	0.000	0.000	0.004	0.004	0.025
Bacteria	Chloroflexi	Unclassifie	OTU_3807	0.000	0.000	0.005	0.004	0.016
Bacteria	Planctomyc	Unclassifie	OTU_2874	0.000	0.000	0.005	0.005	0.044
Bacteria	Candidatus	Saccharibac	OTU_977	0.000	0.000	0.005	0.005	0.031
Bacteria	Proteobacte	Unclassifie	OTU_3593	0.000	0.000	0.005	0.005	0.035
Bacteria	Armatimon	Armatimon	OTU_1395	0.000	0.000	0.005	0.005	0.045
Bacteria	Planctomyc	Aquisphaer	OTU_4215	0.000	0.000	0.005	0.004	0.009
Bacteria	Verrucomic	Terrimicrol	OTU_2488	0.000	0.000	0.005	0.004	0.009
Bacteria	Proteobacte	Asticcacau	OTU_1360	0.000	0.000	0.005	0.004	0.010
Bacteria	Proteobacte	Acidisoma	OTU_2262	0.000	0.000	0.005	0.005	0.020
Bacteria	Planctomyc	Unclassifie	OTU_2255	0.000	0.000	0.005	0.004	0.016
Bacteria	Actinobact	Unclassifie	OTU_6395	0.002	0.003	0.007	0.005	0.041
Bacteria	Proteobacte	Unclassifie	OTU_1595	0.000	0.000	0.005	0.005	0.026
Bacteria	Gemmatim	Gemmatim	OTU_9254	0.000	0.000	0.005	0.006	0.047
Bacteria	candidate d	WPS-1_ger	OTU_1838	0.001	0.002	0.007	0.005	0.038
Bacteria	Unclassifie	Unclassifie	OTU_3044	0.000	0.000	0.006	0.006	0.038
Bacteria	Bacteroidet	Mucilaginil	OTU_936	0.000	0.000	0.006	0.005	0.020
Bacteria	Unclassifie	Unclassifie	OTU_969	0.002	0.003	0.008	0.006	0.029
Bacteria	Proteobacte	Unclassifie	OTU_2199	0.000	0.000	0.006	0.007	0.048
Bacteria	Chloroflexi	Unclassifie	OTU_1333	0.000	0.000	0.006	0.006	0.026
Bacteria	Actinobact	Unclassifie	OTU_6638	0.000	0.000	0.006	0.005	0.020
Bacteria	Chloroflexi	Ktedonobac	OTU_3753	0.000	0.000	0.006	0.005	0.013
Bacteria	Armatimon	Chthonomo	OTU_1247	0.001	0.002	0.008	0.006	0.029
Bacteria	Unclassifie	Unclassifie	OTU_1014	0.000	0.000	0.007	0.007	0.046
Bacteria	Unclassifie	Unclassifie	OTU_2562	0.000	0.000	0.007	0.007	0.043
Bacteria	Unclassifie	Unclassifie	OTU_1766	0.000	0.000	0.007	0.007	0.034
Bacteria	Chloroflexi	Nitrolancea	OTU_1129	0.000	0.000	0.007	0.006	0.018
Bacteria	Acidobacte	Gp1	OTU_2238	0.000	0.000	0.007	0.007	0.043
Bacteria	Unclassifie	Unclassifie	OTU_1968	0.000	0.000	0.007	0.007	0.031
Bacteria	Proteobacte	Unclassifie	OTU_1266	0.001	0.002	0.008	0.007	0.027
Bacteria	Planctomyc	Aquisphaer	OTU_2139	0.001	0.002	0.008	0.005	0.010
Bacteria	Unclassifie	Unclassifie	OTU_2136	0.000	0.000	0.007	0.008	0.042
Bacteria	Actinobact	Amnibacter	OTU_7664	0.001	0.002	0.008	0.007	0.024
Bacteria	Unclassifie	Unclassifie	OTU_2130	0.000	0.000	0.007	0.008	0.049
Bacteria	Firmicutes	Sporosarcin	OTU_3520	0.002	0.003	0.010	0.006	0.010
Bacteria	Bacteroidet	Unclassifie	OTU_1841	0.002	0.003	0.010	0.007	0.021
Bacteria	Actinobact	Aciditerrim	OTU_7239	0.000	0.000	0.008	0.007	0.022
Bacteria	candidate d	WPS-1_ger	OTU_2234	0.002	0.005	0.010	0.005	0.013
Bacteria	Candidatus	Saccharibac	OTU_1845	0.000	0.000	0.008	0.008	0.031
Bacteria	Candidatus	Saccharibac	OTU_7465	0.000	0.000	0.008	0.006	0.006
Bacteria	Unclassifie	Unclassifie	OTU_1322	0.004	0.004	0.013	0.008	0.029
Bacteria	candidate d	WPS-2_ger	OTU_863	0.001	0.003	0.010	0.008	0.019
Bacteria	Candidatus	Saccharibac	OTU_979	0.000	0.000	0.009	0.008	0.017
Bacteria	Firmicutes	Pullulaniba	OTU_3068	0.000	0.000	0.009	0.008	0.015
Bacteria	Armatimon	Chthonomo	OTU_2441	0.000	0.000	0.009	0.010	0.046
Bacteria	candidate d	WPS-2_ger	OTU_1523	0.000	0.000	0.009	0.009	0.025
Bacteria	Actinobact	Blastococc	OTU_6665	0.002	0.002	0.012	0.009	0.029
Bacteria	Verrucomic	Subdivision	OTU_7076	0.000	0.000	0.010	0.009	0.026
Bacteria	Proteobacte	Unclassifie	OTU_6194	0.000	0.000	0.010	0.010	0.027
Bacteria	Planctomyc	Gemmata	OTU_1575	0.000	0.000	0.011	0.011	0.037
Bacteria	Firmicutes	Unclassifie	OTU_3847	0.008	0.009	0.019	0.006	0.020

Bacteria	Proteobacte	Unclassifie	OTU_2730	0.000	0.000	0.011	0.009	0.013
Bacteria	Unclassifie	Unclassifie	OTU_1505	0.000	0.000	0.011	0.012	0.041
Bacteria	Proteobacte	Acidisoma	OTU_5390	0.010	0.009	0.023	0.010	0.029
Bacteria	Proteobacte	Acidisoma	OTU_1325	0.000	0.000	0.013	0.008	0.005
Bacteria	Actinobact	Actinocate	OTU_1826	0.000	0.000	0.013	0.013	0.037
Bacteria	Unclassifie	Unclassifie	OTU_1265	0.000	0.000	0.013	0.011	0.017
Bacteria	Planctomyc	Unclassifie	OTU_1065	0.001	0.002	0.015	0.012	0.022
Bacteria	Gemmatim	Gemmatim	OTU_2688	0.001	0.002	0.015	0.014	0.037
Bacteria	Planctomyc	Gemmata	OTU_4018	0.000	0.000	0.014	0.015	0.041
Bacteria	Planctomyc	Unclassifie	OTU_1734	0.000	0.000	0.015	0.017	0.049
Bacteria	Proteobacte	Unclassifie	OTU_559	0.004	0.005	0.020	0.013	0.019
Bacteria	Planctomyc	Singulispha	OTU_843	0.007	0.007	0.023	0.012	0.010
Bacteria	Proteobacte	Unclassifie	OTU_1181	0.001	0.002	0.017	0.009	0.003
Bacteria	candidate d	WPS-1_ge	OTU_1007	0.003	0.004	0.019	0.012	0.008
Bacteria	Candidatus	Saccharibac	OTU_6023	0.000	0.000	0.016	0.018	0.049
Bacteria	Proteobacte	Castellanie	OTU_233	0.008	0.009	0.024	0.014	0.022
Bacteria	Proteobacte	Azospirillu	OTU_620	0.002	0.003	0.018	0.016	0.030
Bacteria	Unclassifie	Unclassifie	OTU_1049	0.000	0.000	0.017	0.015	0.020
Bacteria	Acidobacte	Gp1	OTU_727	0.007	0.008	0.024	0.016	0.026
Bacteria	Planctomyc	Gemmata	OTU_887	0.000	0.000	0.018	0.011	0.003
Bacteria	Proteobacte	Ensifer	OTU_3823	0.006	0.007	0.024	0.019	0.047
Bacteria	Acidobacte	Unclassifie	OTU_989	0.000	0.000	0.018	0.013	0.007
Bacteria	Actinobact	Nocardioid	OTU_1994	0.004	0.004	0.021	0.013	0.008
Bacteria	Unclassifie	Unclassifie	OTU_668	0.002	0.004	0.020	0.013	0.006
Bacteria	Acidobacte	Acidipila	OTU_336	0.010	0.013	0.028	0.015	0.025
Bacteria	Planctomyc	Gemmata	OTU_660	0.003	0.003	0.021	0.010	0.001
Bacteria	Chloroflexi	Ktedonobac	OTU_1485	0.001	0.003	0.022	0.019	0.025
Bacteria	Verrucomic	Subdivisio	OTU_932	0.006	0.009	0.028	0.023	0.047
Bacteria	Chloroflexi	Unclassifie	OTU_872	0.004	0.003	0.025	0.017	0.012
Bacteria	Proteobacte	Reyranella	OTU_451	0.008	0.007	0.030	0.015	0.005
Bacteria	Proteobacte	Unclassifie	OTU_804	0.003	0.005	0.025	0.017	0.010
Bacteria	Proteobacte	Unclassifie	OTU_1412	0.001	0.002	0.025	0.019	0.014
Bacteria	Proteobacte	Unclassifie	OTU_532	0.000	0.000	0.024	0.022	0.027
Bacteria	Proteobacte	Unclassifie	OTU_7486	0.005	0.008	0.030	0.018	0.007
Bacteria	Unclassifie	Unclassifie	OTU_861	0.002	0.003	0.026	0.027	0.050
Bacteria	Gemmatim	Gemmatim	OTU_531	0.007	0.007	0.032	0.028	0.047
Bacteria	candidate d	WPS-1_ge	OTU_730	0.003	0.004	0.029	0.027	0.038
Bacteria	Planctomyc	Aquisphae	OTU_1610	0.007	0.007	0.034	0.018	0.006
Bacteria	Bacteroide	Niabella	OTU_4262	0.001	0.002	0.029	0.029	0.041
Bacteria	Actinobact	Nocardioid	OTU_925	0.006	0.010	0.035	0.019	0.004
Bacteria	Planctomyc	Singulispha	OTU_2074	0.032	0.017	0.062	0.017	0.005
Bacteria	Candidatus	Saccharibac	OTU_737	0.000	0.000	0.032	0.031	0.030
Bacteria	Chloroflexi	Sphaerobac	OTU_405	0.009	0.010	0.042	0.026	0.013
Bacteria	Acidobacte	Terriglobus	OTU_8499	0.009	0.008	0.042	0.027	0.014
Bacteria	Chloroflexi	Unclassifie	OTU_1551	0.000	0.000	0.033	0.026	0.011
Bacteria	Gemmatim	Gemmatim	OTU_708	0.000	0.000	0.034	0.033	0.032
Bacteria	Proteobacte	Unclassifie	OTU_616	0.006	0.007	0.040	0.017	0.001
Bacteria	Actinobact	Unclassifie	OTU_243	0.010	0.008	0.044	0.029	0.017
Bacteria	Acidobacte	Gp6	OTU_700	0.000	0.000	0.035	0.028	0.012
Bacteria	Chloroflexi	Ktedonobac	OTU_929	0.008	0.010	0.044	0.039	0.047
Bacteria	Gemmatim	Gemmatim	OTU_488	0.003	0.003	0.038	0.028	0.013
Bacteria	Proteobacte	Candidimo	OTU_287	0.008	0.008	0.047	0.034	0.021
Bacteria	Proteobacte	Roseiarcus	OTU_745	0.011	0.012	0.051	0.033	0.015
Bacteria	Acidobacte	Unclassifie	OTU_478	0.014	0.010	0.055	0.037	0.023
Bacteria	Unclassifie	Unclassifie	OTU_894	0.000	0.000	0.042	0.037	0.021
Bacteria	Acidobacte	Candidatus	OTU_1005	0.016	0.013	0.058	0.029	0.005

Bacteria	Proteobacte	Unclassifie	OTU_4198	0.001	0.002	0.045	0.026	0.003
Bacteria	Proteobacte	Haliangium	OTU_395	0.025	0.016	0.071	0.044	0.029
Bacteria	Proteobacte	Unclassifie	OTU_390	0.016	0.016	0.063	0.039	0.016
Bacteria	Bacteroidet	Unclassifie	OTU_644	0.015	0.022	0.062	0.037	0.014
Bacteria	Chloroflexi	Unclassifie	OTU_413	0.000	0.000	0.049	0.053	0.045
Bacteria	Acidobacte	Gp14	OTU_232	0.000	0.000	0.049	0.042	0.017
Bacteria	Proteobacte	Unclassifie	OTU_853	0.001	0.002	0.050	0.032	0.005
Bacteria	Acidobacte	Gp3	OTU_442	0.017	0.018	0.066	0.042	0.017
Bacteria	Bacteroidet	Unclassifie	OTU_85	0.028	0.026	0.079	0.042	0.019
Bacteria	Unclassifie	Unclassifie	OTU_290	0.003	0.006	0.054	0.042	0.014
Bacteria	Chloroflexi	Nitrolancea	OTU_357	0.009	0.008	0.061	0.032	0.004
Bacteria	Actinobacte	Acidimicro	OTU_569	0.005	0.005	0.056	0.046	0.021
Bacteria	Bacteroidet	Unclassifie	OTU_313	0.002	0.002	0.055	0.058	0.043
Bacteria	Unclassifie	Unclassifie	OTU_648	0.000	0.000	0.055	0.031	0.002
Bacteria	Proteobacte	Unclassifie	OTU_343	0.001	0.001	0.056	0.055	0.031
Bacteria	Unclassifie	Unclassifie	OTU_143	0.033	0.032	0.091	0.055	0.033
Bacteria	candidate d	WPS-1_ge	OTU_6644	0.003	0.005	0.063	0.037	0.003
Bacteria	Candidatus	Sacchariba	OTU_4974	0.000	0.000	0.067	0.057	0.018
Bacteria	Acidobacte	Telmatobac	OTU_3128	0.003	0.004	0.072	0.062	0.023
Bacteria	Proteobacte	Unclassifie	OTU_545	0.004	0.005	0.075	0.056	0.013
Bacteria	Acidobacte	Gp1	OTU_5328	0.002	0.003	0.074	0.080	0.050
Bacteria	Proteobacte	Unclassifie	OTU_157	0.034	0.015	0.107	0.024	0.000
Bacteria	Unclassifie	Unclassifie	OTU_197	0.009	0.006	0.082	0.049	0.005
Bacteria	Proteobacte	Rhizomicro	OTU_361	0.015	0.028	0.088	0.073	0.034
Bacteria	Bacteroidet	Unclassifie	OTU_4221	0.001	0.002	0.078	0.086	0.049
Bacteria	Planctomyc	Singulispha	OTU_406	0.012	0.011	0.090	0.082	0.040
Bacteria	Proteobacte	Tahibacter	OTU_114	0.017	0.015	0.098	0.064	0.012
Bacteria	Acidobacte	Gp1	OTU_1367	0.000	0.000	0.084	0.054	0.004
Bacteria	Acidobacte	Gp1	OTU_574	0.002	0.003	0.087	0.091	0.041
Bacteria	Proteobacte	Unclassifie	OTU_386	0.022	0.009	0.108	0.053	0.003
Bacteria	Planctomyc	Planctopiru	OTU_214	0.031	0.030	0.118	0.042	0.001
Bacteria	Proteobacte	Dokdonella	OTU_4322	0.019	0.008	0.108	0.063	0.008
Bacteria	Gemmatim	Gemmatim	OTU_98	0.018	0.013	0.107	0.091	0.035
Bacteria	Proteobacte	Lacibacteri	OTU_323	0.005	0.006	0.095	0.088	0.031
Bacteria	Proteobacte	Unclassifie	OTU_549	0.010	0.012	0.103	0.058	0.003
Bacteria	Proteobacte	Unclassifie	OTU_351	0.004	0.006	0.103	0.068	0.006
Bacteria	Acidobacte	Acidobacte	OTU_318	0.007	0.004	0.109	0.073	0.008
Bacteria	Proteobacte	Unclassifie	OTU_8878	0.000	0.000	0.104	0.099	0.027
Bacteria	Actinobacte	Actinospica	OTU_237	0.029	0.027	0.135	0.110	0.040
Bacteria	Chloroflexi	Unclassifie	OTU_398	0.001	0.002	0.109	0.094	0.019
Bacteria	Chloroflexi	Ktedonoba	OTU_348	0.014	0.014	0.127	0.068	0.003
Bacteria	Actinobacte	Unclassifie	OTU_671	0.036	0.025	0.149	0.089	0.012
Bacteria	Gemmatim	Gemmatim	OTU_1117	0.005	0.008	0.119	0.127	0.048
Bacteria	Actinobacte	Gaiella	OTU_206	0.082	0.062	0.198	0.077	0.008
Bacteria	Proteobacte	Rhizomicro	OTU_164	0.112	0.087	0.230	0.097	0.031
Bacteria	Proteobacte	Pseudolabr	OTU_52	0.108	0.067	0.228	0.072	0.006
Bacteria	Actinobacte	Unclassifie	OTU_8284	0.008	0.007	0.130	0.110	0.021
Bacteria	Proteobacte	Kerstersia	OTU_259	0.002	0.004	0.132	0.118	0.023
Bacteria	Gemmatim	Gemmatim	OTU_99	0.031	0.031	0.170	0.132	0.027
Bacteria	Actinobacte	Acidotherm	OTU_360	0.008	0.009	0.147	0.065	0.001
Bacteria	Verrucomic	Subdivision	OTU_234	0.066	0.072	0.208	0.094	0.008
Bacteria	Actinobacte	Mycobacte	OTU_100	0.048	0.054	0.196	0.124	0.017
Bacteria	Actinobacte	Geodermat	OTU_5883	0.021	0.011	0.174	0.135	0.020
Bacteria	Acidobacte	Granulicell	OTU_140	0.055	0.032	0.213	0.143	0.023
Bacteria	Proteobacte	Unclassifie	OTU_107	0.023	0.020	0.190	0.137	0.014
Bacteria	candidate d	WPS-2_ge	OTU_186	0.015	0.017	0.198	0.164	0.021

Bacteria	Proteobacte	Unclassifie	OTU_9131	0.056	0.066	0.241	0.130	0.007
Bacteria	Proteobacte	Lacibacteri	OTU_156	0.004	0.005	0.190	0.156	0.016
Bacteria	Chloroflexi	Unclassifie	OTU_59	0.041	0.052	0.236	0.191	0.031
Bacteria	Actinobact	Unclassifie	OTU_51	0.103	0.094	0.298	0.207	0.047
Bacteria	Acidobacte	Terriglobus	OTU_188	0.029	0.019	0.229	0.058	0.000
Bacteria	Proteobacte	Skermanell	OTU_129	0.008	0.010	0.210	0.222	0.048
Bacteria	Acidobacte	Gp3	OTU_80	0.075	0.027	0.301	0.176	0.011
Bacteria	Planctomyc	Thermogut	OTU_187	0.035	0.032	0.274	0.132	0.002
Bacteria	Unclassifie	Unclassifie	OTU_144	0.008	0.011	0.256	0.190	0.010
Bacteria	Actinobact	Gaiella	OTU_275	0.091	0.057	0.357	0.129	0.001
Bacteria	Acidobacte	Unclassifie	OTU_609	0.003	0.003	0.273	0.286	0.041
Bacteria	Chloroflexi	Unclassifie	OTU_37	0.118	0.093	0.409	0.286	0.032
Bacteria	Chloroflexi	Unclassifie	OTU_112	0.071	0.076	0.374	0.270	0.021
Bacteria	Actinobact	Catenulispo	OTU_200	0.141	0.122	0.446	0.241	0.013
Bacteria	Acidobacte	Unclassifie	OTU_91	0.117	0.101	0.458	0.358	0.041
Bacteria	Proteobacte	Unclassifie	OTU_92	0.005	0.011	0.347	0.320	0.026
Bacteria	Proteobacte	Unclassifie	OTU_367	0.012	0.013	0.362	0.249	0.007
Bacteria	Acidobacte	Gp1	OTU_26	0.184	0.135	0.544	0.312	0.019
Bacteria	Proteobacte	Unclassifie	OTU_151	0.010	0.015	0.374	0.203	0.002
Bacteria	Proteobacte	Sphingomo	OTU_49	0.148	0.096	0.519	0.218	0.002
Bacteria	Proteobacte	Frateuria	OTU_346	0.058	0.030	0.454	0.193	0.001
Bacteria	Chloroflexi	Unclassifie	OTU_57	0.048	0.054	0.470	0.334	0.012
Bacteria	Unclassifie	Unclassifie	OTU_15	0.062	0.036	0.487	0.364	0.018
Bacteria	Proteobacte	Unclassifie	OTU_27	0.095	0.065	0.521	0.362	0.017
Bacteria	Proteobacte	Burkholder	OTU_28	0.136	0.090	0.600	0.269	0.002
Bacteria	candidate d	WPS-1_ge	OTU_11	0.122	0.099	0.639	0.442	0.017
Bacteria	Actinobact	Unclassifie	OTU_10	0.629	0.176	1.212	0.467	0.013
Bacteria	Actinobact	Gaiella	OTU_25	0.478	0.213	1.064	0.489	0.016
Bacteria	Acidobacte	Gp1	OTU_61	0.017	0.022	0.769	0.578	0.011
Bacteria	Acidobacte	Geothrix	OTU_18	0.132	0.070	0.905	0.543	0.007
Bacteria	Actinobact	Unclassifie	OTU_84	0.035	0.042	0.818	0.525	0.006
Bacteria	Acidobacte	Gp2	OTU_39	0.031	0.031	0.868	0.544	0.005
Bacteria	Chloroflexi	Unclassifie	OTU_534	0.042	0.042	0.901	0.906	0.041
Bacteria	Bacteroidet	Unclassifie	OTU_14	0.114	0.167	0.998	0.888	0.034
Bacteria	Proteobacte	Unclassifie	OTU_5473	0.057	0.068	1.105	0.848	0.014
Bacteria	Proteobacte	Rhodanoba	OTU_5	0.331	0.284	1.916	1.106	0.006
Bacteria	Proteobacte	Unclassifie	OTU_4	0.234	0.263	2.427	1.510	0.006
Archaea	Thaumarch	Nitrososph	OTU_2	0.296	0.250	3.157	2.746	0.028
