# Supplementary material for: Microbial interactions and metabolisms in response to bacterial wilt and black shank pathogens in the tobacco rhizosphere
Source: Front Plant Sci. 2023 Jun 20;14:1200136. doi: 10.3389/fpls.2023.1200136 (PMC10319149; doi:10.3389/fpls.2023.1200136)
Supplement: Supplementary file 3 [file DataSheet_3.pdf]

Additional file 7

Table S7 Genera with significant differences between CK and BWH groups (Unit: %)

Domain	Phylum	Genus	OTU	CK: mean ı	CK: std. de	BWH: mea	BWH: std.	p-values
Bacteria	Actinobact	Streptomyc	OTU_8	2.532	1.773	0.574	0.213	0.022
Bacteria	Acidobacte	Gp4	OTU_74	1.449	0.811	0.053	0.079	0.003
Bacteria	Actinobact	Streptomyc	OTU_34	1.255	0.588	0.413	0.386	0.008
Bacteria	Actinobact	Streptomyc	OTU_3189	0.730	0.399	0.121	0.097	0.005
Bacteria	Proteobact	Sphingobiu	OTU_6	0.782	0.557	0.261	0.094	0.043
Bacteria	Actinobact	Amycolato	OTU_16	0.545	0.247	0.143	0.105	0.003
Bacteria	Verrucomi	Spartobact	OTU_71	0.705	0.366	0.341	0.167	0.038
Bacteria	Actinobact	Arthrobact	OTU_17	0.540	0.313	0.191	0.111	0.022
Bacteria	Actinobact	Janibacter	OTU_42	0.545	0.303	0.200	0.120	0.020
Bacteria	Acidobacte	Gp6	OTU_155	0.422	0.177	0.079	0.096	0.001
Bacteria	Actinobact	Marmorico	OTU_2959	0.404	0.151	0.120	0.073	0.001
Bacteria	Proteobact	Bradyrhizo	OTU_23	0.784	0.265	0.507	0.178	0.039
Bacteria	Acidobacte	Gp6	OTU_201	0.261	0.167	0.020	0.016	0.007
Bacteria	Acidobacte	Gp16	OTU_77	0.306	0.124	0.067	0.057	0.001
Bacteria	Proteobact	Unclassifie	OTU_4511	0.319	0.146	0.099	0.052	0.005
Bacteria	Proteobact	Sphingobiu	OTU_38	0.235	0.100	0.022	0.027	0.001
Bacteria	Proteobact	Unclassifie	OTU_134	0.216	0.136	0.013	0.018	0.005
Bacteria	Actinobact	Lechevalie	OTU_93	0.217	0.145	0.017	0.012	0.008
Bacteria	Unclassifie	Unclassifie	OTU_132	0.224	0.144	0.026	0.022	0.008
Bacteria	Acidobacte	Gp6	OTU_332	0.216	0.175	0.018	0.024	0.020
Bacteria	Acidobacte	Gp6	OTU_70	0.285	0.142	0.088	0.096	0.010
Bacteria	Acidobacte	Gp4	OTU_285	0.222	0.103	0.030	0.051	0.001
Bacteria	Candidatus	Saccharibac	OTU_76	0.174	0.145	0.000	0.000	0.016
Bacteria	Acidobacte	Gp6	OTU_118	0.235	0.090	0.061	0.066	0.001
Bacteria	Actinobact	Kribbella	OTU_96	0.214	0.071	0.041	0.028	0.000
Bacteria	Actinobact	Unclassifie	OTU_249	0.199	0.060	0.032	0.018	0.000
Bacteria	Proteobact	Unclassifie	OTU_202	0.174	0.106	0.010	0.012	0.005
Bacteria	Actinobact	Gaiella	OTU_289	0.245	0.145	0.081	0.057	0.021
Bacteria	Acidobacte	Gp6	OTU_8332	0.215	0.065	0.060	0.058	0.000
Bacteria	Proteobact	Ramlibacte	OTU_48	0.376	0.124	0.227	0.078	0.020
Bacteria	Verrucomi	Subdivisio	OTU_207	0.167	0.147	0.021	0.021	0.033
Bacteria	Acidobacte	Gp4	OTU_3764	0.146	0.099	0.000	0.000	0.006
Bacteria	Acidobacte	Gp4	OTU_1411	0.170	0.051	0.024	0.031	0.000
Bacteria	Gemmatim	Gemmatim	OTU_46	0.180	0.137	0.038	0.018	0.029
Bacteria	Proteobact	Phenylobac	OTU_41	0.276	0.135	0.135	0.096	0.042
Bacteria	Actinobact	Conexibact	OTU_291	0.164	0.085	0.025	0.023	0.003
Bacteria	Actinobact	Phycicoccu	OTU_7584	0.150	0.119	0.022	0.007	0.025
Bacteria	Actinobact	Unclassifie	OTU_8280	0.175	0.133	0.049	0.019	0.041
Bacteria	Acidobacte	Gp6	OTU_508	0.157	0.123	0.034	0.047	0.035
Bacteria	Unclassifie	Unclassifie	OTU_127	0.129	0.096	0.008	0.009	0.013
Bacteria	Verrucomi	Spartobact	OTU_432	0.154	0.107	0.039	0.035	0.026
Bacteria	Acidobacte	Gp6	OTU_110	0.135	0.075	0.021	0.022	0.005
Bacteria	Acidobacte	Gp4	OTU_2467	0.123	0.061	0.010	0.012	0.002
Bacteria	Proteobact	Unclassifie	OTU_4767	0.146	0.096	0.036	0.023	0.018
Bacteria	Proteobact	Unclassifie	OTU_341	0.155	0.081	0.045	0.029	0.008
Bacteria	Proteobact	Arenimona	OTU_130	0.170	0.067	0.062	0.059	0.007
Bacteria	Actinobact	Blastococc	OTU_115	0.133	0.078	0.033	0.025	0.011
Bacteria	Actinobact	Conexibact	OTU_211	0.123	0.041	0.026	0.012	0.000
Bacteria	Acidobacte	Gp4	OTU_7758	0.105	0.098	0.009	0.016	0.036
Bacteria	Actinobact	Solirubrob	OTU_497	0.098	0.082	0.005	0.005	0.019
Bacteria	Actinobact	Gaiella	OTU_896	0.096	0.091	0.003	0.005	0.030
Bacteria	candidate d	WPS-1_ge	OTU_464	0.097	0.085	0.005	0.006	0.024

Bacteria	Proteobacte	Unclassifie	OTU_618	0.107	0.048	0.016	0.009	0.001
Bacteria	Actinobacte	Aquihabita	OTU_299	0.133	0.078	0.046	0.048	0.027
Bacteria	Bacteroidet	Flavisoliba	OTU_120	0.145	0.058	0.059	0.031	0.006
Bacteria	Acidobacte	Gp4	OTU_750	0.093	0.060	0.007	0.015	0.006
Bacteria	Actinobacte	Rhodococc	OTU_40	0.137	0.073	0.054	0.034	0.022
Bacteria	Acidobacte	Aridibacter	OTU_407	0.107	0.065	0.025	0.020	0.012
Bacteria	Acidobacte	Gp4	OTU_8714	0.081	0.060	0.000	0.000	0.009
Bacteria	Actinobacte	Nocardioid	OTU_82	0.178	0.038	0.098	0.047	0.004
Bacteria	Acidobacte	Gp7	OTU_279	0.183	0.074	0.103	0.045	0.032
Bacteria	Actinobacte	Solirubroba	OTU_657	0.077	0.077	0.000	0.000	0.033
Bacteria	Acidobacte	Gp2	OTU_8528	0.112	0.064	0.036	0.036	0.018
Bacteria	Gemmatim	Gemmatim	OTU_296	0.087	0.079	0.012	0.015	0.039
Bacteria	Verrucomic	Unclassifie	OTU_304	0.080	0.052	0.006	0.006	0.007
Bacteria	Actinobacte	Gaiella	OTU_587	0.079	0.062	0.006	0.010	0.016
Bacteria	Actinobacte	Aciditerrim	OTU_638	0.087	0.073	0.014	0.020	0.032
Bacteria	Unclassifie	Unclassifie	OTU_1942	0.075	0.064	0.001	0.002	0.019
Bacteria	Acidobacte	Gp6	OTU_3585	0.091	0.051	0.019	0.019	0.007
Bacteria	Acidobacte	Gp6	OTU_610	0.073	0.078	0.001	0.002	0.045
Bacteria	Unclassifie	Unclassifie	OTU_506	0.076	0.056	0.004	0.004	0.012
Bacteria	Actinobacte	Unclassifie	OTU_7451	0.092	0.056	0.021	0.018	0.012
Bacteria	Actinobacte	Gaiella	OTU_1345	0.073	0.048	0.003	0.003	0.007
Bacteria	Proteobacte	Unclassifie	OTU_513	0.072	0.048	0.004	0.004	0.007
Bacteria	Actinobacte	Gaiella	OTU_909	0.072	0.067	0.005	0.010	0.033
Bacteria	Proteobacte	Unclassifie	OTU_334	0.076	0.071	0.009	0.013	0.042
Bacteria	Acidobacte	Unclassifie	OTU_2086	0.076	0.054	0.009	0.008	0.013
Bacteria	Proteobacte	Unclassifie	OTU_589	0.097	0.054	0.032	0.032	0.018
Bacteria	Proteobacte	Unclassifie	OTU_242	0.126	0.033	0.062	0.028	0.002
Bacteria	Acidobacte	Gp6	OTU_270	0.066	0.065	0.003	0.004	0.035
Bacteria	Proteobacte	Unclassifie	OTU_236	0.076	0.063	0.014	0.009	0.035
Bacteria	Bacteroidet	Terrimonas	OTU_544	0.110	0.052	0.048	0.039	0.025
Bacteria	Acidobacte	Gp4	OTU_8027	0.061	0.044	0.000	0.000	0.007
Bacteria	Gemmatim	Gemmatim	OTU_260	0.066	0.061	0.004	0.006	0.032
Bacteria	Acidobacte	Gp6	OTU_6070	0.066	0.054	0.005	0.011	0.021
Bacteria	Actinobacte	Gaiella	OTU_382	0.069	0.054	0.009	0.009	0.022
Bacteria	Acidobacte	Gp4	OTU_2388	0.066	0.055	0.006	0.010	0.023
Bacteria	Acidobacte	Gp6	OTU_1906	0.077	0.022	0.017	0.017	0.000
Bacteria	Actinobacte	Conexibact	OTU_353	0.068	0.033	0.010	0.012	0.002
Bacteria	Actinobacte	Gaiella	OTU_335	0.073	0.031	0.015	0.011	0.001
Bacteria	Actinobacte	Gaiella	OTU_702	0.071	0.034	0.014	0.014	0.002
Bacteria	Actinobacte	Gaiella	OTU_8708	0.062	0.044	0.005	0.007	0.010
Bacteria	Proteobacte	Haliangium	OTU_3989	0.077	0.025	0.019	0.016	0.000
Bacteria	Actinobacte	Solirubroba	OTU_2102	0.060	0.047	0.003	0.004	0.015
Bacteria	Proteobacte	Minicystis	OTU_402	0.079	0.061	0.023	0.017	0.046
Bacteria	Actinobacte	Gaiella	OTU_366	0.060	0.021	0.005	0.006	0.000
Bacteria	Acidobacte	Gp6	OTU_7014	0.062	0.036	0.007	0.010	0.005
Bacteria	Candidatus	Saccharibac	OTU_882	0.054	0.052	0.000	0.000	0.027
Bacteria	Actinobacte	Dactylospo	OTU_141	0.099	0.044	0.045	0.021	0.014
Bacteria	Acidobacte	Gp16	OTU_496	0.099	0.032	0.045	0.023	0.003
Bacteria	Verrucomic	Opitutus	OTU_645	0.087	0.054	0.033	0.021	0.036
Bacteria	candidate d	WPS-1_gen	OTU_816	0.061	0.035	0.008	0.005	0.005
Bacteria	Proteobacte	Unclassifie	OTU_527	0.054	0.045	0.002	0.003	0.018
Bacteria	Actinobacte	Ilumatobac	OTU_501	0.054	0.047	0.003	0.003	0.022
Bacteria	Acidobacte	Gp4	OTU_1101	0.057	0.029	0.005	0.004	0.002
Bacteria	Acidobacte	Gp6	OTU_6956	0.079	0.047	0.027	0.033	0.035
Bacteria	Actinobacte	Gaiella	OTU_2538	0.051	0.048	0.000	0.000	0.025
Bacteria	Unclassifie	Unclassifie	OTU_658	0.058	0.037	0.007	0.008	0.008

Bacteria	Actinobact	Nocardioid	OTU_2656	0.063	0.040	0.012	0.011	0.012
Bacteria	Proteobacte	Skermanell	OTU_352	0.058	0.033	0.009	0.012	0.005
Bacteria	Chloroflexi	Unclassifie	OTU_1107	0.059	0.034	0.010	0.006	0.006
Bacteria	Armatimon	Armatimon	OTU_258	0.063	0.025	0.014	0.012	0.001
Bacteria	Actinobact	Pseudonoca	OTU_598	0.079	0.045	0.031	0.023	0.031
Bacteria	Proteobacte	Unclassifie	OTU_880	0.054	0.052	0.007	0.004	0.049
Bacteria	Gemmatim	Gemmatim	OTU_293	0.088	0.027	0.041	0.032	0.010
Bacteria	candidate d	WPS-1_ge	OTU_479	0.059	0.034	0.012	0.013	0.007
Bacteria	candidate d	WPS-1_ge	OTU_646	0.047	0.041	0.000	0.000	0.020
Bacteria	Acidobacte	Gp6	OTU_435	0.052	0.043	0.006	0.007	0.026
Bacteria	Actinobact	Unclassifie	OTU_1717	0.047	0.033	0.001	0.002	0.007
Bacteria	Actinobact	Conexibact	OTU_500	0.047	0.033	0.001	0.002	0.008
Bacteria	Bacteroidet	Phaeodacty	OTU_873	0.045	0.048	0.000	0.000	0.042
Bacteria	Actinobact	Actinophyt	OTU_4412	0.045	0.035	0.000	0.000	0.012
Bacteria	Actinobact	Unclassifie	OTU_3055	0.048	0.025	0.003	0.004	0.002
Bacteria	Acidobacte	Gp6	OTU_1025	0.054	0.038	0.009	0.012	0.017
Bacteria	Unclassifie	Unclassifie	OTU_403	0.045	0.033	0.002	0.003	0.011
Bacteria	Unclassifie	Unclassifie	OTU_956	0.047	0.042	0.004	0.004	0.029
Bacteria	Actinobact	Conexibact	OTU_358	0.054	0.018	0.011	0.009	0.000
Bacteria	Actinobact	Aquihabita	OTU_1418	0.057	0.031	0.014	0.012	0.008
Bacteria	Unclassifie	Unclassifie	OTU_590	0.046	0.044	0.003	0.006	0.039
Bacteria	Verrucomi	Subdivisio	OTU_493	0.050	0.025	0.008	0.007	0.003
Bacteria	Unclassifie	Unclassifie	OTU_1346	0.051	0.031	0.009	0.009	0.009
Bacteria	Proteobacte	Unclassifie	OTU_193	0.077	0.040	0.035	0.019	0.034
Bacteria	Proteobacte	Unclassifie	OTU_1096	0.049	0.026	0.007	0.009	0.004
Bacteria	Acidobacte	Gp4	OTU_340	0.059	0.038	0.018	0.029	0.041
Bacteria	Proteobacte	Lysobacter	OTU_814	0.043	0.034	0.002	0.002	0.015
Bacteria	Actinobact	Asanoa	OTU_6734	0.042	0.042	0.002	0.002	0.037
Bacteria	Actinobact	Nocardioid	OTU_3526	0.041	0.035	0.001	0.002	0.018
Bacteria	Proteobacte	Pseudorhoc	OTU_7191	0.082	0.023	0.041	0.016	0.003
Bacteria	Proteobacte	Unclassifie	OTU_7432	0.068	0.035	0.028	0.020	0.021
Bacteria	Unclassifie	Unclassifie	OTU_4079	0.046	0.037	0.006	0.010	0.023
Bacteria	Verrucomi	Spartobacte	OTU_614	0.042	0.039	0.002	0.003	0.030
Bacteria	candidate d	WPS-1_ge	OTU_591	0.049	0.023	0.009	0.008	0.002
Bacteria	Verrucomi	Unclassifie	OTU_800	0.045	0.036	0.005	0.005	0.022
Bacteria	Acidobacte	Gp5	OTU_526	0.058	0.039	0.019	0.022	0.039
Bacteria	Proteobacte	Unclassifie	OTU_414	0.051	0.027	0.011	0.014	0.006
Bacteria	Proteobacte	Unclassifie	OTU_822	0.044	0.038	0.004	0.006	0.030
Bacteria	Acidobacte	Gp10	OTU_561	0.039	0.034	0.000	0.000	0.020
Bacteria	Unclassifie	Unclassifie	OTU_449	0.046	0.031	0.008	0.009	0.014
Bacteria	Proteobacte	Pedomicrol	OTU_1126	0.042	0.025	0.004	0.008	0.005
Bacteria	Proteobacte	Unclassifie	OTU_1616	0.049	0.036	0.011	0.007	0.025
Bacteria	Acidobacte	Aridibacter	OTU_696	0.044	0.031	0.006	0.009	0.014
Bacteria	Actinobact	Unclassifie	OTU_391	0.043	0.015	0.005	0.003	0.000
Bacteria	Acidobacte	Gp6	OTU_345	0.049	0.030	0.011	0.012	0.013
Bacteria	Verrucomi	Subdivisio	OTU_268	0.072	0.032	0.035	0.027	0.033
Bacteria	Actinobact	Gaiella	OTU_755	0.057	0.025	0.019	0.011	0.005
Bacteria	Proteobacte	Unclassifie	OTU_1235	0.046	0.035	0.008	0.017	0.030
Bacteria	Proteobacte	Unclassifie	OTU_576	0.062	0.032	0.025	0.015	0.018
Bacteria	Latescibact	Latescibact	OTU_5436	0.046	0.033	0.009	0.011	0.021
Bacteria	candidate d	WPS-1_ge	OTU_716	0.042	0.028	0.005	0.007	0.011
Bacteria	Acidobacte	Gp6	OTU_6273	0.046	0.028	0.009	0.011	0.011
Bacteria	candidate d	WPS-1_ge	OTU_667	0.049	0.027	0.013	0.012	0.009
Bacteria	Proteobacte	Unclassifie	OTU_553	0.058	0.024	0.022	0.033	0.035
Bacteria	Actinobact	Aquihabita	OTU_1180	0.040	0.022	0.004	0.010	0.003
Bacteria	Proteobacte	Sphingomo	OTU_1805	0.039	0.036	0.003	0.003	0.034

Bacteria	Unclassifie	Unclassifie	OTU_1026	0.037	0.032	0.002	0.003	0.021
Bacteria	Proteobacte	Povalibacte	OTU_350	0.041	0.034	0.006	0.005	0.029
Bacteria	Actinobacte	Gaiella	OTU_1998	0.038	0.025	0.002	0.003	0.007
Bacteria	Armatimon	Armatimon	OTU_582	0.041	0.022	0.006	0.009	0.004
Bacteria	Planctomyc	Zavarzinell	OTU_718	0.043	0.034	0.009	0.013	0.033
Bacteria	candidate d	WPS-1_ger	OTU_326	0.050	0.028	0.015	0.014	0.014
Bacteria	Proteobacte	Unclassifie	OTU_921	0.034	0.021	0.000	0.000	0.004
Bacteria	Actinobacte	Unclassifie	OTU_8659	0.037	0.030	0.004	0.006	0.023
Bacteria	Proteobacte	Unclassifie	OTU_878	0.038	0.017	0.005	0.004	0.001
Bacteria	Gemmatim	Gemmatim	OTU_354	0.037	0.013	0.004	0.006	0.000
Bacteria	Proteobacte	Unclassifie	OTU_782	0.049	0.033	0.017	0.017	0.044
Bacteria	Unclassifie	Unclassifie	OTU_8085	0.032	0.027	0.000	0.000	0.016
Bacteria	Bacteroidet	Unclassifie	OTU_1185	0.035	0.032	0.003	0.007	0.034
Bacteria	Proteobacte	Unclassifie	OTU_280	0.036	0.020	0.004	0.005	0.004
Bacteria	Actinobacte	Unclassifie	OTU_848	0.031	0.034	0.000	0.000	0.044
Bacteria	Proteobacte	Unclassifie	OTU_385	0.038	0.028	0.007	0.005	0.021
Bacteria	Acidobacte	Gp3	OTU_1020	0.035	0.030	0.004	0.004	0.030
Bacteria	candidate d	WPS-1_ger	OTU_776	0.034	0.031	0.003	0.003	0.035
Bacteria	Actinobacte	Conexibact	OTU_4156	0.034	0.021	0.004	0.004	0.006
Bacteria	Acidobacte	Gp6	OTU_674	0.032	0.027	0.002	0.003	0.022
Bacteria	Acidobacte	Gp17	OTU_2055	0.032	0.024	0.002	0.004	0.013
Bacteria	Verrucomic	Subdivision	OTU_5426	0.041	0.008	0.012	0.013	0.000
Bacteria	Acidobacte	Gp6	OTU_3140	0.037	0.027	0.008	0.015	0.029
Bacteria	Proteobacte	Lysobacter	OTU_8277	0.032	0.031	0.003	0.003	0.042
Bacteria	Actinobacte	Aciditerrim	OTU_739	0.039	0.015	0.010	0.007	0.001
Bacteria	Chloroflexi	Unclassifie	OTU_1171	0.029	0.022	0.001	0.001	0.011
Bacteria	Chloroflexi	Unclassifie	OTU_568	0.032	0.021	0.004	0.006	0.010
Bacteria	Proteobacte	Cupriavidu	OTU_320	0.040	0.024	0.012	0.015	0.022
Bacteria	candidate d	WPS-1_ger	OTU_1535	0.028	0.018	0.000	0.000	0.005
Bacteria	Actinobacte	Thermoleop	OTU_1112	0.031	0.014	0.004	0.003	0.001
Bacteria	Unclassifie	Unclassifie	OTU_751	0.029	0.020	0.001	0.003	0.007
Bacteria	Acidobacte	Gp6	OTU_7800	0.029	0.020	0.002	0.003	0.008
Bacteria	Proteobacte	Andersenie	OTU_941	0.030	0.025	0.002	0.004	0.024
Bacteria	Gemmatim	Gemmatim	OTU_1104	0.031	0.018	0.003	0.005	0.004
Bacteria	Unclassifie	Unclassifie	OTU_7920	0.027	0.022	0.000	0.000	0.014
Bacteria	Gemmatim	Gemmatim	OTU_958	0.027	0.030	0.000	0.000	0.049
Bacteria	candidate d	WPS-1_ger	OTU_1919	0.029	0.025	0.003	0.003	0.025
Bacteria	Acidobacte	Gp6	OTU_5378	0.037	0.023	0.011	0.009	0.020
Bacteria	candidate d	WPS-1_ger	OTU_797	0.026	0.018	0.000	0.000	0.007
Bacteria	Actinobacte	Unclassifie	OTU_546	0.058	0.020	0.032	0.013	0.013
Bacteria	Planctomyc	Pirellula	OTU_1057	0.041	0.018	0.015	0.015	0.012
Bacteria	Actinobacte	Unclassifie	OTU_723	0.033	0.025	0.008	0.006	0.029
Bacteria	Verrucomic	Spartobacte	OTU_976	0.026	0.022	0.001	0.001	0.018
Bacteria	Proteobacte	Unclassifie	OTU_642	0.027	0.018	0.002	0.002	0.006
Bacteria	Cyanobacte	Bacillariop	OTU_719	0.030	0.027	0.004	0.006	0.043
Bacteria	Planctomyc	Pirellula	OTU_1711	0.036	0.012	0.011	0.013	0.002
Bacteria	Actinobacte	Nocardioid	OTU_2286	0.037	0.016	0.012	0.008	0.003
Bacteria	Actinobacte	Gaiella	OTU_935	0.037	0.012	0.012	0.012	0.001
Bacteria	Unclassifie	Unclassifie	OTU_1623	0.034	0.021	0.009	0.012	0.018
Bacteria	Acidobacte	Blastocatel	OTU_7207	0.025	0.027	0.001	0.001	0.047
Bacteria	Actinobacte	Unclassifie	OTU_518	0.032	0.016	0.007	0.009	0.005
Bacteria	Acidobacte	Gp6	OTU_4035	0.031	0.022	0.006	0.005	0.022
Bacteria	Actinobacte	Aquihabita	OTU_684	0.026	0.021	0.002	0.003	0.019
Bacteria	Unclassifie	Unclassifie	OTU_2969	0.024	0.019	0.000	0.001	0.013
Bacteria	Actinobacte	Gaiella	OTU_726	0.031	0.018	0.008	0.006	0.010
Bacteria	Proteobacte	Unclassifie	OTU_821	0.025	0.023	0.002	0.002	0.029

Bacteria	Acidobacte	Gp3	OTU_7700	0.034	0.021	0.010	0.020	0.046
Bacteria	Actinobact	Iamia	OTU_917	0.024	0.023	0.000	0.000	0.031
Bacteria	Actinobact	Gaiella	OTU_5633	0.025	0.024	0.001	0.002	0.033
Bacteria	Verrucomic	Subdivision	OTU_483	0.027	0.016	0.004	0.004	0.006
Bacteria	Actinobact	Conexibact	OTU_560	0.030	0.016	0.006	0.007	0.005
Bacteria	Acidobacte	Aridibacter	OTU_6837	0.029	0.023	0.005	0.004	0.029
Bacteria	candidate d	WPS-1_ge	OTU_8125	0.026	0.020	0.004	0.003	0.020
Bacteria	Acidobacte	Gp4	OTU_842	0.030	0.014	0.007	0.013	0.007
Bacteria	Acidobacte	Gp6	OTU_2420	0.029	0.006	0.007	0.009	0.000
Bacteria	Acidobacte	Gp7	OTU_904	0.023	0.022	0.000	0.000	0.028
Bacteria	Proteobacte	Nitrosospir	OTU_4299	0.027	0.016	0.004	0.006	0.007
Bacteria	Acidobacte	Candidatus	OTU_838	0.029	0.023	0.006	0.007	0.040
Bacteria	Acidobacte	Gp1	OTU_734	0.023	0.025	0.000	0.000	0.047
Bacteria	Acidobacte	Gp3	OTU_1017	0.027	0.024	0.005	0.004	0.045
Bacteria	Actinobact	Unclassifie	OTU_5308	0.022	0.020	0.000	0.000	0.020
Bacteria	Proteobacte	Unclassifie	OTU_1051	0.026	0.011	0.004	0.006	0.001
Bacteria	Acidobacte	Gp3	OTU_3754	0.023	0.016	0.001	0.002	0.009
Bacteria	candidate d	WPS-1_ge	OTU_6891	0.023	0.020	0.001	0.001	0.025
Bacteria	Proteobacte	Panacagrim	OTU_573	0.027	0.014	0.005	0.006	0.004
Bacteria	Proteobacte	Unclassifie	OTU_2100	0.022	0.015	0.001	0.001	0.006
Bacteria	Unclassifie	Unclassifie	OTU_1719	0.023	0.022	0.001	0.002	0.038
Bacteria	Planctomyc	Gemmata	OTU_899	0.043	0.013	0.021	0.012	0.007
Bacteria	Acidobacte	Gp7	OTU_1601	0.021	0.017	0.000	0.000	0.013
Bacteria	Bacteroidet	Niastella	OTU_1837	0.023	0.015	0.002	0.004	0.007
Bacteria	Verrucomic	Subdivision	OTU_1194	0.022	0.017	0.001	0.003	0.013
Bacteria	Actinobact	Thermoleo	OTU_5310	0.022	0.018	0.001	0.002	0.017
Bacteria	Acidobacte	Gp4	OTU_1424	0.026	0.019	0.005	0.012	0.032
Bacteria	Proteobacte	Povalibacte	OTU_1074	0.023	0.017	0.002	0.004	0.013
Bacteria	Actinobact	Conexibact	OTU_4812	0.025	0.015	0.004	0.004	0.007
Bacteria	Proteobacte	Bosea	OTU_383	0.043	0.021	0.022	0.013	0.045
Bacteria	Actinobact	Conexibact	OTU_1676	0.026	0.022	0.005	0.005	0.043
Bacteria	Proteobacte	Sphingomo	OTU_1414	0.037	0.017	0.017	0.017	0.044
Bacteria	Chloroflexi	Unclassifie	OTU_682	0.028	0.015	0.008	0.014	0.018
Bacteria	Actinobact	Unclassifie	OTU_6819	0.027	0.020	0.007	0.009	0.039
Bacteria	Proteobacte	Unclassifie	OTU_1155	0.020	0.019	0.000	0.000	0.028
Bacteria	Proteobacte	Unclassifie	OTU_1482	0.022	0.019	0.002	0.005	0.027
Bacteria	Unclassifie	Unclassifie	OTU_1369	0.023	0.019	0.004	0.008	0.032
Bacteria	Actinobact	Aciditerrim	OTU_356	0.037	0.018	0.017	0.008	0.027
Bacteria	Actinobact	Hamadaea	OTU_430	0.024	0.017	0.004	0.004	0.020
Bacteria	Bacteroidet	Unclassifie	OTU_8205	0.021	0.017	0.002	0.003	0.019
Bacteria	Acidobacte	Gp4	OTU_951	0.026	0.013	0.007	0.007	0.005
Bacteria	Proteobacte	Pseudolabr	OTU_7843	0.034	0.012	0.015	0.017	0.033
Bacteria	Actinobact	Microbacte	OTU_1047	0.030	0.015	0.012	0.008	0.014
Bacteria	Armatimon	Armatimon	OTU_788	0.020	0.013	0.001	0.002	0.007
Bacteria	Gemmatim	Gemmatim	OTU_412	0.022	0.012	0.004	0.003	0.003
Bacteria	Planctomyc	Gemmata	OTU_2315	0.022	0.015	0.004	0.003	0.013
Bacteria	Actinobact	Saccharopo	OTU_1198	0.020	0.013	0.002	0.004	0.006
Bacteria	Bacteroidet	Flavisoliba	OTU_7049	0.023	0.019	0.004	0.003	0.042
Bacteria	Actinobact	Unclassifie	OTU_845	0.023	0.014	0.005	0.002	0.012
Bacteria	Proteobacte	Labilithrix	OTU_604	0.025	0.017	0.007	0.004	0.025
Bacteria	candidate d	WPS-1_ge	OTU_995	0.020	0.019	0.002	0.004	0.041
Bacteria	Proteobacte	Unclassifie	OTU_1270	0.020	0.015	0.003	0.006	0.019
Bacteria	Actinobact	Gaiella	OTU_7601	0.020	0.014	0.003	0.002	0.013
Bacteria	Armatimon	Armatimon	OTU_870	0.022	0.017	0.004	0.004	0.027
Bacteria	Unclassifie	Unclassifie	OTU_1603	0.017	0.018	0.000	0.000	0.036
Bacteria	Unclassifie	Unclassifie	OTU_3201	0.020	0.017	0.003	0.005	0.033

Bacteria	candidate d	WPS-1_gei	OTU_1310	0.023	0.013	0.005	0.005	0.012
Bacteria	Proteobacte	Unclassifie	OTU_1827	0.022	0.012	0.005	0.007	0.009
Bacteria	Unclassifie	Unclassifie	OTU_970	0.018	0.017	0.001	0.003	0.040
Archaea	Thaumarch	Nitrososph	OTU_4268	0.017	0.016	0.000	0.000	0.029
Bacteria	Actinobact	Unclassifie	OTU_1599	0.018	0.013	0.001	0.001	0.014
Bacteria	Acidobacte	Gp6	OTU_4250	0.020	0.016	0.003	0.005	0.035
Bacteria	Unclassifie	Unclassifie	OTU_1050	0.016	0.015	0.000	0.000	0.022
Bacteria	Bacteroidet	Unclassifie	OTU_957	0.029	0.012	0.013	0.011	0.020
Bacteria	Proteobacte	Methyloph	OTU_511	0.020	0.015	0.004	0.004	0.024
Bacteria	Actinobact	Solirubroba	OTU_764	0.016	0.017	0.000	0.000	0.041
Bacteria	Proteobacte	Inquilinus	OTU_1480	0.019	0.017	0.003	0.004	0.046
Bacteria	Actinobact	Unclassifie	OTU_4640	0.018	0.012	0.002	0.004	0.011
Bacteria	Actinobact	Gaiella	OTU_1070	0.028	0.015	0.013	0.008	0.037
Bacteria	Bacteroidet	Flavisoliba	OTU_8472	0.021	0.017	0.005	0.005	0.045
Bacteria	Acidobacte	Gp25	OTU_1538	0.015	0.016	0.000	0.000	0.045
Bacteria	Armatimon	Armatimon	OTU_1123	0.029	0.014	0.014	0.008	0.031
Bacteria	candidate d	WPS-1_gei	OTU_920	0.018	0.009	0.003	0.003	0.002
Bacteria	Actinobact	Nocardioid	OTU_4115	0.017	0.009	0.003	0.003	0.003
Bacteria	Actinobact	Unclassifie	OTU_2442	0.018	0.014	0.003	0.002	0.029
Bacteria	Proteobacte	Unclassifie	OTU_1434	0.016	0.013	0.002	0.003	0.021
Bacteria	Proteobacte	Unclassifie	OTU_8769	0.015	0.013	0.000	0.001	0.024
Bacteria	Proteobacte	Unclassifie	OTU_1496	0.014	0.013	0.000	0.000	0.022
Bacteria	Gemmatim	Gemmatim	OTU_486	0.014	0.011	0.000	0.000	0.009
Bacteria	Verrucomi	Terrimicrol	OTU_2332	0.015	0.012	0.000	0.001	0.014
Bacteria	Acidobacte	Gp6	OTU_7880	0.019	0.014	0.004	0.006	0.034
Bacteria	Unclassifie	Unclassifie	OTU_2794	0.014	0.010	0.000	0.000	0.006
Bacteria	Acidobacte	Bryobacter	OTU_1316	0.016	0.010	0.002	0.004	0.007
Bacteria	candidate d	WPS-1_gei	OTU_1630	0.016	0.013	0.002	0.003	0.028
Bacteria	Unclassifie	Unclassifie	OTU_1281	0.015	0.014	0.001	0.002	0.031
Bacteria	Unclassifie	Unclassifie	OTU_874	0.015	0.012	0.001	0.001	0.020
Bacteria	Proteobacte	Unclassifie	OTU_3548	0.017	0.012	0.003	0.003	0.017
Bacteria	Bacteroidet	Unclassifie	OTU_890	0.018	0.015	0.004	0.004	0.050
Bacteria	Actinobact	Unclassifie	OTU_1062	0.014	0.013	0.000	0.000	0.029
Bacteria	Actinobact	Saccharoth	OTU_2471	0.016	0.007	0.003	0.004	0.001
Bacteria	Planctomyc	Pirellula	OTU_2971	0.015	0.011	0.001	0.002	0.015
Bacteria	Proteobacte	Unclassifie	OTU_5292	0.014	0.015	0.000	0.001	0.047
Bacteria	Unclassifie	Unclassifie	OTU_1557	0.013	0.013	0.000	0.000	0.028
Bacteria	Planctomyc	Pirellula	OTU_1492	0.017	0.010	0.004	0.006	0.014
Bacteria	Actinobact	Modestoba	OTU_2951	0.022	0.011	0.009	0.007	0.024
Bacteria	Unclassifie	Unclassifie	OTU_3622	0.013	0.013	0.000	0.000	0.029
Bacteria	Proteobacte	Unclassifie	OTU_1465	0.018	0.009	0.005	0.006	0.008
Bacteria	Proteobacte	Unclassifie	OTU_1721	0.013	0.008	0.000	0.000	0.003
Bacteria	Armatimon	Chthonomo	OTU_839	0.018	0.011	0.005	0.007	0.018
Bacteria	Proteobacte	Unclassifie	OTU_2431	0.016	0.014	0.003	0.004	0.043
Bacteria	Actinobact	Nocardioid	OTU_2483	0.013	0.008	0.000	0.000	0.004
Bacteria	candidate d	WPS-1_gei	OTU_1377	0.015	0.012	0.002	0.002	0.024
Bacteria	Acidobacte	Gp16	OTU_1249	0.016	0.012	0.003	0.003	0.028
Bacteria	Actinobact	Thermoleo	OTU_2841	0.014	0.008	0.001	0.001	0.004
Bacteria	Verrucomi	Subdivisio	OTU_3356	0.013	0.011	0.000	0.001	0.019
Bacteria	Chloroflexi	Unclassifie	OTU_2778	0.014	0.011	0.001	0.002	0.016
Bacteria	Bacteroidet	Flavisoliba	OTU_6302	0.015	0.011	0.003	0.004	0.018
Bacteria	Acidobacte	Gp6	OTU_6984	0.013	0.012	0.000	0.000	0.029
Bacteria	candidate d	WPS-1_gei	OTU_1965	0.014	0.012	0.001	0.002	0.026
Bacteria	Planctomyc	Tepidispha	OTU_4314	0.013	0.012	0.000	0.000	0.027
Bacteria	Planctomyc	Zavarzinell	OTU_2294	0.013	0.008	0.000	0.000	0.005
Bacteria	Proteobacte	Unclassifie	OTU_1883	0.013	0.010	0.000	0.000	0.011

Bacteria	Unclassifie	Unclassifie	OTU_1362	0.013	0.013	0.000	0.000	0.035
Bacteria	Actinobact	Unclassifie	OTU_2283	0.019	0.013	0.006	0.004	0.035
Bacteria	Actinobact	Crossiella	OTU_2371	0.013	0.008	0.000	0.001	0.004
Bacteria	Actinobact	Aquihabita	OTU_1283	0.016	0.012	0.004	0.004	0.026
Bacteria	Actinobact	Unclassifie	OTU_2744	0.014	0.007	0.002	0.003	0.002
Bacteria	Verrucomi	Subdivision	OTU_2272	0.015	0.009	0.003	0.003	0.009
Bacteria	Proteobact	Unclassifie	OTU_1064	0.016	0.013	0.004	0.004	0.043
Bacteria	Unclassifie	Unclassifie	OTU_1230	0.015	0.008	0.003	0.003	0.006
Bacteria	Planctomy	Singulispha	OTU_9305	0.013	0.010	0.001	0.002	0.018
Bacteria	Unclassifie	Unclassifie	OTU_1863	0.012	0.013	0.000	0.000	0.046
Bacteria	Latescibact	Latescibact	OTU_3355	0.012	0.013	0.000	0.000	0.046
Bacteria	Proteobact	Unclassifie	OTU_830	0.014	0.006	0.002	0.003	0.002
Bacteria	Unclassifie	Unclassifie	OTU_5626	0.011	0.010	0.000	0.000	0.017
Bacteria	Acidobacte	Unclassifie	OTU_1647	0.011	0.012	0.000	0.000	0.043
Bacteria	Planctomy	Unclassifie	OTU_1532	0.012	0.007	0.000	0.001	0.005
Bacteria	Unclassifie	Unclassifie	OTU_1606	0.016	0.009	0.005	0.003	0.009
Bacteria	Verrucomi	Subdivision	OTU_1517	0.012	0.013	0.001	0.001	0.048
Bacteria	Chloroflexi	Unclassifie	OTU_2769	0.011	0.009	0.000	0.000	0.011
Bacteria	Gemmatim	Gemmatim	OTU_1241	0.011	0.008	0.000	0.000	0.009
Bacteria	Actinobact	Unclassifie	OTU_1277	0.016	0.011	0.005	0.004	0.033
Bacteria	Bacteroidet	Unclassifie	OTU_810	0.011	0.012	0.000	0.000	0.039
Bacteria	Actinobact	Agromyces	OTU_485	0.011	0.012	0.000	0.000	0.045
Bacteria	candidate d	WPS-1_gei	OTU_7250	0.011	0.010	0.000	0.000	0.021
Bacteria	Proteobact	Bdellovibri	OTU_1115	0.013	0.011	0.002	0.003	0.035
Bacteria	Chloroflexi	Unclassifie	OTU_2208	0.011	0.009	0.000	0.000	0.021
Bacteria	Actinobact	Unclassifie	OTU_840	0.014	0.011	0.003	0.002	0.038
Bacteria	Proteobact	Unclassifie	OTU_2387	0.017	0.007	0.007	0.006	0.009
Bacteria	Acidobacte	Aridibacter	OTU_1641	0.011	0.011	0.001	0.002	0.046
Bacteria	Acidobacte	Gp16	OTU_2250	0.010	0.011	0.000	0.000	0.035
Bacteria	Proteobact	Unclassifie	OTU_7271	0.012	0.010	0.001	0.002	0.032
Bacteria	Actinobact	Unclassifie	OTU_3053	0.013	0.009	0.002	0.003	0.018
Bacteria	Unclassifie	Unclassifie	OTU_1292	0.018	0.009	0.008	0.006	0.029
Bacteria	Acidobacte	Blastocatel	OTU_2445	0.011	0.008	0.000	0.001	0.011
Bacteria	Latescibact	Latescibact	OTU_2755	0.010	0.010	0.000	0.000	0.029
Bacteria	Chloroflexi	Unclassifie	OTU_1044	0.010	0.010	0.000	0.000	0.028
Bacteria	Proteobact	Unclassifie	OTU_7407	0.017	0.007	0.006	0.008	0.020
Bacteria	Bacteroidet	Unclassifie	OTU_2603	0.011	0.010	0.001	0.001	0.036
Bacteria	Proteobact	Unclassifie	OTU_3039	0.010	0.007	0.000	0.000	0.007
Bacteria	Proteobact	Labilithrix	OTU_1193	0.020	0.009	0.009	0.008	0.046
Bacteria	Acidobacte	Gp25	OTU_256	0.010	0.011	0.000	0.000	0.046
Bacteria	candidate d	WPS-1_gei	OTU_2446	0.010	0.008	0.000	0.000	0.012
Bacteria	Bacteroidet	Chryseolin	OTU_2417	0.010	0.006	0.000	0.000	0.002
Bacteria	candidate d	WPS-1_gei	OTU_2873	0.012	0.008	0.002	0.005	0.019
Bacteria	Planctomy	Unclassifie	OTU_2541	0.012	0.010	0.002	0.003	0.031
Bacteria	Acidobacte	Gp6	OTU_355	0.010	0.008	0.000	0.000	0.017
Bacteria	Proteobact	Noviherbas	OTU_7351	0.015	0.006	0.005	0.006	0.009
Bacteria	Verrucomi	Spartobact	OTU_4809	0.010	0.010	0.000	0.000	0.033
Bacteria	Firmicutes	Unclassifie	OTU_2845	0.012	0.007	0.002	0.006	0.017
Bacteria	Acidobacte	Gp3	OTU_1798	0.012	0.008	0.002	0.004	0.018
Bacteria	Armatimon	Chthonom	OTU_1917	0.010	0.011	0.000	0.000	0.048
Bacteria	Actinobact	Cellulomor	OTU_1938	0.012	0.007	0.002	0.002	0.010
Bacteria	Proteobact	Geminicoc	OTU_2260	0.009	0.010	0.000	0.000	0.043
Bacteria	Planctomy	Blastopirel	OTU_1534	0.009	0.007	0.000	0.000	0.011
Bacteria	Actinobact	Conexibact	OTU_612	0.009	0.008	0.000	0.001	0.025
Bacteria	Actinobact	Conexibact	OTU_456	0.010	0.009	0.001	0.001	0.030
Bacteria	Proteobact	Unclassifie	OTU_2151	0.009	0.009	0.000	0.000	0.038

Bacteria	Bacteroidet	Unclassifie	OTU_4163	0.009	0.008	0.000	0.000	0.018
Bacteria	Unclassifie	Unclassifie	OTU_3596	0.009	0.010	0.000	0.000	0.042
Bacteria	Acidobacte	Gp11	OTU_1537	0.009	0.008	0.000	0.000	0.027
Bacteria	Cyanobacte	GpXIII	OTU_1504	0.009	0.010	0.000	0.000	0.045
Bacteria	Chloroflexi	Unclassifie	OTU_1560	0.011	0.009	0.002	0.003	0.030
Bacteria	Actinobact	Conexibact	OTU_1829	0.009	0.009	0.000	0.000	0.032
Bacteria	Unclassifie	Unclassifie	OTU_4392	0.009	0.007	0.000	0.000	0.015
Bacteria	Planctomyc	Pirellula	OTU_6664	0.009	0.006	0.001	0.001	0.005
Bacteria	Armatimon	Chthonomo	OTU_6933	0.016	0.004	0.008	0.008	0.024
Bacteria	Actinobact	Catelliglob	OTU_3483	0.009	0.006	0.000	0.000	0.009
Bacteria	Proteobacte	Stella	OTU_9383	0.010	0.007	0.002	0.002	0.014
Bacteria	Actinobact	Conexibact	OTU_5450	0.009	0.007	0.000	0.000	0.015
Bacteria	Armatimon	Armatimon	OTU_1509	0.009	0.008	0.000	0.000	0.025
Bacteria	Actinobact	Unclassifie	OTU_7038	0.010	0.008	0.002	0.002	0.032
Bacteria	Candidatus	Saccharibac	OTU_857	0.008	0.008	0.000	0.000	0.033
Bacteria	Chloroflexi	Unclassifie	OTU_1621	0.011	0.007	0.002	0.004	0.018
Bacteria	Unclassifie	Unclassifie	OTU_3064	0.008	0.005	0.000	0.000	0.003
Bacteria	Bacteroidet	Ferruginiba	OTU_1133	0.009	0.008	0.001	0.001	0.030
Bacteria	Verrucomic	Subdivision	OTU_9375	0.008	0.008	0.000	0.000	0.024
Bacteria	Planctomyc	Unclassifie	OTU_7512	0.010	0.009	0.002	0.004	0.047
Bacteria	Acidobacte	Gp16	OTU_1376	0.009	0.005	0.000	0.001	0.005
Bacteria	Unclassifie	Unclassifie	OTU_8947	0.008	0.008	0.000	0.000	0.036
Bacteria	Candidatus	Saccharibac	OTU_339	0.009	0.008	0.001	0.001	0.033
Bacteria	Planctomyc	Pirellula	OTU_1713	0.008	0.008	0.000	0.000	0.042
Bacteria	Armatimon	Chthonomo	OTU_2549	0.008	0.008	0.000	0.001	0.033
Bacteria	Acidobacte	Gp6	OTU_7338	0.008	0.006	0.000	0.000	0.009
Bacteria	Planctomyc	Planctopiru	OTU_2128	0.008	0.007	0.000	0.000	0.022
Bacteria	Proteobacte	Enhygromy	OTU_1851	0.009	0.007	0.001	0.002	0.026
Bacteria	Candidatus	Saccharibac	OTU_5805	0.009	0.007	0.001	0.001	0.023
Bacteria	Unclassifie	Unclassifie	OTU_6882	0.011	0.008	0.003	0.004	0.047
Bacteria	Proteobacte	Unclassifie	OTU_793	0.008	0.006	0.001	0.001	0.013
Bacteria	Proteobacte	Unclassifie	OTU_1728	0.008	0.004	0.000	0.001	0.001
Bacteria	Actinobact	Iamia	OTU_2518	0.008	0.003	0.000	0.001	0.000
Bacteria	Proteobacte	Unclassifie	OTU_1004	0.009	0.007	0.001	0.002	0.033
Bacteria	Unclassifie	Unclassifie	OTU_4819	0.008	0.008	0.000	0.000	0.038
Bacteria	Acidobacte	Gp3	OTU_901	0.007	0.006	0.000	0.000	0.015
Bacteria	Proteobacte	Unclassifie	OTU_2788	0.009	0.006	0.002	0.002	0.014
Bacteria	Proteobacte	Unclassifie	OTU_3392	0.007	0.005	0.000	0.000	0.007
Bacteria	Proteobacte	Unclassifie	OTU_3786	0.007	0.006	0.000	0.000	0.018
Bacteria	Acidobacte	Gp4	OTU_3425	0.007	0.007	0.000	0.000	0.031
Bacteria	Actinobact	Aciditerrim	OTU_2084	0.008	0.006	0.001	0.001	0.011
Bacteria	Unclassifie	Unclassifie	OTU_3933	0.007	0.007	0.000	0.000	0.035
Bacteria	candidate d	WPS-1_ger	OTU_9283	0.007	0.008	0.000	0.000	0.040
Bacteria	Unclassifie	Unclassifie	OTU_1615	0.007	0.004	0.000	0.000	0.003
Bacteria	Planctomyc	Gemmata	OTU_2039	0.008	0.006	0.001	0.002	0.011
Bacteria	Acidobacte	Gp6	OTU_3297	0.009	0.007	0.002	0.003	0.029
Bacteria	candidate d	WPS-1_ger	OTU_1429	0.008	0.007	0.001	0.002	0.034
Bacteria	Actinobact	Aciditerrim	OTU_1500	0.009	0.007	0.001	0.002	0.024
Bacteria	Proteobacte	Unclassifie	OTU_6747	0.007	0.007	0.000	0.000	0.025
Bacteria	Acidobacte	Gp10	OTU_1677	0.009	0.006	0.002	0.003	0.015
Bacteria	candidate d	WPS-1_ger	OTU_7605	0.007	0.006	0.000	0.000	0.013
Bacteria	Verrucomic	Subdivision	OTU_4051	0.008	0.006	0.001	0.002	0.021
Bacteria	Unclassifie	Unclassifie	OTU_9118	0.007	0.007	0.000	0.000	0.037
Bacteria	Planctomyc	Unclassifie	OTU_2312	0.007	0.004	0.000	0.000	0.001
Bacteria	Bacteroidet	Sediminiba	OTU_1794	0.009	0.007	0.002	0.003	0.047
Bacteria	Chloroflexi	Unclassifie	OTU_2635	0.007	0.007	0.000	0.000	0.043

Bacteria	Acidobacte	Gp6	OTU_2521	0.007	0.005	0.000	0.000	0.008
Bacteria	Gemmatim	Gemmatim	OTU_948	0.007	0.006	0.000	0.000	0.025
Bacteria	Unclassifie	Unclassifie	OTU_2003	0.008	0.006	0.001	0.002	0.020
Bacteria	Verrucomi	Spartobacte	OTU_2401	0.007	0.007	0.000	0.000	0.042
Bacteria	Proteobacte	Chelatococ	OTU_2012	0.006	0.006	0.000	0.000	0.029
Bacteria	Bacteroidet	Chryseolin	OTU_892	0.006	0.006	0.000	0.000	0.025
Bacteria	Proteobacte	Unclassifie	OTU_8293	0.006	0.004	0.000	0.000	0.005
Bacteria	Planctomy	Unclassifie	OTU_1478	0.006	0.005	0.000	0.000	0.014
Bacteria	Planctomy	Zavarzinell	OTU_5281	0.008	0.006	0.002	0.002	0.036
Bacteria	candidate d	WPS-1_ge	OTU_2548	0.006	0.006	0.000	0.000	0.032
Bacteria	Armatimon	Armatimon	OTU_3113	0.006	0.006	0.000	0.000	0.025
Bacteria	candidate d	WPS-1_ge	OTU_9312	0.006	0.005	0.000	0.000	0.017
Bacteria	Actinobact	Gaiella	OTU_7254	0.009	0.004	0.003	0.004	0.007
Bacteria	Unclassifie	Unclassifie	OTU_2080	0.006	0.006	0.000	0.000	0.037
Bacteria	Proteobacte	Unclassifie	OTU_2409	0.006	0.005	0.000	0.000	0.016
Bacteria	Acidobacte	Gp4	OTU_1494	0.007	0.003	0.001	0.002	0.001
Bacteria	Acidobacte	Gp6	OTU_4404	0.007	0.007	0.001	0.001	0.042
Bacteria	Actinobact	Gaiella	OTU_3444	0.006	0.006	0.000	0.000	0.023
Bacteria	Armatimon	Armatimon	OTU_4866	0.006	0.005	0.000	0.000	0.011
Bacteria	Actinobact	Unclassifie	OTU_4580	0.007	0.006	0.001	0.001	0.035
Bacteria	Proteobacte	Unclassifie	OTU_3076	0.006	0.006	0.000	0.000	0.033
Bacteria	Planctomy	Gemmata	OTU_6905	0.006	0.006	0.000	0.001	0.043
Bacteria	Actinobact	Ilumatobac	OTU_3819	0.007	0.006	0.001	0.001	0.039
Bacteria	Actinobact	Conexibact	OTU_2276	0.006	0.005	0.000	0.000	0.021
Bacteria	Actinobact	Nakamurel	OTU_9035	0.008	0.006	0.002	0.003	0.029
Bacteria	Actinobact	Sphaerispo	OTU_3720	0.006	0.005	0.000	0.000	0.024
Bacteria	Actinobact	Iamia	OTU_4097	0.007	0.006	0.001	0.001	0.034
Bacteria	Actinobact	Unclassifie	OTU_3396	0.006	0.005	0.000	0.000	0.019
Bacteria	Actinobact	Iamia	OTU_1659	0.007	0.006	0.001	0.002	0.048
Bacteria	Planctomy	Zavarzinell	OTU_2846	0.008	0.006	0.002	0.004	0.045
Bacteria	Armatimon	Armatimon	OTU_1896	0.006	0.004	0.000	0.000	0.011
Bacteria	Acidobacte	Gp17	OTU_8421	0.006	0.003	0.000	0.000	0.002
Bacteria	Planctomy	Unclassifie	OTU_2584	0.006	0.006	0.000	0.000	0.036
Bacteria	candidate d	WPS-1_ge	OTU_6717	0.006	0.006	0.000	0.000	0.045
Bacteria	Planctomy	Unclassifie	OTU_2244	0.009	0.006	0.004	0.004	0.044
Bacteria	Planctomy	Unclassifie	OTU_3856	0.006	0.005	0.000	0.000	0.017
Bacteria	Proteobacte	Unclassifie	OTU_1459	0.006	0.005	0.000	0.000	0.029
Bacteria	Acidobacte	Blastocatel	OTU_1741	0.007	0.005	0.002	0.002	0.025
Bacteria	Planctomy	Zavarzinell	OTU_5824	0.006	0.005	0.000	0.000	0.014
Bacteria	Acidobacte	Gp16	OTU_2911	0.007	0.006	0.001	0.002	0.038
Bacteria	Chloroflexi	Unclassifie	OTU_3749	0.006	0.005	0.000	0.000	0.026
Bacteria	Planctomy	Singulisph	OTU_2727	0.006	0.004	0.000	0.000	0.014
Bacteria	Planctomy	Unclassifie	OTU_3806	0.006	0.004	0.000	0.000	0.012
Bacteria	Verrucomi	Spartobacte	OTU_2866	0.006	0.006	0.000	0.000	0.033
Bacteria	Planctomy	Isosphaera	OTU_2246	0.007	0.005	0.002	0.003	0.035
Bacteria	Planctomy	Singulisph	OTU_3706	0.007	0.006	0.002	0.002	0.049
Bacteria	Gemmatim	Gemmatim	OTU_1347	0.006	0.006	0.001	0.001	0.039
Bacteria	Latescibact	Latescibact	OTU_4546	0.005	0.005	0.000	0.000	0.019
Bacteria	Unclassifie	Unclassifie	OTU_2504	0.005	0.004	0.000	0.000	0.008
Bacteria	Bacteroidet	Unclassifie	OTU_2087	0.005	0.006	0.000	0.000	0.044
Bacteria	Planctomy	Tepidispha	OTU_5781	0.005	0.006	0.000	0.000	0.042
Bacteria	Acidobacte	Gp6	OTU_3945	0.005	0.005	0.000	0.000	0.032
Bacteria	Acidobacte	Gp6	OTU_3325	0.005	0.005	0.000	0.000	0.035
Bacteria	Verrucomi	Subdivisio	OTU_3939	0.006	0.005	0.001	0.002	0.033
Bacteria	Planctomy	Planctomic	OTU_3460	0.005	0.003	0.000	0.000	0.002
Bacteria	Unclassifie	Unclassifie	OTU_7035	0.005	0.004	0.000	0.000	0.013

Bacteria	Actinobact	Unclassifie	OTU_2166	0.006	0.003	0.000	0.001	0.002
Bacteria	Acidobacte	Gp16	OTU_2624	0.005	0.005	0.000	0.000	0.029
Bacteria	Actinobact	Gaiella	OTU_6926	0.007	0.005	0.002	0.003	0.040
Bacteria	Candidatus	Saccharibac	OTU_1958	0.006	0.005	0.001	0.003	0.028
Bacteria	Chloroflexi	Unclassifie	OTU_6189	0.005	0.005	0.000	0.000	0.021
Bacteria	Proteobacte	Unclassifie	OTU_1836	0.006	0.005	0.001	0.001	0.031
Bacteria	Proteobacte	Unclassifie	OTU_1320	0.005	0.005	0.000	0.000	0.031
Bacteria	Unclassifie	Unclassifie	OTU_5952	0.005	0.005	0.000	0.000	0.037
Bacteria	Verrucomic	Unclassifie	OTU_2451	0.007	0.005	0.002	0.003	0.043
Bacteria	Candidatus	Saccharibac	OTU_7481	0.005	0.006	0.000	0.000	0.049
Bacteria	Actinobact	Gaiella	OTU_5514	0.006	0.006	0.001	0.001	0.049
Bacteria	Proteobacte	Unclassifie	OTU_3371	0.007	0.005	0.002	0.002	0.035
Bacteria	Verrucomic	Unclassifie	OTU_4063	0.005	0.006	0.000	0.000	0.050
Bacteria	Unclassifie	Unclassifie	OTU_3601	0.005	0.005	0.000	0.000	0.031
Bacteria	Unclassifie	Unclassifie	OTU_1318	0.005	0.005	0.000	0.000	0.027
Bacteria	Actinobact	Gaiella	OTU_6209	0.006	0.003	0.001	0.002	0.003
Bacteria	Proteobacte	Haliangium	OTU_3125	0.005	0.005	0.000	0.000	0.040
Bacteria	Acidobacte	Gp6	OTU_5353	0.005	0.005	0.000	0.000	0.028
Bacteria	Armatimon	Armatimon	OTU_1951	0.007	0.004	0.002	0.002	0.025
Bacteria	Proteobacte	Unclassifie	OTU_1881	0.006	0.005	0.001	0.001	0.044
Bacteria	Proteobacte	Unclassifie	OTU_4636	0.005	0.005	0.000	0.000	0.028
Bacteria	Bacteroidet	Ferruginiba	OTU_4321	0.006	0.005	0.001	0.002	0.040
Bacteria	Actinobact	Unclassifie	OTU_3321	0.005	0.003	0.000	0.000	0.007
Bacteria	Verrucomic	Unclassifie	OTU_3454	0.005	0.004	0.000	0.000	0.023
Bacteria	Proteobacte	Haliangium	OTU_3091	0.005	0.004	0.000	0.000	0.017
Bacteria	candidate d	WPS-1_ger	OTU_4134	0.005	0.005	0.000	0.000	0.031
Bacteria	Proteobacte	Unclassifie	OTU_6843	0.005	0.005	0.000	0.000	0.034
Bacteria	Chloroflexi	Ornatilinea	OTU_3850	0.005	0.004	0.000	0.000	0.029
Bacteria	Actinobact	Solirubroba	OTU_7926	0.005	0.004	0.000	0.000	0.014
Bacteria	Gemmatim	Gemmatim	OTU_5098	0.004	0.004	0.000	0.000	0.030
Bacteria	Planctomyc	Pirellula	OTU_3432	0.005	0.004	0.000	0.001	0.026
Bacteria	Proteobacte	Unclassifie	OTU_6521	0.005	0.005	0.001	0.001	0.050
Bacteria	Planctomyc	Unclassifie	OTU_7472	0.004	0.005	0.000	0.000	0.043
Bacteria	Actinobact	Unclassifie	OTU_4060	0.005	0.005	0.000	0.001	0.046
Bacteria	Acidobacte	Gp10	OTU_4730	0.004	0.004	0.000	0.000	0.031
Bacteria	Bacteroidet	Terrimonas	OTU_6910	0.004	0.005	0.000	0.000	0.042
Bacteria	Acidobacte	Gp5	OTU_2586	0.004	0.004	0.000	0.000	0.021
Bacteria	Proteobacte	Unclassifie	OTU_3422	0.004	0.004	0.000	0.000	0.023
Bacteria	Proteobacte	Unclassifie	OTU_1375	0.004	0.004	0.000	0.000	0.041
Bacteria	Planctomyc	Zavarzinell	OTU_5643	0.004	0.005	0.000	0.000	0.049
Bacteria	Unclassifie	Unclassifie	OTU_4293	0.004	0.004	0.000	0.000	0.029
Bacteria	candidate d	WPS-1_ger	OTU_4611	0.004	0.003	0.000	0.000	0.013
Bacteria	Actinobact	Unclassifie	OTU_5208	0.004	0.004	0.000	0.000	0.024
Bacteria	Verrucomic	Unclassifie	OTU_1366	0.005	0.004	0.001	0.002	0.037
Bacteria	candidate d	WPS-1_ger	OTU_3121	0.004	0.004	0.000	0.000	0.026
Bacteria	Unclassifie	Unclassifie	OTU_4642	0.004	0.004	0.000	0.000	0.048
Bacteria	Proteobacte	Unclassifie	OTU_3405	0.004	0.004	0.000	0.000	0.045
Bacteria	Proteobacte	Unclassifie	OTU_4898	0.004	0.004	0.000	0.000	0.020
Bacteria	Proteobacte	Labilithrix	OTU_5862	0.004	0.004	0.000	0.001	0.048
Bacteria	Proteobacte	Paracoccus	OTU_1699	0.005	0.004	0.001	0.002	0.025
Bacteria	Unclassifie	Unclassifie	OTU_4902	0.004	0.004	0.000	0.000	0.047
Bacteria	Bacteroidet	Unclassifie	OTU_7622	0.004	0.004	0.000	0.000	0.038
Bacteria	Chloroflexi	Unclassifie	OTU_4666	0.004	0.004	0.000	0.000	0.040
Bacteria	Unclassifie	Unclassifie	OTU_4753	0.004	0.004	0.000	0.000	0.036
Bacteria	Armatimon	Chthonomo	OTU_2108	0.004	0.004	0.000	0.000	0.035
Bacteria	Planctomyc	Gemmata	OTU_6831	0.004	0.003	0.000	0.000	0.023

Bacteria	Actinobact	Ilumatobac	OTU_7858	0.004	0.004	0.000	0.000	0.030
Bacteria	Actinobact	Unclassifie	OTU_2685	0.004	0.004	0.000	0.000	0.034
Bacteria	Acidobacte	Gp25	OTU_2232	0.004	0.003	0.000	0.000	0.019
Bacteria	Gemmatim	Gemmatim	OTU_7368	0.004	0.003	0.000	0.000	0.017
Bacteria	Acidobacte	Gp6	OTU_7291	0.004	0.004	0.000	0.000	0.045
Bacteria	Armatimon	Armatimon	OTU_2405	0.004	0.004	0.000	0.000	0.042
Bacteria	Armatimon	Armatimon	OTU_3382	0.005	0.004	0.001	0.002	0.047
Bacteria	Unclassifie	Unclassifie	OTU_4196	0.004	0.004	0.000	0.000	0.030
Bacteria	Chloroflexi	Litorilinea	OTU_5391	0.004	0.003	0.000	0.000	0.021
Bacteria	Planctomyc	Unclassifie	OTU_3697	0.004	0.003	0.000	0.000	0.019
Bacteria	Planctomyc	Thermogut	OTU_4149	0.004	0.004	0.000	0.000	0.038
Bacteria	Proteobacte	Unclassifie	OTU_2820	0.004	0.004	0.000	0.000	0.047
Bacteria	Actinobact	Marmorico	OTU_9340	0.004	0.003	0.001	0.002	0.034
Bacteria	Acidobacte	Gp4	OTU_5736	0.004	0.004	0.000	0.000	0.050
Bacteria	Bacteroidet	Ohtaekwan	OTU_1120	0.003	0.002	0.000	0.000	0.002
Bacteria	Actinobact	Unclassifie	OTU_7326	0.003	0.002	0.000	0.000	0.004
Bacteria	Acidobacte	Gp1	OTU_2070	0.003	0.004	0.000	0.000	0.043
Bacteria	Unclassifie	Unclassifie	OTU_7617	0.003	0.003	0.000	0.000	0.029
Bacteria	Unclassifie	Unclassifie	OTU_2284	0.003	0.003	0.000	0.000	0.029
Bacteria	Armatimon	Chthonomo	OTU_2731	0.003	0.002	0.000	0.000	0.005
Bacteria	Acidobacte	Gp6	OTU_2495	0.003	0.003	0.000	0.000	0.036
Bacteria	Proteobacte	Solimonas	OTU_2123	0.003	0.004	0.000	0.000	0.043
Bacteria	Proteobacte	Unclassifie	OTU_6078	0.003	0.004	0.000	0.000	0.047
Bacteria	Verrucomic	Luteolibact	OTU_3787	0.003	0.003	0.000	0.000	0.035
Bacteria	Proteobacte	Unclassifie	OTU_2340	0.003	0.002	0.000	0.000	0.008
Bacteria	Proteobacte	Unclassifie	OTU_3281	0.003	0.003	0.000	0.000	0.012
Bacteria	Verrucomic	Unclassifie	OTU_2869	0.003	0.003	0.000	0.000	0.038
Bacteria	Armatimon	Chthonomo	OTU_4176	0.003	0.003	0.000	0.000	0.038
Bacteria	Planctomyc	Unclassifie	OTU_3721	0.003	0.003	0.000	0.000	0.019
Bacteria	Acidobacte	Gp6	OTU_5299	0.003	0.003	0.000	0.000	0.012
Bacteria	Acidobacte	Bryobacter	OTU_1053	0.003	0.003	0.000	0.000	0.026
Bacteria	Planctomyc	Zavarzinell	OTU_6146	0.003	0.004	0.000	0.000	0.046
Bacteria	Unclassifie	Unclassifie	OTU_2980	0.003	0.003	0.000	0.000	0.043
Bacteria	Planctomyc	Candidatus	OTU_4721	0.003	0.004	0.000	0.000	0.048
Bacteria	Proteobacte	Sandaracin	OTU_6792	0.003	0.003	0.000	0.000	0.025
Bacteria	Acidobacte	Unclassifie	OTU_1831	0.003	0.003	0.000	0.001	0.024
Bacteria	Acidobacte	Gp6	OTU_1157	0.003	0.003	0.000	0.000	0.017
Bacteria	Proteobacte	Azonexus	OTU_2893	0.003	0.003	0.000	0.000	0.041
Bacteria	Proteobacte	Peredibacte	OTU_2320	0.003	0.003	0.000	0.000	0.014
Bacteria	Bacteroidet	Unclassifie	OTU_4673	0.003	0.003	0.000	0.000	0.048
Bacteria	Chloroflexi	Unclassifie	OTU_2173	0.005	0.003	0.002	0.002	0.039
Bacteria	Unclassifie	Unclassifie	OTU_1547	0.003	0.003	0.000	0.000	0.015
Bacteria	Planctomyc	Singulispha	OTU_3667	0.003	0.003	0.000	0.000	0.021
Bacteria	Planctomyc	Unclassifie	OTU_2204	0.003	0.003	0.000	0.000	0.041
Bacteria	Bacteroidet	Unclassifie	OTU_1842	0.003	0.003	0.000	0.000	0.037
Bacteria	Proteobacte	Unclassifie	OTU_3921	0.003	0.003	0.000	0.000	0.035
Bacteria	Chloroflexi	Unclassifie	OTU_5913	0.003	0.003	0.000	0.000	0.036
Bacteria	Proteobacte	Unclassifie	OTU_1182	0.003	0.003	0.000	0.000	0.038
Bacteria	Verrucomic	Subdivision	OTU_4741	0.003	0.003	0.000	0.000	0.042
Bacteria	Bacteroidet	Unclassifie	OTU_6943	0.003	0.003	0.000	0.000	0.036
Bacteria	Unclassifie	Unclassifie	OTU_1948	0.003	0.002	0.000	0.000	0.015
Bacteria	Verrucomic	Unclassifie	OTU_9266	0.003	0.003	0.000	0.000	0.021
Bacteria	Actinobact	Aciditerrim	OTU_4042	0.003	0.003	0.000	0.000	0.037
Bacteria	Bacteroidet	Solitalea	OTU_6017	0.003	0.003	0.000	0.000	0.043
Bacteria	Acidobacte	Gp6	OTU_6021	0.003	0.003	0.000	0.000	0.045
Bacteria	Actinobact	Iamia	OTU_4186	0.003	0.003	0.000	0.000	0.048

Bacteria	Planctomyc	Zavarzinell	OTU_2449	0.003	0.003	0.000	0.000	0.025
Bacteria	Acidobacte	Gp4	OTU_2370	0.003	0.003	0.000	0.000	0.033
Bacteria	Unclassifie	Unclassifie	OTU_6450	0.003	0.003	0.000	0.000	0.042
Bacteria	Chloroflexi	Unclassifie	OTU_2094	0.003	0.002	0.000	0.000	0.019
Bacteria	Proteobacte	Unclassifie	OTU_8374	0.003	0.003	0.000	0.000	0.041
Bacteria	Unclassifie	Unclassifie	OTU_3481	0.003	0.003	0.000	0.000	0.041
Bacteria	Actinobact	Aciditerrim	OTU_3900	0.003	0.003	0.000	0.000	0.032
Bacteria	Proteobacte	Labilithrix	OTU_5073	0.003	0.003	0.000	0.000	0.043
Bacteria	Proteobacte	Unclassifie	OTU_3470	0.003	0.002	0.000	0.000	0.032
Bacteria	Unclassifie	Unclassifie	OTU_3566	0.002	0.002	0.000	0.000	0.024
Bacteria	Actinobact	Unclassifie	OTU_6102	0.002	0.003	0.000	0.000	0.047
Bacteria	Armatimon	Fimbriimor	OTU_3750	0.002	0.003	0.000	0.000	0.039
Bacteria	Unclassifie	Unclassifie	OTU_5014	0.002	0.002	0.000	0.000	0.024
Bacteria	Planctomyc	Unclassifie	OTU_5593	0.002	0.003	0.000	0.000	0.038
Bacteria	Proteobacte	Unclassifie	OTU_4547	0.002	0.003	0.000	0.000	0.046
Bacteria	Proteobacte	Unclassifie	OTU_5971	0.002	0.002	0.000	0.000	0.036
Bacteria	Chloroflexi	Oscillochlo	OTU_2519	0.002	0.003	0.000	0.000	0.049
Bacteria	Acidobacte	Gp16	OTU_5152	0.002	0.002	0.000	0.000	0.024
Bacteria	Armatimon	Armatimon	OTU_6140	0.002	0.002	0.000	0.000	0.028
Bacteria	Chloroflexi	Unclassifie	OTU_2403	0.002	0.003	0.000	0.000	0.049
Bacteria	Unclassifie	Unclassifie	OTU_8341	0.002	0.003	0.000	0.000	0.044
Bacteria	Proteobacte	Unclassifie	OTU_8681	0.002	0.003	0.000	0.000	0.047
Bacteria	Unclassifie	Unclassifie	OTU_3300	0.002	0.002	0.000	0.000	0.036
Bacteria	Proteobacte	Hephaestia	OTU_6054	0.002	0.002	0.000	0.000	0.050
Bacteria	Proteobacte	Unclassifie	OTU_7728	0.002	0.002	0.000	0.000	0.015
Bacteria	Unclassifie	Unclassifie	OTU_3979	0.002	0.002	0.000	0.000	0.050
Bacteria	Planctomyc	Zavarzinell	OTU_4633	0.002	0.002	0.000	0.000	0.043
Bacteria	Latescibact	Latescibact	OTU_6469	0.002	0.002	0.000	0.000	0.029
Bacteria	Unclassifie	Unclassifie	OTU_3417	0.002	0.002	0.000	0.000	0.048
Bacteria	Verrucomic	Subdivision	OTU_5519	0.002	0.002	0.000	0.000	0.033
Bacteria	Proteobacte	Unclassifie	OTU_3581	0.002	0.002	0.000	0.000	0.037
Bacteria	Verrucomic	Spartobacte	OTU_6381	0.002	0.002	0.000	0.000	0.044
Bacteria	Chloroflexi	Litorilinea	OTU_4531	0.002	0.002	0.000	0.000	0.049
Bacteria	Unclassifie	Unclassifie	OTU_3164	0.002	0.002	0.000	0.000	0.043
Bacteria	Verrucomic	Subdivision	OTU_1714	0.002	0.002	0.000	0.000	0.039
Bacteria	Verrucomic	Unclassifie	OTU_6230	0.002	0.002	0.000	0.000	0.039
Bacteria	Gemmatim	Gemmatim	OTU_2314	0.002	0.002	0.000	0.000	0.039
Bacteria	Verrucomic	Subdivision	OTU_7595	0.002	0.002	0.000	0.000	0.039
Bacteria	Planctomyc	Unclassifie	OTU_7054	0.002	0.002	0.000	0.000	0.024
Bacteria	Verrucomic	Subdivision	OTU_9149	0.002	0.002	0.000	0.000	0.024
Bacteria	Proteobacte	Unclassifie	OTU_4290	0.002	0.002	0.000	0.000	0.024
Bacteria	Unclassifie	Unclassifie	OTU_3239	0.002	0.002	0.000	0.000	0.045
Bacteria	Verrucomic	Subdivision	OTU_3046	0.000	0.000	0.002	0.002	0.043
Bacteria	Proteobacte	Unclassifie	OTU_1192	0.000	0.000	0.002	0.002	0.049
Bacteria	Proteobacte	Unclassifie	OTU_1127	0.000	0.000	0.002	0.002	0.049
Bacteria	Unclassifie	Unclassifie	OTU_3089	0.000	0.000	0.002	0.002	0.037
Bacteria	Actinobact	Amycolato	OTU_1297	0.000	0.000	0.002	0.002	0.024
Bacteria	Firmicutes	Desulfospo	OTU_5140	0.000	0.000	0.002	0.002	0.038
Bacteria	Proteobacte	Unclassifie	OTU_4860	0.000	0.000	0.002	0.002	0.038
Bacteria	Proteobacte	Geobacter	OTU_2372	0.000	0.000	0.002	0.002	0.038
Bacteria	Proteobacte	Unclassifie	OTU_2455	0.000	0.000	0.002	0.003	0.044
Bacteria	Unclassifie	Unclassifie	OTU_5086	0.000	0.000	0.002	0.002	0.034
Bacteria	candidate d	WPS-2_ge	OTU_7442	0.000	0.000	0.003	0.003	0.039
Bacteria	Acidobacte	Edaphobac	OTU_5251	0.000	0.000	0.003	0.002	0.009
Bacteria	Unclassifie	Unclassifie	OTU_4184	0.000	0.000	0.003	0.003	0.042
Bacteria	Proteobacte	Unclassifie	OTU_1134	0.000	0.000	0.003	0.003	0.048

Bacteria	Proteobacte	Unclassifie	OTU_3630	0.000	0.000	0.003	0.003	0.036
Bacteria	Proteobacte	Alkanibact	OTU_4596	0.000	0.000	0.003	0.003	0.033
Bacteria	Unclassifie	Unclassifie	OTU_3557	0.000	0.000	0.003	0.004	0.048
Bacteria	Proteobacte	Unclassifie	OTU_9226	0.000	0.000	0.003	0.004	0.049
Bacteria	Unclassifie	Unclassifie	OTU_5828	0.000	0.000	0.003	0.002	0.007
Bacteria	Armatimon	Armatimon	OTU_1395	0.000	0.000	0.003	0.003	0.042
Bacteria	Verrucomi	Subdivisio	OTU_3431	0.000	0.000	0.003	0.004	0.044
Bacteria	Proteobacte	Unclassifie	OTU_5232	0.000	0.000	0.003	0.003	0.020
Archaea	Crenarchae	Unclassifie	OTU_1648	0.000	0.000	0.003	0.003	0.023
Bacteria	candidate d	WPS-1_ge	OTU_6434	0.000	0.000	0.003	0.004	0.047
Bacteria	Proteobacte	Unclassifie	OTU_3021	0.000	0.000	0.004	0.003	0.017
Archaea	Euryarchae	Unclassifie	OTU_1174	0.000	0.000	0.004	0.003	0.023
Bacteria	Planctomyc	Unclassifie	OTU_3897	0.000	0.000	0.004	0.004	0.029
Bacteria	Proteobacte	Unclassifie	OTU_2691	0.001	0.002	0.005	0.004	0.033
Bacteria	Firmicutes	Clostridiun	OTU_7821	0.000	0.000	0.004	0.003	0.012
Bacteria	Gemmatim	Gemmatim	OTU_2326	0.000	0.000	0.004	0.004	0.022
Bacteria	Unclassifie	Unclassifie	OTU_3087	0.000	0.000	0.004	0.004	0.025
Bacteria	Candidatus	Sacchariba	OTU_4005	0.000	0.000	0.004	0.005	0.048
Bacteria	Firmicutes	Unclassifie	OTU_1995	0.000	0.000	0.004	0.004	0.040
Bacteria	Unclassifie	Unclassifie	OTU_3172	0.000	0.000	0.004	0.004	0.040
Bacteria	Unclassifie	Unclassifie	OTU_3292	0.000	0.000	0.004	0.005	0.045
Bacteria	Proteobacte	Asticcacau	OTU_1360	0.000	0.000	0.004	0.004	0.031
Bacteria	Verrucomi	Subdivisio	OTU_5912	0.000	0.000	0.004	0.004	0.031
Bacteria	Chloroflexi	Unclassifie	OTU_3148	0.000	0.000	0.004	0.005	0.045
Bacteria	Acidobacte	Candidatus	OTU_629	0.000	0.000	0.004	0.005	0.045
Bacteria	Planctomyc	Zavarzinell	OTU_3170	0.000	0.000	0.005	0.003	0.005
Bacteria	candidate d	WPS-1_ge	OTU_1838	0.001	0.002	0.006	0.005	0.033
Bacteria	Proteobacte	Brenneria	OTU_4465	0.000	0.000	0.005	0.005	0.043
Bacteria	Planctomyc	Aquisphae	OTU_2139	0.001	0.002	0.006	0.005	0.021
Bacteria	Unclassifie	Unclassifie	OTU_2342	0.000	0.000	0.006	0.006	0.047
Bacteria	Firmicutes	Clostridiun	OTU_3477	0.000	0.000	0.006	0.006	0.044
Bacteria	Acidobacte	Gp18	OTU_5033	0.000	0.000	0.006	0.006	0.033
Bacteria	Proteobacte	Acidisoma	OTU_1325	0.000	0.000	0.006	0.006	0.037
Bacteria	Firmicutes	Clostridiun	OTU_2716	0.000	0.000	0.006	0.006	0.029
Bacteria	Armatimon	Chthonomo	OTU_1167	0.000	0.000	0.006	0.006	0.020
Bacteria	Proteobacte	Brevundim	OTU_690	0.000	0.000	0.006	0.007	0.045
Bacteria	Chloroflexi	Ktedonoba	OTU_2364	0.000	0.000	0.006	0.007	0.041
Bacteria	Bacteroidet	Unclassifie	OTU_1234	0.000	0.000	0.007	0.005	0.014
Bacteria	Proteobacte	Unclassifie	OTU_1181	0.001	0.002	0.008	0.007	0.043
Bacteria	Proteobacte	Unclassifie	OTU_2807	0.000	0.000	0.007	0.007	0.043
Bacteria	Firmicutes	Clostridiun	OTU_2821	0.000	0.000	0.007	0.006	0.018
Bacteria	Proteobacte	Unclassifie	OTU_1595	0.000	0.000	0.007	0.006	0.027
Bacteria	Unclassifie	Unclassifie	OTU_3044	0.000	0.000	0.007	0.007	0.040
Bacteria	Unclassifie	Unclassifie	OTU_1542	0.002	0.004	0.009	0.006	0.032
Bacteria	Bacteroidet	Lacibacter	OTU_1558	0.000	0.000	0.007	0.006	0.020
Bacteria	candidate d	WPS-2_ge	OTU_1523	0.000	0.000	0.007	0.006	0.019
Bacteria	Verrucomi	Subdivisio	OTU_7076	0.000	0.000	0.007	0.006	0.023
Bacteria	Unclassifie	Unclassifie	OTU_3202	0.000	0.000	0.007	0.008	0.047
Bacteria	Planctomyc	Unclassifie	OTU_1065	0.001	0.002	0.008	0.006	0.016
Bacteria	Gemmatim	Gemmatim	OTU_3187	0.002	0.004	0.009	0.007	0.040
Bacteria	candidate d	WPS-2_ge	OTU_421	0.003	0.005	0.011	0.008	0.048
Bacteria	Candidatus	Sacchariba	OTU_6023	0.000	0.000	0.007	0.007	0.021
Bacteria	Firmicutes	Clostridiun	OTU_4020	0.000	0.000	0.008	0.006	0.012
Bacteria	Bacteroidet	Unclassifie	OTU_1324	0.000	0.000	0.008	0.008	0.034
Bacteria	Proteobacte	Unclassifie	OTU_2199	0.000	0.000	0.008	0.006	0.013
Bacteria	Unclassifie	Unclassifie	OTU_2168	0.000	0.000	0.008	0.008	0.028

Bacteria	Firmicutes	Unclassifie	OTU_3035	0.000	0.000	0.008	0.009	0.049
Bacteria	Unclassifie	Unclassifie	OTU_1049	0.000	0.000	0.008	0.007	0.015
Bacteria	Acidobacte	Gp6	OTU_6204	0.000	0.000	0.009	0.008	0.022
Bacteria	Candidatus	Saccharibac	OTU_7465	0.000	0.000	0.009	0.008	0.022
Bacteria	Bacteroidet	Flavisoliba	OTU_5911	0.000	0.000	0.009	0.008	0.018
Bacteria	Unclassifie	Unclassifie	OTU_879	0.000	0.000	0.010	0.009	0.026
Bacteria	Actinobacte	Unclassifie	OTU_796	0.000	0.000	0.010	0.010	0.038
Bacteria	Candidatus	Saccharibac	OTU_2330	0.000	0.000	0.010	0.010	0.032
Bacteria	Proteobacte	Unclassifie	OTU_2154	0.003	0.003	0.013	0.008	0.010
Bacteria	Unclassifie	Unclassifie	OTU_2809	0.000	0.000	0.010	0.010	0.034
Bacteria	Candidatus	Saccharibac	OTU_7253	0.003	0.004	0.013	0.009	0.014
Bacteria	Planctomyc	Unclassifie	OTU_2045	0.000	0.000	0.011	0.008	0.012
Bacteria	Proteobacte	Unclassifie	OTU_1834	0.000	0.000	0.011	0.009	0.017
Bacteria	Planctomyc	Gemmata	OTU_660	0.003	0.003	0.013	0.010	0.027
Bacteria	Unclassifie	Unclassifie	OTU_1086	0.000	0.000	0.011	0.010	0.023
Archaea	Euryarchae	Methanoce	OTU_1594	0.000	0.000	0.011	0.011	0.029
Bacteria	candidate d	WPS-2_ger	OTU_863	0.001	0.003	0.013	0.007	0.004
Bacteria	Unclassifie	Unclassifie	OTU_5504	0.000	0.000	0.012	0.013	0.045
Bacteria	Chloroflexi	Ktedonobac	OTU_1028	0.000	0.000	0.012	0.013	0.044
Bacteria	Proteobacte	Nevskia	OTU_2526	0.004	0.006	0.017	0.012	0.031
Bacteria	Acidobacte	Gp1	OTU_1635	0.000	0.000	0.013	0.012	0.023
Bacteria	Chloroflexi	Unclassifie	OTU_1486	0.007	0.010	0.020	0.012	0.039
Bacteria	Firmicutes	Clostridium	OTU_1915	0.000	0.000	0.014	0.011	0.017
Bacteria	Proteobacte	Reyranella	OTU_451	0.008	0.007	0.021	0.012	0.024
Bacteria	Proteobacte	Unclassifie	OTU_1412	0.001	0.002	0.015	0.012	0.023
Bacteria	Planctomyc	Unclassifie	OTU_3370	0.005	0.005	0.019	0.014	0.041
Bacteria	Armatimon	Chthonomo	OTU_7409	0.000	0.000	0.015	0.012	0.013
Bacteria	Acidobacte	Acidipila	OTU_336	0.010	0.013	0.025	0.013	0.048
Bacteria	Acidobacte	Gp1	OTU_727	0.007	0.008	0.022	0.013	0.022
Bacteria	Proteobacte	Unclassifie	OTU_867	0.012	0.010	0.027	0.013	0.027
Bacteria	Proteobacte	Unclassifie	OTU_1605	0.004	0.005	0.020	0.016	0.036
Bacteria	Bacteroidet	Unclassifie	OTU_1449	0.004	0.007	0.020	0.007	0.001
Bacteria	Proteobacte	Magnetosp	OTU_930	0.006	0.010	0.022	0.012	0.016
Bacteria	Proteobacte	Unclassifie	OTU_351	0.004	0.006	0.020	0.017	0.047
Bacteria	Acidobacte	Telmatobac	OTU_3128	0.003	0.004	0.019	0.010	0.004
Bacteria	Proteobacte	Unclassifie	OTU_853	0.001	0.002	0.017	0.015	0.027
Bacteria	candidate d	WPS-1_ger	OTU_730	0.003	0.004	0.019	0.012	0.008
Bacteria	Planctomyc	Unclassifie	OTU_8726	0.006	0.009	0.023	0.013	0.014
Bacteria	Unclassifie	Unclassifie	OTU_790	0.006	0.006	0.022	0.012	0.009
Bacteria	Actinobacte	Unclassifie	OTU_1588	0.017	0.015	0.035	0.014	0.041
Bacteria	Proteobacte	Unclassifie	OTU_1092	0.001	0.002	0.019	0.018	0.032
Bacteria	Acidobacte	Unclassifie	OTU_4823	0.006	0.006	0.024	0.014	0.010
Bacteria	Unclassifie	Unclassifie	OTU_2385	0.000	0.000	0.018	0.016	0.021
Bacteria	Unclassifie	Unclassifie	OTU_669	0.000	0.000	0.019	0.021	0.050
Bacteria	Acidobacte	Gp3	OTU_760	0.002	0.003	0.023	0.018	0.020
Bacteria	Planctomyc	Singulispha	OTU_406	0.012	0.011	0.033	0.015	0.009
Bacteria	Acidobacte	Unclassifie	OTU_478	0.014	0.010	0.039	0.022	0.024
Bacteria	Proteobacte	Unclassifie	OTU_616	0.006	0.007	0.032	0.023	0.021
Bacteria	Unclassifie	Unclassifie	OTU_4187	0.010	0.011	0.036	0.017	0.006
Bacteria	Unclassifie	Unclassifie	OTU_854	0.000	0.000	0.026	0.029	0.048
Bacteria	Gemmatim	Gemmatim	OTU_531	0.007	0.007	0.033	0.015	0.002
Bacteria	Gemmatim	Gemmatim	OTU_9151	0.002	0.002	0.029	0.025	0.026
Bacteria	Proteobacte	Nevskia	OTU_547	0.015	0.019	0.042	0.025	0.039
Bacteria	Bacteroidet	Unclassifie	OTU_313	0.002	0.002	0.029	0.019	0.007
Bacteria	Acidobacte	Gp3	OTU_370	0.004	0.004	0.032	0.031	0.046
Bacteria	Bacteroidet	Chitinopha	OTU_324	0.000	0.000	0.029	0.023	0.013

Bacteria	Proteobacte	Labilithrix	OTU_440	0.016	0.016	0.045	0.028	0.035
Bacteria	Acidobacte	Gp6	OTU_444	0.004	0.004	0.034	0.031	0.038
Bacteria	Acidobacte	Unclassifie	OTU_609	0.003	0.003	0.033	0.029	0.028
Bacteria	Gemmatim	Gemmatim	OTU_7297	0.000	0.000	0.031	0.030	0.030
Bacteria	Acidobacte	Candidatus	OTU_1005	0.016	0.013	0.047	0.022	0.009
Bacteria	Actinobact	Geodermat	OTU_5883	0.021	0.011	0.052	0.032	0.038
Bacteria	Acidobacte	Granulicell	OTU_274	0.009	0.006	0.041	0.030	0.025
Bacteria	Actinobact	Unclassifie	OTU_243	0.010	0.008	0.042	0.030	0.026
Bacteria	Actinobact	Acidimicro	OTU_569	0.005	0.005	0.037	0.029	0.021
Bacteria	Candidatus	Saccharibac	OTU_575	0.000	0.000	0.033	0.035	0.044
Bacteria	Acidobacte	Acidobacte	OTU_318	0.007	0.004	0.041	0.037	0.044
Bacteria	Actinobact	Actinospica	OTU_626	0.018	0.024	0.053	0.034	0.044
Bacteria	Unclassifie	Unclassifie	OTU_290	0.003	0.006	0.039	0.026	0.008
Bacteria	Proteobacte	Aquabacter	OTU_4241	0.010	0.009	0.046	0.030	0.014
Bacteria	Gemmatim	Gemmatim	OTU_488	0.003	0.003	0.040	0.025	0.006
Bacteria	Chloroflexi	Ktedonobac	OTU_2596	0.000	0.000	0.038	0.033	0.020
Bacteria	Proteobacte	Lacibacteri	OTU_156	0.004	0.005	0.046	0.037	0.021
Bacteria	Acidobacte	Terriglobus	OTU_8499	0.009	0.008	0.051	0.037	0.020
Bacteria	Chloroflexi	Ktedonobac	OTU_158	0.000	0.000	0.047	0.049	0.039
Bacteria	candidate d	WPS-2_ge	OTU_186	0.015	0.017	0.065	0.034	0.006
Bacteria	Acidobacte	Granulicell	OTU_140	0.055	0.032	0.106	0.050	0.044
Bacteria	Acidobacte	Gp2	OTU_509	0.024	0.026	0.075	0.040	0.017
Bacteria	Firmicutes	Unclassifie	OTU_733	0.006	0.015	0.059	0.052	0.031
Bacteria	Chloroflexi	Ktedonobac	OTU_929	0.008	0.010	0.063	0.049	0.022
Bacteria	Proteobacte	Unclassifie	OTU_137	0.080	0.019	0.137	0.037	0.004
Bacteria	Planctomyc	Planctopiru	OTU_214	0.031	0.030	0.091	0.043	0.010
Bacteria	Proteobacte	Unclassifie	OTU_798	0.039	0.023	0.100	0.062	0.039
Bacteria	Proteobacte	Unclassifie	OTU_367	0.012	0.013	0.080	0.049	0.008
Bacteria	Actinobact	Unclassifie	OTU_7479	0.043	0.041	0.113	0.028	0.003
Bacteria	Proteobacte	Haliangium	OTU_395	0.025	0.016	0.099	0.060	0.013
Bacteria	Proteobacte	Unclassifie	OTU_386	0.022	0.009	0.097	0.037	0.001
Bacteria	Acidobacte	Terriglobus	OTU_188	0.029	0.019	0.106	0.032	0.000
Bacteria	Proteobacte	Kerstersia	OTU_259	0.002	0.004	0.081	0.059	0.009
Bacteria	Proteobacte	Unclassifie	OTU_157	0.034	0.015	0.122	0.069	0.011
Bacteria	Bacteroidet	Unclassifie	OTU_85	0.028	0.026	0.123	0.092	0.029
Bacteria	Proteobacte	Skermanell	OTU_129	0.008	0.010	0.103	0.059	0.004
Bacteria	Verrucomic	Subdivision	OTU_9278	0.034	0.052	0.141	0.096	0.026
Bacteria	Unclassifie	Unclassifie	OTU_181	0.008	0.014	0.114	0.109	0.036
Bacteria	Gemmatim	Gemmatim	OTU_87	0.044	0.071	0.157	0.112	0.044
Bacteria	Gemmatim	Gemmatim	OTU_98	0.018	0.013	0.134	0.095	0.014
Bacteria	Gemmatim	Gemmatim	OTU_65	0.001	0.002	0.127	0.138	0.047
Bacteria	Chloroflexi	Unclassifie	OTU_245	0.016	0.016	0.145	0.085	0.005
Bacteria	Planctomyc	Thermogut	OTU_187	0.035	0.032	0.169	0.070	0.001
Bacteria	Proteobacte	Rhizomicro	OTU_164	0.112	0.087	0.246	0.104	0.021
Bacteria	Chloroflexi	Unclassifie	OTU_53	0.027	0.026	0.183	0.169	0.045
Bacteria	Proteobacte	Unclassifie	OTU_151	0.010	0.015	0.168	0.077	0.001
Bacteria	Candidatus	Saccharibac	OTU_116	0.042	0.046	0.212	0.158	0.025
Bacteria	Acidobacte	Candidatus	OTU_113	0.196	0.131	0.377	0.175	0.047
Bacteria	Proteobacte	Sphingomo	OTU_49	0.148	0.096	0.338	0.202	0.049
Bacteria	Acidobacte	Geothrix	OTU_4589	0.064	0.043	0.263	0.176	0.020
Bacteria	Proteobacte	Burkholder	OTU_28	0.136	0.090	0.352	0.162	0.011
Bacteria	Chloroflexi	Unclassifie	OTU_112	0.071	0.076	0.288	0.160	0.009
Bacteria	Acidobacte	Gp3	OTU_80	0.075	0.027	0.292	0.230	0.041
Bacteria	Chloroflexi	Unclassifie	OTU_534	0.042	0.042	0.261	0.149	0.006
Bacteria	Chloroflexi	Unclassifie	OTU_37	0.118	0.093	0.350	0.241	0.041
Bacteria	Chloroflexi	Unclassifie	OTU_59	0.041	0.052	0.279	0.177	0.009

Bacteria	Actinobact	Unclassifie	OTU_84	0.035	0.042	0.280	0.102	0.000
Bacteria	Chloroflexi	Unclassifie	OTU_183	0.000	0.000	0.249	0.232	0.025
Bacteria	Acidobacte	Gp1	OTU_205	0.107	0.149	0.370	0.158	0.006
Bacteria	candidate d	WPS-1_ge	OTU_11	0.122	0.099	0.386	0.222	0.017
Bacteria	Firmicutes	Unclassifie	OTU_9	0.047	0.078	0.315	0.285	0.043
Bacteria	Actinobact	Unclassifie	OTU_51	0.103	0.094	0.377	0.143	0.001
Bacteria	Acidobacte	Unclassifie	OTU_91	0.117	0.101	0.392	0.279	0.037
Bacteria	Unclassifie	Unclassifie	OTU_15	0.062	0.036	0.349	0.231	0.013
Bacteria	Acidobacte	Gp1	OTU_61	0.017	0.022	0.347	0.155	0.001
Bacteria	Unclassifie	Unclassifie	OTU_29	0.136	0.120	0.572	0.267	0.003
Bacteria	Acidobacte	Gp1	OTU_26	0.184	0.135	0.680	0.507	0.037
Bacteria	Acidobacte	Geothrix	OTU_18	0.132	0.070	0.796	0.534	0.013
Bacteria	Proteobacte	Unclassifie	OTU_27	0.095	0.065	1.157	0.768	0.008
Bacteria	Proteobacte	Rhodanoba	OTU_5	0.331	0.284	1.872	1.115	0.008
Archaea	Thaumarch	Nitrososph	OTU_2	0.296	0.250	9.382	4.143	0.001
