# Supplementary material for: Microbial interactions and metabolisms in response to bacterial wilt and black shank pathogens in the tobacco rhizosphere
Source: Front Plant Sci. 2023 Jun 20;14:1200136. doi: 10.3389/fpls.2023.1200136 (PMC10319149; doi:10.3389/fpls.2023.1200136)
Supplement: Supplementary file 4 [file DataSheet_4.pdf]

Additional file 8

Table S8 Genera with significant differences between CK and BSM groups (Unit: %)

Domain	Phylum	Genus	OTU	CK: mean re	CK: std. dev	BSM: mean	BSM: std. de	p-values
Bacteria	Acidobacter	Gp4	OTU_74	1.449	0.811	0.090	0.135	0.003
Bacteria	Proteobacter	Sphingobium	OTU_6	0.782	0.557	0.210	0.135	0.030
Bacteria	Actinobacter	Streptomyce	OTU_3189	0.730	0.399	0.266	0.162	0.019
Bacteria	Verrucomicr	Spartobacter	OTU_71	0.705	0.366	0.300	0.188	0.026
Bacteria	Acidobacter	Gp6	OTU_155	0.422	0.177	0.103	0.131	0.002
Bacteria	Proteobacter	Unclassified	OTU_4511	0.319	0.146	0.098	0.053	0.005
Bacteria	Proteobacter	Sphingobium	OTU_38	0.235	0.100	0.032	0.033	0.001
Bacteria	Acidobacter	Gp6	OTU_201	0.261	0.167	0.065	0.068	0.018
Bacteria	Acidobacter	Gp16	OTU_77	0.306	0.124	0.115	0.135	0.015
Bacteria	Actinobacter	Lechevalieri	OTU_93	0.217	0.145	0.029	0.016	0.011
Bacteria	Acidobacter	Gp6	OTU_332	0.216	0.175	0.034	0.043	0.028
Bacteria	Acidobacter	Gp6	OTU_70	0.285	0.142	0.105	0.102	0.018
Bacteria	Proteobacter	Unclassified	OTU_134	0.216	0.136	0.041	0.032	0.011
Bacteria	Acidobacter	Gp4	OTU_285	0.222	0.103	0.056	0.074	0.004
Bacteria	Actinobacter	Gaiella	OTU_289	0.245	0.145	0.086	0.048	0.023
Bacteria	Proteobacter	Unclassified	OTU_202	0.174	0.106	0.017	0.017	0.006
Bacteria	Acidobacter	Gp4	OTU_1411	0.170	0.051	0.023	0.027	0.000
Bacteria	Acidobacter	Gp4	OTU_3764	0.146	0.099	0.003	0.005	0.006
Bacteria	Candidatus S	Saccharibact	OTU_76	0.174	0.145	0.033	0.056	0.040
Bacteria	Acidobacter	Gp6	OTU_508	0.157	0.123	0.023	0.023	0.023
Bacteria	Proteobacter	Ramlibacter	OTU_48	0.376	0.124	0.242	0.083	0.034
Bacteria	Proteobacter	Georgfuchsi	OTU_277	0.229	0.113	0.096	0.053	0.019
Bacteria	Acidobacter	Gp6	OTU_8332	0.215	0.065	0.083	0.101	0.013
Bacteria	Actinobacter	Unclassified	OTU_249	0.199	0.060	0.074	0.058	0.001
Bacteria	Proteobacter	Unclassified	OTU_4767	0.146	0.096	0.027	0.021	0.013
Bacteria	Proteobacter	Unclassified	OTU_341	0.155	0.081	0.048	0.049	0.012
Bacteria	Acidobacter	Gp4	OTU_2467	0.123	0.061	0.024	0.033	0.003
Bacteria	Acidobacter	Gp6	OTU_110	0.135	0.075	0.039	0.042	0.013
Bacteria	Acidobacter	Gp4	OTU_7758	0.105	0.098	0.011	0.015	0.039
Bacteria	Gemmatimo	Gemmatimo	OTU_198	0.145	0.078	0.052	0.065	0.031
Bacteria	candidate di	WPS-1_gen	OTU_464	0.097	0.085	0.005	0.007	0.024
Bacteria	Proteobacter	Arenimonas	OTU_130	0.170	0.067	0.078	0.082	0.039
Bacteria	Acidobacter	Gp6	OTU_2748	0.102	0.101	0.012	0.012	0.050
Bacteria	Acidobacter	Gp4	OTU_750	0.093	0.060	0.005	0.005	0.006
Bacteria	Actinobacter	Solirubrobac	OTU_497	0.098	0.082	0.011	0.010	0.026
Bacteria	Actinobacter	Blastococcu	OTU_115	0.133	0.078	0.048	0.025	0.023
Bacteria	Actinobacter	Solirubrobac	OTU_657	0.077	0.077	0.000	0.001	0.034
Bacteria	Proteobacter	Unclassified	OTU_618	0.107	0.048	0.030	0.028	0.004
Bacteria	Actinobacter	Aciditerrimo	OTU_638	0.087	0.073	0.013	0.016	0.030
Bacteria	Verrucomicr	Spartobacter	OTU_522	0.131	0.050	0.058	0.052	0.017
Bacteria	Acidobacter	Gp4	OTU_8714	0.081	0.060	0.009	0.011	0.016
Bacteria	Gemmatimo	Gemmatimo	OTU_296	0.087	0.079	0.016	0.018	0.047
Bacteria	Acidobacter	Gp6	OTU_3585	0.091	0.051	0.020	0.017	0.007
Bacteria	Acidobacter	Gp6	OTU_610	0.073	0.078	0.002	0.003	0.046
Bacteria	Unclassified	Unclassified	OTU_1942	0.075	0.064	0.005	0.006	0.023
Bacteria	Proteobacter	Unclassified	OTU_513	0.072	0.048	0.004	0.004	0.007
Bacteria	Proteobacter	Massilia	OTU_216	0.125	0.065	0.057	0.032	0.033
Bacteria	Proteobacter	Unclassified	OTU_242	0.126	0.033	0.059	0.026	0.001
Bacteria	Actinobacter	Gaiella	OTU_909	0.072	0.067	0.006	0.011	0.036
Bacteria	Proteobacter	Pelomonas	OTU_239	0.145	0.051	0.079	0.057	0.039
Bacteria	Verrucomicr	Unclassified	OTU_304	0.080	0.052	0.015	0.023	0.013
Bacteria	Actinobacter	Gaiella	OTU_587	0.079	0.062	0.014	0.019	0.028
Bacteria	Unclassified	Unclassified	OTU_506	0.076	0.056	0.011	0.012	0.019
Bacteria	Acidobacter	Gp6	OTU_270	0.066	0.065	0.003	0.003	0.035
Bacteria	Actinobacter	Unclassified	OTU_7451	0.092	0.056	0.030	0.022	0.024
Bacteria	Acidobacter	Gp6	OTU_6956	0.079	0.047	0.017	0.014	0.010
Bacteria	Actinobacter	Gaiella	OTU_1345	0.073	0.048	0.011	0.015	0.011
Bacteria	Proteobacter	Unclassified	OTU_589	0.097	0.054	0.038	0.023	0.023
Bacteria	Gemmatimo	Gemmatimo	OTU_293	0.088	0.027	0.028	0.016	0.000
Bacteria	Proteobacter	Pseudoduga	OTU_178	0.124	0.045	0.065	0.039	0.020
Bacteria	Acidobacter	Gp4	OTU_2388	0.066	0.055	0.007	0.013	0.026

Bacteria	Gemmatimo	Gemmatimo	OTU_260	0.066	0.061	0.008	0.009	0.040
Bacteria	Acidobacter	Unclassified	OTU_2086	0.076	0.054	0.019	0.023	0.030
Bacteria	Candidatus S	Saccharibact	OTU_882	0.054	0.052	0.000	0.000	0.027
Bacteria	Acidobacter	Gp4	OTU_8027	0.061	0.044	0.009	0.011	0.015
Bacteria	Actinobacter	Solirubrobac	OTU_2102	0.060	0.047	0.008	0.007	0.021
Bacteria	Actinobacter	Gaiella	OTU_382	0.069	0.054	0.017	0.017	0.043
Bacteria	Actinobacter	Gaiella	OTU_2538	0.051	0.048	0.000	0.000	0.025
Bacteria	Acidobacter	Gp6	OTU_6070	0.066	0.054	0.015	0.020	0.045
Bacteria	candidate di	WPS-1_gen	OTU_816	0.061	0.035	0.011	0.010	0.006
Bacteria	Acidobacter	Gp4	OTU_1101	0.057	0.029	0.006	0.005	0.002
Bacteria	Actinobacter	Gaiella	OTU_8708	0.062	0.044	0.012	0.015	0.020
Bacteria	Actinobacter	Ilumatobact	OTU_501	0.054	0.047	0.005	0.005	0.027
Bacteria	Actinobacter	Gaiella	OTU_366	0.060	0.021	0.011	0.012	0.000
Bacteria	Acidobacter	Gp6	OTU_159	0.087	0.041	0.038	0.022	0.018
Bacteria	Actinobacter	Gaiella	OTU_702	0.071	0.034	0.023	0.020	0.008
Bacteria	Proteobacter	Unclassified	OTU_527	0.054	0.045	0.007	0.007	0.028
Bacteria	Proteobacter	Haliangium	OTU_3989	0.077	0.025	0.030	0.021	0.002
Bacteria	Verrucomicr	Spartobacter	OTU_1311	0.087	0.046	0.041	0.018	0.036
Bacteria	Actinobacter	Gaiella	OTU_335	0.073	0.031	0.027	0.024	0.009
Bacteria	Acidobacter	Gp6	OTU_7014	0.062	0.036	0.016	0.018	0.013
Bacteria	Proteobacter	Unclassified	OTU_463	0.079	0.034	0.034	0.031	0.021
Bacteria	Acidobacter	Gp6	OTU_1906	0.077	0.022	0.032	0.032	0.010
Bacteria	Unclassified	Unclassified	OTU_658	0.058	0.037	0.014	0.021	0.020
Bacteria	Proteobacter	Unclassified	OTU_7432	0.068	0.035	0.024	0.017	0.013
Bacteria	Actinobacter	Conexibacte	OTU_500	0.047	0.033	0.004	0.005	0.011
Bacteria	Chloroflexi	Unclassified	OTU_1107	0.059	0.034	0.016	0.016	0.012
Bacteria	Actinobacter	Conexibacte	OTU_353	0.068	0.033	0.026	0.016	0.013
Bacteria	Actinobacter	Asanoa	OTU_6734	0.042	0.042	0.000	0.000	0.032
Bacteria	Unclassified	Unclassified	OTU_956	0.047	0.042	0.005	0.006	0.033
Bacteria	Actinobacter	Unclassified	OTU_1717	0.047	0.033	0.006	0.009	0.012
Bacteria	Verrucomicr	Spartobacter	OTU_614	0.042	0.039	0.002	0.003	0.031
Bacteria	Acidobacter	Aridibacter	OTU_696	0.044	0.031	0.006	0.008	0.012
Bacteria	Proteobacter	Unclassified	OTU_1096	0.049	0.026	0.010	0.010	0.005
Bacteria	Armatimona	Armatimona	OTU_258	0.063	0.025	0.024	0.025	0.012
Bacteria	Unclassified	Unclassified	OTU_4079	0.046	0.037	0.008	0.005	0.027
Bacteria	candidate di	WPS-1_gen	OTU_716	0.042	0.028	0.004	0.004	0.009
Bacteria	Verrucomicr	Subdivision3	OTU_268	0.072	0.032	0.035	0.023	0.027
Bacteria	Proteobacter	Unclassified	OTU_414	0.051	0.027	0.014	0.019	0.011
Bacteria	Proteobacter	Unclassified	OTU_822	0.044	0.038	0.007	0.009	0.038
Bacteria	Proteobacter	Unclassified	OTU_1616	0.049	0.036	0.012	0.010	0.030
Bacteria	Actinobacter	Nocardioide	OTU_3526	0.041	0.035	0.004	0.004	0.026
Bacteria	Acidobacter	Gp6	OTU_345	0.049	0.030	0.012	0.011	0.015
Bacteria	Acidobacter	Gp10	OTU_561	0.039	0.034	0.002	0.002	0.026
Bacteria	Actinobacter	Unclassified	OTU_3055	0.048	0.025	0.011	0.017	0.008
Bacteria	candidate di	WPS-1_gen	OTU_479	0.059	0.034	0.022	0.015	0.025
Bacteria	Acidobacter	Gp6	OTU_1025	0.054	0.038	0.017	0.014	0.041
Bacteria	Proteobacter	Lysobacter	OTU_814	0.043	0.034	0.007	0.007	0.028
Bacteria	Proteobacter	Povalibacter	OTU_350	0.041	0.034	0.006	0.006	0.030
Bacteria	Unclassified	Unclassified	OTU_403	0.045	0.033	0.010	0.011	0.028
Bacteria	Proteobacter	Pedomicrobi	OTU_1126	0.042	0.025	0.007	0.008	0.008
Bacteria	Acidobacter	Gp6	OTU_6273	0.046	0.028	0.011	0.010	0.014
Bacteria	Unclassified	Unclassified	OTU_1346	0.051	0.031	0.016	0.009	0.023
Bacteria	Verrucomicr	Subdivision3	OTU_493	0.050	0.025	0.017	0.019	0.015
Bacteria	Proteobacter	Unclassified	OTU_782	0.049	0.033	0.016	0.021	0.044
Bacteria	Unclassified	Unclassified	OTU_449	0.046	0.031	0.013	0.012	0.027
Bacteria	Proteobacter	Sphingomon	OTU_1805	0.039	0.036	0.006	0.004	0.047
Bacteria	Armatimona	Armatimona	OTU_582	0.041	0.022	0.008	0.008	0.006
Bacteria	Unclassified	Unclassified	OTU_1026	0.037	0.032	0.005	0.007	0.032
Bacteria	Proteobacter	Unclassified	OTU_921	0.034	0.021	0.002	0.004	0.005
Bacteria	Latescibacte	Latescibacte	OTU_5436	0.046	0.033	0.014	0.017	0.046
Bacteria	candidate di	WPS-1_gen	OTU_591	0.049	0.023	0.018	0.012	0.010
Bacteria	Proteobacter	Unclassified	OTU_1169	0.045	0.027	0.014	0.010	0.020
Bacteria	Bacteroidete	Unclassified	OTU_1185	0.035	0.032	0.004	0.006	0.039
Bacteria	Acidobacter	Gp6	OTU_674	0.032	0.027	0.001	0.001	0.020
Bacteria	Proteobacter	Sphingomon	OTU_1414	0.037	0.017	0.007	0.005	0.002

Bacteria	Proteobacter	Pseudorhodo	OTU_7191	0.082	0.023	0.051	0.017	0.016
Bacteria	Actinobacter	Gaiella	OTU_755	0.057	0.025	0.026	0.023	0.034
Bacteria	Actinobacter	Aquihabitan	OTU_1180	0.040	0.022	0.010	0.011	0.008
Bacteria	Actinobacter	Gaiella	OTU_935	0.037	0.012	0.007	0.005	0.000
Bacteria	Actinobacter	Gaiella	OTU_1998	0.038	0.025	0.008	0.008	0.015
Bacteria	Bacteroidete	Terrimonas	OTU_1390	0.041	0.032	0.012	0.009	0.049
Bacteria	Acidobacter	Gp3	OTU_1020	0.035	0.030	0.006	0.005	0.040
Bacteria	Gemmatimo	Gemmatimo	OTU_354	0.037	0.013	0.008	0.007	0.000
Bacteria	candidate di	WPS-1_gen	OTU_776	0.034	0.031	0.005	0.007	0.045
Bacteria	Gemmatimo	Gemmatimo	OTU_1104	0.031	0.018	0.002	0.003	0.004
Bacteria	Actinobacter	Virgisporan	OTU_1436	0.035	0.022	0.008	0.011	0.013
Bacteria	Acidobacter	Gp6	OTU_7800	0.029	0.020	0.002	0.003	0.008
Bacteria	Actinobacter	Unclassified	OTU_8659	0.037	0.030	0.009	0.007	0.048
Bacteria	Acidobacter	Gp6	OTU_3140	0.037	0.027	0.010	0.014	0.037
Bacteria	Actinobacter	Unclassified	OTU_391	0.043	0.015	0.016	0.007	0.002
Bacteria	Unclassified	Unclassified	OTU_1623	0.034	0.021	0.007	0.008	0.011
Bacteria	Proteobacter	Anderseniell	OTU_941	0.030	0.025	0.003	0.005	0.028
Bacteria	Proteobacter	Novosphing	OTU_7005	0.034	0.026	0.007	0.004	0.032
Bacteria	Proteobacter	Unclassified	OTU_5265	0.037	0.021	0.012	0.008	0.014
Bacteria	candidate di	WPS-1_gen	OTU_1535	0.028	0.018	0.002	0.003	0.008
Bacteria	Actinobacter	Conexibacte	OTU_560	0.030	0.016	0.004	0.004	0.004
Bacteria	Chloroflexi	Unclassified	OTU_1171	0.029	0.022	0.004	0.004	0.018
Bacteria	Planctomyce	Pirellula	OTU_1711	0.036	0.012	0.011	0.013	0.002
Bacteria	Acidobacter	Gp17	OTU_2055	0.032	0.024	0.007	0.012	0.034
Bacteria	candidate di	WPS-1_gen	OTU_797	0.026	0.018	0.001	0.002	0.008
Bacteria	Acidobacter	Gp4	OTU_1424	0.026	0.019	0.001	0.003	0.012
Bacteria	Actinobacter	Thermoleop	OTU_1112	0.031	0.014	0.007	0.007	0.002
Bacteria	Acidobacter	Gp4	OTU_842	0.030	0.014	0.006	0.007	0.002
Bacteria	Verrucomicr	Spartobacter	OTU_976	0.026	0.022	0.002	0.002	0.024
Bacteria	Armatimona	Armatimona	OTU_1589	0.030	0.017	0.007	0.008	0.008
Bacteria	Unclassified	Unclassified	OTU_2969	0.024	0.019	0.001	0.001	0.014
Bacteria	Unclassified	Unclassified	OTU_751	0.029	0.020	0.006	0.009	0.017
Bacteria	Acidobacter	Aridibacter	OTU_6837	0.029	0.023	0.005	0.005	0.030
Bacteria	candidate di	WPS-1_gen	OTU_1919	0.029	0.025	0.006	0.008	0.044
Bacteria	Actinobacter	Conexibacte	OTU_4156	0.034	0.021	0.011	0.009	0.024
Bacteria	Acidobacter	Gp3	OTU_7700	0.034	0.021	0.011	0.016	0.037
Bacteria	Unclassified	Unclassified	OTU_1719	0.023	0.022	0.000	0.001	0.033
Bacteria	Bacteroidete	Unclassified	OTU_1718	0.028	0.023	0.006	0.007	0.037
Bacteria	Actinobacter	Conexibacte	OTU_358	0.054	0.018	0.031	0.016	0.030
Bacteria	Acidobacter	Gp3	OTU_1017	0.027	0.024	0.005	0.006	0.048
Bacteria	Acidobacter	Gp7	OTU_904	0.023	0.022	0.001	0.001	0.031
Bacteria	Proteobacter	Unclassified	OTU_642	0.027	0.018	0.006	0.006	0.015
Bacteria	candidate di	WPS-1_gen	OTU_8125	0.026	0.020	0.005	0.006	0.026
Bacteria	Proteobacter	Unclassified	OTU_1051	0.026	0.011	0.005	0.005	0.001
Bacteria	Actinobacter	Aquihabitan	OTU_684	0.026	0.021	0.004	0.004	0.032
Bacteria	Acidobacter	Gp6	OTU_4035	0.031	0.022	0.010	0.007	0.041
Bacteria	Acidobacter	Gp6	OTU_2420	0.029	0.006	0.009	0.007	0.000
Bacteria	Actinobacter	Conexibacte	OTU_1676	0.026	0.022	0.005	0.006	0.043
Bacteria	Bacteroidete	Unclassified	OTU_8205	0.021	0.017	0.001	0.002	0.016
Bacteria	Actinobacter	Gaiella	OTU_726	0.031	0.018	0.011	0.010	0.027
Bacteria	candidate di	WPS-1_gen	OTU_6891	0.023	0.020	0.003	0.007	0.037
Bacteria	Proteobacter	Nitrosospira	OTU_4299	0.027	0.016	0.007	0.005	0.013
Bacteria	Acidobacter	Gp7	OTU_1601	0.021	0.017	0.001	0.002	0.017
Bacteria	Verrucomicr	Subdivision3	OTU_5426	0.041	0.008	0.022	0.017	0.019
Bacteria	Actinobacter	Unclassified	OTU_5308	0.022	0.020	0.003	0.004	0.033
Bacteria	candidate di	WPS-1_gen	OTU_995	0.020	0.019	0.001	0.001	0.028
Bacteria	Proteobacter	Povalibacter	OTU_1074	0.023	0.017	0.004	0.003	0.018
Bacteria	Verrucomicr	Subdivision3	OTU_1194	0.022	0.017	0.003	0.003	0.019
Bacteria	Verrucomicr	Subdivision3	OTU_483	0.027	0.016	0.008	0.008	0.018
Bacteria	Actinobacter	Gaiella	OTU_1070	0.028	0.015	0.009	0.007	0.014
Bacteria	Proteobacter	Unclassified	OTU_1270	0.020	0.015	0.002	0.003	0.013
Bacteria	Acidobacter	Gp4	OTU_770	0.022	0.020	0.004	0.005	0.046
Bacteria	Bacteroidete	Niastella	OTU_1837	0.023	0.015	0.005	0.005	0.015
Bacteria	Proteobacter	Unclassified	OTU_1541	0.027	0.013	0.009	0.010	0.011
Bacteria	Unclassified	Unclassified	OTU_3201	0.020	0.017	0.002	0.006	0.027

Bacteria	Gemmatimo	Gemmatimo	OTU_412	0.022	0.012	0.005	0.004	0.004
Bacteria	Proteobacter	Pseudolabry	OTU_7843	0.034	0.012	0.016	0.010	0.009
Bacteria	Bacteroidete	Unclassified	OTU_957	0.029	0.012	0.012	0.006	0.006
Bacteria	Actinobacter	Gaiella	OTU_7346	0.018	0.019	0.000	0.001	0.043
Bacteria	Proteobacter	Labilithrix	OTU_604	0.025	0.017	0.007	0.006	0.030
Bacteria	Acidobacter	Gp3	OTU_3754	0.023	0.016	0.007	0.009	0.039
Bacteria	Proteobacter	Unclassified	OTU_2100	0.022	0.015	0.006	0.005	0.023
Bacteria	Proteobacter	Microvirga	OTU_1997	0.021	0.018	0.005	0.003	0.047
Bacteria	Planctomyce	Gemmata	OTU_2315	0.022	0.015	0.006	0.005	0.025
Bacteria	Unclassified	Unclassified	OTU_1603	0.017	0.018	0.001	0.002	0.046
Bacteria	Candidatus S	Saccharibact	OTU_795	0.028	0.015	0.012	0.011	0.047
Bacteria	Proteobacter	Caulobacter	OTU_562	0.030	0.008	0.015	0.009	0.004
Bacteria	Actinobacter	Unclassified	OTU_1599	0.018	0.013	0.002	0.003	0.017
Bacteria	Armatimona	Armatimona	OTU_788	0.020	0.013	0.004	0.004	0.015
Bacteria	candidate di	WPS-1_gen	OTU_920	0.018	0.009	0.003	0.003	0.002
Bacteria	Proteobacter	Panacagrimo	OTU_573	0.027	0.014	0.012	0.009	0.031
Bacteria	Acidobacter	Gp6	OTU_7880	0.019	0.014	0.004	0.005	0.026
Bacteria	Acidobacter	Gp25	OTU_1538	0.015	0.016	0.000	0.000	0.045
Bacteria	Gemmatimo	Gemmatimo	OTU_486	0.014	0.011	0.000	0.000	0.009
Bacteria	Proteobacter	Unclassified	OTU_1434	0.016	0.013	0.002	0.003	0.025
Bacteria	Verrucomicr	Terrimicrobi	OTU_2332	0.015	0.012	0.001	0.001	0.017
Bacteria	Proteobacter	Unclassified	OTU_2431	0.016	0.014	0.003	0.005	0.034
Bacteria	Proteobacter	Unclassified	OTU_1496	0.014	0.013	0.001	0.001	0.028
Bacteria	Planctomyce	Pirellula	OTU_2971	0.015	0.011	0.001	0.002	0.015
Bacteria	Unclassified	Unclassified	OTU_2794	0.014	0.010	0.001	0.001	0.009
Bacteria	Unclassified	Unclassified	OTU_1281	0.015	0.014	0.002	0.002	0.038
Bacteria	Acidobacter	Gp16	OTU_1249	0.016	0.012	0.003	0.003	0.025
Bacteria	Unclassified	Unclassified	OTU_3622	0.013	0.013	0.000	0.000	0.029
Bacteria	Actinobacter	Unclassified	OTU_2283	0.019	0.013	0.006	0.008	0.040
Bacteria	Proteobacter	Unclassified	OTU_8769	0.015	0.013	0.002	0.003	0.038
Bacteria	Chloroflexi	Unclassified	OTU_2778	0.014	0.011	0.001	0.001	0.016
Bacteria	Acidobacter	Bryobacter	OTU_1316	0.016	0.010	0.003	0.004	0.012
Bacteria	Acidobacter	Gp6	OTU_6984	0.013	0.012	0.000	0.000	0.029
Bacteria	Bacteroidete	Chryseoline	OTU_960	0.016	0.013	0.003	0.003	0.037
Bacteria	Proteobacter	Unclassified	OTU_1465	0.018	0.009	0.006	0.004	0.009
Bacteria	Bacteroidete	Flavisolibac	OTU_6302	0.015	0.011	0.003	0.004	0.021
Bacteria	Proteobacter	Unclassified	OTU_1721	0.013	0.008	0.001	0.001	0.004
Bacteria	Proteobacter	Noviherbasp	OTU_7351	0.015	0.006	0.002	0.002	0.001
Bacteria	Actinobacter	Unclassified	OTU_1062	0.014	0.013	0.001	0.001	0.044
Bacteria	Unclassified	Unclassified	OTU_1292	0.018	0.009	0.006	0.006	0.013
Bacteria	Unclassified	Unclassified	OTU_1557	0.013	0.013	0.001	0.002	0.042
Bacteria	Verrucomicr	Subdivision3	OTU_1517	0.012	0.013	0.000	0.000	0.038
Bacteria	Proteobacter	Bauldia	OTU_1063	0.020	0.012	0.008	0.008	0.043
Bacteria	Verrucomicr	Subdivision3	OTU_3179	0.013	0.012	0.001	0.002	0.038
Bacteria	candidate di	WPS-1_gen	OTU_1965	0.014	0.012	0.002	0.002	0.036
Bacteria	Unclassified	Unclassified	OTU_1863	0.012	0.013	0.000	0.000	0.046
Bacteria	Planctomyce	Tepidisphae	OTU_4314	0.013	0.012	0.001	0.002	0.038
Bacteria	Actinobacter	Unclassified	OTU_3931	0.012	0.010	0.001	0.001	0.020
Bacteria	Verrucomicr	Subdivision3	OTU_3356	0.013	0.011	0.002	0.002	0.032
Bacteria	Proteobacter	Unclassified	OTU_3102	0.014	0.010	0.003	0.003	0.024
Bacteria	Verrucomicr	Spartobacter	OTU_6076	0.013	0.011	0.002	0.002	0.027
Bacteria	candidate di	WPS-1_gen	OTU_2873	0.012	0.008	0.001	0.002	0.008
Bacteria	Proteobacter	Unclassified	OTU_5005	0.013	0.012	0.002	0.003	0.040
Bacteria	Chloroflexi	Unclassified	OTU_2769	0.011	0.009	0.000	0.000	0.011
Bacteria	Proteobacter	Unclassified	OTU_1883	0.013	0.010	0.001	0.002	0.019
Bacteria	Proteobacter	Labilithrix	OTU_1193	0.020	0.009	0.008	0.005	0.017
Bacteria	Actinobacter	Saccharothri	OTU_2471	0.016	0.007	0.005	0.005	0.005
Bacteria	candidate di	WPS-1_gen	OTU_1377	0.015	0.012	0.003	0.003	0.045
Bacteria	Firmicutes	Unclassified	OTU_2845	0.012	0.007	0.001	0.002	0.004
Bacteria	Actinobacter	Actinoplane	OTU_1936	0.012	0.012	0.001	0.001	0.041
Bacteria	Proteobacter	Unclassified	OTU_830	0.014	0.006	0.003	0.003	0.002
Bacteria	Actinobacter	Aquihabitan	OTU_1283	0.016	0.012	0.005	0.003	0.042
Bacteria	Verrucomicr	Subdivision3	OTU_2272	0.015	0.009	0.004	0.005	0.019
Bacteria	Armatimona	Chthonomor	OTU_1984	0.014	0.011	0.004	0.004	0.047
Bacteria	Planctomyce	Pirellula	OTU_1492	0.017	0.010	0.007	0.006	0.039

Bacteria	Actinobacter	Unclassified	OTU_2744	0.014	0.007	0.004	0.003	0.005
Bacteria	Proteobacter	Unclassified	OTU_7271	0.012	0.010	0.001	0.002	0.032
Bacteria	Acidobacter	Gp16	OTU_2250	0.010	0.011	0.000	0.000	0.035
Bacteria	Actinobacter	Nocardioide	OTU_4115	0.017	0.009	0.007	0.006	0.024
Bacteria	Acidobacter	Gp3	OTU_1798	0.012	0.008	0.001	0.002	0.011
Bacteria	Planctomyce	Singulisphae	OTU_9305	0.013	0.010	0.003	0.003	0.031
Bacteria	Actinobacter	Nocardioide	OTU_2483	0.013	0.008	0.003	0.005	0.014
Bacteria	Unclassified	Unclassified	OTU_5626	0.011	0.010	0.001	0.002	0.029
Bacteria	Proteobacter	Unclassified	OTU_3039	0.010	0.007	0.000	0.000	0.007
Bacteria	candidate di	WPS-1_gen	OTU_7250	0.011	0.010	0.001	0.001	0.029
Bacteria	Acidobacter	Gp25	OTU_256	0.010	0.011	0.000	0.000	0.046
Bacteria	Gemmatimo	Gemmatimo	OTU_1241	0.011	0.008	0.001	0.001	0.015
Bacteria	Chloroflexi	Unclassified	OTU_2208	0.011	0.009	0.001	0.001	0.029
Bacteria	Acidobacter	Blastocatella	OTU_2445	0.011	0.008	0.001	0.003	0.016
Bacteria	candidate di	WPS-1_gen	OTU_2446	0.010	0.008	0.001	0.001	0.016
Bacteria	Proteobacter	Geminicocc	OTU_2260	0.009	0.010	0.000	0.000	0.043
Bacteria	Planctomyce	Unclassified	OTU_2541	0.012	0.010	0.003	0.003	0.038
Bacteria	Bacteroidete	Chryseoline	OTU_2417	0.010	0.006	0.001	0.001	0.003
Bacteria	Acidobacter	Gp6	OTU_3297	0.009	0.007	0.000	0.001	0.010
Bacteria	Proteobacter	Unclassified	OTU_2151	0.009	0.009	0.000	0.000	0.038
Bacteria	Proteobacter	Unclassified	OTU_2387	0.017	0.007	0.008	0.008	0.047
Bacteria	Planctomyce	Zavarzinella	OTU_2294	0.013	0.008	0.004	0.006	0.041
Bacteria	Acidobacter	Gp6	OTU_355	0.010	0.008	0.001	0.001	0.024
Bacteria	Planctomyce	Unclassified	OTU_1532	0.012	0.007	0.003	0.003	0.016
Bacteria	Proteobacter	Unclassified	OTU_1004	0.009	0.007	0.000	0.000	0.017
Bacteria	Unclassified	Unclassified	OTU_4392	0.009	0.007	0.000	0.000	0.015
Bacteria	Actinobacter	Crossiella	OTU_2371	0.013	0.008	0.004	0.006	0.035
Bacteria	Candidatus S	Saccharibact	OTU_5805	0.009	0.007	0.000	0.000	0.015
Bacteria	Actinobacter	Unclassified	OTU_7038	0.010	0.008	0.002	0.003	0.033
Bacteria	Proteobacter	Unclassified	OTU_3194	0.012	0.007	0.003	0.003	0.013
Bacteria	Planctomyce	Unclassified	OTU_2244	0.009	0.006	0.001	0.001	0.005
Bacteria	Actinobacter	Conexibacte	OTU_612	0.009	0.008	0.001	0.002	0.034
Bacteria	Armatimona	Armatimona	OTU_2615	0.011	0.007	0.002	0.002	0.022
Bacteria	Actinobacter	Catelliglobo	OTU_3483	0.009	0.006	0.001	0.001	0.013
Bacteria	Proteobacter	Enhygromyx	OTU_1851	0.009	0.007	0.001	0.001	0.023
Bacteria	Bacteroidete	Unclassified	OTU_4163	0.009	0.008	0.001	0.001	0.030
Bacteria	Armatimona	Chthonomor	OTU_2549	0.008	0.008	0.001	0.001	0.034
Bacteria	Proteobacter	Rhodopseud	OTU_959	0.010	0.007	0.002	0.002	0.026
Bacteria	Proteobacter	Unclassified	OTU_7407	0.017	0.007	0.009	0.006	0.043
Bacteria	Bacteroidete	Terrimonas	OTU_4471	0.008	0.007	0.001	0.001	0.024
Bacteria	Planctomyce	Pirellula	OTU_6664	0.009	0.006	0.002	0.003	0.011
Bacteria	Actinobacter	Conexibacte	OTU_5450	0.009	0.007	0.001	0.002	0.026
Bacteria	Actinobacter	Cellulomona	OTU_1938	0.012	0.007	0.004	0.005	0.041
Bacteria	Bacteroidete	Chitinophag	OTU_2805	0.008	0.008	0.001	0.001	0.046
Bacteria	Proteobacter	Unclassified	OTU_3392	0.007	0.005	0.000	0.000	0.007
Bacteria	Bacteroidete	Flavitalea	OTU_3621	0.010	0.008	0.002	0.002	0.047
Bacteria	Proteobacter	Unclassified	OTU_3786	0.007	0.006	0.000	0.000	0.018
Bacteria	Unclassified	Unclassified	OTU_3933	0.007	0.007	0.000	0.000	0.035
Bacteria	Proteobacter	Stella	OTU_9383	0.010	0.007	0.003	0.003	0.032
Bacteria	Planctomyce	Zavarzinella	OTU_5281	0.008	0.006	0.001	0.002	0.021
Bacteria	Proteobacter	Unclassified	OTU_6747	0.007	0.007	0.000	0.000	0.025
Bacteria	Planctomyce	Planctopirus	OTU_2128	0.008	0.007	0.001	0.002	0.036
Bacteria	Verrucomicr	Subdivision3	OTU_9375	0.008	0.008	0.001	0.002	0.049
Bacteria	Actinobacter	Aciditerrimo	OTU_2084	0.008	0.006	0.001	0.002	0.015
Bacteria	Bacteroidete	Flavobacteri	OTU_2345	0.008	0.007	0.001	0.001	0.045
Bacteria	Bacteroidete	Terrimonas	OTU_4309	0.009	0.006	0.002	0.003	0.018
Bacteria	Actinobacter	Gaiella	OTU_663	0.011	0.004	0.005	0.006	0.028
Bacteria	Verrucomicr	Spartobacter	OTU_2401	0.007	0.007	0.000	0.000	0.042
Bacteria	Actinobacter	Iamia	OTU_2518	0.008	0.003	0.001	0.003	0.000
Bacteria	Proteobacter	Chelatococc	OTU_2012	0.006	0.006	0.000	0.000	0.029
Bacteria	Planctomyce	Unclassified	OTU_1478	0.006	0.005	0.000	0.000	0.014
Bacteria	Acidobacter	Gp6	OTU_7338	0.008	0.006	0.001	0.002	0.024
Bacteria	Armatimona	Armatimona	OTU_3113	0.006	0.006	0.000	0.000	0.025
Bacteria	candidate di	WPS-1_gen	OTU_9312	0.006	0.005	0.000	0.000	0.017
Bacteria	Unclassified	Unclassified	OTU_2003	0.008	0.006	0.001	0.002	0.028

Bacteria	Actinobacte	Gaiella	OTU_9030	0.006	0.005	0.000	0.000	0.012
Bacteria	Unclassified	Unclassified	OTU_2080	0.006	0.006	0.000	0.000	0.037
Bacteria	Acidobacter	Gp6	OTU_2521	0.007	0.005	0.001	0.001	0.014
Bacteria	Armatimona	Armatimona	OTU_4866	0.006	0.005	0.000	0.000	0.011
Bacteria	Proteobacte	Unclassified	OTU_3076	0.006	0.006	0.000	0.000	0.033
Bacteria	candidate di	WPS-1_gen	OTU_2548	0.006	0.006	0.000	0.001	0.038
Bacteria	Acidobacter	Gp10	OTU_1677	0.009	0.006	0.003	0.004	0.036
Bacteria	Actinobacte	Conexibacte	OTU_2276	0.006	0.005	0.000	0.000	0.021
Bacteria	Actinobacte	Nakamurella	OTU_9035	0.008	0.006	0.002	0.002	0.025
Bacteria	Actinobacte	Iamia	OTU_4097	0.007	0.006	0.001	0.001	0.032
Bacteria	Armatimona	Armatimona	OTU_2872	0.006	0.006	0.000	0.001	0.028
Bacteria	Actinobacte	Unclassified	OTU_6829	0.007	0.006	0.001	0.001	0.046
Bacteria	candidate di	WPS-1_gen	OTU_7605	0.007	0.006	0.001	0.001	0.032
Bacteria	Acidobacter	Gp16	OTU_2911	0.007	0.006	0.001	0.002	0.032
Bacteria	candidate di	WPS-1_gen	OTU_6717	0.006	0.006	0.000	0.000	0.045
Bacteria	Gemmatimo	Gemmatimo	OTU_5487	0.009	0.006	0.003	0.003	0.042
Bacteria	Proteobacte	Unclassified	OTU_8333	0.009	0.006	0.004	0.003	0.041
Bacteria	Chloroflexi	Unclassified	OTU_3749	0.006	0.005	0.000	0.000	0.026
Bacteria	Planctomyce	Unclassified	OTU_3806	0.006	0.004	0.000	0.000	0.012
Bacteria	Unclassified	Unclassified	OTU_2504	0.005	0.004	0.000	0.000	0.008
Bacteria	Proteobacte	Unclassified	OTU_1459	0.006	0.005	0.000	0.001	0.036
Bacteria	Gemmatimo	Gemmatimo	OTU_6895	0.005	0.005	0.000	0.000	0.031
Bacteria	Planctomyce	Tepidisphae	OTU_5781	0.005	0.006	0.000	0.000	0.042
Bacteria	Actinobacte	Sphaerispora	OTU_3720	0.006	0.005	0.001	0.001	0.036
Bacteria	Actinobacte	Gaiella	OTU_3444	0.006	0.006	0.001	0.001	0.045
Bacteria	Acidobacter	Blastocatella	OTU_1741	0.007	0.005	0.002	0.003	0.044
Bacteria	Unclassified	Unclassified	OTU_5952	0.005	0.005	0.000	0.000	0.037
Bacteria	Planctomyce	Planctomicr	OTU_3460	0.005	0.003	0.000	0.000	0.002
Bacteria	Acidobacter	Gp6	OTU_2883	0.006	0.005	0.001	0.001	0.026
Bacteria	Verrucomic	Unclassified	OTU_4063	0.005	0.006	0.000	0.000	0.050
Bacteria	Unclassified	Unclassified	OTU_3601	0.005	0.005	0.000	0.000	0.031
Bacteria	Proteobacte	Unclassified	OTU_8293	0.006	0.004	0.001	0.002	0.019
Bacteria	Proteobacte	Unclassified	OTU_2409	0.006	0.005	0.001	0.002	0.043
Bacteria	Actinobacte	Aquihabitan	OTU_7295	0.009	0.005	0.004	0.003	0.038
Bacteria	Unclassified	Unclassified	OTU_1318	0.005	0.005	0.000	0.000	0.027
Bacteria	Planctomyce	Pirellula	OTU_3432	0.005	0.004	0.000	0.000	0.017
Bacteria	Acidobacter	Gp17	OTU_8421	0.006	0.003	0.001	0.002	0.003
Bacteria	Acidobacter	Gp6	OTU_5353	0.005	0.005	0.000	0.000	0.028
Bacteria	Chloroflexi	Unclassified	OTU_6189	0.005	0.005	0.000	0.001	0.027
Bacteria	Armatimona	Armatimona	OTU_3382	0.005	0.004	0.000	0.000	0.013
Bacteria	Planctomyce	Zavarzinella	OTU_5824	0.006	0.005	0.001	0.001	0.029
Bacteria	Unclassified	Unclassified	OTU_7035	0.005	0.004	0.001	0.001	0.022
Bacteria	Actinobacte	Unclassified	OTU_3321	0.005	0.003	0.000	0.000	0.007
Bacteria	candidate di	WPS-1_gen	OTU_4134	0.005	0.005	0.000	0.000	0.031
Bacteria	Proteobacte	Unclassified	OTU_6843	0.005	0.005	0.000	0.000	0.034
Bacteria	Chloroflexi	Ornatilinea	OTU_3850	0.005	0.004	0.000	0.000	0.029
Bacteria	Planctomyce	Unclassified	OTU_3856	0.006	0.005	0.001	0.002	0.046
Bacteria	Actinobacte	Solirubrobac	OTU_7926	0.005	0.004	0.000	0.000	0.014
Bacteria	Chloroflexi	Unclassified	OTU_2173	0.005	0.003	0.001	0.001	0.003
Bacteria	Unclassified	Unclassified	OTU_3540	0.007	0.004	0.002	0.003	0.032
Bacteria	Armatimona	Armatimona	OTU_1951	0.007	0.004	0.002	0.003	0.041
Bacteria	Latescibacte	Latescibacte	OTU_4546	0.005	0.005	0.001	0.001	0.046
Bacteria	Planctomyce	Unclassified	OTU_7472	0.004	0.005	0.000	0.000	0.043
Bacteria	Planctomyce	Unclassified	OTU_2312	0.007	0.004	0.003	0.001	0.015
Bacteria	Proteobacte	Unclassified	OTU_1715	0.005	0.005	0.000	0.001	0.042
Bacteria	Acidobacter	Gp10	OTU_4730	0.004	0.004	0.000	0.000	0.031
Bacteria	Bacteroidete	Terrimonas	OTU_6910	0.004	0.005	0.000	0.000	0.042
Bacteria	Armatimona	Armatimona	OTU_4155	0.005	0.004	0.001	0.002	0.044
Bacteria	Gemmatimo	Gemmatimo	OTU_1736	0.004	0.004	0.000	0.000	0.038
Bacteria	Gemmatimo	Gemmatimo	OTU_2871	0.004	0.004	0.000	0.000	0.026
Bacteria	candidate di	WPS-1_gen	OTU_4611	0.004	0.003	0.000	0.000	0.013
Bacteria	Actinobacte	Unclassified	OTU_5208	0.004	0.004	0.000	0.000	0.024
Bacteria	Gemmatimo	Gemmatimo	OTU_2111	0.004	0.004	0.000	0.001	0.023
Bacteria	Planctomyce	Blastopirell	OTU_6162	0.005	0.004	0.001	0.001	0.033
Bacteria	Unclassified	Unclassified	OTU_4642	0.004	0.004	0.000	0.000	0.048

Bacteria	Proteobacter	Unclassified	OTU_3405	0.004	0.004	0.000	0.000	0.045
Bacteria	Proteobacter	Unclassified	OTU_4898	0.004	0.004	0.000	0.000	0.020
Bacteria	Bacteroidete	Unclassified	OTU_7622	0.004	0.004	0.000	0.000	0.038
Bacteria	Bacteroidete	Terrimonas	OTU_3283	0.004	0.003	0.000	0.000	0.005
Bacteria	Acidobacter	Gp6	OTU_7291	0.004	0.004	0.000	0.000	0.045
Bacteria	Actinobacte	Gaiella	OTU_6209	0.006	0.003	0.002	0.002	0.014
Bacteria	Chloroflexi	Litorilinea	OTU_5391	0.004	0.003	0.000	0.000	0.021
Bacteria	Planctomyce	Unclassified	OTU_3697	0.004	0.003	0.000	0.000	0.019
Bacteria	Planctomyce	Thermogutta	OTU_4149	0.004	0.004	0.000	0.000	0.038
Bacteria	Proteobacter	Unclassified	OTU_2820	0.004	0.004	0.000	0.000	0.047
Bacteria	Acidobacter	Gp4	OTU_5736	0.004	0.004	0.000	0.000	0.050
Bacteria	Bacteroidete	Ohtaekwang	OTU_1120	0.003	0.002	0.000	0.000	0.002
Bacteria	Actinobacte	Unclassified	OTU_7326	0.003	0.002	0.000	0.000	0.004
Bacteria	Actinobacte	Unclassified	OTU_2685	0.004	0.004	0.000	0.001	0.047
Bacteria	candidate di	WPS-1_gen	OTU_3121	0.004	0.004	0.001	0.001	0.049
Bacteria	Actinobacte	Actinomyce	OTU_2063	0.006	0.002	0.003	0.002	0.017
Bacteria	Unclassified	Unclassified	OTU_7617	0.003	0.003	0.000	0.000	0.029
Bacteria	Acidobacter	Gp5	OTU_2586	0.004	0.004	0.001	0.001	0.050
Bacteria	Armatimona	Chthonomor	OTU_2731	0.003	0.002	0.000	0.000	0.005
Bacteria	Acidobacter	Gp6	OTU_2495	0.003	0.003	0.000	0.000	0.036
Bacteria	Proteobacter	Unclassified	OTU_6078	0.003	0.004	0.000	0.000	0.047
Bacteria	Actinobacte	Ilumatobacte	OTU_7858	0.004	0.004	0.000	0.001	0.048
Bacteria	Verrucomic	Luteolibacte	OTU_3787	0.003	0.003	0.000	0.000	0.035
Bacteria	Proteobacter	Unclassified	OTU_3281	0.003	0.003	0.000	0.000	0.012
Bacteria	Armatimona	Chthonomor	OTU_4176	0.003	0.003	0.000	0.000	0.038
Bacteria	Planctomyce	Unclassified	OTU_3721	0.003	0.003	0.000	0.000	0.019
Bacteria	Acidobacter	Gp6	OTU_5299	0.003	0.003	0.000	0.000	0.012
Bacteria	Planctomyce	Zavarzinella	OTU_6146	0.003	0.004	0.000	0.000	0.046
Bacteria	Proteobacter	Unclassified	OTU_2472	0.004	0.003	0.001	0.001	0.041
Bacteria	Proteobacter	Unclassified	OTU_1323	0.005	0.003	0.002	0.002	0.042
Bacteria	Proteobacter	Sandaracinu	OTU_6792	0.003	0.003	0.000	0.000	0.025
Bacteria	Bacteroidete	Unclassified	OTU_4673	0.003	0.003	0.000	0.000	0.048
Bacteria	Acidobacter	Gp3	OTU_2324	0.003	0.003	0.000	0.000	0.048
Bacteria	Chloroflexi	Unclassified	OTU_5913	0.003	0.003	0.000	0.000	0.036
Bacteria	Verrucomic	Subdivision3	OTU_4741	0.003	0.003	0.000	0.000	0.042
Bacteria	Verrucomic	Unclassified	OTU_9266	0.003	0.003	0.000	0.000	0.021
Bacteria	Proteobacter	Unclassified	OTU_2340	0.003	0.002	0.001	0.001	0.018
Bacteria	Acidobacter	Gp6	OTU_6021	0.003	0.003	0.000	0.000	0.045
Bacteria	Acidobacter	Gp4	OTU_2370	0.003	0.003	0.000	0.000	0.033
Bacteria	Unclassified	Unclassified	OTU_6450	0.003	0.003	0.000	0.000	0.042
Bacteria	Proteobacter	Unclassified	OTU_8374	0.003	0.003	0.000	0.000	0.041
Bacteria	Actinobacte	Aciditerrimo	OTU_3900	0.003	0.003	0.000	0.000	0.032
Bacteria	Unclassified	Unclassified	OTU_3566	0.002	0.002	0.000	0.000	0.024
Bacteria	Planctomyce	Unclassified	OTU_5593	0.002	0.003	0.000	0.000	0.038
Bacteria	Proteobacter	Unclassified	OTU_4547	0.002	0.003	0.000	0.000	0.046
Bacteria	Proteobacter	Unclassified	OTU_5971	0.002	0.002	0.000	0.000	0.036
Bacteria	Verrucomic	Unclassified	OTU_6359	0.003	0.002	0.000	0.001	0.030
Bacteria	Chloroflexi	Oscillochlor	OTU_2519	0.002	0.003	0.000	0.000	0.049
Bacteria	Acidobacter	Gp16	OTU_5152	0.002	0.002	0.000	0.000	0.024
Bacteria	Armatimona	Armatimona	OTU_6140	0.002	0.002	0.000	0.000	0.028
Bacteria	Chloroflexi	Unclassified	OTU_2403	0.002	0.003	0.000	0.000	0.049
Bacteria	Unclassified	Unclassified	OTU_8341	0.002	0.003	0.000	0.000	0.044
Bacteria	Proteobacter	Unclassified	OTU_8681	0.002	0.003	0.000	0.000	0.047
Bacteria	Unclassified	Unclassified	OTU_3300	0.002	0.002	0.000	0.000	0.036
Bacteria	Planctomyce	Zavarzinella	OTU_3782	0.002	0.002	0.000	0.000	0.043
Bacteria	Proteobacter	Hephaestia	OTU_6054	0.002	0.002	0.000	0.000	0.050
Bacteria	Unclassified	Unclassified	OTU_3979	0.002	0.002	0.000	0.000	0.050
Bacteria	Armatimona	Armatimona	OTU_4794	0.002	0.002	0.000	0.000	0.028
Bacteria	Latescibacte	Latescibacte	OTU_6469	0.002	0.002	0.000	0.000	0.029
Bacteria	Unclassified	Unclassified	OTU_3417	0.002	0.002	0.000	0.000	0.048
Bacteria	Verrucomic	Subdivision3	OTU_5519	0.002	0.002	0.000	0.000	0.033
Bacteria	Chloroflexi	Litorilinea	OTU_4531	0.002	0.002	0.000	0.000	0.049
Bacteria	Unclassified	Unclassified	OTU_3164	0.002	0.002	0.000	0.000	0.043
Bacteria	Gemmatimo	Gemmatimo	OTU_2314	0.002	0.002	0.000	0.000	0.039
Bacteria	Verrucomic	Subdivision3	OTU_7595	0.002	0.002	0.000	0.000	0.039

Bacteria	Verrucomicr	Subdivision3	OTU_9149	0.002	0.002	0.000	0.000	0.024
Bacteria	Proteobacter	Unclassified	OTU_4290	0.002	0.002	0.000	0.000	0.024
Bacteria	Unclassified	Unclassified	OTU_3239	0.002	0.002	0.000	0.000	0.045
Bacteria	Proteobacter	Unclassified	OTU_2148	0.002	0.002	0.000	0.000	0.041
Bacteria	Bacteroidete	Unclassified	OTU_1554	0.002	0.002	0.000	0.000	0.048
Bacteria	Bacteroidete	Taibaiella	OTU_7937	0.000	0.000	0.001	0.001	0.043
Archaea	Euryarchaeo	Methanosarc	OTU_3774	0.000	0.000	0.001	0.001	0.049
Bacteria	Planctomyce	Gemmata	OTU_9224	0.000	0.000	0.001	0.001	0.041
Bacteria	Unclassified	Unclassified	OTU_8804	0.000	0.000	0.001	0.001	0.041
Bacteria	Firmicutes	Unclassified	OTU_4228	0.000	0.000	0.001	0.001	0.041
Bacteria	Unclassified	Unclassified	OTU_3185	0.000	0.000	0.001	0.001	0.036
Bacteria	Proteobacter	Unclassified	OTU_1978	0.000	0.000	0.001	0.001	0.036
Bacteria	Bacteroidete	Dyadobacter	OTU_5159	0.000	0.000	0.001	0.001	0.042
Bacteria	Planctomyce	Planctopirus	OTU_5068	0.000	0.000	0.001	0.001	0.034
Bacteria	Unclassified	Unclassified	OTU_5607	0.000	0.000	0.001	0.001	0.033
Bacteria	Firmicutes	Sporomusa	OTU_5740	0.000	0.000	0.001	0.001	0.041
Bacteria	Planctomyce	Unclassified	OTU_2876	0.000	0.000	0.001	0.001	0.019
Bacteria	Cyanobacter	Bacillarioph	OTU_7604	0.000	0.000	0.001	0.001	0.042
Bacteria	Proteobacter	Phaselicystis	OTU_7772	0.000	0.000	0.001	0.001	0.040
Bacteria	Proteobacter	Unclassified	OTU_4916	0.000	0.000	0.001	0.001	0.040
Bacteria	Unclassified	Unclassified	OTU_3270	0.000	0.000	0.001	0.001	0.039
Bacteria	Planctomyce	Singulisphae	OTU_2702	0.000	0.000	0.001	0.001	0.012
Bacteria	Unclassified	Unclassified	OTU_5339	0.000	0.000	0.001	0.001	0.039
Bacteria	Unclassified	Unclassified	OTU_4013	0.000	0.000	0.001	0.001	0.023
Bacteria	Verrucomicr	Subdivision3	OTU_2700	0.000	0.000	0.001	0.001	0.045
Bacteria	Proteobacter	Steroidobact	OTU_6196	0.000	0.000	0.001	0.001	0.047
Bacteria	Planctomyce	Singulisphae	OTU_3550	0.000	0.000	0.001	0.001	0.047
Bacteria	Firmicutes	Leuconostoc	OTU_5998	0.000	0.000	0.001	0.001	0.044
Bacteria	Proteobacter	Methylococc	OTU_4880	0.000	0.000	0.001	0.002	0.042
Bacteria	Proteobacter	Unclassified	OTU_8033	0.000	0.000	0.001	0.002	0.047
Bacteria	Planctomyce	Unclassified	OTU_7424	0.000	0.000	0.001	0.001	0.032
Bacteria	Firmicutes	Romboutsia	OTU_1849	0.000	0.000	0.002	0.002	0.046
Bacteria	Cyanobacter	Bacillarioph	OTU_5026	0.000	0.000	0.002	0.002	0.046
Bacteria	Unclassified	Unclassified	OTU_5433	0.000	0.000	0.002	0.001	0.018
Bacteria	Chlamydiae	Unclassified	OTU_3656	0.000	0.000	0.002	0.001	0.015
Bacteria	Proteobacter	Legionella	OTU_5465	0.000	0.000	0.002	0.002	0.048
Bacteria	Proteobacter	Unclassified	OTU_3593	0.000	0.000	0.002	0.002	0.042
Bacteria	Candidatus S	Saccharibact	OTU_3030	0.000	0.000	0.002	0.002	0.043
Bacteria	Planctomyce	Zavarzinella	OTU_2360	0.000	0.000	0.002	0.002	0.019
Bacteria	Bacteroidete	Taibaiella	OTU_7519	0.000	0.000	0.002	0.001	0.017
Bacteria	Planctomyce	Zavarzinella	OTU_3171	0.000	0.000	0.002	0.002	0.031
Bacteria	Firmicutes	Clostridium	OTU_4020	0.000	0.000	0.002	0.002	0.039
Bacteria	Firmicutes	Clostridium	OTU_2716	0.000	0.000	0.002	0.002	0.039
Bacteria	Actinobacter	Unclassified	OTU_2865	0.000	0.000	0.002	0.002	0.025
Bacteria	Actinobacter	Conexibacte	OTU_3337	0.000	0.000	0.002	0.002	0.040
Bacteria	Planctomyce	Aquisphaera	OTU_3407	0.000	0.000	0.002	0.002	0.023
Bacteria	Proteobacter	Unclassified	OTU_4058	0.000	0.000	0.002	0.002	0.042
Bacteria	Acidobacter	Gp3	OTU_2421	0.000	0.000	0.002	0.002	0.037
Bacteria	Acidobacter	Gp3	OTU_5531	0.000	0.000	0.002	0.002	0.031
Bacteria	Chloroflexi	Unclassified	OTU_8365	0.000	0.000	0.002	0.002	0.037
Bacteria	Proteobacter	Aquicella	OTU_6118	0.000	0.000	0.002	0.002	0.033
Bacteria	Planctomyce	Singulisphae	OTU_3225	0.000	0.000	0.002	0.002	0.037
Bacteria	Proteobacter	Unclassified	OTU_2455	0.000	0.000	0.002	0.003	0.042
Bacteria	Proteobacter	Unclassified	OTU_1879	0.000	0.000	0.002	0.002	0.030
Bacteria	Candidatus S	Saccharibact	OTU_4104	0.000	0.000	0.002	0.002	0.024
Bacteria	Proteobacter	Bdellovibrio	OTU_3670	0.000	0.000	0.003	0.003	0.047
Bacteria	Bacteroidete	Unclassified	OTU_7471	0.000	0.000	0.003	0.003	0.047
Bacteria	Unclassified	Unclassified	OTU_3236	0.000	0.000	0.003	0.003	0.047
Bacteria	Proteobacter	Unclassified	OTU_7467	0.000	0.000	0.003	0.002	0.008
Bacteria	Proteobacter	Nitrospirillu	OTU_7835	0.000	0.000	0.003	0.003	0.040
Bacteria	Planctomyce	Gemmata	OTU_1575	0.000	0.000	0.003	0.003	0.031
Bacteria	Proteobacter	Unclassified	OTU_2997	0.000	0.000	0.003	0.002	0.019
Bacteria	Bacteroidete	Unclassified	OTU_2247	0.000	0.000	0.003	0.003	0.048
Bacteria	Acidobacter	Gp15	OTU_2726	0.000	0.000	0.003	0.003	0.035
Bacteria	Unclassified	Unclassified	OTU_3803	0.000	0.000	0.003	0.003	0.030

Bacteria	Parcubacteri	Parcubacteri	OTU_2096	0.000	0.000	0.003	0.003	0.034
Bacteria	Proteobacter	Unclassified	OTU_1742	0.000	0.000	0.003	0.003	0.046
Bacteria	Unclassified	Unclassified	OTU_1491	0.000	0.000	0.003	0.003	0.021
Bacteria	Proteobacter	Unclassified	OTU_2535	0.000	0.000	0.003	0.003	0.049
Bacteria	Proteobacter	Unclassified	OTU_1595	0.000	0.000	0.003	0.002	0.010
Bacteria	Proteobacter	Unclassified	OTU_1036	0.000	0.000	0.003	0.003	0.050
Bacteria	Proteobacter	Unclassified	OTU_3915	0.000	0.000	0.003	0.003	0.040
Bacteria	Armatimona	Armatimona	OTU_3690	0.000	0.000	0.003	0.003	0.032
Bacteria	Unclassified	Unclassified	OTU_1814	0.000	0.000	0.003	0.003	0.042
Bacteria	Verrucomicr	Subdivision3	OTU_7076	0.000	0.000	0.003	0.003	0.035
Bacteria	Bacteroidete	Sphingobact	OTU_1294	0.000	0.000	0.003	0.003	0.035
Bacteria	Proteobacter	Aquicella	OTU_7342	0.000	0.000	0.003	0.003	0.015
Bacteria	Proteobacter	Unclassified	OTU_3673	0.000	0.000	0.003	0.003	0.030
Bacteria	Armatimona	Armatimona	OTU_1395	0.000	0.000	0.003	0.003	0.020
Bacteria	Firmicutes	Unclassified	OTU_2066	0.000	0.000	0.003	0.004	0.049
Bacteria	Spirochaetes	Spirochaeta	OTU_5225	0.000	0.000	0.003	0.004	0.038
Bacteria	Planctomyce	Schlesneria	OTU_4464	0.000	0.000	0.003	0.004	0.038
Bacteria	Verrucomicr	Spartobacter	OTU_5659	0.000	0.000	0.003	0.003	0.025
Bacteria	Proteobacter	Legionella	OTU_3760	0.000	0.000	0.003	0.003	0.021
Bacteria	Acidobacter	Gp18	OTU_5033	0.000	0.000	0.004	0.003	0.027
Bacteria	Acidobacter	Acidipila	OTU_2350	0.000	0.000	0.004	0.004	0.035
Bacteria	Chlamydiae	Simkania	OTU_4297	0.000	0.000	0.004	0.003	0.019
Bacteria	Proteobacter	Unclassified	OTU_3169	0.000	0.000	0.004	0.003	0.008
Bacteria	Acidobacter	Gp3	OTU_1925	0.000	0.000	0.004	0.004	0.039
Bacteria	Unclassified	Unclassified	OTU_3172	0.000	0.000	0.004	0.003	0.014
Bacteria	Actinobacter	Unclassified	OTU_1685	0.001	0.002	0.005	0.004	0.045
Bacteria	Armatimona	Chthonomor	OTU_2887	0.000	0.000	0.004	0.003	0.018
Bacteria	Proteobacter	Unclassified	OTU_3021	0.000	0.000	0.004	0.003	0.004
Bacteria	Unclassified	Unclassified	OTU_1730	0.000	0.000	0.004	0.004	0.028
Bacteria	candidate di	WPS-2_gen	OTU_3757	0.000	0.000	0.004	0.003	0.015
Bacteria	Proteobacter	Anaeromyxo	OTU_5058	0.000	0.000	0.004	0.003	0.004
Bacteria	Unclassified	Unclassified	OTU_1780	0.000	0.000	0.004	0.004	0.034
Bacteria	Firmicutes	Clostridium	OTU_5192	0.000	0.000	0.004	0.003	0.009
Bacteria	Proteobacter	Unclassified	OTU_2367	0.001	0.002	0.006	0.004	0.015
Bacteria	Proteobacter	Unclassified	OTU_3994	0.000	0.000	0.004	0.004	0.014
Bacteria	Candidatus S	Saccharibact	OTU_4005	0.000	0.000	0.004	0.005	0.046
Bacteria	Acidobacter	Gp3	OTU_8879	0.000	0.000	0.004	0.003	0.010
Bacteria	Unclassified	Unclassified	OTU_5937	0.000	0.000	0.005	0.005	0.029
Bacteria	Proteobacter	Unclassified	OTU_2068	0.000	0.000	0.005	0.005	0.020
Bacteria	Chloroflexi	Ktedonobact	OTU_2516	0.000	0.000	0.006	0.005	0.023
Bacteria	Acidobacter	Gp1	OTU_1635	0.000	0.000	0.006	0.006	0.035
Bacteria	Acidobacter	Candidatus I	OTU_629	0.000	0.000	0.006	0.006	0.036
Bacteria	Gemmatimo	Gemmatimo	OTU_701	0.000	0.000	0.006	0.007	0.044
Bacteria	Bacteroidete	Unclassified	OTU_1841	0.002	0.003	0.009	0.007	0.041
Bacteria	candidate di	WPS-2_gen	OTU_1523	0.000	0.000	0.007	0.006	0.021
Bacteria	Proteobacter	Unclassified	OTU_2199	0.000	0.000	0.007	0.008	0.047
Bacteria	Gemmatimo	Gemmatimo	OTU_708	0.000	0.000	0.008	0.006	0.011
Bacteria	Unclassified	Unclassified	OTU_2358	0.000	0.000	0.008	0.008	0.026
Bacteria	Candidatus S	Saccharibact	OTU_1757	0.000	0.000	0.008	0.009	0.043
Bacteria	Acidobacter	Acidipila	OTU_2415	0.000	0.000	0.009	0.006	0.006
Bacteria	Proteobacter	Acidisoma	OTU_5390	0.010	0.009	0.020	0.006	0.032
Bacteria	Proteobacter	Brevundimo	OTU_690	0.000	0.000	0.010	0.009	0.022
Bacteria	Proteobacter	Unclassified	OTU_1412	0.001	0.002	0.012	0.007	0.004
Bacteria	Proteobacter	Unclassified	OTU_680	0.002	0.002	0.012	0.011	0.036
Bacteria	Bacteroidete	Mucilaginiba	OTU_936	0.000	0.000	0.011	0.007	0.005
Bacteria	Planctomyce	Gemmata	OTU_660	0.003	0.003	0.014	0.010	0.026
Bacteria	Actinobacter	Unclassified	OTU_1980	0.000	0.000	0.011	0.010	0.019
Bacteria	Acidobacter	Gp6	OTU_700	0.000	0.000	0.012	0.010	0.021
Bacteria	Proteobacter	Unclassified	OTU_853	0.001	0.002	0.013	0.009	0.009
Bacteria	Acidobacter	Gp1	OTU_1367	0.000	0.000	0.013	0.015	0.046
Bacteria	Verrucomicr	Subdivision3	OTU_932	0.006	0.009	0.020	0.013	0.040
Bacteria	Proteobacter	Unclassified	OTU_1181	0.001	0.002	0.015	0.013	0.027
Bacteria	Unclassified	Unclassified	OTU_1322	0.004	0.004	0.018	0.015	0.044
Bacteria	Planctomyce	Gemmata	OTU_887	0.000	0.000	0.014	0.014	0.029
Bacteria	Proteobacter	Azospirillum	OTU_620	0.002	0.003	0.016	0.010	0.008

Bacteria	Proteobacter	Phenylobact	OTU_5860	0.010	0.012	0.024	0.013	0.044
Bacteria	Bacteroidete	Unclassified	OTU_1449	0.004	0.007	0.019	0.016	0.044
Bacteria	Acidobacter	Gp14	OTU_232	0.000	0.000	0.015	0.016	0.039
Bacteria	Chloroflexi	Unclassified	OTU_872	0.004	0.003	0.019	0.014	0.026
Bacteria	Acidobacter	Acidipila	OTU_336	0.010	0.013	0.029	0.011	0.008
Bacteria	Proteobacter	Lacibacteriu	OTU_323	0.005	0.006	0.026	0.012	0.002
Bacteria	Acidobacter	Unclassified	OTU_4823	0.006	0.006	0.027	0.020	0.026
Bacteria	Proteobacter	Reyranella	OTU_451	0.008	0.007	0.030	0.024	0.048
Bacteria	Proteobacter	Unclassified	OTU_616	0.006	0.007	0.029	0.015	0.004
Bacteria	Gemmatimo	Gemmatimo	OTU_531	0.007	0.007	0.030	0.024	0.037
Bacteria	Bacteroidete	Unclassified	OTU_313	0.002	0.002	0.025	0.018	0.010
Bacteria	Bacteroidete	Pedobacter	OTU_1339	0.005	0.005	0.029	0.026	0.044
Bacteria	Unclassified	Unclassified	OTU_669	0.000	0.000	0.026	0.027	0.042
Bacteria	Chloroflexi	Unclassified	OTU_1486	0.007	0.010	0.032	0.024	0.025
Bacteria	Proteobacter	Unclassified	OTU_343	0.001	0.001	0.027	0.027	0.038
Bacteria	Chloroflexi	Thermomari	OTU_992	0.000	0.000	0.027	0.030	0.049
Bacteria	Acidobacter	Unclassified	OTU_478	0.014	0.010	0.041	0.024	0.022
Bacteria	Proteobacter	Unclassified	OTU_390	0.016	0.016	0.044	0.024	0.023
Bacteria	Proteobacter	Unclassified	OTU_351	0.004	0.006	0.034	0.026	0.018
Bacteria	Unclassified	Unclassified	OTU_181	0.008	0.014	0.039	0.031	0.037
Bacteria	Planctomyce	Singulisphae	OTU_406	0.012	0.011	0.048	0.034	0.025
Bacteria	Proteobacter	Pseudoxanth	OTU_145	0.010	0.011	0.047	0.040	0.048
Bacteria	Acidobacter	Granulicella	OTU_274	0.009	0.006	0.046	0.031	0.015
Bacteria	Unclassified	Unclassified	OTU_290	0.003	0.006	0.045	0.047	0.050
Bacteria	Acidobacter	Gp3	OTU_370	0.004	0.004	0.046	0.043	0.036
Bacteria	Proteobacter	Unclassified	OTU_549	0.010	0.012	0.052	0.040	0.029
Bacteria	Chloroflexi	Nitrolancea	OTU_357	0.009	0.008	0.053	0.048	0.047
Bacteria	Actinobacter	Unclassified	OTU_243	0.010	0.008	0.055	0.048	0.043
Bacteria	Chloroflexi	Ktedonobact	OTU_929	0.008	0.010	0.054	0.046	0.034
Bacteria	Acidobacter	Acidobacter	OTU_318	0.007	0.004	0.057	0.051	0.036
Bacteria	Bacteroidete	Unclassified	OTU_644	0.015	0.022	0.065	0.043	0.019
Bacteria	Actinobacter	Acidothermu	OTU_360	0.008	0.009	0.058	0.049	0.028
Bacteria	Proteobacter	Unclassified	OTU_386	0.022	0.009	0.073	0.047	0.023
Bacteria	Acidobacter	Candidatus S	OTU_365	0.043	0.038	0.095	0.051	0.047
Bacteria	Proteobacter	Unclassified	OTU_157	0.034	0.015	0.087	0.052	0.031
Bacteria	Planctomyce	Planctopirus	OTU_214	0.031	0.030	0.085	0.041	0.015
Bacteria	Proteobacter	Lacibacteriu	OTU_156	0.004	0.005	0.060	0.050	0.021
Bacteria	Actinobacter	Acidimicrob	OTU_569	0.005	0.005	0.062	0.050	0.019
Bacteria	Actinobacter	Unclassified	OTU_671	0.036	0.025	0.094	0.054	0.028
Bacteria	Chloroflexi	Unclassified	OTU_53	0.027	0.026	0.087	0.035	0.003
Bacteria	Proteobacter	Unclassified	OTU_367	0.012	0.013	0.076	0.057	0.020
Bacteria	Proteobacter	Kerstersia	OTU_259	0.002	0.004	0.067	0.044	0.006
Bacteria	Acidobacter	Terriglobus	OTU_8499	0.009	0.008	0.076	0.063	0.026
Bacteria	Acidobacter	Granulicella	OTU_140	0.055	0.032	0.132	0.059	0.012
Bacteria	Proteobacter	Skermanella	OTU_129	0.008	0.010	0.087	0.070	0.020
Bacteria	Actinobacter	Unclassified	OTU_7479	0.043	0.041	0.131	0.091	0.043
Bacteria	Proteobacter	Unclassified	OTU_738	0.076	0.048	0.167	0.077	0.020
Bacteria	Proteobacter	Tahibacter	OTU_114	0.017	0.015	0.117	0.086	0.018
Bacteria	Proteobacter	Pseudolabry	OTU_52	0.108	0.067	0.216	0.104	0.041
Bacteria	Bacteroidete	Unclassified	OTU_85	0.028	0.026	0.154	0.089	0.007
Bacteria	Actinobacter	Gaiella	OTU_206	0.082	0.062	0.209	0.107	0.019
Bacteria	Actinobacter	Gaiella	OTU_275	0.091	0.057	0.219	0.126	0.034
Bacteria	Planctomyce	Thermogutta	OTU_187	0.035	0.032	0.165	0.099	0.010
Bacteria	Proteobacter	Rhizomicrob	OTU_164	0.112	0.087	0.248	0.101	0.018
Bacteria	Chloroflexi	Unclassified	OTU_57	0.048	0.054	0.198	0.121	0.014
Bacteria	Acidobacter	Terriglobus	OTU_188	0.029	0.019	0.181	0.105	0.006
Bacteria	Proteobacter	Unclassified	OTU_151	0.010	0.015	0.172	0.104	0.004
Bacteria	Acidobacter	Unclassified	OTU_91	0.117	0.101	0.302	0.127	0.010
Bacteria	Acidobacter	Gp1	OTU_61	0.017	0.022	0.243	0.198	0.019
Bacteria	Actinobacter	Unclassified	OTU_51	0.103	0.094	0.335	0.215	0.026
Bacteria	Unclassified	Unclassified	OTU_29	0.136	0.120	0.370	0.223	0.033
Bacteria	Proteobacter	Unclassified	OTU_9131	0.056	0.066	0.296	0.172	0.007
Bacteria	Chloroflexi	Unclassified	OTU_59	0.041	0.052	0.313	0.183	0.005
Bacteria	Proteobacter	Unclassified	OTU_5473	0.057	0.068	0.333	0.232	0.016
Bacteria	candidate di	WPS-1_gen	OTU_11	0.122	0.099	0.506	0.397	0.039

Bacteria	Unclassified	Unclassified	OTU_15	0.062	0.036	0.448	0.283	0.009
Bacteria	Proteobacter	Burkholderia	OTU_28	0.136	0.090	0.529	0.311	0.012
Bacteria	Acidobacter	Geothrix	OTU_18	0.132	0.070	0.552	0.409	0.030
Bacteria	Proteobacter	Unclassified	OTU_12	0.275	0.167	0.763	0.458	0.027
Bacteria	Proteobacter	Unclassified	OTU_27	0.095	0.065	0.663	0.444	0.012
Bacteria	Actinobacter	Unclassified	OTU_10	0.629	0.176	1.227	0.625	0.040
Bacteria	Proteobacter	Unclassified	OTU_4	0.234	0.263	1.540	1.396	0.043
Archaea	Thaumarcha	Nitrososphae	OTU_2	0.296	0.250	1.987	1.454	0.018
