# Supplementary material for: Microbial interactions and metabolisms in response to bacterial wilt and black shank pathogens in the tobacco rhizosphere
Source: Front Plant Sci. 2023 Jun 20;14:1200136. doi: 10.3389/fpls.2023.1200136 (PMC10319149; doi:10.3389/fpls.2023.1200136)
Supplement: Supplementary file 5 [file DataSheet_5.pdf]

Additional file 9

Table S9 Genera with significant differences between CK and BSH groups (Unit: %)

Domain	Phylum	Genus	OTU	CK: mean ı	CK: std. de	BSH: mean	BSH: std. c	p-values
Bacteria	Actinobact	Streptomyc	OTU_8	2.532	1.773	0.550	0.525	0.021
Bacteria	Acidobacte	Gp4	OTU_74	1.449	0.811	0.083	0.111	0.003
Bacteria	Actinobact	Streptomyc	OTU_34	1.255	0.588	0.273	0.227	0.003
Bacteria	Proteobacte	Sphingobiu	OTU_6	0.782	0.557	0.039	0.037	0.010
Bacteria	Actinobact	Streptomyc	OTU_3189	0.730	0.399	0.113	0.084	0.004
Bacteria	Verrucomic	Spartobacte	OTU_71	0.705	0.366	0.235	0.134	0.011
Bacteria	Actinobact	Arthrobacte	OTU_17	0.540	0.313	0.149	0.094	0.013
Bacteria	Acidobacte	Gp6	OTU_155	0.422	0.177	0.044	0.033	0.001
Bacteria	Actinobact	Amycolato	OTU_16	0.545	0.247	0.176	0.208	0.009
Bacteria	Actinobact	Janibacter	OTU_42	0.545	0.303	0.184	0.143	0.017
Bacteria	Proteobacte	Unclassifie	OTU_4511	0.319	0.146	0.051	0.030	0.002
Bacteria	Actinobact	Marmorico	OTU_2959	0.404	0.151	0.141	0.142	0.005
Bacteria	Acidobacte	Gp6	OTU_201	0.261	0.167	0.007	0.007	0.005
Bacteria	Acidobacte	Gp6	OTU_70	0.285	0.142	0.034	0.023	0.002
Bacteria	Acidobacte	Gp16	OTU_77	0.306	0.124	0.064	0.060	0.001
Bacteria	Proteobacte	Rhizobium	OTU_43	0.358	0.156	0.126	0.074	0.005
Bacteria	Proteobacte	Sphingobiu	OTU_38	0.235	0.100	0.007	0.013	0.001
Bacteria	Proteobacte	Ramlibacte	OTU_48	0.376	0.124	0.158	0.081	0.002
Bacteria	Acidobacte	Gp6	OTU_118	0.235	0.090	0.030	0.021	0.000
Bacteria	Acidobacte	Gp6	OTU_332	0.216	0.175	0.012	0.011	0.017
Bacteria	Acidobacte	Gp4	OTU_285	0.222	0.103	0.021	0.021	0.001
Bacteria	Actinobact	Lechevalie	OTU_93	0.217	0.145	0.020	0.016	0.009
Bacteria	Proteobacte	Sphingomo	OTU_86	0.344	0.150	0.148	0.050	0.010
Bacteria	Proteobacte	Unclassifie	OTU_134	0.216	0.136	0.023	0.018	0.007
Bacteria	Acidobacte	Gp6	OTU_8332	0.215	0.065	0.030	0.017	0.000
Bacteria	Proteobacte	Georgfuchs	OTU_277	0.229	0.113	0.047	0.061	0.003
Bacteria	Actinobact	Kribbella	OTU_96	0.214	0.071	0.039	0.037	0.000
Bacteria	Unclassifie	Unclassifie	OTU_132	0.224	0.144	0.049	0.054	0.014
Bacteria	Proteobacte	Unclassifie	OTU_202	0.174	0.106	0.004	0.005	0.004
Bacteria	Proteobacte	Unclassifie	OTU_106	0.232	0.120	0.064	0.080	0.009
Bacteria	Verrucomic	Subdivision	OTU_207	0.167	0.147	0.002	0.004	0.021
Bacteria	Proteobacte	Unclassifie	OTU_162	0.306	0.095	0.143	0.067	0.003
Bacteria	Acidobacte	Gp4	OTU_1411	0.170	0.051	0.010	0.006	0.000
Bacteria	candidate d	WPS-1_ge	OTU_94	0.239	0.160	0.084	0.058	0.040
Bacteria	Actinobact	Gaiella	OTU_289	0.245	0.145	0.094	0.065	0.032
Bacteria	Candidatus	Saccharibac	OTU_76	0.174	0.145	0.024	0.053	0.030
Bacteria	Actinobact	Unclassifie	OTU_249	0.199	0.060	0.049	0.064	0.000
Bacteria	Acidobacte	Gp7	OTU_2456	0.186	0.089	0.037	0.054	0.003
Bacteria	Acidobacte	Gp4	OTU_295	0.168	0.128	0.020	0.032	0.018
Bacteria	Bacteroidet	Terrimonas	OTU_192	0.149	0.158	0.003	0.003	0.045
Bacteria	Acidobacte	Gp6	OTU_508	0.157	0.123	0.014	0.013	0.018
Bacteria	Gemmatim	Gemmatim	OTU_46	0.180	0.137	0.039	0.057	0.033
Bacteria	Acidobacte	Gp4	OTU_3764	0.146	0.099	0.005	0.011	0.007
Bacteria	Actinobact	Unclassifie	OTU_8280	0.175	0.133	0.035	0.022	0.027
Bacteria	Acidobacte	Gp6	OTU_244	0.146	0.140	0.010	0.014	0.036
Bacteria	Verrucomic	Opitutus	OTU_208	0.160	0.072	0.027	0.031	0.001
Bacteria	Gemmatim	Gemmatim	OTU_198	0.145	0.078	0.016	0.019	0.003
Bacteria	Proteobacte	Phenylobac	OTU_41	0.276	0.135	0.148	0.071	0.049
Bacteria	Proteobacte	Arenimona	OTU_130	0.170	0.067	0.041	0.073	0.004
Bacteria	Proteobacte	Variovorax	OTU_2056	0.151	0.074	0.025	0.008	0.003
Bacteria	Proteobacte	Pelomonas	OTU_239	0.145	0.051	0.022	0.025	0.000
Bacteria	Actinobact	Phycicoccu	OTU_7584	0.150	0.119	0.028	0.016	0.030

Bacteria	Verrucomi	Spartobact	OTU_432	0.154	0.107	0.034	0.035	0.021
Bacteria	Acidobacte	Gp4	OTU_2467	0.123	0.061	0.007	0.011	0.001
Bacteria	Proteobact	Unclassifie	OTU_4767	0.146	0.096	0.031	0.029	0.016
Bacteria	Actinobact	Conexibact	OTU_291	0.164	0.085	0.051	0.073	0.018
Bacteria	Proteobact	Unclassifie	OTU_381	0.146	0.109	0.035	0.044	0.033
Bacteria	Acidobacte	Gp6	OTU_110	0.135	0.075	0.024	0.014	0.005
Bacteria	Unclassifie	Unclassifie	OTU_127	0.129	0.096	0.022	0.031	0.022
Bacteria	Actinobact	Aquihabita	OTU_299	0.133	0.078	0.026	0.029	0.008
Bacteria	Unclassifie	Unclassifie	OTU_150	0.138	0.080	0.033	0.053	0.013
Bacteria	Proteobact	Rhizobium	OTU_179	0.139	0.089	0.034	0.037	0.018
Bacteria	Acidobacte	Gp4	OTU_7758	0.105	0.098	0.002	0.003	0.028
Bacteria	Acidobacte	Gp7	OTU_279	0.183	0.074	0.081	0.049	0.010
Bacteria	Proteobact	Unclassifie	OTU_341	0.155	0.081	0.054	0.041	0.014
Bacteria	candidate d	WPS-1_ge	OTU_464	0.097	0.085	0.001	0.002	0.020
Bacteria	Proteobact	Unclassifie	OTU_469	0.107	0.090	0.012	0.016	0.027
Bacteria	Acidobacte	Gp6	OTU_2748	0.102	0.101	0.010	0.011	0.046
Bacteria	Verrucomi	Spartobact	OTU_522	0.131	0.050	0.040	0.036	0.002
Bacteria	Proteobact	Sphingomo	OTU_75	0.171	0.073	0.079	0.078	0.040
Bacteria	Proteobact	Unclassifie	OTU_618	0.107	0.048	0.016	0.010	0.001
Bacteria	Bacteroide	Flavisoliba	OTU_120	0.145	0.058	0.056	0.019	0.005
Bacteria	Acidobacte	Gp6	OTU_6281	0.106	0.061	0.018	0.017	0.006
Bacteria	Acidobacte	Aridibacte	OTU_407	0.107	0.065	0.019	0.018	0.009
Bacteria	Acidobacte	Gp4	OTU_750	0.093	0.060	0.006	0.011	0.006
Bacteria	Proteobact	Pseudodug	OTU_178	0.124	0.045	0.037	0.034	0.001
Bacteria	Bacteroide	Terrimonas	OTU_544	0.110	0.052	0.023	0.017	0.003
Bacteria	Actinobact	Solirubrob	OTU_497	0.098	0.082	0.013	0.018	0.028
Bacteria	Acidobacte	Gp6	OTU_210	0.086	0.090	0.002	0.003	0.045
Bacteria	Actinobact	Gaiella	OTU_896	0.096	0.091	0.013	0.020	0.047
Bacteria	Acidobacte	Gp2	OTU_8528	0.112	0.064	0.031	0.045	0.017
Bacteria	Actinobact	Blastococc	OTU_115	0.133	0.078	0.052	0.031	0.030
Bacteria	Actinobact	Rhodococc	OTU_40	0.137	0.073	0.057	0.057	0.039
Bacteria	Proteobact	Massilia	OTU_216	0.125	0.065	0.046	0.029	0.015
Bacteria	Acidobacte	Gp6	OTU_3585	0.091	0.051	0.012	0.018	0.004
Bacteria	Proteobact	Unclassifie	OTU_242	0.126	0.033	0.048	0.019	0.000
Bacteria	Verrucomi	Subdivisio	OTU_624	0.117	0.046	0.040	0.040	0.005
Bacteria	Actinobact	Aciditerrin	OTU_638	0.087	0.073	0.011	0.005	0.027
Bacteria	Acidobacte	Gp6	OTU_6956	0.079	0.047	0.005	0.005	0.004
Bacteria	Acidobacte	Gp16	OTU_496	0.099	0.032	0.025	0.019	0.000
Bacteria	Acidobacte	Gp6	OTU_159	0.087	0.041	0.012	0.008	0.002
Bacteria	Verrucomi	Opitutus	OTU_645	0.087	0.054	0.013	0.007	0.008
Bacteria	Acidobacte	Gp4	OTU_8714	0.081	0.060	0.008	0.016	0.014
Bacteria	Verrucomi	Unclassifie	OTU_304	0.080	0.052	0.007	0.008	0.007
Bacteria	Acidobacte	Gp6	OTU_610	0.073	0.078	0.000	0.000	0.043
Bacteria	Proteobact	Minicystis	OTU_402	0.079	0.061	0.007	0.007	0.016
Bacteria	Actinobact	Solirubrob	OTU_657	0.077	0.077	0.005	0.009	0.042
Bacteria	Proteobact	Luteimona	OTU_195	0.098	0.062	0.027	0.021	0.019
Bacteria	Unclassifie	Unclassifie	OTU_1942	0.075	0.064	0.004	0.009	0.023
Bacteria	Proteobact	Unclassifie	OTU_334	0.076	0.071	0.006	0.013	0.036
Bacteria	Actinobact	Conexibact	OTU_211	0.123	0.041	0.053	0.062	0.029
Bacteria	Unclassifie	Unclassifie	OTU_506	0.076	0.056	0.006	0.008	0.014
Bacteria	Acidobacte	Unclassifie	OTU_2086	0.076	0.054	0.007	0.006	0.011
Bacteria	Verrucomi	Subdivisio	OTU_713	0.089	0.066	0.021	0.011	0.028
Bacteria	Acidobacte	Gp6	OTU_1906	0.077	0.022	0.009	0.008	0.000
Bacteria	Verrucomi	Spartobact	OTU_1311	0.087	0.046	0.020	0.013	0.006
Bacteria	Proteobact	Unclassifie	OTU_589	0.097	0.054	0.031	0.015	0.013
Bacteria	Acidobacte	Gp6	OTU_270	0.066	0.065	0.000	0.000	0.030

Bacteria	Proteobacte	Unclassifie	OTU_513	0.072	0.048	0.006	0.007	0.008
Bacteria	Bacteroidet	Niastella	OTU_445	0.094	0.042	0.028	0.033	0.006
Bacteria	Proteobacte	Povalibacte	OTU_697	0.072	0.064	0.009	0.010	0.035
Bacteria	Proteobacte	Haliangium	OTU_3989	0.077	0.025	0.014	0.021	0.000
Bacteria	Acidobacte	Gp4	OTU_2388	0.066	0.055	0.003	0.004	0.019
Bacteria	Actinobact	Gaiella	OTU_1345	0.073	0.048	0.011	0.012	0.011
Bacteria	Gemmatim	Gemmatim	OTU_293	0.088	0.027	0.027	0.017	0.000
Bacteria	Bacteroidet	Parafilimor	OTU_889	0.064	0.058	0.006	0.003	0.031
Bacteria	Acidobacte	Gp4	OTU_8027	0.061	0.044	0.003	0.004	0.009
Bacteria	Proteobacte	Unclassifie	OTU_463	0.079	0.034	0.021	0.017	0.002
Bacteria	Proteobacte	Pseudorhoc	OTU_7191	0.082	0.023	0.024	0.017	0.000
Bacteria	Actinobact	Gaiella	OTU_382	0.069	0.054	0.011	0.015	0.027
Bacteria	Actinobact	Gaiella	OTU_702	0.071	0.034	0.013	0.013	0.002
Bacteria	Acidobacte	Gp6	OTU_4904	0.063	0.053	0.005	0.003	0.024
Bacteria	Gemmatim	Gemmatim	OTU_260	0.066	0.061	0.009	0.006	0.042
Bacteria	Proteobacte	Unclassifie	OTU_7432	0.068	0.035	0.011	0.007	0.003
Bacteria	Actinobact	Dactylospo	OTU_141	0.099	0.044	0.042	0.034	0.018
Bacteria	Actinobact	Unclassifie	OTU_408	0.085	0.051	0.028	0.024	0.023
Bacteria	Acidobacte	Gp6	OTU_6070	0.066	0.054	0.010	0.013	0.029
Bacteria	Actinobact	Unclassifie	OTU_7451	0.092	0.056	0.035	0.019	0.035
Bacteria	Acidobacte	Gp7	OTU_228	0.089	0.056	0.033	0.027	0.038
Bacteria	Acidobacte	Gp4	OTU_1101	0.057	0.029	0.002	0.002	0.002
Bacteria	Candidatus	Saccharibac	OTU_882	0.054	0.052	0.000	0.000	0.027
Bacteria	Acidobacte	Gp6	OTU_7014	0.062	0.036	0.008	0.012	0.005
Bacteria	Actinobact	Gaiella	OTU_366	0.060	0.021	0.006	0.008	0.000
Bacteria	Verrucomi	Subdivision	OTU_268	0.072	0.032	0.019	0.024	0.004
Bacteria	Proteobacte	Altererythr	OTU_308	0.065	0.056	0.011	0.010	0.039
Bacteria	Unclassifie	Unclassifie	OTU_658	0.058	0.037	0.005	0.005	0.007
Bacteria	Actinobact	Gaiella	OTU_335	0.073	0.031	0.020	0.018	0.003
Bacteria	Verrucomi	Spartobacte	OTU_218	0.109	0.031	0.056	0.020	0.003
Bacteria	Proteobacte	Unclassifie	OTU_553	0.058	0.024	0.005	0.006	0.001
Bacteria	Actinobact	Gaiella	OTU_8708	0.062	0.044	0.009	0.011	0.015
Bacteria	candidate d	WPS-1_gei	OTU_479	0.059	0.034	0.006	0.005	0.004
Bacteria	candidate d	WPS-1_gei	OTU_816	0.061	0.035	0.009	0.007	0.005
Bacteria	Acidobacte	Gp7	OTU_2331	0.056	0.039	0.005	0.004	0.010
Bacteria	Proteobacte	Ochrobactr	OTU_3974	0.071	0.050	0.019	0.019	0.032
Bacteria	Actinobact	Ilumatobac	OTU_501	0.054	0.047	0.003	0.003	0.023
Bacteria	Actinobact	Nocardioid	OTU_2656	0.063	0.040	0.012	0.016	0.013
Bacteria	Unclassifie	Unclassifie	OTU_7265	0.052	0.047	0.002	0.004	0.027
Bacteria	Actinobact	Conexibact	OTU_353	0.068	0.033	0.018	0.018	0.006
Bacteria	Verrucomi	Subdivision	OTU_783	0.051	0.036	0.001	0.002	0.009
Bacteria	Acidobacte	Gp6	OTU_1025	0.054	0.038	0.004	0.006	0.011
Bacteria	Actinobact	Pseudonoca	OTU_598	0.079	0.045	0.030	0.015	0.025
Bacteria	Actinobact	Gaiella	OTU_2538	0.051	0.048	0.002	0.005	0.030
Bacteria	Armatimon	Armatimon	OTU_258	0.063	0.025	0.014	0.015	0.001
Bacteria	Actinobact	Solirubroba	OTU_2102	0.060	0.047	0.012	0.016	0.031
Bacteria	Acidobacte	Gp5	OTU_526	0.058	0.039	0.010	0.013	0.014
Bacteria	Proteobacte	Unclassifie	OTU_880	0.054	0.052	0.006	0.007	0.047
Bacteria	Proteobacte	Unclassifie	OTU_527	0.054	0.045	0.007	0.003	0.028
Bacteria	Chloroflexi	Unclassifie	OTU_1107	0.059	0.034	0.012	0.016	0.008
Bacteria	Actinobact	Terrabacte	OTU_7197	0.090	0.047	0.044	0.023	0.041
Bacteria	Actinobact	Aquihabita	OTU_1418	0.057	0.031	0.011	0.012	0.005
Bacteria	candidate d	WPS-1_gei	OTU_646	0.047	0.041	0.001	0.001	0.022
Bacteria	Unclassifie	Unclassifie	OTU_1346	0.051	0.031	0.006	0.009	0.006
Bacteria	Latescibact	Latescibact	OTU_5436	0.046	0.033	0.002	0.002	0.009
Bacteria	candidate d	WPS-1_gei	OTU_591	0.049	0.023	0.005	0.005	0.001

Bacteria	Bacteroidet	Phaeodacty	OTU_873	0.045	0.048	0.001	0.002	0.045
Bacteria	Actinobact	Actinophyt	OTU_4412	0.045	0.035	0.001	0.001	0.013
Bacteria	Proteobacte	Unclassifie	OTU_2868	0.051	0.025	0.007	0.005	0.002
Bacteria	Proteobacte	Unclassifie	OTU_414	0.051	0.027	0.008	0.010	0.003
Bacteria	Actinobact	Conexibact	OTU_500	0.047	0.033	0.004	0.007	0.010
Bacteria	Acidobacte	Aridibacter	OTU_696	0.044	0.031	0.002	0.002	0.008
Bacteria	Unclassifie	Unclassifie	OTU_449	0.046	0.031	0.004	0.004	0.009
Bacteria	Planctomyc	Zavarzinell	OTU_718	0.043	0.034	0.002	0.002	0.013
Bacteria	Acidobacte	Gp4	OTU_340	0.059	0.038	0.017	0.022	0.029
Bacteria	Proteobacte	Unclassifie	OTU_782	0.049	0.033	0.008	0.008	0.013
Bacteria	Verrucomic	Unclassifie	OTU_800	0.045	0.036	0.004	0.003	0.019
Bacteria	Acidobacte	Gp6	OTU_345	0.049	0.030	0.008	0.007	0.009
Bacteria	Verrucomic	Spartobacte	OTU_614	0.042	0.039	0.001	0.002	0.027
Bacteria	Unclassifie	Unclassifie	OTU_590	0.046	0.044	0.005	0.010	0.047
Bacteria	Proteobacte	Reyranella	OTU_595	0.060	0.041	0.020	0.013	0.037
Bacteria	Unclassifie	Unclassifie	OTU_403	0.045	0.033	0.005	0.004	0.015
Bacteria	Proteobacte	Lysobacter	OTU_814	0.043	0.034	0.003	0.003	0.016
Bacteria	Actinobact	Nocardioid	OTU_3526	0.041	0.035	0.002	0.003	0.019
Bacteria	Actinobact	Unclassifie	OTU_1717	0.047	0.033	0.008	0.011	0.015
Bacteria	Verrucomic	Subdivision	OTU_493	0.050	0.025	0.011	0.013	0.004
Bacteria	candidate d	WPS-1_gen	OTU_667	0.049	0.027	0.010	0.007	0.007
Bacteria	Proteobacte	Unclassifie	OTU_1235	0.046	0.035	0.006	0.009	0.022
Bacteria	Acidobacte	Gp3	OTU_1183	0.051	0.026	0.012	0.010	0.005
Bacteria	Proteobacte	Unclassifie	OTU_1096	0.049	0.026	0.010	0.009	0.005
Bacteria	Proteobacte	Unclassifie	OTU_1616	0.049	0.036	0.010	0.009	0.024
Bacteria	Unclassifie	Unclassifie	OTU_956	0.047	0.042	0.008	0.013	0.046
Bacteria	Bacteroidet	Mucilaginil	OTU_1401	0.050	0.023	0.011	0.009	0.003
Bacteria	Proteobacte	Unclassifie	OTU_576	0.062	0.032	0.024	0.014	0.016
Bacteria	Acidobacte	Gp6	OTU_6273	0.046	0.028	0.008	0.010	0.009
Bacteria	candidate d	WPS-1_gen	OTU_716	0.042	0.028	0.004	0.006	0.009
Bacteria	Proteobacte	Unclassifie	OTU_822	0.044	0.038	0.006	0.014	0.038
Bacteria	Unclassifie	Unclassifie	OTU_4079	0.046	0.037	0.009	0.017	0.034
Bacteria	Acidobacte	Gp6	OTU_3140	0.037	0.027	0.000	0.000	0.008
Bacteria	Armatimon	Armatimon	OTU_582	0.041	0.022	0.003	0.004	0.003
Bacteria	Acidobacte	Gp6	OTU_1382	0.039	0.041	0.002	0.003	0.050
Bacteria	Proteobacte	Sphingomo	OTU_1805	0.039	0.036	0.002	0.004	0.031
Bacteria	Proteobacte	Skermanell	OTU_352	0.058	0.033	0.022	0.023	0.034
Bacteria	Acidobacte	Unclassifie	OTU_271	0.052	0.017	0.015	0.014	0.001
Bacteria	Planctomyc	Pirellula	OTU_1057	0.041	0.018	0.005	0.005	0.001
Bacteria	Actinobact	Gaiella	OTU_755	0.057	0.025	0.021	0.018	0.009
Bacteria	Acidobacte	Gp10	OTU_561	0.039	0.034	0.003	0.003	0.029
Bacteria	Actinobact	Unclassifie	OTU_3055	0.048	0.025	0.012	0.015	0.009
Bacteria	Actinobact	Unclassifie	OTU_391	0.043	0.015	0.008	0.007	0.000
Bacteria	Proteobacte	Pedomicrol	OTU_1126	0.042	0.025	0.008	0.009	0.008
Bacteria	Actinobact	Aquihabita	OTU_1180	0.040	0.022	0.005	0.007	0.003
Bacteria	Bacteroidet	Unclassifie	OTU_1185	0.035	0.032	0.000	0.000	0.024
Bacteria	Bacteroidet	Terrimonas	OTU_1390	0.041	0.032	0.007	0.007	0.026
Bacteria	Proteobacte	Sphingomo	OTU_1414	0.037	0.017	0.003	0.004	0.001
Bacteria	Proteobacte	Unclassifie	OTU_280	0.036	0.020	0.002	0.002	0.003
Bacteria	candidate d	WPS-1_gen	OTU_776	0.034	0.031	0.000	0.000	0.024
Bacteria	Proteobacte	Povalibacte	OTU_350	0.041	0.034	0.008	0.005	0.035
Bacteria	Bacteroidet	Sediminiba	OTU_721	0.047	0.024	0.013	0.009	0.007
Bacteria	Acidobacte	Gp4	OTU_2665	0.040	0.030	0.006	0.009	0.021
Bacteria	Acidobacte	Gp3	OTU_1020	0.035	0.030	0.002	0.002	0.023
Bacteria	Verrucomic	Subdivision	OTU_5426	0.041	0.008	0.008	0.008	0.000
Bacteria	Proteobacte	Unclassifie	OTU_9399	0.048	0.031	0.015	0.011	0.027

Bacteria	Unclassifie	Unclassifie	OTU_1026	0.037	0.032	0.004	0.005	0.030
Bacteria	Proteobacte	Unclassifie	OTU_3729	0.037	0.034	0.004	0.006	0.037
Bacteria	Unclassifie	Unclassifie	OTU_1623	0.034	0.021	0.001	0.002	0.004
Bacteria	Actinobact	Unclassifie	OTU_8659	0.037	0.030	0.005	0.006	0.026
Bacteria	Acidobacte	Gp3	OTU_7700	0.034	0.021	0.002	0.003	0.005
Bacteria	candidate d	WPS-1_ge	OTU_326	0.050	0.028	0.018	0.020	0.029
Bacteria	Proteobacte	Unclassifie	OTU_921	0.034	0.021	0.002	0.004	0.005
Bacteria	Actinobact	Unclassifie	OTU_848	0.031	0.034	0.000	0.000	0.044
Bacteria	Proteobacte	Unclassifie	OTU_385	0.038	0.028	0.006	0.006	0.021
Bacteria	Planctomyc	Pirellula	OTU_1711	0.036	0.012	0.005	0.006	0.000
Bacteria	Proteobacte	Novosphin	OTU_7005	0.034	0.026	0.003	0.003	0.017
Bacteria	Gemmatim	Gemmatim	OTU_354	0.037	0.013	0.006	0.010	0.000
Bacteria	Actinobact	Conexibact	OTU_358	0.054	0.018	0.023	0.027	0.028
Bacteria	Unclassifie	Unclassifie	OTU_703	0.039	0.022	0.009	0.017	0.013
Bacteria	Proteobacte	Unclassifie	OTU_878	0.038	0.017	0.008	0.004	0.002
Bacteria	Proteobacte	Unclassifie	OTU_491	0.038	0.018	0.009	0.006	0.003
Bacteria	Unclassifie	Unclassifie	OTU_592	0.033	0.032	0.004	0.005	0.043
Bacteria	Proteobacte	Chondromy	OTU_746	0.053	0.030	0.023	0.016	0.046
Bacteria	Actinobact	Gaiella	OTU_935	0.037	0.012	0.008	0.012	0.000
Bacteria	Acidobacte	Gp6	OTU_674	0.032	0.027	0.003	0.004	0.025
Bacteria	Proteobacte	Bosea	OTU_383	0.043	0.021	0.014	0.010	0.008
Bacteria	Acidobacte	Gp17	OTU_2055	0.032	0.024	0.003	0.005	0.016
Bacteria	Actinobact	Gaiella	OTU_1998	0.038	0.025	0.009	0.009	0.019
Bacteria	Actinobact	Conexibact	OTU_4156	0.034	0.021	0.005	0.003	0.008
Bacteria	Proteobacte	Unclassifie	OTU_5265	0.037	0.021	0.009	0.005	0.009
Bacteria	Armatimon	Armatimon	OTU_1589	0.030	0.017	0.002	0.004	0.003
Bacteria	Chloroflexi	Unclassifie	OTU_682	0.028	0.015	0.000	0.000	0.001
Bacteria	candidate d	WPS-1_ge	OTU_1535	0.028	0.018	0.000	0.000	0.005
Bacteria	Gemmatim	Gemmatim	OTU_1104	0.031	0.018	0.003	0.003	0.004
Bacteria	Proteobacte	Unclassifie	OTU_1169	0.045	0.027	0.017	0.015	0.037
Bacteria	Proteobacte	Andersenie	OTU_941	0.030	0.025	0.002	0.005	0.025
Bacteria	Acidobacte	Gp4	OTU_842	0.030	0.014	0.002	0.003	0.001
Bacteria	Gemmatim	Gemmatim	OTU_958	0.027	0.030	0.000	0.000	0.049
Bacteria	Acidobacte	Gp6	OTU_466	0.027	0.018	0.000	0.000	0.006
Bacteria	Acidobacte	Geothrix	OTU_717	0.031	0.028	0.005	0.007	0.041
Bacteria	Proteobacte	Unclassifie	OTU_900	0.031	0.025	0.004	0.005	0.027
Bacteria	Acidobacte	Gp6	OTU_7800	0.029	0.020	0.003	0.005	0.009
Bacteria	candidate d	WPS-1_ge	OTU_1919	0.029	0.025	0.003	0.006	0.025
Bacteria	Chloroflexi	Unclassifie	OTU_1171	0.029	0.022	0.002	0.005	0.015
Bacteria	Acidobacte	Aridibacter	OTU_6837	0.029	0.023	0.002	0.002	0.018
Bacteria	Verrucomic	Subdivision	OTU_566	0.040	0.021	0.014	0.014	0.019
Bacteria	Actinobact	Nocardioid	OTU_2286	0.037	0.016	0.011	0.018	0.011
Bacteria	Proteobacte	Nitrosospir	OTU_4299	0.027	0.016	0.001	0.001	0.003
Bacteria	Actinobact	Virgisporai	OTU_1436	0.035	0.022	0.009	0.010	0.017
Bacteria	Unclassifie	Unclassifie	OTU_1391	0.028	0.015	0.002	0.003	0.003
Bacteria	Unclassifie	Unclassifie	OTU_1073	0.026	0.027	0.000	0.000	0.039
Bacteria	Unclassifie	Unclassifie	OTU_8085	0.032	0.027	0.006	0.013	0.044
Bacteria	Actinobact	Micromono	OTU_2165	0.032	0.028	0.007	0.002	0.049
Bacteria	Chloroflexi	Unclassifie	OTU_568	0.032	0.021	0.007	0.011	0.018
Bacteria	Acidobacte	Blastocatel	OTU_7207	0.025	0.027	0.000	0.000	0.043
Bacteria	Actinobact	Unclassifie	OTU_723	0.033	0.025	0.008	0.006	0.031
Bacteria	Actinobact	Unclassifie	OTU_7795	0.032	0.022	0.006	0.008	0.021
Bacteria	candidate d	WPS-1_ge	OTU_8125	0.026	0.020	0.001	0.002	0.013
Bacteria	Bacteroidet	Unclassifie	OTU_1718	0.028	0.023	0.003	0.003	0.022
Bacteria	Actinobact	Aciditerrim	OTU_739	0.039	0.015	0.014	0.012	0.004
Bacteria	Acidobacte	Gp6	OTU_2420	0.029	0.006	0.004	0.005	0.000

Bacteria	Actinobact	Conexibact	OTU_560	0.030	0.016	0.004	0.008	0.004
Bacteria	Proteobacte	Unclassifie	OTU_580	0.031	0.020	0.006	0.006	0.015
Bacteria	Proteobacte	Unclassifie	OTU_1051	0.026	0.011	0.001	0.003	0.000
Bacteria	Verrucomic	Subdivisior	OTU_483	0.027	0.016	0.003	0.003	0.005
Bacteria	Verrucomic	Opitutus	OTU_6677	0.028	0.023	0.003	0.003	0.026
Bacteria	Actinobact	Aquihabita	OTU_684	0.026	0.021	0.001	0.003	0.019
Bacteria	candidate d	WPS-1_ge	OTU_797	0.026	0.018	0.002	0.003	0.009
Bacteria	Unclassifie	Unclassifie	OTU_751	0.029	0.020	0.005	0.006	0.014
Bacteria	Acidobacte	Gp4	OTU_1424	0.026	0.019	0.002	0.004	0.013
Bacteria	Actinobact	Iamia	OTU_917	0.024	0.023	0.000	0.000	0.031
Bacteria	Verrucomic	Spartobacte	OTU_976	0.026	0.022	0.003	0.006	0.027
Bacteria	Acidobacte	Gp6	OTU_4035	0.031	0.022	0.007	0.004	0.026
Bacteria	Armatimon	Armatimon	OTU_1123	0.029	0.014	0.006	0.004	0.003
Bacteria	Unclassifie	Unclassifie	OTU_7920	0.027	0.022	0.004	0.006	0.030
Bacteria	Acidobacte	Gp4	OTU_951	0.026	0.013	0.003	0.004	0.002
Bacteria	Proteobacte	Unclassifie	OTU_821	0.025	0.023	0.002	0.006	0.034
Bacteria	Unclassifie	Unclassifie	OTU_1719	0.023	0.022	0.000	0.000	0.031
Bacteria	Acidobacte	Gp7	OTU_904	0.023	0.022	0.000	0.000	0.028
Bacteria	Actinobact	Aciditerrim	OTU_433	0.037	0.019	0.014	0.014	0.025
Bacteria	Proteobacte	Rhizobium	OTU_2356	0.028	0.022	0.006	0.003	0.031
Bacteria	Proteobacte	Panacagrim	OTU_573	0.027	0.014	0.004	0.005	0.003
Bacteria	Unclassifie	Unclassifie	OTU_2969	0.024	0.019	0.002	0.002	0.018
Bacteria	Verrucomic	Subdivisior	OTU_1194	0.022	0.017	0.000	0.000	0.010
Bacteria	Proteobacte	Pseudolabr	OTU_7843	0.034	0.012	0.012	0.006	0.001
Bacteria	Proteobacte	Povalibacte	OTU_1074	0.023	0.017	0.001	0.002	0.011
Bacteria	Proteobacte	Unclassifie	OTU_642	0.027	0.018	0.006	0.012	0.019
Bacteria	Acidobacte	Gp3	OTU_1017	0.027	0.024	0.005	0.005	0.050
Bacteria	Actinobact	Thermoleo	OTU_1112	0.031	0.014	0.010	0.011	0.007
Bacteria	Acidobacte	Gp3	OTU_3754	0.023	0.016	0.002	0.002	0.009
Bacteria	Unclassifie	Unclassifie	OTU_2649	0.022	0.021	0.000	0.000	0.030
Bacteria	Actinobact	Gaiella	OTU_726	0.031	0.018	0.010	0.013	0.025
Bacteria	Bacteroidet	Niastella	OTU_1837	0.023	0.015	0.002	0.003	0.007
Bacteria	Planctomyc	Gemmata	OTU_899	0.043	0.013	0.021	0.020	0.032
Bacteria	Bacteroidet	Unclassifie	OTU_8205	0.021	0.017	0.000	0.000	0.012
Bacteria	Actinobact	Unclassifie	OTU_845	0.023	0.014	0.001	0.002	0.005
Bacteria	Gemmatim	Gemmatim	OTU_412	0.022	0.012	0.001	0.002	0.002
Bacteria	Actinobact	Gaiella	OTU_5633	0.025	0.024	0.004	0.004	0.048
Bacteria	Acidobacte	Candidatus	OTU_5632	0.028	0.024	0.007	0.006	0.049
Bacteria	Actinobact	Unclassifie	OTU_5308	0.022	0.020	0.001	0.002	0.023
Bacteria	Proteobacte	Unclassifie	OTU_1304	0.028	0.020	0.007	0.008	0.028
Bacteria	Actinobact	Conexibact	OTU_4812	0.025	0.015	0.004	0.004	0.007
Bacteria	Proteobacte	Lysobacter	OTU_321	0.027	0.021	0.006	0.009	0.040
Bacteria	candidate d	WPS-1_ge	OTU_825	0.029	0.018	0.008	0.006	0.020
Bacteria	Acidobacte	Gp10	OTU_952	0.027	0.017	0.006	0.008	0.015
Bacteria	candidate d	WPS-1_ge	OTU_6891	0.023	0.020	0.002	0.004	0.033
Bacteria	Acidobacte	Gp4	OTU_770	0.022	0.020	0.002	0.004	0.031
Bacteria	Unclassifie	Unclassifie	OTU_3201	0.020	0.017	0.000	0.000	0.016
Bacteria	Proteobacte	Unclassifie	OTU_1827	0.022	0.012	0.002	0.003	0.003
Bacteria	Latescibact	Latescibact	OTU_898	0.023	0.018	0.003	0.006	0.020
Bacteria	candidate d	WPS-1_ge	OTU_1310	0.023	0.013	0.002	0.003	0.005
Bacteria	Chloroflexi	Unclassifie	OTU_4434	0.022	0.019	0.001	0.002	0.025
Bacteria	Actinobact	Thermoleo	OTU_5310	0.022	0.018	0.002	0.004	0.021
Bacteria	Armatimon	Armatimon	OTU_788	0.020	0.013	0.000	0.000	0.005
Bacteria	Bacteroidet	Unclassifie	OTU_957	0.029	0.012	0.010	0.009	0.004
Bacteria	Proteobacte	Methylobac	OTU_662	0.024	0.021	0.005	0.007	0.042
Bacteria	Proteobacte	Unclassifie	OTU_1270	0.020	0.015	0.001	0.002	0.011

Bacteria	Actinobact	Gaiella	OTU_1070	0.028	0.015	0.009	0.006	0.012
Bacteria	Actinobact	Unclassifie	OTU_6819	0.027	0.020	0.007	0.006	0.041
Bacteria	candidate d	WPS-1_ge	OTU_995	0.020	0.019	0.001	0.001	0.028
Bacteria	Acidobacte	Gp6	OTU_540	0.021	0.021	0.002	0.003	0.049
Bacteria	Actinobact	Allokutzne	OTU_6352	0.020	0.020	0.001	0.001	0.038
Bacteria	Actinobact	Aciditerrin	OTU_369	0.032	0.018	0.013	0.012	0.039
Bacteria	Acidobacte	Gp1	OTU_1753	0.021	0.019	0.003	0.004	0.033
Bacteria	Acidobacte	Gp6	OTU_7880	0.019	0.014	0.000	0.000	0.010
Bacteria	Gemmatim	Gemmatim	OTU_741	0.021	0.018	0.002	0.003	0.029
Bacteria	Actinobact	Hamadaea	OTU_430	0.024	0.017	0.005	0.004	0.025
Bacteria	Candidatus	Sacchariba	OTU_795	0.028	0.015	0.009	0.009	0.019
Bacteria	Proteobact	Inquilinus	OTU_1480	0.019	0.017	0.000	0.000	0.025
Bacteria	Planctomy	Gemmata	OTU_2315	0.022	0.015	0.004	0.003	0.013
Bacteria	Actinobact	Mycobacte	OTU_631	0.035	0.018	0.016	0.009	0.032
Bacteria	Planctomy	Unclassifie	OTU_1816	0.020	0.018	0.002	0.001	0.034
Bacteria	Bacteroide	Unclassifie	OTU_890	0.018	0.015	0.000	0.000	0.016
Bacteria	Actinobact	Unclassifie	OTU_1105	0.027	0.012	0.009	0.005	0.004
Bacteria	Unclassifie	Unclassifie	OTU_705	0.019	0.019	0.002	0.003	0.044
Bacteria	Actinobact	Gaiella	OTU_7346	0.018	0.019	0.000	0.000	0.040
Bacteria	Acidobacte	Gp3	OTU_851	0.026	0.015	0.009	0.008	0.022
Bacteria	candidate d	WPS-1_ge	OTU_920	0.018	0.009	0.001	0.001	0.001
Bacteria	Actinobact	Unclassifie	OTU_520	0.027	0.015	0.009	0.011	0.027
Bacteria	Acidobacte	Gp6	OTU_4250	0.020	0.016	0.002	0.003	0.026
Bacteria	Proteobact	Unclassifie	OTU_9435	0.031	0.015	0.014	0.012	0.031
Bacteria	candidate d	WPS-1_ge	OTU_585	0.031	0.013	0.013	0.008	0.013
Bacteria	Proteobact	Labilithrix	OTU_604	0.025	0.017	0.007	0.009	0.034
Bacteria	Unclassifie	Unclassifie	OTU_1603	0.017	0.018	0.000	0.000	0.036
Bacteria	Unclassifie	Unclassifie	OTU_769	0.019	0.017	0.003	0.005	0.037
Bacteria	Proteobact	Unclassifie	OTU_1146	0.019	0.008	0.003	0.006	0.001
Bacteria	Actinobact	Saccharoth	OTU_2471	0.016	0.007	0.000	0.000	0.001
Bacteria	Verrucomi	Subdivisio	OTU_1030	0.022	0.017	0.006	0.005	0.040
Bacteria	Acidobacte	Bryobacter	OTU_1316	0.016	0.010	0.000	0.000	0.004
Bacteria	Proteobact	Methyloph	OTU_511	0.020	0.015	0.004	0.005	0.025
Bacteria	Acidobacte	Gp6	OTU_528	0.021	0.013	0.005	0.004	0.014
Bacteria	Actinobact	Unclassifie	OTU_1599	0.018	0.013	0.002	0.002	0.016
Bacteria	Armatimon	Armatimon	OTU_870	0.022	0.017	0.006	0.009	0.046
Bacteria	Acidobacte	Gp3	OTU_613	0.021	0.013	0.006	0.008	0.017
Bacteria	Proteobact	Labilithrix	OTU_1193	0.020	0.009	0.004	0.002	0.002
Bacteria	Acidobacte	Gp5	OTU_809	0.028	0.008	0.013	0.006	0.001
Bacteria	Bacteroide	Chryseolin	OTU_960	0.016	0.013	0.000	0.000	0.015
Bacteria	Proteobact	Unclassifie	OTU_607	0.018	0.016	0.003	0.003	0.039
Bacteria	Proteobact	Methyloba	OTU_693	0.015	0.010	0.000	0.000	0.006
Bacteria	Bacteroide	Flavisoliba	OTU_8472	0.021	0.017	0.005	0.005	0.048
Bacteria	Acidobacte	Gp25	OTU_1538	0.015	0.016	0.000	0.000	0.045
Archaea	Thaumarch	Nitrososph	OTU_4268	0.017	0.016	0.002	0.002	0.042
Bacteria	candidate d	WPS-1_ge	OTU_2155	0.017	0.009	0.002	0.002	0.002
Bacteria	Verrucomi	Subdivisio	OTU_2272	0.015	0.009	0.000	0.000	0.003
Bacteria	Proteobact	Unclassifie	OTU_1434	0.016	0.013	0.002	0.003	0.020
Bacteria	Unclassifie	Unclassifie	OTU_374	0.016	0.015	0.001	0.002	0.040
Bacteria	Planctomy	Pirellula	OTU_2971	0.015	0.011	0.000	0.000	0.010
Bacteria	Acidobacte	Gp6	OTU_3783	0.019	0.016	0.004	0.005	0.043
Bacteria	Unclassifie	Unclassifie	OTU_678	0.018	0.015	0.003	0.003	0.038
Bacteria	candidate d	WPS-1_ge	OTU_1630	0.016	0.013	0.001	0.003	0.025
Bacteria	Actinobact	Saccharopo	OTU_1198	0.020	0.013	0.006	0.007	0.023
Bacteria	Unclassifie	Unclassifie	OTU_1415	0.018	0.016	0.004	0.005	0.048
Bacteria	Chloroflexi	Unclassifie	OTU_698	0.018	0.010	0.003	0.005	0.008

Bacteria	Proteobacte	Reyranella	OTU_2459	0.020	0.015	0.006	0.004	0.034
Bacteria	Proteobacte	Unclassifie	OTU_1496	0.014	0.013	0.000	0.000	0.022
Bacteria	Gemmatim	Gemmatim	OTU_486	0.014	0.011	0.000	0.000	0.009
Bacteria	Unclassifie	Unclassifie	OTU_2794	0.014	0.010	0.000	0.000	0.006
Bacteria	Planctomyc	Thermogut	OTU_2150	0.015	0.011	0.001	0.002	0.011
Bacteria	Actinobact	Unclassifie	OTU_5066	0.016	0.015	0.001	0.002	0.041
Bacteria	Proteobacte	Aggregicoc	OTU_1943	0.020	0.015	0.006	0.002	0.040
Bacteria	Acidobacte	Gp16	OTU_1249	0.016	0.012	0.002	0.004	0.020
Bacteria	candidate d	WPS-1_ge	OTU_1377	0.015	0.012	0.001	0.001	0.017
Bacteria	Planctomyc	Pirellula	OTU_1492	0.017	0.010	0.003	0.005	0.010
Bacteria	Unclassifie	Unclassifie	OTU_962	0.017	0.012	0.003	0.002	0.017
Bacteria	Proteobacte	Unclassifie	OTU_5292	0.014	0.015	0.000	0.000	0.043
Bacteria	Unclassifie	Unclassifie	OTU_813	0.018	0.014	0.004	0.004	0.032
Bacteria	Proteobacte	Unclassifie	OTU_1064	0.016	0.013	0.002	0.004	0.023
Bacteria	Proteobacte	Pseudomor	OTU_362	0.015	0.012	0.001	0.002	0.016
Bacteria	Actinobact	Nocardioid	OTU_4115	0.017	0.009	0.004	0.004	0.005
Bacteria	Proteobacte	Unclassifie	OTU_903	0.017	0.015	0.004	0.006	0.049
Bacteria	Chloroflexi	Unclassifie	OTU_2778	0.014	0.011	0.000	0.000	0.013
Bacteria	Unclassifie	Unclassifie	OTU_1281	0.015	0.014	0.002	0.003	0.037
Bacteria	Proteobacte	Unclassifie	OTU_2431	0.016	0.014	0.003	0.007	0.045
Bacteria	Proteobacte	Unclassifie	OTU_8769	0.015	0.013	0.001	0.002	0.033
Bacteria	Unclassifie	Unclassifie	OTU_874	0.015	0.012	0.001	0.003	0.026
Bacteria	Armatimon	Chthonomo	OTU_1984	0.014	0.011	0.001	0.002	0.018
Bacteria	Verrucomi	Subdivisio	OTU_3356	0.013	0.011	0.000	0.000	0.017
Bacteria	Actinobact	Unclassifie	OTU_4640	0.018	0.012	0.005	0.004	0.026
Bacteria	Actinobact	Unclassifie	OTU_5906	0.019	0.013	0.006	0.008	0.046
Bacteria	Proteobacte	Bauldia	OTU_1063	0.020	0.012	0.007	0.008	0.031
Bacteria	Actinobact	Aquihabita	OTU_871	0.014	0.013	0.001	0.002	0.036
Bacteria	Armatimon	Chthonomo	OTU_839	0.018	0.011	0.005	0.007	0.019
Bacteria	Proteobacte	Unclassifie	OTU_5005	0.013	0.012	0.001	0.001	0.023
Bacteria	Acidobacte	Gp6	OTU_6984	0.013	0.012	0.000	0.000	0.029
Bacteria	Actinobact	Aquihabita	OTU_1283	0.016	0.012	0.003	0.006	0.025
Bacteria	Verrucomi	Terrimicrol	OTU_2332	0.015	0.012	0.002	0.002	0.024
Bacteria	Planctomyc	Tepidispha	OTU_4314	0.013	0.012	0.000	0.000	0.027
Bacteria	Proteobacte	Unclassifie	OTU_1883	0.013	0.010	0.000	0.000	0.011
Bacteria	Verrucomi	Spartobacte	OTU_6076	0.013	0.011	0.001	0.002	0.017
Bacteria	Verrucomi	Subdivisio	OTU_3179	0.013	0.012	0.000	0.000	0.030
Bacteria	Unclassifie	Unclassifie	OTU_1362	0.013	0.013	0.000	0.000	0.035
Bacteria	Unclassifie	Unclassifie	OTU_1438	0.014	0.012	0.002	0.002	0.033
Bacteria	Proteobacte	Phenylobac	OTU_1875	0.015	0.012	0.003	0.003	0.032
Bacteria	Planctomyc	Singulispha	OTU_9305	0.013	0.010	0.001	0.002	0.015
Bacteria	Acidobacte	Gp6	OTU_3634	0.012	0.010	0.000	0.000	0.017
Bacteria	Actinobact	Unclassifie	OTU_1062	0.014	0.013	0.001	0.003	0.045
Bacteria	candidate d	WPS-1_ge	OTU_2873	0.012	0.008	0.000	0.000	0.006
Bacteria	Verrucomi	Subdivisio	OTU_1517	0.012	0.013	0.000	0.000	0.038
Bacteria	candidate d	WPS-1_ge	OTU_1965	0.014	0.012	0.001	0.003	0.032
Bacteria	Actinobact	Unclassifie	OTU_2744	0.014	0.007	0.002	0.003	0.002
Bacteria	Verrucomi	Spartobacte	OTU_4804	0.012	0.013	0.000	0.000	0.044
Bacteria	Planctomyc	Unclassifie	OTU_2541	0.012	0.010	0.000	0.000	0.013
Bacteria	Bacteroide	Ohtaekwan	OTU_1059	0.013	0.011	0.001	0.002	0.022
Bacteria	Unclassifie	Unclassifie	OTU_1606	0.016	0.009	0.004	0.004	0.007
Bacteria	Proteobacte	Sphingomo	OTU_1954	0.012	0.008	0.000	0.000	0.007
Bacteria	Verrucomi	Luteolibact	OTU_1021	0.013	0.012	0.001	0.001	0.031
Bacteria	Proteobacte	Unclassifie	OTU_7271	0.012	0.010	0.000	0.000	0.020
Bacteria	Acidobacte	Gp3	OTU_1798	0.012	0.008	0.000	0.000	0.006
Bacteria	Planctomyc	Unclassifie	OTU_1532	0.012	0.007	0.000	0.000	0.004

Bacteria	Proteobacte	Arenimona	OTU_8763	0.014	0.011	0.003	0.007	0.037
Bacteria	Unclassifie	Unclassifie	OTU_1863	0.012	0.013	0.000	0.000	0.046
Bacteria	Latescibact	Latescibact	OTU_3355	0.012	0.013	0.000	0.000	0.046
Bacteria	Unclassifie	Unclassifie	OTU_1230	0.015	0.008	0.003	0.005	0.008
Bacteria	Actinobact	Rhodococc	OTU_3768	0.014	0.010	0.002	0.003	0.022
Bacteria	Proteobacte	Unclassifie	OTU_1465	0.018	0.009	0.007	0.007	0.021
Bacteria	Actinobact	Unclassifie	OTU_3931	0.012	0.010	0.001	0.001	0.019
Bacteria	Proteobacte	Novosphin	OTU_7828	0.014	0.007	0.002	0.005	0.005
Bacteria	Acidobacte	Unclassifie	OTU_1647	0.011	0.012	0.000	0.000	0.043
Bacteria	Acidobacte	Aridibacter	OTU_1641	0.011	0.011	0.000	0.000	0.033
Bacteria	Proteobacte	Unclassifie	OTU_2387	0.017	0.007	0.006	0.008	0.011
Bacteria	Proteobacte	Unclassifie	OTU_1721	0.013	0.008	0.002	0.003	0.006
Bacteria	Armatimon	Chthonomo	OTU_6933	0.016	0.004	0.005	0.006	0.002
Bacteria	Actinobact	Unclassifie	OTU_840	0.014	0.011	0.003	0.005	0.031
Bacteria	Proteobacte	Bdellovibri	OTU_1115	0.013	0.011	0.001	0.002	0.029
Bacteria	Actinobact	Actinoplan	OTU_1936	0.012	0.012	0.001	0.001	0.038
Bacteria	Proteobacte	Unclassifie	OTU_3735	0.014	0.011	0.003	0.004	0.029
Bacteria	Chloroflexi	Unclassifie	OTU_2769	0.011	0.009	0.000	0.000	0.011
Bacteria	Bacteroidet	Flavisoliba	OTU_6302	0.015	0.011	0.004	0.007	0.044
Bacteria	Proteobacte	Noviherbas	OTU_7351	0.015	0.006	0.003	0.006	0.003
Bacteria	Bacteroidet	Unclassifie	OTU_810	0.011	0.012	0.000	0.000	0.039
Bacteria	Actinobact	Nocardioid	OTU_2483	0.013	0.008	0.002	0.003	0.009
Bacteria	candidate d	WPS-1_gei	OTU_7250	0.011	0.010	0.000	0.000	0.021
Bacteria	Acidobacte	Blastocatel	OTU_2445	0.011	0.008	0.000	0.000	0.009
Bacteria	Chloroflexi	Unclassifie	OTU_1621	0.011	0.007	0.000	0.000	0.004
Bacteria	Bacteroidet	Unclassifie	OTU_2603	0.011	0.010	0.000	0.000	0.030
Bacteria	Planctomyc	Unclassifie	OTU_1467	0.014	0.011	0.003	0.004	0.035
Bacteria	Unclassifie	Unclassifie	OTU_5626	0.011	0.010	0.001	0.002	0.024
Bacteria	Chloroflexi	Unclassifie	OTU_2208	0.011	0.009	0.000	0.000	0.021
Bacteria	Chloroflexi	Unclassifie	OTU_1560	0.011	0.009	0.000	0.000	0.014
Bacteria	Verrucomic	Opitutus	OTU_2746	0.016	0.011	0.006	0.006	0.045
Bacteria	Acidobacte	Gp16	OTU_2250	0.010	0.011	0.000	0.000	0.035
Bacteria	Planctomyc	Zavarzinell	OTU_2294	0.013	0.008	0.002	0.005	0.014
Bacteria	Planctomyc	Unclassifie	OTU_7512	0.010	0.009	0.000	0.000	0.016
Bacteria	Firmicutes	Unclassifie	OTU_2845	0.012	0.007	0.002	0.003	0.005
Bacteria	Chloroflexi	Unclassifie	OTU_1913	0.010	0.009	0.000	0.000	0.020
Bacteria	Latescibact	Latescibact	OTU_2755	0.010	0.010	0.000	0.000	0.029
Bacteria	Chloroflexi	Unclassifie	OTU_1044	0.010	0.010	0.000	0.000	0.028
Bacteria	Proteobacte	Unclassifie	OTU_3102	0.014	0.010	0.004	0.006	0.044
Bacteria	Proteobacte	Unclassifie	OTU_3039	0.010	0.007	0.000	0.000	0.007
Bacteria	Acidobacte	Gp25	OTU_256	0.010	0.011	0.000	0.000	0.046
Bacteria	Verrucomic	Subdivisior	OTU_2223	0.010	0.011	0.000	0.000	0.049
Bacteria	candidate d	WPS-1_gei	OTU_2446	0.010	0.008	0.000	0.000	0.012
Bacteria	Proteobacte	Acidisoma	OTU_8834	0.011	0.009	0.001	0.001	0.022
Bacteria	Bacteroidet	Chryseolin	OTU_2417	0.010	0.006	0.000	0.000	0.002
Bacteria	Bacteroidet	Hydrotalea	OTU_1094	0.011	0.007	0.002	0.002	0.009
Bacteria	Proteobacte	Unclassifie	OTU_3194	0.012	0.007	0.002	0.002	0.006
Bacteria	Gemmatim	Gemmatim	OTU_2325	0.010	0.010	0.000	0.000	0.042
Bacteria	Acidobacte	Gp6	OTU_355	0.010	0.008	0.000	0.000	0.017
Bacteria	Actinobact	Conexibact	OTU_456	0.010	0.009	0.000	0.000	0.022
Bacteria	Proteobacte	Unclassifie	OTU_830	0.014	0.006	0.004	0.006	0.011
Bacteria	Actinobact	Conexibact	OTU_5472	0.010	0.010	0.001	0.001	0.038
Bacteria	Bacteroidet	Chryseolin	OTU_1212	0.010	0.010	0.000	0.000	0.043
Bacteria	Planctomyc	Pirellula	OTU_6664	0.009	0.006	0.000	0.000	0.004
Bacteria	Acidobacte	Gp6	OTU_3297	0.009	0.007	0.000	0.000	0.008
Bacteria	Actinobact	Conexibact	OTU_612	0.009	0.008	0.000	0.000	0.021

Bacteria	Planctomyc	Unclassifie	OTU_2244	0.009	0.006	0.000	0.000	0.003
Bacteria	Unclassifie	Unclassifie	OTU_2557	0.009	0.010	0.000	0.000	0.044
Bacteria	Proteobacte	Geminicocc	OTU_2260	0.009	0.010	0.000	0.000	0.043
Bacteria	Acidobacte	Gp6	OTU_5139	0.009	0.010	0.000	0.000	0.047
Bacteria	Planctomyc	Blastopirel	OTU_1534	0.009	0.007	0.000	0.000	0.011
Bacteria	Proteobacte	Caulobacte	OTU_1229	0.012	0.008	0.003	0.005	0.031
Bacteria	Verrucomic	Opitutus	OTU_1426	0.009	0.010	0.000	0.000	0.048
Bacteria	Bacteroidet	Terrimonas	OTU_4309	0.009	0.006	0.000	0.000	0.004
Bacteria	Proteobacte	Unclassifie	OTU_2151	0.009	0.009	0.000	0.000	0.038
Bacteria	Verrucomic	Spartobacte	OTU_4809	0.010	0.010	0.001	0.001	0.044
Bacteria	Unclassifie	Unclassifie	OTU_3596	0.009	0.010	0.000	0.000	0.042
Bacteria	Cyanobacte	GpXIII	OTU_1504	0.009	0.010	0.000	0.000	0.045
Bacteria	Armatimon	Armatimon	OTU_2615	0.011	0.007	0.002	0.002	0.015
Bacteria	Bacteroidet	Ferruginiba	OTU_1133	0.009	0.008	0.000	0.000	0.022
Bacteria	Chloroflexi	Unclassifie	OTU_2104	0.009	0.010	0.000	0.000	0.045
Bacteria	Proteobacte	Unclassifie	OTU_1004	0.009	0.007	0.000	0.000	0.017
Bacteria	Proteobacte	Caulobacte	OTU_2506	0.011	0.009	0.002	0.002	0.035
Bacteria	Proteobacte	Chondromy	OTU_3839	0.010	0.007	0.001	0.001	0.009
Bacteria	Actinobact	Conexibact	OTU_1829	0.009	0.009	0.000	0.000	0.032
Bacteria	Unclassifie	Unclassifie	OTU_4392	0.009	0.007	0.000	0.000	0.015
Bacteria	Gemmatim	Gemmatim	OTU_1241	0.011	0.008	0.002	0.003	0.028
Bacteria	Actinobact	Conexibact	OTU_5450	0.009	0.007	0.000	0.000	0.015
Bacteria	Bacteroidet	Unclassifie	OTU_1716	0.009	0.008	0.000	0.000	0.023
Bacteria	Actinobact	Aciditerrim	OTU_1500	0.009	0.007	0.000	0.000	0.011
Bacteria	Verrucomic	Spartobacte	OTU_7907	0.008	0.009	0.000	0.000	0.042
Bacteria	Candidatus	Saccharibac	OTU_857	0.008	0.008	0.000	0.000	0.033
Bacteria	Verrucomic	Subdivision	OTU_4999	0.010	0.008	0.002	0.002	0.029
Bacteria	Bacteroidet	Terrimonas	OTU_4471	0.008	0.007	0.000	0.000	0.018
Bacteria	Unclassifie	Unclassifie	OTU_3064	0.008	0.005	0.000	0.000	0.003
Bacteria	Verrucomic	Subdivision	OTU_9375	0.008	0.008	0.000	0.000	0.024
Bacteria	Bacteroidet	Unclassifie	OTU_4163	0.009	0.008	0.001	0.001	0.026
Bacteria	Verrucomic	Unclassifie	OTU_3688	0.008	0.007	0.000	0.000	0.020
Bacteria	Bacteroidet	Ferruginiba	OTU_7690	0.009	0.008	0.001	0.001	0.035
Bacteria	Bacteroidet	Chitinopha	OTU_2805	0.008	0.008	0.000	0.000	0.034
Bacteria	Verrucomic	Subdivision	OTU_4051	0.008	0.006	0.000	0.000	0.012
Bacteria	Unclassifie	Unclassifie	OTU_8947	0.008	0.008	0.000	0.000	0.036
Bacteria	Proteobacte	Unclassifie	OTU_2304	0.009	0.008	0.001	0.001	0.029
Bacteria	Planctomyc	Pirellula	OTU_1713	0.008	0.008	0.000	0.000	0.042
Bacteria	Proteobacte	Rhodopseu	OTU_959	0.010	0.007	0.002	0.002	0.025
Bacteria	Proteobacte	Unclassifie	OTU_2017	0.008	0.007	0.000	0.000	0.018
Bacteria	Acidobacte	Gp6	OTU_7338	0.008	0.006	0.000	0.000	0.009
Bacteria	Planctomyc	Planctopiru	OTU_2128	0.008	0.007	0.000	0.000	0.022
Bacteria	Planctomyc	Planctopiru	OTU_3538	0.010	0.009	0.002	0.002	0.048
Bacteria	Actinobact	Aciditerrim	OTU_2084	0.008	0.006	0.000	0.000	0.008
Bacteria	Planctomyc	Zavarzinell	OTU_2846	0.008	0.006	0.000	0.000	0.010
Bacteria	Bacteroidet	Flavobacte	OTU_2345	0.008	0.007	0.000	0.000	0.027
Bacteria	candidate d	WPS-1_ger	OTU_1429	0.008	0.007	0.001	0.001	0.025
Bacteria	Bacteroidet	Ferruginiba	OTU_6886	0.010	0.007	0.002	0.003	0.028
Bacteria	Acidobacte	Gp5	OTU_8200	0.009	0.006	0.001	0.003	0.012
Bacteria	Unclassifie	Unclassifie	OTU_6882	0.011	0.008	0.003	0.004	0.050
Bacteria	Unclassifie	Unclassifie	OTU_4819	0.008	0.008	0.000	0.000	0.038
Bacteria	Armatimon	Armatimon	OTU_1509	0.009	0.008	0.001	0.002	0.044
Bacteria	Chloroflexi	Unclassifie	OTU_2617	0.007	0.008	0.000	0.000	0.040
Bacteria	Proteobacte	Unclassifie	OTU_3392	0.007	0.005	0.000	0.000	0.007
Bacteria	Proteobacte	Unclassifie	OTU_3786	0.007	0.006	0.000	0.000	0.018
Bacteria	Planctomyc	Singulispha	OTU_3706	0.007	0.006	0.000	0.000	0.014

Bacteria	Acidobacte	Gp4	OTU_3425	0.007	0.007	0.000	0.000	0.031
Bacteria	Proteobacte	Unclassifie	OTU_2788	0.009	0.006	0.002	0.002	0.016
Bacteria	Unclassifie	Unclassifie	OTU_3933	0.007	0.007	0.000	0.000	0.035
Bacteria	Chloroflexi	Unclassifie	OTU_1923	0.008	0.008	0.001	0.002	0.042
Bacteria	Bacteroidet	Sediminiba	OTU_1794	0.009	0.007	0.002	0.002	0.037
Bacteria	Proteobacte	Unclassifie	OTU_2419	0.011	0.006	0.004	0.004	0.026
Bacteria	candidate d	WPS-1_gen	OTU_9283	0.007	0.008	0.000	0.000	0.040
Bacteria	Acidobacte	Blastocatel	OTU_1741	0.007	0.005	0.000	0.000	0.008
Bacteria	Actinobacte	Iamia	OTU_2518	0.008	0.003	0.001	0.001	0.000
Bacteria	Proteobacte	Enhygromy	OTU_1851	0.009	0.007	0.001	0.003	0.037
Bacteria	Candidatus	Saccharibac	OTU_5805	0.009	0.007	0.001	0.003	0.034
Bacteria	Actinobacte	Aquihabita	OTU_7295	0.009	0.005	0.002	0.002	0.004
Bacteria	candidate d	WPS-1_gen	OTU_7605	0.007	0.006	0.000	0.000	0.013
Bacteria	Proteobacte	Unclassifie	OTU_1728	0.008	0.004	0.001	0.001	0.001
Bacteria	Acidobacte	Gp16	OTU_1376	0.009	0.005	0.002	0.002	0.010
Bacteria	Acidobacte	Gp10	OTU_1677	0.009	0.006	0.002	0.004	0.021
Bacteria	Unclassifie	Unclassifie	OTU_9118	0.007	0.007	0.000	0.000	0.037
Bacteria	Planctomyc	Unclassifie	OTU_2312	0.007	0.004	0.000	0.000	0.001
Bacteria	Actinobacte	Unclassifie	OTU_6829	0.007	0.006	0.000	0.000	0.024
Bacteria	Proteobacte	Haliangium	OTU_8128	0.007	0.006	0.000	0.000	0.018
Bacteria	Acidobacte	Gp6	OTU_2521	0.007	0.005	0.000	0.000	0.008
Bacteria	Planctomyc	Zavarzinell	OTU_5281	0.008	0.006	0.001	0.001	0.026
Bacteria	Unclassifie	Unclassifie	OTU_2003	0.008	0.006	0.001	0.001	0.019
Bacteria	Unclassifie	Unclassifie	OTU_5032	0.007	0.007	0.000	0.000	0.042
Bacteria	Actinobacte	Nakamurel	OTU_9035	0.008	0.006	0.001	0.002	0.014
Bacteria	Proteobacte	Unclassifie	OTU_1201	0.007	0.005	0.000	0.000	0.009
Bacteria	Actinobacte	Unclassifie	OTU_4580	0.007	0.006	0.000	0.000	0.024
Bacteria	Actinobacte	Iamia	OTU_4097	0.007	0.006	0.000	0.000	0.021
Bacteria	Planctomyc	Isosphaera	OTU_2246	0.007	0.005	0.001	0.001	0.012
Bacteria	Planctomyc	Unclassifie	OTU_1396	0.008	0.006	0.001	0.002	0.031
Bacteria	Proteobacte	Chelatococ	OTU_2012	0.006	0.006	0.000	0.000	0.029
Bacteria	Acidobacte	Gp5	OTU_3530	0.006	0.007	0.000	0.000	0.043
Bacteria	Proteobacte	Unclassifie	OTU_8293	0.006	0.004	0.000	0.000	0.005
Bacteria	Acidobacte	Gp3	OTU_4707	0.006	0.006	0.000	0.000	0.030
Bacteria	Planctomyc	Unclassifie	OTU_1478	0.006	0.005	0.000	0.000	0.014
Bacteria	Verrucomic	Subdivision	OTU_3939	0.006	0.005	0.000	0.000	0.014
Bacteria	Actinobacte	Catelliglob	OTU_3483	0.009	0.006	0.002	0.004	0.049
Bacteria	Planctomyc	Gemmata	OTU_6905	0.006	0.006	0.000	0.000	0.035
Bacteria	candidate d	WPS-1_gen	OTU_2548	0.006	0.006	0.000	0.000	0.032
Bacteria	Armatimon	Armatimon	OTU_2872	0.006	0.006	0.000	0.000	0.020
Bacteria	Proteobacte	Sphingobiu	OTU_7661	0.006	0.007	0.000	0.000	0.040
Bacteria	Armatimon	Armatimon	OTU_3113	0.006	0.006	0.000	0.000	0.025
Bacteria	Actinobacte	Iamia	OTU_1433	0.007	0.006	0.001	0.002	0.025
Bacteria	candidate d	WPS-1_gen	OTU_9312	0.006	0.005	0.000	0.000	0.017
Bacteria	Planctomyc	Rubinispha	OTU_3817	0.006	0.005	0.000	0.000	0.017
Bacteria	Unclassifie	Unclassifie	OTU_2080	0.006	0.006	0.000	0.000	0.037
Bacteria	Proteobacte	Unclassifie	OTU_2409	0.006	0.005	0.000	0.000	0.016
Bacteria	Proteobacte	Unclassifie	OTU_8333	0.009	0.006	0.003	0.004	0.034
Bacteria	Actinobacte	Gaiella	OTU_3444	0.006	0.006	0.000	0.000	0.023
Bacteria	Armatimon	Armatimon	OTU_4866	0.006	0.005	0.000	0.000	0.011
Bacteria	Firmicutes	Unclassifie	OTU_2796	0.007	0.005	0.001	0.002	0.015
Bacteria	Acidobacte	Gp10	OTU_4195	0.008	0.006	0.002	0.002	0.022
Bacteria	Proteobacte	Unclassifie	OTU_3076	0.006	0.006	0.000	0.000	0.033
Bacteria	Acidobacte	Gp6	OTU_4404	0.007	0.007	0.001	0.002	0.045
Bacteria	Unclassifie	Unclassifie	OTU_2062	0.009	0.006	0.003	0.004	0.047
Bacteria	Actinobacte	Conexibact	OTU_2276	0.006	0.005	0.000	0.000	0.021

Bacteria	Armatimon	Armatimon	OTU_1951	0.007	0.004	0.001	0.002	0.008
Bacteria	Actinobact	Unclassifie	OTU_3396	0.006	0.005	0.000	0.000	0.019
Bacteria	Proteobacte	Unclassifie	OTU_1165	0.006	0.006	0.001	0.001	0.037
Bacteria	Bacteroidet	Unclassifie	OTU_1821	0.006	0.003	0.000	0.000	0.003
Bacteria	Acidobacte	Gp3	OTU_901	0.007	0.006	0.002	0.003	0.047
Bacteria	Acidobacte	Gp3	OTU_1774	0.006	0.005	0.000	0.000	0.019
Bacteria	candidate d	WPS-1_ge	OTU_1973	0.008	0.006	0.002	0.002	0.040
Bacteria	Armatimon	Armatimon	OTU_1896	0.006	0.004	0.000	0.000	0.011
Bacteria	Acidobacte	Gp17	OTU_8421	0.006	0.003	0.000	0.000	0.002
Bacteria	Planctomyc	Unclassifie	OTU_2584	0.006	0.006	0.000	0.000	0.036
Bacteria	candidate d	WPS-1_ge	OTU_6717	0.006	0.006	0.000	0.000	0.045
Bacteria	Actinobact	Ilumatobac	OTU_3819	0.007	0.006	0.001	0.002	0.047
Bacteria	Planctomyc	Gemmata	OTU_2039	0.008	0.006	0.002	0.002	0.033
Bacteria	Actinobact	Gaiella	OTU_7254	0.009	0.004	0.003	0.004	0.019
Bacteria	Planctomyc	Unclassifie	OTU_3856	0.006	0.005	0.000	0.000	0.017
Bacteria	Candidatus	Saccharibac	OTU_1958	0.006	0.005	0.001	0.001	0.014
Bacteria	Actinobact	Gaiella	OTU_5514	0.006	0.006	0.000	0.000	0.032
Bacteria	Proteobacte	Arenimona	OTU_4086	0.007	0.006	0.001	0.003	0.046
Bacteria	Proteobacte	Unclassifie	OTU_1881	0.006	0.005	0.000	0.000	0.024
Bacteria	Acidobacte	Unclassifie	OTU_3182	0.006	0.005	0.000	0.000	0.029
Bacteria	Proteobacte	Unclassifie	OTU_8932	0.006	0.005	0.000	0.000	0.019
Bacteria	Proteobacte	Jahnella	OTU_1242	0.006	0.004	0.000	0.000	0.012
Bacteria	Planctomyc	Unclassifie	OTU_3806	0.006	0.004	0.000	0.000	0.012
Bacteria	Verrucomi	Spartobacte	OTU_2866	0.006	0.006	0.000	0.000	0.033
Bacteria	Acidobacte	Gp16	OTU_2911	0.007	0.006	0.002	0.003	0.043
Bacteria	Unclassifie	Unclassifie	OTU_2504	0.005	0.004	0.000	0.000	0.008
Bacteria	Latescibact	Latescibact	OTU_3746	0.006	0.005	0.001	0.001	0.031
Bacteria	Bacteroidet	Unclassifie	OTU_2087	0.005	0.006	0.000	0.000	0.044
Bacteria	Gemmatim	Gemmatim	OTU_6895	0.005	0.005	0.000	0.000	0.031
Bacteria	Planctomyc	Tepidispha	OTU_5781	0.005	0.006	0.000	0.000	0.042
Bacteria	Acidobacte	Gp6	OTU_3945	0.005	0.005	0.000	0.000	0.032
Bacteria	Acidobacte	Gp6	OTU_3325	0.005	0.005	0.000	0.000	0.035
Bacteria	Planctomyc	Planctomic	OTU_3460	0.005	0.003	0.000	0.000	0.002
Bacteria	Chloroflexi	Unclassifie	OTU_2173	0.005	0.003	0.000	0.000	0.002
Bacteria	Proteobacte	Unclassifie	OTU_3371	0.007	0.005	0.002	0.002	0.032
Bacteria	Proteobacte	Unclassifie	OTU_885	0.005	0.005	0.000	0.000	0.036
Bacteria	Unclassifie	Unclassifie	OTU_7035	0.005	0.004	0.000	0.000	0.013
Bacteria	Armatimon	Armatimon	OTU_4155	0.005	0.004	0.000	0.000	0.017
Bacteria	Acidobacte	Gp16	OTU_2624	0.005	0.005	0.000	0.000	0.029
Bacteria	Chloroflexi	Unclassifie	OTU_6189	0.005	0.005	0.000	0.000	0.021
Bacteria	Verrucomi	Subdivisio	OTU_3133	0.005	0.004	0.000	0.000	0.011
Bacteria	Unclassifie	Unclassifie	OTU_2567	0.005	0.004	0.000	0.000	0.013
Bacteria	Verrucomi	Unclassifie	OTU_1366	0.005	0.004	0.000	0.000	0.014
Bacteria	Unclassifie	Unclassifie	OTU_5952	0.005	0.005	0.000	0.000	0.037
Bacteria	Acidobacte	Gp6	OTU_2883	0.006	0.005	0.001	0.001	0.025
Bacteria	Planctomyc	Blastopirel	OTU_6162	0.005	0.004	0.000	0.000	0.013
Bacteria	Unclassifie	Unclassifie	OTU_1615	0.007	0.004	0.002	0.004	0.046
Bacteria	Verrucomi	Unclassifie	OTU_4063	0.005	0.006	0.000	0.000	0.050
Bacteria	Planctomyc	Zavarzinell	OTU_5824	0.006	0.005	0.001	0.001	0.024
Bacteria	Chloroflexi	Bellilinea	OTU_1380	0.005	0.003	0.000	0.000	0.003
Bacteria	Unclassifie	Unclassifie	OTU_3601	0.005	0.005	0.000	0.000	0.031
Bacteria	Bacteroidet	Mucilaginil	OTU_3094	0.007	0.005	0.002	0.002	0.041
Bacteria	Proteobacte	Unclassifie	OTU_6521	0.005	0.005	0.000	0.000	0.032
Bacteria	Unclassifie	Unclassifie	OTU_1318	0.005	0.005	0.000	0.000	0.027
Bacteria	Unclassifie	Unclassifie	OTU_3540	0.007	0.004	0.002	0.002	0.014
Bacteria	Planctomyc	Pirellula	OTU_3432	0.005	0.004	0.000	0.000	0.017

Bacteria	Proteobacte	Haliangium	OTU_3125	0.005	0.005	0.000	0.000	0.040
Bacteria	Acidobacte	Gp6	OTU_5353	0.005	0.005	0.000	0.000	0.028
Bacteria	Proteobacte	Unclassifie	OTU_1836	0.006	0.005	0.001	0.002	0.041
Bacteria	Actinobacte	Unclassifie	OTU_4060	0.005	0.005	0.000	0.000	0.031
Bacteria	Planctomyc	Planctopiru	OTU_2497	0.006	0.005	0.001	0.001	0.041
Bacteria	Proteobacte	Unclassifie	OTU_4636	0.005	0.005	0.000	0.000	0.028
Bacteria	Actinobacte	Unclassifie	OTU_1598	0.008	0.004	0.003	0.003	0.020
Bacteria	Proteobacte	Unclassifie	OTU_5048	0.005	0.005	0.000	0.000	0.035
Bacteria	Acidobacte	Gp17	OTU_1738	0.005	0.005	0.001	0.001	0.030
Bacteria	Actinobacte	Unclassifie	OTU_3321	0.005	0.003	0.000	0.000	0.007
Bacteria	Proteobacte	Unclassifie	OTU_4389	0.005	0.005	0.000	0.000	0.034
Bacteria	Verrucomic	Unclassifie	OTU_3454	0.005	0.004	0.000	0.000	0.023
Bacteria	Proteobacte	Haliangium	OTU_3091	0.005	0.004	0.000	0.000	0.017
Bacteria	candidate d	WPS-1_ge	OTU_4134	0.005	0.005	0.000	0.000	0.031
Bacteria	Proteobacte	Unclassifie	OTU_6843	0.005	0.005	0.000	0.000	0.034
Bacteria	Chloroflexi	Ornatilinea	OTU_3850	0.005	0.004	0.000	0.000	0.029
Bacteria	Proteobacte	Unclassifie	OTU_9466	0.005	0.005	0.000	0.000	0.046
Bacteria	Actinobacte	Solirubroba	OTU_7926	0.005	0.004	0.000	0.000	0.014
Bacteria	Actinobacte	Ferrimicrob	OTU_4303	0.005	0.004	0.000	0.000	0.015
Bacteria	Acidobacte	Gp6	OTU_2450	0.005	0.005	0.000	0.000	0.032
Bacteria	Proteobacte	Phaselicyst	OTU_1824	0.004	0.005	0.000	0.000	0.046
Bacteria	Gemmatim	Gemmatim	OTU_2111	0.004	0.004	0.000	0.000	0.016
Bacteria	Planctomyc	Unclassifie	OTU_7472	0.004	0.005	0.000	0.000	0.043
Bacteria	Acidobacte	Gp6	OTU_8678	0.005	0.003	0.001	0.001	0.010
Bacteria	Acidobacte	Gp10	OTU_4730	0.004	0.004	0.000	0.000	0.031
Bacteria	Proteobacte	Byssovorax	OTU_1681	0.004	0.004	0.000	0.000	0.039
Bacteria	Acidobacte	Gp4	OTU_1494	0.007	0.003	0.003	0.004	0.039
Bacteria	Bacteroidet	Terrimonas	OTU_6910	0.004	0.005	0.000	0.000	0.042
Bacteria	Proteobacte	Unclassifie	OTU_3422	0.004	0.004	0.000	0.000	0.023
Bacteria	Unclassifie	Unclassifie	OTU_4293	0.004	0.004	0.000	0.000	0.029
Bacteria	Gemmatim	Gemmatim	OTU_1736	0.004	0.004	0.000	0.000	0.038
Bacteria	Gemmatim	Gemmatim	OTU_2871	0.004	0.004	0.000	0.000	0.026
Bacteria	candidate d	WPS-1_ge	OTU_4611	0.004	0.003	0.000	0.000	0.013
Bacteria	Actinobacte	Actinomyc	OTU_2063	0.006	0.002	0.002	0.002	0.004
Bacteria	Actinobacte	Unclassifie	OTU_5208	0.004	0.004	0.000	0.000	0.024
Bacteria	BRC1	BRC1_gen	OTU_4523	0.004	0.004	0.000	0.000	0.046
Bacteria	candidate d	WPS-1_ge	OTU_3121	0.004	0.004	0.000	0.000	0.026
Bacteria	Proteobacte	Unclassifie	OTU_4898	0.004	0.004	0.000	0.000	0.020
Bacteria	Proteobacte	Rhizomicro	OTU_2172	0.004	0.004	0.000	0.000	0.022
Bacteria	Candidatus	Saccharibac	OTU_1664	0.004	0.004	0.000	0.000	0.045
Bacteria	candidate d	WPS-1_ge	OTU_2029	0.006	0.004	0.002	0.002	0.044
Bacteria	Actinobacte	Unclassifie	OTU_2916	0.004	0.003	0.000	0.000	0.018
Bacteria	Proteobacte	Unclassifie	OTU_2187	0.004	0.004	0.000	0.000	0.040
Bacteria	Unclassifie	Unclassifie	OTU_4902	0.004	0.004	0.000	0.000	0.047
Bacteria	Bacteroidet	Terrimonas	OTU_3283	0.004	0.003	0.000	0.000	0.005
Bacteria	Chloroflexi	Unclassifie	OTU_4666	0.004	0.004	0.000	0.000	0.040
Bacteria	Planctomyc	Gemmata	OTU_6831	0.004	0.003	0.000	0.000	0.023
Bacteria	Actinobacte	Ilumatobac	OTU_7858	0.004	0.004	0.000	0.000	0.030
Bacteria	Actinobacte	Unclassifie	OTU_2685	0.004	0.004	0.000	0.000	0.034
Bacteria	Actinobacte	Unclassifie	OTU_2166	0.006	0.003	0.002	0.003	0.044
Bacteria	Acidobacte	Gp25	OTU_2232	0.004	0.003	0.000	0.000	0.019
Bacteria	Proteobacte	Unclassifie	OTU_4647	0.004	0.002	0.000	0.000	0.005
Bacteria	Acidobacte	Gp6	OTU_7291	0.004	0.004	0.000	0.000	0.045
Bacteria	Armatimon	Armatimon	OTU_2405	0.004	0.004	0.000	0.000	0.042
Bacteria	Proteobacte	Paracoccus	OTU_1699	0.005	0.004	0.001	0.002	0.032
Bacteria	Unclassifie	Unclassifie	OTU_4196	0.004	0.004	0.000	0.000	0.030

Bacteria	Chloroflexi	Litorilinea	OTU_5391	0.004	0.003	0.000	0.000	0.021
Bacteria	Acidobacte	Gp5	OTU_2586	0.004	0.004	0.001	0.001	0.040
Bacteria	Planctomyc	Unclassifie	OTU_3697	0.004	0.003	0.000	0.000	0.019
Bacteria	Planctomyc	Thermogut	OTU_4149	0.004	0.004	0.000	0.000	0.038
Bacteria	Planctomyc	Unclassifie	OTU_5613	0.004	0.002	0.000	0.000	0.007
Bacteria	Proteobacte	Unclassifie	OTU_2820	0.004	0.004	0.000	0.000	0.047
Bacteria	Acidobacte	Gp4	OTU_5736	0.004	0.004	0.000	0.000	0.050
Bacteria	Bacteroidet	Ohtaekwan	OTU_1120	0.003	0.002	0.000	0.000	0.002
Bacteria	Actinobact	Nocardioid	OTU_4117	0.003	0.004	0.000	0.000	0.047
Bacteria	Acidobacte	Gp1	OTU_2070	0.003	0.004	0.000	0.000	0.043
Bacteria	Unclassifie	Unclassifie	OTU_7617	0.003	0.003	0.000	0.000	0.029
Bacteria	Unclassifie	Unclassifie	OTU_2284	0.003	0.003	0.000	0.000	0.029
Bacteria	Acidobacte	Gp6	OTU_2495	0.003	0.003	0.000	0.000	0.036
Bacteria	Proteobacte	Solimonas	OTU_2123	0.003	0.004	0.000	0.000	0.043
Bacteria	Proteobacte	Unclassifie	OTU_6078	0.003	0.004	0.000	0.000	0.047
Bacteria	Verrucomic	Luteolibact	OTU_3787	0.003	0.003	0.000	0.000	0.035
Bacteria	Proteobacte	Unclassifie	OTU_2340	0.003	0.002	0.000	0.000	0.008
Bacteria	Acidobacte	Gp6	OTU_1823	0.003	0.004	0.000	0.000	0.046
Bacteria	Unclassifie	Unclassifie	OTU_3616	0.004	0.003	0.001	0.001	0.029
Bacteria	Verrucomic	Unclassifie	OTU_2869	0.003	0.003	0.000	0.000	0.038
Bacteria	Armatimon	Chthonomo	OTU_4176	0.003	0.003	0.000	0.000	0.038
Bacteria	Planctomyc	Unclassifie	OTU_3721	0.003	0.003	0.000	0.000	0.019
Bacteria	Acidobacte	Gp6	OTU_5299	0.003	0.003	0.000	0.000	0.012
Bacteria	Acidobacte	Bryobacter	OTU_1053	0.003	0.003	0.000	0.000	0.026
Bacteria	Planctomyc	Zavarzinell	OTU_6146	0.003	0.004	0.000	0.000	0.046
Bacteria	Unclassifie	Unclassifie	OTU_2980	0.003	0.003	0.000	0.000	0.043
Bacteria	Planctomyc	Candidatus	OTU_4721	0.003	0.004	0.000	0.000	0.048
Bacteria	Unclassifie	Unclassifie	OTU_2422	0.003	0.003	0.000	0.000	0.043
Bacteria	Proteobacte	Sandaracin	OTU_6792	0.003	0.003	0.000	0.000	0.025
Bacteria	Proteobacte	Azonexus	OTU_2893	0.003	0.003	0.000	0.000	0.041
Bacteria	Bacteroidet	Unclassifie	OTU_4673	0.003	0.003	0.000	0.000	0.048
Bacteria	Actinobact	Unclassifie	OTU_7326	0.003	0.002	0.000	0.001	0.008
Bacteria	Unclassifie	Unclassifie	OTU_1547	0.003	0.003	0.000	0.000	0.015
Bacteria	Planctomyc	Unclassifie	OTU_2204	0.003	0.003	0.000	0.000	0.041
Bacteria	Bacteroidet	Unclassifie	OTU_1842	0.003	0.003	0.000	0.000	0.037
Bacteria	Proteobacte	Unclassifie	OTU_3921	0.003	0.003	0.000	0.000	0.035
Bacteria	Acidobacte	Gp3	OTU_2324	0.003	0.003	0.000	0.000	0.048
Bacteria	Acidobacte	Gp16	OTU_1274	0.004	0.003	0.001	0.001	0.020
Bacteria	Chloroflexi	Unclassifie	OTU_5913	0.003	0.003	0.000	0.000	0.036
Bacteria	Proteobacte	Unclassifie	OTU_1182	0.003	0.003	0.000	0.000	0.038
Bacteria	Verrucomic	Subdivisio	OTU_4741	0.003	0.003	0.000	0.000	0.042
Bacteria	Bacteroidet	Unclassifie	OTU_6943	0.003	0.003	0.000	0.000	0.036
Bacteria	Unclassifie	Unclassifie	OTU_1948	0.003	0.002	0.000	0.000	0.015
Bacteria	Verrucomic	Unclassifie	OTU_9266	0.003	0.003	0.000	0.000	0.021
Bacteria	Acidobacte	Gp6	OTU_6021	0.003	0.003	0.000	0.000	0.045
Bacteria	Actinobact	Iamia	OTU_4186	0.003	0.003	0.000	0.000	0.048
Bacteria	Verrucomic	Unclassifie	OTU_6359	0.003	0.002	0.000	0.000	0.015
Bacteria	Acidobacte	Gp4	OTU_2370	0.003	0.003	0.000	0.000	0.033
Bacteria	Unclassifie	Unclassifie	OTU_6450	0.003	0.003	0.000	0.000	0.042
Bacteria	Proteobacte	Unclassifie	OTU_5012	0.003	0.003	0.000	0.000	0.026
Bacteria	Chloroflexi	Unclassifie	OTU_2094	0.003	0.002	0.000	0.000	0.019
Bacteria	Proteobacte	Unclassifie	OTU_8374	0.003	0.003	0.000	0.000	0.041
Bacteria	Bacteroidet	Unclassifie	OTU_2214	0.003	0.002	0.000	0.000	0.016
Bacteria	Actinobact	Aciditerrim	OTU_3900	0.003	0.003	0.000	0.000	0.032
Bacteria	Acidobacte	Gp6	OTU_3950	0.003	0.003	0.000	0.000	0.048
Bacteria	Proteobacte	Labilithrix	OTU_5073	0.003	0.003	0.000	0.000	0.043

Bacteria	Proteobacte	Unclassifie	OTU_3281	0.003	0.003	0.001	0.001	0.043
Bacteria	Unclassifie	Unclassifie	OTU_3566	0.002	0.002	0.000	0.000	0.024
Bacteria	Actinobacte	Unclassifie	OTU_6102	0.002	0.003	0.000	0.000	0.047
Bacteria	Armatimon	Fimbriimor	OTU_3750	0.002	0.003	0.000	0.000	0.039
Bacteria	Proteobacte	Unclassifie	OTU_2747	0.002	0.003	0.000	0.000	0.039
Bacteria	Unclassifie	Unclassifie	OTU_5014	0.002	0.002	0.000	0.000	0.024
Bacteria	Planctomyc	Unclassifie	OTU_5593	0.002	0.003	0.000	0.000	0.038
Bacteria	Proteobacte	Unclassifie	OTU_5971	0.002	0.002	0.000	0.000	0.036
Bacteria	Chloroflexi	Oscillochlo	OTU_2519	0.002	0.003	0.000	0.000	0.049
Bacteria	Acidobacte	Gp16	OTU_5152	0.002	0.002	0.000	0.000	0.024
Bacteria	Armatimon	Armatimon	OTU_6140	0.002	0.002	0.000	0.000	0.028
Bacteria	Chloroflexi	Unclassifie	OTU_2403	0.002	0.003	0.000	0.000	0.049
Bacteria	Unclassifie	Unclassifie	OTU_8341	0.002	0.003	0.000	0.000	0.044
Bacteria	Proteobacte	Unclassifie	OTU_8681	0.002	0.003	0.000	0.000	0.047
Bacteria	Unclassifie	Unclassifie	OTU_3300	0.002	0.002	0.000	0.000	0.036
Bacteria	Planctomyc	Zavarzinell	OTU_3782	0.002	0.002	0.000	0.000	0.043
Bacteria	Planctomyc	Pirellula	OTU_3886	0.002	0.002	0.000	0.000	0.038
Bacteria	Proteobacte	Hephaestia	OTU_6054	0.002	0.002	0.000	0.000	0.050
Bacteria	Unclassifie	Unclassifie	OTU_3979	0.002	0.002	0.000	0.000	0.050
Bacteria	Planctomyc	Zavarzinell	OTU_4633	0.002	0.002	0.000	0.000	0.043
Bacteria	Armatimon	Armatimon	OTU_4794	0.002	0.002	0.000	0.000	0.028
Bacteria	Proteobacte	Cystobacte	OTU_9432	0.004	0.002	0.002	0.002	0.043
Bacteria	Latescibact	Latescibact	OTU_6469	0.002	0.002	0.000	0.000	0.029
Bacteria	Unclassifie	Unclassifie	OTU_3417	0.002	0.002	0.000	0.000	0.048
Bacteria	Proteobacte	Unclassifie	OTU_2897	0.002	0.002	0.000	0.000	0.045
Bacteria	Verrucomic	Subdivision	OTU_5519	0.002	0.002	0.000	0.000	0.033
Bacteria	Proteobacte	Unclassifie	OTU_3581	0.002	0.002	0.000	0.000	0.037
Bacteria	Verrucomic	Spartobacte	OTU_6381	0.002	0.002	0.000	0.000	0.044
Bacteria	Chloroflexi	Litorilinea	OTU_4531	0.002	0.002	0.000	0.000	0.049
Bacteria	Unclassifie	Unclassifie	OTU_3164	0.002	0.002	0.000	0.000	0.043
Bacteria	Verrucomic	Unclassifie	OTU_6230	0.002	0.002	0.000	0.000	0.039
Bacteria	Firmicutes	Unclassifie	OTU_4045	0.002	0.002	0.000	0.000	0.039
Bacteria	Gemmatim	Gemmatim	OTU_2314	0.002	0.002	0.000	0.000	0.039
Bacteria	Verrucomic	Subdivision	OTU_7595	0.002	0.002	0.000	0.000	0.039
Bacteria	Planctomyc	Unclassifie	OTU_7054	0.002	0.002	0.000	0.000	0.024
Bacteria	Verrucomic	Subdivision	OTU_9149	0.002	0.002	0.000	0.000	0.024
Bacteria	Proteobacte	Unclassifie	OTU_4290	0.002	0.002	0.000	0.000	0.024
Bacteria	Planctomyc	Unclassifie	OTU_8632	0.002	0.002	0.000	0.000	0.042
Bacteria	Unclassifie	Unclassifie	OTU_3239	0.002	0.002	0.000	0.000	0.045
Bacteria	Unclassifie	Unclassifie	OTU_3916	0.002	0.002	0.000	0.000	0.043
Bacteria	Proteobacte	Unclassifie	OTU_2148	0.002	0.002	0.000	0.000	0.041
Bacteria	Proteobacte	Unclassifie	OTU_4632	0.000	0.000	0.001	0.001	0.044
Bacteria	candidate d	WPS-1_ge	OTU_2059	0.000	0.000	0.001	0.001	0.047
Bacteria	Actinobacte	Sinosporan	OTU_6470	0.000	0.000	0.001	0.001	0.047
Bacteria	Proteobacte	Unclassifie	OTU_2174	0.000	0.000	0.001	0.001	0.045
Bacteria	Unclassifie	Unclassifie	OTU_5607	0.000	0.000	0.001	0.002	0.045
Bacteria	Firmicutes	Clostridium	OTU_2990	0.000	0.000	0.001	0.002	0.048
Bacteria	Bacteroidet	Sphingobac	OTU_1409	0.000	0.000	0.001	0.002	0.045
Bacteria	Firmicutes	Clostridium	OTU_2716	0.000	0.000	0.002	0.002	0.039
Bacteria	Proteobacte	Unclassifie	OTU_4916	0.000	0.000	0.002	0.002	0.034
Bacteria	Planctomyc	Singulispha	OTU_7882	0.000	0.000	0.002	0.002	0.047
Bacteria	Planctomyc	Unclassifie	OTU_7268	0.000	0.000	0.002	0.002	0.041
Bacteria	Actinobacte	Conexibact	OTU_3337	0.000	0.000	0.002	0.002	0.034
Bacteria	Planctomyc	Unclassifie	OTU_4039	0.000	0.000	0.002	0.002	0.039
Bacteria	Proteobacte	Unclassifie	OTU_1089	0.000	0.000	0.002	0.001	0.015
Bacteria	Proteobacte	Unclassifie	OTU_9518	0.000	0.000	0.002	0.002	0.048

Bacteria	Proteobacte	Unclassifie	OTU_5793	0.000	0.000	0.002	0.002	0.037
Bacteria	Chloroflexi	Litorilinea	OTU_4373	0.000	0.000	0.002	0.002	0.037
Bacteria	Unclassifie	Unclassifie	OTU_4701	0.000	0.000	0.002	0.002	0.046
Bacteria	Unclassifie	Unclassifie	OTU_3946	0.000	0.000	0.002	0.002	0.047
Bacteria	Proteobacte	Unclassifie	OTU_4638	0.000	0.000	0.002	0.002	0.047
Bacteria	Firmicutes	Clostridiun	OTU_5192	0.000	0.000	0.002	0.002	0.043
Bacteria	Proteobacte	Aquicella	OTU_9452	0.000	0.000	0.002	0.001	0.005
Bacteria	candidate d	WPS-1_ge	OTU_926	0.000	0.000	0.002	0.002	0.040
Bacteria	Actinobact	Unclassifie	OTU_9188	0.000	0.000	0.002	0.002	0.033
Bacteria	Actinobact	Unclassifie	OTU_1569	0.000	0.000	0.002	0.002	0.016
Bacteria	Actinobact	Aciditerrim	OTU_8512	0.000	0.000	0.002	0.002	0.030
Bacteria	Planctomyc	Singulispha	OTU_3647	0.000	0.000	0.003	0.003	0.049
Bacteria	Proteobacte	Unclassifie	OTU_1036	0.000	0.000	0.003	0.003	0.039
Bacteria	Proteobacte	Parvibacul	OTU_4418	0.000	0.000	0.003	0.003	0.036
Bacteria	Unclassifie	Unclassifie	OTU_3172	0.000	0.000	0.003	0.003	0.022
Bacteria	Armatimon	Armatimon	OTU_1395	0.000	0.000	0.003	0.003	0.041
Bacteria	Firmicutes	Thalassoba	OTU_6739	0.000	0.000	0.003	0.003	0.026
Bacteria	Unclassifie	Unclassifie	OTU_5126	0.000	0.000	0.003	0.002	0.012
Bacteria	Proteobacte	Unclassifie	OTU_4157	0.000	0.000	0.003	0.003	0.023
Bacteria	Proteobacte	Unclassifie	OTU_6564	0.000	0.000	0.004	0.002	0.001
Bacteria	Verrucomi	Subdivisio	OTU_7076	0.000	0.000	0.004	0.003	0.025
Bacteria	Unclassifie	Unclassifie	OTU_1419	0.000	0.000	0.004	0.004	0.044
Bacteria	candidate d	WPS-2_ge	OTU_1795	0.000	0.000	0.004	0.004	0.049
Bacteria	Actinobact	Aciditerrim	OTU_7239	0.000	0.000	0.004	0.004	0.038
Bacteria	Chloroflexi	Ktedonoba	OTU_1383	0.000	0.000	0.004	0.004	0.041
Bacteria	Chloroflexi	Unclassifie	OTU_3215	0.000	0.000	0.004	0.004	0.034
Bacteria	Proteobacte	Nitrospirill	OTU_7835	0.000	0.000	0.004	0.004	0.049
Archaea	Euryarchae	Methanoce	OTU_1594	0.000	0.000	0.004	0.004	0.026
Bacteria	Bacteroidet	Flavitalea	OTU_815	0.000	0.000	0.004	0.004	0.043
Bacteria	Proteobacte	Acidisoma	OTU_2262	0.000	0.000	0.004	0.004	0.027
Bacteria	Planctomyc	Unclassifie	OTU_2255	0.000	0.000	0.004	0.003	0.012
Bacteria	Proteobacte	Alkanibact	OTU_4596	0.000	0.000	0.004	0.004	0.040
Bacteria	Acidobacte	Gp6	OTU_1246	0.001	0.002	0.006	0.005	0.041
Bacteria	Proteobacte	Labilithrix	OTU_9211	0.000	0.000	0.004	0.005	0.049
Bacteria	Proteobacte	Unclassifie	OTU_9226	0.000	0.000	0.005	0.004	0.030
Bacteria	Proteobacte	Unclassifie	OTU_4762	0.000	0.000	0.005	0.006	0.048
Bacteria	Proteobacte	Unclassifie	OTU_1978	0.000	0.000	0.005	0.005	0.044
Bacteria	Unclassifie	Unclassifie	OTU_1730	0.000	0.000	0.005	0.005	0.027
Bacteria	Actinobact	Unclassifie	OTU_6395	0.002	0.003	0.007	0.005	0.040
Bacteria	Armatimon	Armatimon	OTU_1361	0.005	0.004	0.010	0.005	0.043
Bacteria	Unclassifie	Unclassifie	OTU_2136	0.000	0.000	0.006	0.006	0.042
Bacteria	Candidatus	Saccharib	OTU_1845	0.000	0.000	0.006	0.004	0.008
Bacteria	Proteobacte	Unclassifie	OTU_2199	0.000	0.000	0.006	0.007	0.049
Bacteria	Armatimon	Chthonom	OTU_7409	0.000	0.000	0.006	0.006	0.033
Bacteria	Proteobacte	Unclassifie	OTU_1595	0.000	0.000	0.006	0.006	0.025
Bacteria	candidate d	WPS-2_ge	OTU_7442	0.000	0.000	0.006	0.006	0.021
Bacteria	Unclassifie	Unclassifie	OTU_1499	0.001	0.002	0.008	0.005	0.014
Bacteria	Proteobacte	Unclassifie	OTU_666	0.000	0.000	0.007	0.007	0.049
Bacteria	Firmicutes	Clostridiun	OTU_1915	0.000	0.000	0.007	0.005	0.009
Bacteria	Chloroflexi	Thermoma	OTU_2027	0.000	0.000	0.007	0.008	0.043
Bacteria	Planctomyc	Aquisphae	OTU_2139	0.001	0.002	0.008	0.004	0.001
Bacteria	Unclassifie	Unclassifie	OTU_2648	0.000	0.000	0.008	0.007	0.017
Bacteria	Proteobacte	Unclassifie	OTU_1092	0.001	0.002	0.010	0.006	0.010
Bacteria	Proteobacte	Unclassifie	OTU_1602	0.000	0.000	0.009	0.008	0.026
Bacteria	Proteobacte	Unclassifie	OTU_1181	0.001	0.002	0.010	0.009	0.043
Bacteria	Proteobacte	Unclassifie	OTU_8066	0.000	0.000	0.009	0.010	0.047

Bacteria	Proteobacte	Paraperluci	OTU_1778	0.000	0.000	0.009	0.009	0.032
Bacteria	Firmicutes	Pullulaniba	OTU_3068	0.000	0.000	0.009	0.009	0.032
Bacteria	Candidatus	Saccharibac	OTU_1757	0.000	0.000	0.010	0.008	0.011
Bacteria	Firmicutes	Unclassifie	OTU_3116	0.000	0.000	0.010	0.011	0.043
Bacteria	Unclassifie	Unclassifie	OTU_2130	0.000	0.000	0.010	0.010	0.030
Bacteria	Candidatus	Saccharibac	OTU_7465	0.000	0.000	0.010	0.010	0.029
Bacteria	Unclassifie	Unclassifie	OTU_1899	0.000	0.000	0.011	0.012	0.046
Bacteria	Proteobacte	Acidisoma	OTU_1325	0.000	0.000	0.011	0.007	0.004
Bacteria	candidate d	WPS-2_gei	OTU_1523	0.000	0.000	0.011	0.011	0.032
Bacteria	Acidobacte	Acidipila	OTU_2415	0.000	0.000	0.011	0.011	0.031
Bacteria	Unclassifie	Unclassifie	OTU_1054	0.002	0.004	0.014	0.012	0.035
Bacteria	Bacteroidet	Unclassifie	OTU_1234	0.000	0.000	0.012	0.005	0.001
Bacteria	Proteobacte	Unclassifie	OTU_6959	0.007	0.004	0.019	0.011	0.025
Bacteria	Unclassifie	Unclassifie	OTU_668	0.002	0.004	0.015	0.013	0.035
Bacteria	Unclassifie	Unclassifie	OTU_3044	0.000	0.000	0.013	0.011	0.015
Bacteria	candidate d	WPS-1_gei	OTU_730	0.003	0.004	0.016	0.009	0.005
Bacteria	candidate d	WPS-2_gei	OTU_863	0.001	0.003	0.015	0.013	0.034
Bacteria	Unclassifie	Unclassifie	OTU_1086	0.000	0.000	0.014	0.015	0.041
Bacteria	Proteobacte	Unclassifie	OTU_1142	0.001	0.002	0.015	0.015	0.041
Bacteria	Proteobacte	Acidisoma	OTU_5390	0.010	0.009	0.024	0.010	0.017
Bacteria	Bacteroidet	Arachidico	OTU_673	0.000	0.000	0.014	0.010	0.006
Bacteria	Gemmatim	Gemmatim	OTU_488	0.003	0.003	0.017	0.015	0.039
Bacteria	Unclassifie	Unclassifie	OTU_861	0.002	0.003	0.017	0.012	0.014
Bacteria	Acidobacte	Unclassifie	OTU_1393	0.000	0.000	0.015	0.017	0.048
Bacteria	Proteobacte	Unclassifie	OTU_532	0.000	0.000	0.016	0.016	0.029
Bacteria	Candidatus	Saccharibac	OTU_977	0.000	0.000	0.016	0.017	0.036
Bacteria	Acidobacte	Unclassifie	OTU_4823	0.006	0.006	0.023	0.014	0.016
Bacteria	Acidobacte	Gp3	OTU_8039	0.007	0.011	0.025	0.013	0.015
Bacteria	Unclassifie	Unclassifie	OTU_1049	0.000	0.000	0.019	0.021	0.048
Bacteria	Proteobacte	Acidisoma	OTU_605	0.004	0.006	0.023	0.020	0.045
Bacteria	Actinobact	Nocardioid	OTU_1994	0.004	0.004	0.024	0.021	0.041
Bacteria	Planctomyc	Unclassifie	OTU_1065	0.001	0.002	0.022	0.017	0.016
Bacteria	Gemmatim	Gemmatim	OTU_708	0.000	0.000	0.024	0.019	0.014
Bacteria	Unclassifie	Unclassifie	OTU_608	0.006	0.005	0.031	0.027	0.046
Bacteria	Chloroflexi	Ktedonobac	OTU_1485	0.001	0.003	0.027	0.027	0.043
Bacteria	Proteobacte	Unclassifie	OTU_2730	0.000	0.000	0.025	0.022	0.018
Bacteria	Bacteroidet	Unclassifie	OTU_452	0.002	0.003	0.028	0.021	0.016
Bacteria	Acidobacte	Gp1	OTU_5960	0.012	0.011	0.038	0.026	0.038
Bacteria	Unclassifie	Unclassifie	OTU_894	0.000	0.000	0.025	0.025	0.032
Bacteria	Acidobacte	Unclassifie	OTU_478	0.014	0.010	0.039	0.022	0.020
Bacteria	Acidobacte	Unclassifie	OTU_989	0.000	0.000	0.026	0.017	0.004
Bacteria	Proteobacte	Unclassifie	OTU_1412	0.001	0.002	0.029	0.015	0.002
Bacteria	Acidobacte	Gp1	OTU_1367	0.000	0.000	0.027	0.021	0.012
Bacteria	Chloroflexi	Unclassifie	OTU_872	0.004	0.003	0.031	0.021	0.011
Bacteria	Proteobacte	Reyranella	OTU_451	0.008	0.007	0.037	0.019	0.004
Bacteria	Proteobacte	Unclassifie	OTU_6194	0.000	0.000	0.029	0.029	0.031
Bacteria	Planctomyc	Aquisphaer	OTU_1610	0.007	0.007	0.037	0.030	0.034
Bacteria	Gemmatim	Gemmatim	OTU_241	0.019	0.020	0.050	0.028	0.029
Bacteria	Actinobact	Unclassifie	OTU_7308	0.034	0.025	0.066	0.023	0.024
Bacteria	Candidatus	Saccharibac	OTU_737	0.000	0.000	0.033	0.023	0.007
Bacteria	Proteobacte	Unclassifie	OTU_7486	0.005	0.008	0.039	0.025	0.008
Bacteria	Actinobact	Unclassifie	OTU_692	0.006	0.007	0.041	0.035	0.033
Bacteria	Actinobact	Acidimicro	OTU_569	0.005	0.005	0.041	0.039	0.046
Bacteria	Chloroflexi	Sphaerobac	OTU_405	0.009	0.010	0.047	0.024	0.004
Bacteria	Gemmatim	Gemmatim	OTU_531	0.007	0.007	0.045	0.025	0.005
Bacteria	Proteobacte	Unclassifie	OTU_616	0.006	0.007	0.044	0.019	0.001

Bacteria	Bacteroidet	Mucilaginil	OTU_552	0.015	0.020	0.053	0.037	0.035
Bacteria	Proteobacte	Unclassifie	OTU_5717	0.002	0.002	0.040	0.031	0.014
Bacteria	Acidobacte	Telmatobac	OTU_3128	0.003	0.004	0.044	0.029	0.007
Bacteria	Chloroflexi	Unclassifie	OTU_398	0.001	0.002	0.045	0.049	0.048
Bacteria	Verrucomic	Subdivision	OTU_594	0.012	0.017	0.057	0.037	0.015
Bacteria	Proteobacte	Roseiarcus	OTU_745	0.011	0.012	0.056	0.041	0.021
Bacteria	Gemmatim	Gemmatim	OTU_949	0.012	0.017	0.061	0.049	0.035
Bacteria	Chloroflexi	Ktedonoba	OTU_929	0.008	0.010	0.060	0.023	0.000
Bacteria	Proteobacte	Unclassifie	OTU_680	0.002	0.002	0.055	0.044	0.016
Bacteria	Proteobacte	Unclassifie	OTU_390	0.016	0.016	0.071	0.045	0.015
Bacteria	Proteobacte	Candidimo	OTU_287	0.008	0.008	0.067	0.045	0.010
Bacteria	Proteobacte	Unclassifie	OTU_853	0.001	0.002	0.060	0.050	0.017
Bacteria	Unclassifie	Unclassifie	OTU_290	0.003	0.006	0.062	0.060	0.035
Bacteria	Bacteroidet	Unclassifie	OTU_4221	0.001	0.002	0.061	0.066	0.047
Bacteria	Gemmatim	Gemmatim	OTU_1117	0.005	0.008	0.066	0.058	0.025
Bacteria	Proteobacte	Labilithrix	OTU_1934	0.020	0.024	0.083	0.062	0.032
Bacteria	Proteobacte	Nevskia	OTU_547	0.015	0.019	0.080	0.058	0.022
Bacteria	Acidobacte	Granulicell	OTU_140	0.055	0.032	0.120	0.042	0.006
Bacteria	Acidobacte	Gp1	OTU_5328	0.002	0.003	0.067	0.064	0.031
Bacteria	Unclassifie	Unclassifie	OTU_197	0.009	0.006	0.076	0.038	0.002
Bacteria	Proteobacte	Unclassifie	OTU_137	0.080	0.019	0.148	0.067	0.033
Bacteria	Bacteroidet	Unclassifie	OTU_644	0.015	0.022	0.084	0.039	0.002
Bacteria	Actinobact	Humibacter	OTU_5334	0.025	0.014	0.097	0.069	0.028
Bacteria	Planctomyc	Planctopiru	OTU_214	0.031	0.030	0.108	0.053	0.006
Bacteria	Proteobacte	Haliangium	OTU_395	0.025	0.016	0.105	0.075	0.026
Bacteria	Actinobact	Unclassifie	OTU_8284	0.008	0.007	0.091	0.068	0.014
Bacteria	Proteobacte	Unclassifie	OTU_351	0.004	0.006	0.089	0.064	0.010
Bacteria	Actinobact	Unclassifie	OTU_243	0.010	0.008	0.103	0.081	0.019
Bacteria	Chloroflexi	Unclassifie	OTU_183	0.000	0.000	0.093	0.083	0.020
Bacteria	Verrucomic	Subdivision	OTU_234	0.066	0.072	0.161	0.084	0.040
Bacteria	Acidobacte	Terriglobus	OTU_8499	0.009	0.008	0.104	0.098	0.037
Bacteria	Acidobacte	Gp3	OTU_442	0.017	0.018	0.118	0.093	0.024
Bacteria	Proteobacte	Unclassifie	OTU_549	0.010	0.012	0.112	0.060	0.003
Bacteria	Actinobact	Gaiella	OTU_206	0.082	0.062	0.184	0.084	0.023
Bacteria	Acidobacte	Gp14	OTU_232	0.000	0.000	0.105	0.100	0.027
Bacteria	Actinobact	Unclassifie	OTU_671	0.036	0.025	0.152	0.128	0.047
Bacteria	Proteobacte	Kerstersia	OTU_259	0.002	0.004	0.124	0.064	0.002
Bacteria	Acidobacte	Gp1	OTU_269	0.001	0.003	0.125	0.117	0.027
Bacteria	Chloroflexi	Ktedonoba	OTU_348	0.014	0.014	0.144	0.119	0.023
Bacteria	Actinobact	Acidotherm	OTU_360	0.008	0.009	0.140	0.109	0.015
Bacteria	candidate d	WPS-2_gei	OTU_186	0.015	0.017	0.155	0.146	0.039
Bacteria	Proteobacte	Unclassifie	OTU_4198	0.001	0.002	0.142	0.134	0.027
Bacteria	Proteobacte	Pseudolabr	OTU_52	0.108	0.067	0.249	0.079	0.003
Bacteria	Proteobacte	Rhizomicro	OTU_164	0.112	0.087	0.255	0.121	0.026
Bacteria	Acidobacte	Acidobacte	OTU_318	0.007	0.004	0.152	0.110	0.010
Bacteria	Gemmatim	Gemmatim	OTU_219	0.003	0.006	0.151	0.164	0.048
Bacteria	Proteobacte	Unclassifie	OTU_386	0.022	0.009	0.171	0.099	0.005
Bacteria	Actinobact	Actinospica	OTU_153	0.030	0.039	0.185	0.145	0.026
Bacteria	Proteobacte	Unclassifie	OTU_157	0.034	0.015	0.190	0.073	0.001
Bacteria	Acidobacte	Unclassifie	OTU_91	0.117	0.101	0.289	0.164	0.037
Bacteria	Unclassifie	Unclassifie	OTU_29	0.136	0.120	0.328	0.186	0.040
Bacteria	Proteobacte	Sphingomo	OTU_49	0.148	0.096	0.344	0.148	0.013
Bacteria	Acidobacte	Terriglobus	OTU_188	0.029	0.019	0.235	0.108	0.001
Bacteria	Proteobacte	Lacibacteri	OTU_156	0.004	0.005	0.219	0.158	0.009
Bacteria	Proteobacte	Mizugakiib	OTU_88	0.023	0.042	0.240	0.100	0.000
Bacteria	Proteobacte	Skermanell	OTU_129	0.008	0.010	0.228	0.186	0.016

Bacteria	Actinobact	Mycobacte	OTU_100	0.048	0.054	0.275	0.186	0.014
Bacteria	Gemmatim	Gemmatim	OTU_99	0.031	0.031	0.268	0.252	0.042
Bacteria	Bacteroidet	Unclassifie	OTU_85	0.028	0.026	0.290	0.277	0.041
Bacteria	Planctomyc	Thermogut	OTU_187	0.035	0.032	0.297	0.204	0.011
Bacteria	Acidobacte	Gp2	OTU_64	0.003	0.006	0.293	0.299	0.038
Bacteria	Acidobacte	Gp1	OTU_205	0.107	0.149	0.406	0.208	0.009
Bacteria	Candidatus	Saccharibac	OTU_116	0.042	0.046	0.341	0.249	0.016
Bacteria	Chloroflexi	Ktedonobac	OTU_1159	0.026	0.022	0.327	0.329	0.047
Bacteria	Proteobacte	Unclassifie	OTU_92	0.005	0.011	0.316	0.228	0.009
Bacteria	Proteobacte	Unclassifie	OTU_367	0.012	0.013	0.340	0.245	0.009
Bacteria	Actinobact	Unclassifie	OTU_51	0.103	0.094	0.432	0.327	0.033
Bacteria	Chloroflexi	Unclassifie	OTU_57	0.048	0.054	0.409	0.205	0.002
Bacteria	Chloroflexi	Unclassifie	OTU_112	0.071	0.076	0.435	0.387	0.042
Bacteria	Proteobacte	Unclassifie	OTU_199	0.005	0.010	0.404	0.388	0.030
Bacteria	Proteobacte	Frateuria	OTU_346	0.058	0.030	0.464	0.211	0.001
Bacteria	Unclassifie	Unclassifie	OTU_15	0.062	0.036	0.468	0.220	0.002
Bacteria	Proteobacte	Unclassifie	OTU_9131	0.056	0.066	0.473	0.401	0.028
Bacteria	Acidobacte	Gp1	OTU_26	0.184	0.135	0.616	0.274	0.004
Bacteria	Acidobacte	Gp2	OTU_39	0.031	0.031	0.497	0.382	0.014
Bacteria	Proteobacte	Unclassifie	OTU_151	0.010	0.015	0.502	0.301	0.003
Bacteria	Actinobact	Unclassifie	OTU_84	0.035	0.042	0.541	0.396	0.012
Bacteria	candidate d	WPS-1_ger	OTU_11	0.122	0.099	0.630	0.317	0.003
Bacteria	Proteobacte	Burkholder	OTU_28	0.136	0.090	0.652	0.302	0.002
Bacteria	Acidobacte	Gp3	OTU_80	0.075	0.027	0.602	0.395	0.010
Bacteria	Actinobact	Unclassifie	OTU_60	0.094	0.112	0.731	0.464	0.008
Bacteria	Acidobacte	Gp1	OTU_61	0.017	0.022	0.794	0.537	0.006
Bacteria	Acidobacte	Geothrix	OTU_18	0.132	0.070	0.913	0.479	0.003
Bacteria	Chloroflexi	Unclassifie	OTU_534	0.042	0.042	0.834	0.606	0.011
Bacteria	Bacteroidet	Unclassifie	OTU_14	0.114	0.167	1.180	1.159	0.046
Bacteria	Proteobacte	Unclassifie	OTU_5473	0.057	0.068	1.127	0.804	0.010
Bacteria	Proteobacte	Unclassifie	OTU_27	0.095	0.065	1.366	1.100	0.018
Bacteria	Proteobacte	Unclassifie	OTU_12	0.275	0.167	1.580	1.201	0.024
Bacteria	Proteobacte	Rhodanoba	OTU_5	0.331	0.284	2.909	2.134	0.015
Bacteria	Proteobacte	Unclassifie	OTU_4	0.234	0.263	4.001	2.605	0.006
Archaea	Thaumarch	Nitrososph	OTU_2	0.296	0.250	4.833	4.776	0.040
